# A critical review of potential modifiers of air pollutant associations with dementia and related outcomes

**DOI:** 10.1016/j.neuro.2026.103470

**Published:** 2026-05-22

**Authors:** JeongWon Han, Yina Li, Sangji Lee, Katie M. Lynch, Erin E. Bennett, Qi Ying, Eun Sug Park, Richard L. Smith, James D. Stewart, Eric A. Whitsel, Melinda C. Power, Xiaohui Xu

**Affiliations:** a Texas A&M University, United States; b George Washington University, United States; c University of North Carolina at Chapel Hill, United States

**Keywords:** Dementia, Alzheimer’s Disease, Neurodegenerative Diseases, Air Pollution, Cognitive, Health, Effect modification

## Abstract

**Background:**

Air pollution has been linked with poor cognitive and brain health, including Alzheimer’s Disease and Related Dementia (ADRD). However, whether specific individuals are more susceptible than others remains unclear.

**Purpose:**

We aimed to review potential modifiers of the air pollution-cognitive/brain health association and identify subgroups potentially susceptible to adverse effects of air pollution exposure.

**Methods:**

We identified published, peer-reviewed articles in the Embase and Medline databases through the Ovid and PubMed search platforms and used Covidence, an online software tool, to extract relevant data from eligible articles. We then systematically reviewed and summarized the evidence on how health behaviors, genetic/molecular factors, environmental factors, and individual factors modify the association between ambient air pollution and cognitive/brain health.

**Results:**

Seventy-four publications, out of 6372 relevant publications, met the eligibility criteria and were included in this review. Available studies suggested that race/ethnicity, geographic location, *apolipoprotein E (APOE) e*4 allele, socioeconomic status (SES), body mass index (BMI), and diet may modify the air pollution-ADRD association, and that age and education do not, although the available evidence precludes firm conclusions. Limited reporting precludes conclusions about other modifiers.

**Conclusion:**

Efforts to reduce the harmful effects of air pollution exposure should consider sensitive groups. The current literature suggests differential susceptibility to the adverse effects of air pollution on cognitive and brain health. Future studies should include standardized reporting of effect modification for the likely modifiers identified here, allowing for more robust conclusions.

## 1. Introduction

Cognitive impairment is considered a significant public health problem. Alzheimer’s Disease and Related Dementias (ADRD) are irreversible and progressive brain diseases affecting as many as 5.5 million Americans, and the number of cases of Alzheimer’s Disease is projected to triple to 14 million in 2050 (Center for Disease Control and Prevention, 2018). ADRD is the sixth leading cause of death in the United States (Shi et al., 2021a, 2021b) and is considered the primary cause of mental disability among the older population (Center for Disease Control and Prevention, 2018). Furthermore, despite global efforts to develop treatments for ADRD, no meaningful curative therapies have been identified, and only a limited number of FDA-approved medications appear to offer modest benefits (Yadollahikhales and Rojas, 2023). Therefore, current efforts to address the coming epidemic emphasize prevention strategies to delay the onset of rather than to treat ADRD.

Reducing environmental pollution may help prevent ADRD. A substantial number of studies have identified environmental risk factors, including air pollution (Chandra et al., 2022; Fu and Yung, 2020; Peters et al., 2019; Weuve et al., 2021), tobacco smoke (Chen et al., 2013), arsenic (Dani, 2010), and lead () as risk factors for impaired cognitive function and ADRD. Air pollution is of particular interest. While we have effective strategies to reduce air pollution, approximately 91% of the world’s population resides in areas with air quality below the World Health Organization’s standards (WHO, 2022).

Multiple systematic reviews suggest a link between air pollution and cognitive and brain health, although firm conclusions remain elusive due to the heterogeneity of findings (Chandra et al., 2022; Fu and Yung, 2020; Lopuszanska and Samardakiewicz, 2020; Mahfoz et al., 2021; Peters et al., 2019; Weuve et al., 2021). Although methodological limitations may contribute to heterogeneity across studies (Weuve et al., 2021), this heterogeneity may also arise from differing susceptibilities of the studied populations. None of the reviews primarily focused on identifying potential modifiers. Thus, our study aimed to conduct a systematic review to identify potential modifiers of the association between air pollution and cognitive or brain health, including ADRD, to understand whether specific subgroups are more susceptible to adverse effects of air pollution exposure.

## 2. Methods

### 2.1. Tools and data sources

We used the Preferred Reporting Items for Systematic Reviews and Meta-Analyses (PRISMA) guidelines to develop and report this systematic review (Moher et al., 2009). We registered and documented our study protocol with PROSPERO (PROSPERO 2022 CRD42022322836). Details are available at https://www.crd.york.ac.uk/prospero/display_record.php?RecordID=322836.

We searched the Medline and Embase databases via Ovid and PubMed to identify the existing literature on risk factors that modify the association between air pollution and ADRD. We utilized Covidence, an online software tool that facilitates the automation of the review process, including publication screening, full-text assessment, and data extraction, for this systematic review.

### 2.2. Literature search and selection

Our inclusion and exclusion criteria are provided in Appendix S1. Briefly, we included English-language epidemiological or population-based studies of adults aged 18 years or older, which reported results on potential modifiers of the association between long-term ambient air pollution exposure and cognitive or brain health measures. Review articles, commentaries, animal or molecular studies, conference abstracts, and non-peer-reviewed publications were excluded from our review.

Three of our team members (J.H.; Y.L.; D.L.) worked independently on study selection. First, J.H. assessed the preliminary literature search to develop the search protocol. After the search protocol was standardized (Appendix S2), a complete article search was performed, including articles available through February 1, 2024, and the identified articles were imported into Covidence and de-duplicated. Two researchers (J.H.; Y.L.) independently screened titles and abstracts to identify relevant publications and then reviewed the full texts of all potentially eligible studies reporting modification. Conflicts were resolved by discussion and consensus among researchers. We supplemented the search protocol by examining the reference lists of all articles selected for full-text review and of existing systematic reviews to identify any additional studies our search protocol may have missed.

### 2.3. Data extraction

The data from each eligible study was extracted independently by two of three team members (J.H.; Y.L.; D.L.) and included authors, exposures and unit increment (e.g., ozone; per interquartile range [IQR] increase), outcome types and measures (e.g., cognitive test; immediate recall of a 10-word list), modifiers and their strata (e.g., gender; male and female), parameter estimates from regression models including 95% confidence intervals (if provided) within strata of modifiers (e.g., hazard ratio, odds ratio, or regression coefficient, etc.).

Parameter estimates within strata of modifiers were grouped by modifier type and summarized in Tables E1–E20 and forest plots (generated only if evaluated by ≥3 studies; see Figure S2).

To judge the strength of the evidence supporting effect modification in any sample, we examined both statistical significance and differences in effect size estimates across levels of the effect modifiers to evaluate whether the factors act as key effect modifiers. For evaluating statistical significance, we extracted the p-value associated with the air pollution exposure ˟ modifier cross-product term. If that p-value was unavailable, we judged statistical significance by the absence of overlap among modifier stratum-specific 95% confidence intervals (CIs) or reports of “significance” or “non-significance”. If effect size estimates were presented only graphically across modifier levels – along with 95% CIs (as shaded areas and ranges) – without numerical point estimates, we incorporated them qualitatively to assess the magnitude of differences of effect estimates. Parameter estimates were extracted from a review of all accessible resources from eligible studies, including tables, figures, and supplemental materials. Two members (J.H.; D.L.) verified extracted effect sizes and p-values.

### 2.4. Quality and bias assessment

For each eligible study, two of three team members (J.H.; Y.L.; D.L.) independently conducted a quality and bias assessment utilizing appropriate critical appraisal checklist tools from The Joanna Briggs Institute (JBI) (Buccheri and Sharifi, 2017). To be specific, the checklist tools from the JBI are designed to evaluate the risk of bias of published studies for developing systematic review literature. Depending on the study designs of the eligible studies, three critical appraisal tools were used: the Assessment of Risk of Bias for Analytical Cross-sectional Studies, the Checklist for Case-Control Studies, and the Checklist for Cohort Studies. Details of each critical appraisal tool can be found elsewhere (Joanna Briggs Institute). Each checklist tool presents a series of appraisal questions, followed by an overall appraisal decision, and yields a risk of bias assessment categorized as high (1–3 “yes” scores), moderate (4–6 “yes” scores), or low (7 or more “yes” scores). Scores on the individual items of the assessment tools are provided for all eligible articles in the Supplemental Materials (Table S1–3).

### 2.5. Data synthesis

We grouped evidence around potential modifiers into four classes: individual (gender, employment status, race/ethnicity, age, education, BMI (in kg/m^2^), geographic location, SES, comorbidity, etc.), health behaviors (physical activity, dietary diversity, smoking status, and alcohol drinking status), genetic/molecular (*APOE* e4, telomere length, estrogen receptor beta polymorphisms, etc.), and environmental (neighborhood stress, noise, cooking fuel, and ventilation in indoor cooking). As a meta-analysis was not feasible in our study due to variations across the primary publications, such as heterogeneous definitions of risk factors and/or effect modifiers, we summarized the results qualitatively by inspecting tables and figures.

## 3. Results

### 3.1. Overview of eligible studies

Our literature search on MEDLINE and EMBASE using the PubMed and Ovid platforms identified 4617 and 5666 articles, respectively, preceded by a preliminary search by the study authors that identified 18 articles. The Covidence platform automatically removed 3929 duplicates, yielding 6372 articles for screening review. After screening the title and abstract, we excluded 6083 irrelevant studies, yielding 289 unique studies for full review (Figure S1). Ultimately, 74 studies met our eligibility criteria for inclusion in this review (Ailshire et al., 2017; Ailshire and Walsemann, 2021; Ailshire and Crimmins, 2014; Alemany et al., 2021; Cacciottolo et al., 2017; Carey et al., 2018; Cerza et al., 2019; Chen et al., 2021; Chen et al., 2022; Chen et al., 2022; Chen et al., 2017; Chen et al., 2020; Christensen et al., 2022; Cleary et al., 2018; Colicino et al., 2016; Colicino et al., 2014; Colicino et al., 2017; Crous-Bou et al., 2020; Cullen et al., 2018; de Crom et al., 2022; Fehsel et al., 2016; Gale et al., 2020; Gao et al., 2022; Gatto et al., 2014; Grande et al., 2021; He et al., 2022; Hedges et al., 2020; Hedges et al., 2019; Hu et al., 2023; Hu et al., 2022; Iaccarino et al., 2021; Kim et al., 2019; Kulick et al., 2020; Lee et al., 2022; Lee et al., 2019; Li et al., 2022; Li et al., 2022; Loop et al., 2013; Lucht et al., 2022; Ma et al., 2022; Mortamais et al., 2021; Nunez et al., 2021; Oudin et al., 2019; Parra et al., 2022; Power et al., 2013; Ran et al., 2021; Ranft et al., 2009; Salinas-Rodríguez et al., 2018; Schikowski et al., 2015; Semmens et al., 2022; Shaffer et al., 2021; Shi et al., 2021a; Shi et al., 2020; Shi et al., 2023; Shin et al., 2019; Sun and Gu, 2008; Tallon et al., 2017; Tzivian, Dlugaj, Winkler, Hennig, et al., 2016; Tzivian, Dlugaj, Winkler, Weinmayr, et al., 2016; Tzivian et al., 2017; Wang et al., 2020; Wang et al., 2022; Wellenius et al., 2012; Wu et al., 2015; Wyatt et al., 2023; Yang et al., 2022; Yao et al., 2021; Younan et al., 2021; Yu et al., 2020; Yuchi et al., 2020; Zhang et al., 2023; Zhu et al., 2022).

Most identified studies were cohort studies (55 of 74), and the largest proportion (i.e., 30 of 74) reported associations between PM or NO/NOx and cognitive test scores or their changes. Across the eligible studies, 46% were conducted in North America, 30% in Europe, 16% in Asia, and 2% in other regions (South America and worldwide). The median publication year was 2020. Additional information about each study is available in Appendix S3.

#### 3.1.1. Risk of bias

Overall risk of bias across the eligible studies was low. These studies used appropriate statistical analyses and clearly identified confounding factors. However, for cohort studies, a substantial number of studies did not provide information on 1) whether the participants were free of diseases at the time of study start, 2) completeness of follow-up, or 3) strategies to address incomplete follow-up. Among cross-sectional studies, several studies did not clearly define the inclusion criteria of the study population. Despite these limitations, which may have introduced uncertainty in the evaluation of risk of bias, the pooled estimates support the robustness across the studies. A complete list of bias assessments is provided in Tables S1–S3.

### 3.2. Modifiers

Table S4 provides information on which studies considered which modifiers of the air pollution-cognitive/brain health. Out of 74 studies, 74% reported individual modifiers, 35% reported one or more genetic/molecular modifiers, 28% reported one or more health behaviors modifiers, and 7% reported one or more environmental factors as effect modifiers. Of the individual modifiers, studies most frequently tested age (50%) and gender (49%).

#### 3.2.1. Individual characteristics

Age and gender were the most commonly considered potential modifiers, while few studies (7%) considered modification by race/ethnicity. Of the 37 studies that examined age as a modifier, only 7 reported statistically significant modifications (Table E1). Among these studies, whether stronger air pollution-cognitive impairment associations were found in older or younger age groups varied. For example, according to a study by Lee and colleagues, the effect of PM_2.5_ exposure was more pronounced on cognitive function among older groups (aged 65–75 or >75 years)(beta: −0.09, 95% CI: −0.13 - −0.05 or −0.12, 95% CI:−0.19 - −0.05) than the younger group (50–65 years)(beta: −0.27, 95% CI: −0.44 - −0.11, P-*interaction*<0.00001), though the pattern of the finding was inverted with PM10. In contrast, according to Yao et al., the association with a distance-to-roadway proxy for air pollution exposure was more pronounced among participants < 80 years (OR=1.50; 95% CI:1.22, 1.84) than among those 80 years or older (OR=1.13; 95% CI:1.01, 1.28; *P-interaction: 0.008*). Among 36 studies that examined gender as an effect modifier, significant effect modification was reported in only 9 studies (Hedges et al., 2019; Lee et al., 2022; Li et al., 2022; Shaffer et al., 2021; Shi et al., 2021a; Shi et al., 2020; Shin et al., 2019; Tzivian, Dlugaj, Winkler, Hennig, et al., 2016) (Table E2). Most of these studies reported that the risk of poor cognitive or brain health associated with exposure to air pollution was higher among women than men. For example, a stronger correlation was reported in females than in males in a study of PM2.5 and Global Cognitive Score (GCS) (Tzivian, Dlugaj, Winkler, Hennig, et al., 2016). Similarly, more significant effects of air pollution exposure, including nitrogen oxides and nitrogen dioxide on the left and/or right hippocampal volume(s), were found for women (β for an interaction term of air pollutant and gender: −2.58** (p <0.01) (nitrogen dioxides on left Hippocampal Volume) and −1.33** (nitrogen oxides on left Hippocampal Volume), and −0.98*(nitrogen oxides on right Hippocampal Volume)) than for men (Hedges et al., 2019). Among 20 studies, 4 revealed significant modifications by education. However, whether higher education appears to mute or exacerbate the effect of air pollution on cognitive and brain health differs across these studies (Table E3). For example, according to Wellenius et al., 2012, individuals living in Boston who had attended at least some college and lived near a major roadway showed greater risk (OR=1.54, 95% CI: 1.1 – 2.17) of cognitive impairment compared to those who received high school education or less (OR=0.86, 95% CI: 0.66 – 1.12) (P-*interaction*: 0.007) (Wellenius et al., 2012). On the other hand, a study revealed that an adverse PM_2.5_-AD association was found in groups that received only literacy/primary school education (HR=1.13/1.1; 95% CI=1.09 – 1.2/ 0.99 – 1.15) compared to those who attended junior high/senior high school (HR=0.98/ 0.86; 95% CI=0.77 – 1.24/ 0.26 – 2.81) (Yang et al., 2022). Out of 14 studies that discussed other measures of socioeconomic status (SES) as a modifier, seven studies showed significantly stronger associations with cognitive outcomes in lower versus higher SES groups (Cerza et al., 2019; Christensen et al., 2022; Li et al., 2022; Shi et al., 2021a; Shi et al., 2023; Shin et al., 2019; Sun and Gu, 2008) (Table E4).

Of the 5 studies, 3 demonstrated significant modifications by race/ethnicity (Shi et al., 2021a, 2020, 2023) (Table E5). Among these studies, conflicting results were reported regarding which race/ethnicity group experienced the greatest impact of air pollution. However, studies reported Hispanic populations were at a lower risk of NO2, PM2.5, and PM10 exposure on cognitive health compared to White-non-Hispanic and Black-non-Hispanic groups, with one study presenting a relevant stratified analysis (Kulick et al., 2020). Six of 15 studies showed a significant modification of air pollution-cognitive outcome associations by measures of urbanicity or population density (Gao et al., 2022; Lee et al., 2019; Li et al., 2022; Shi et al., 2021a; Shi et al., 2020; Shin et al., 2019) (Table E6). Two papers that used the U.S. study population reported stronger effects of PM2.5 on cognitive health in urban and high-population-density areas relative to rural and low-population-density areas. Lee and colleagues reported that people living in metropolitan areas (HR=1.05, 95% CI=1.05 – 1.054) were more susceptible to PM2.5 exposure than those living in rural areas (HR=1.036, 95% CI=1.03 – 1.04) (Lee et al., 2019) while Shi and colleagues found consistent reporting that American populations living in higher population density areas (Q3 or Q4) exposed to PM_2.5_ had a statistically higher chance of getting ADRD compared to those living in lower population density (Q2 or Q1) (Shi et al., 2020). In contrast, Shin et al. (2019) reported that associations between PM2.5 and performance on domain-specific cognitive testing, including the frontal assessment battery and digit span-forward test, were stronger among rural than suburban/urban residents in South Korea.

Among 12 studies, only one reported statistically significant differences in air pollution-cognitive/brain health associations by BMI groups (Chen et al., 2015) (Table E7). However, most observed stronger effects of air pollution exposure in the higher versus the lower BMI groups, regardless of the statistical significance of their stratified analyses. For example, According to Women’s Health Initiative Memory Study conducted by Chen and colleagues, PM_2.5_ was associated with statistically lower brain volumes of parietal white matter (β=−2.21, 95% CI= −2.8 – −1.62) in obese women (BMI(≥30) than in overweight women (BMI=25–29)(β=0.–0.47, 95% CI= −1.02 – 0.08) and women at even lower BMI(<25) (β=0.61, 95% CI= −0.02 – 1.24)(P*interaction*<0.01) (Chen et al., 2015). Similarly, Chen et al. (2015) reported that regional (e.g., frontal, parietal, and temporal) white matter volumes were lower in women with higher BMI (≥30 vs. <25) (Pinteraction >0.1), although the difference was not statistically significant. In addition, two papers conducted in Germany by the same research group examined BMI in cross-sectional studies, (Tzivian, Dlugaj, Winkler, Hennig, et al., 2016; Tzivian, Dlugaj, Winkler, Weinmayr, et al., 2016) however, they identified a similar pattern of stronger PM_2.5_- cognitive impairment associations at higher BMI.

Neither study (Hu et al., 2022; Yang et al., 2022) examining the modifying effect of marital status found evidence to support modification (Table E8). Employment status was considered as a modifier in 3 articles, and none yielded statistical significance (Table E9).

Five of 22 studies found a significant modification of air pollution-associated cognitive function by physical or mental health comorbidities (Chen et al., 2017; Gale et al., 2020; Grande et al., 2021; Hedges et al., 2020; Tallon et al., 2017) (Table E10). Individual studies reported on different comorbidities. Among these studies, a substantial amount of literature has investigated cardiometabolic diseases as existing conditions. For example, a study by Carey et al. included ischemic heart disease, stroke, heart failure, and diabetes as the comorbidity factors (Carey et al., 2018). Some studies focused on depression, diabetes, hypertension, hypercholesterolemia, embolism, and heart disease (infarction, angina pectoris, heart failure) (Salinas-Rodríguez et al., 2018), and another study conducted by Hedges and colleagues used overall self-rated health (survey to answer one of the following; excellent (1), good (2), fair (3), poor (4), do not know or prefer not to answer) (Hedges et al., 2019). There was also substantial variation in the presence and direction of modification by specific comorbidities where reported. The risk of exposure to air pollution was greater in the subgroup having depression and this pattern was consistent across every study that examined depression as a potential effect modifier, despite an absence of statistical significance (Tallon et al., 2017; Tzivian, Dlugaj, Winkler, Weinmayr, et al., 2016; Yao et al., 2021).

#### 3.2.2. Genetic/molecular indicators

Genetic/molecular indicators were the second most commonly tested class of modifiers. A total of 26 studies examined potential effect modifications (Alemany et al., 2021; Cacciottolo et al., 2017; Chen et al., 2022; Chen et al., 2020; Cleary et al., 2018; Colicino et al., 2016; Colicino et al., 2014; Colicino et al., 2017; Crous-Bou et al., 2020; de Crom et al., 2022; Fehsel et al., 2016; Kulick et al., 2020; Li et al., 2022; Ma et al., 2022; Mortamais et al., 2021; Oudin et al., 2019; Parra et al., 2022; Power et al., 2013; Schikowski et al., 2015; Semmens et al., 2022; Shaffer et al., 2021; Tzivian, Dlugaj, Winkler, Hennig, et al., 2016; Tzivian, Dlugaj, Winkler, Weinmayr, et al., 2016; Wang et al., 2022; Wu et al., 2015; Zhang et al., 2023) and seven showed statistical significance by genetic/molecular factors (Alemany et al., 2021; Colicino et al., 2017; Crous-Bou et al., 2020; Fehsel et al., 2016; Kulick et al., 2020; Li et al., 2022; Schikowski et al., 2015) (Table E12).

Among these studies that examined *APOE* e4 as an effect modifier (Alemany et al., 2021; Cacciottolo et al., 2017; Chen et al., 2022; Chen et al., 2020; Cleary et al., 2018; Crous-Bou et al., 2020; de Crom et al., 2022; Kulick et al., 2020; Li et al., 2022; Ma et al., 2022; Mortamais et al., 2021; Oudin et al., 2019; Parra et al., 2022; Schikowski et al., 2015; Semmens et al., 2022; Shaffer et al., 2021; Tzivian, Dlugaj, Winkler, Hennig, et al., 2016; Tzivian, Dlugaj, Winkler, Weinmayr, et al., 2016; Wang et al., 2020; Wu et al., 2015), most reported a higher risk of air pollution-associated cognitive impairments in the *APOE* e4 carriers compared to the non-carriers, although some were not statistically significant. Relatively few other genetic or molecular markers were examined in multiple studies. Significant effect modifications were observed for mitochondrial DNA haplogroups (Colicino et al., 2014), telomere length (Colicino et al., 2017), and C-reactive protein (Colicino et al., 2017), as well as estrogen receptor alleles (Fehsel et al., 2016).

#### 3.2.3. Health behavior factors

Six eligible studies examined dietary factors as potential effect modifiers encompassing the MIND-like diet (Chen et al., 2021), diverse diet (Yao et al., 2021), folate/B6/B12 supplementation (C. Chen et al., 2022), eating fish (He et al., 2022), plant-based diet (Zhu et al., 2022), and ginko *biloba* treatment (Semmens et al., 2022) (Table E11). Three of 6 studies identified statistically significant modification by dietary factors, with PM_2.5_ as a primary air pollutant. (Chen et al., 2021; Chen et al., 2022; Zhu et al., 2022). Among these 3 studies that showed less harmful air pollution- cognitive or brain health associations in healthy eating groups relative to their counterparts, the findings were presented using hazard ratio for the risk of incident all-cause dementia (Chen et al., 2022) and poor cognitive function (Zhu et al., 2022) while Chen et al., 2021 employed regression model coefficients on brain magnetic resonance imaging (MRI) scans to measure the brain volumes. Seven studies analyzed the potential differential effects of air pollution on impaired cognitive function by physical activity (Cullen et al., 2018; Gao et al., 2022; Lee et al., 2022; Shin et al., 2019; Tallon et al., 2017; Wang et al., 2020; Yao et al., 2021) (Table E13). Both studies reporting significant effect modification had consistent findings about which subgroup is more vulnerable to air pollution exposure on cognitive outcomes (Lee et al., 2022; Shin et al., 2019). The study conducted by Shin and colleagues (Shin et al., 2019) showed a significantly greater effect of PM2.5 on word list recognition function among those who were physically inactive compared to those who were physically active. However, other two studies reported that physically active group was more susceptible to air pollution effect on cognitive health outcome (Gao et al., 2022; Yao et al., 2021).

Fifteen studies examined smoking status as a modifier (Ailshire and Crimmins, 2014; Carey et al., 2018; Chen et al., 2022; Gao et al., 2022; Kulick et al., 2020; Lee et al., 2022; Li et al., 2022; Power et al., 2011; Salinas-Rodríguez et al., 2018; Shin et al., 2019; Tallon et al., 2017; Tzivian, Dlugaj, Winkler, Hennig, et al., 2016; Tzivian, Dlugaj, Winkler, Weinmayr, et al., 2016; Wang et al., 2020; Yao et al., 2021) where six of them reported being statistically significant (Ailshire and Crimmins, 2014; Chen et al., 2022; Lee et al., 2022; Li et al., 2022; Shin et al., 2019; Tzivian, Dlugaj, Winkler, Weinmayr, et al., 2016) (Table E14). Four studies indicated a more pronounced effect of air pollution in current or former smokers compared to non-smokers (Ailshire and Crimmins, 2014; Li et al., 2022; Shin et al., 2019; Tzivian, Dlugaj, Winkler, Weinmayr, et al., 2016) and two indicated an inverted association (Chen et al., 2022; Lee et al., 2022).

For example, one study showed that the effect of PM2.5 exposure on cognitive impairment was attenuated in the non-smoking group (OR: 1.01, 95% CI: 0.85 – 1.21) compared to former or current smokers (OR: 1.39, 95% CI: 1.12 – 1.71, P-*interaction*: 0.02) (Tzivian, Dlugaj, Winkler, Weinmayr, et al., 2016).

Furthermore, among 8 studies (Gao et al., 2022; Lee et al., 2022; Salinas-Rodríguez et al., 2018; Shin et al., 2019; Tzivian, Dlugaj, Winkler, Hennig, et al., 2016; Tzivian, Dlugaj, Winkler, Weinmayr, et al., 2016; Wang et al., 2020; Yao et al., 2021), four indicated significantly differential effects of air pollution by drinking status (Lee et al., 2022; Shin et al., 2019; Tzivian, Dlugaj, Winkler, Hennig, et al., 2016; Tzivian, Dlugaj, Winkler, Weinmayr, et al., 2016) (Table E15). Three studies reported that the adverse air pollution effect was stronger in the current/former drinking group compared to the never drinking group (Gao et al., 2022; Shin et al., 2019; Yao et al., 2021), whereas five other studies reported stronger effects among never drinkers.

#### 3.2.4. Environmental factors

Relatively few studies investigated environmental modifiers. One study evaluated whether neighborhood stressors can modify cognitive function with exposure to air pollution. Ailshire et al., 2017 found more adverse effects of PM_2.5_ exposure on cognitive decline among adults (≥55 years old) in the US living in high-stress neighborhoods compared to those exposed to comparatively low neighborhood stressors (Ailshire et al., 2017) (Table E16). Two studies reported that the types of cooking fuel used at home (Table E17) and ventilation during indoor cooking at home acted as modifiers of outdoor air pollution exposure (Table E18), with inconsistent results (Salinas-Rodríguez et al., 2018; Yao et al., 2021). Two other studies (Tzivian, Dlugaj, Winkler, Weinmayr, et al., 2016; Tzivian et al., 2017) conducted by the same study group suggested that the air pollution – cognitive health relationship was stronger in the subpopulation who were exposed to higher noise (≥60 dB) compared to the low noise group (<60 dB) (Table E19). Few studies also examined other factors as potential modifiers, including exposure to traffic (Ranft et al., 2009), exposure to PM_2.5_ (Gao et al., 2022), user behavior, focusing on habitual use of the app during gameplay (Wyatt et al., 2023), and amyloid status (Alemany et al., 2021) (Table E20, E21).

## 4. Discussion

To our knowledge, this study is the first systematic review to summarize the findings on potential modifiers of the associations between air pollution and ADRD, and between air pollution and cognitive decline/impairment. We included 74 studies that examined individual characteristics, genetic/molecular factors, health behaviors, environmental and other factors as potential effect modifiers. Individual and genetic risk factors as potential modifiers have been mainly studied. In addition, we noted that a few studies considered other ambient air pollutants (Gao et al., 2022; Ranft et al., 2009) and other markers of brain or cognitive health (Alemany et al., 2021) as the modifiers. Most potential modifiers encountered heterogeneity in exposure assessment, outcome definitions, and modifier parameterization, limiting our ability to draw firm conclusions about whether these factors can be considered definite potential effect modifiers. Therefore, we summarized the findings from eligible studies and presented the list of key modifiers based on our analytic approach in 2.3 Data Extraction above.

### 4.1. Individual-level modifiers

Only a tiny proportion of studies on age (7 out of 37), gender (9 out of 37), or education (4 out of 20) reported significant differences across groups. Overall patterns across eligible studies suggested that air pollution had stronger effects on cognition in the older age group than in the younger group. However, the available evidence was insufficient to conclude this age factor as a key effect modifier in our review. Furthermore, age as an effect modifier should be interpreted with caution due to differences in study eligibility criteria, particularly heterogeneity in the age category below 65 years, even though the age cutoff points across studies are the same.

For the gender variable as a potential modifier, adverse associations were generally stronger in women than in men. Women may be at greater risk of the air pollution-ADRD relationship than men due to higher cerebral blood flow in women, which could lead to differential susceptibility to environmental toxins such as air pollution (Cosgrove et al., 2007). However, the difference in effect estimates when comparing males and females was small. Therefore, we did not classify gender as a key effect modifier in this review, despite the relatively large number of studies reporting statistically significant modifying effects by gender.

We also did not include education as a key modifier of the air pollution-ADRD relationship, mainly because different cut-off points were used across multiple eligible studies. Additionally, observed effect modification by education might be due to confounding or a three-way interaction (i.e., SES).

Most studies (9 out of 14) did not provide effect size estimates for the SES variable. Although heterogeneity existed in the metrics used to measure SES, half of the studies reported significant SES modifications, and the air pollution-cognition/brain health associations were generally stronger in low-SES than in high-SES groups. Low-SES groups are more likely to experience unhealthy behaviors such as eating low-quality foods or having less opportunity to receive cognitive-stimulating experiences, including higher education, which are all known to exacerbate cognitive health (Zhao, 2025).

For the race/ethnicity factor as a potential modifier, the studies consistently suggested that the Hispanic group is less likely to be affected by air pollution, indicating that race/ethnicity might be an important effect modifier, although findings varied by which racial group was most vulnerable to air pollution effects. Depending on the types of air pollution or the outcomes of diseases, studies reported conflicting results on which group was at the greatest risk, between White and Black.

Geographic location may be an important factor as most articles reporting stratified effect estimates suggested that people living in urban areas experienced greater air pollution effects than non-urban residents, regardless of statistical significance. Indeed, urban residents are more likely to experience poor air quality due to high levels of air pollutants (Strosnider, 2017).

While not all selected studies reported significant effect modifications of BMI, point estimates from stratified analyses were generally consistent, with stronger air pollution-cognitive/brain health associations in those with higher BMI. Effect modification by BMI may be driven by effects on inflammation, which, in turn, increases the risk of poor cognitive and brain health (Maurer et al., 2026). Previous research has provided evidence that neuroinflammation caused by obesity harms brain structures and leads to cognitive impairment (Guillemot-Legris and Muccioli, 2017). Furthermore, although most studies reported small differences in effect size estimates across BMI subgroups, population-level impacts could be clinically meaningful, given the rapidly increasing prevalence of obesity worldwide (Khaleghi et al., 2025).

The small number of studies that considered marital status or employment status as an effect modifier limits our ability to draw firm conclusions about differential susceptibility across categories.

Among studies investigating comorbidity factors, diabetes and hypertension were the most commonly considered potential modifiers, although the findings did not yield a homogeneous summary. While most of the comorbidity factors did not exhibit consistent association, depression appeared to have consistent findings across all eligible literature (Tallon et al., 2017; Tzivian, Dlugaj, Winkler, Hennig, et al., 2016; Tzivian, Dlugaj, Winkler, Weinmayr, et al., 2016; Yao et al., 2021), which employed the same measure, the CES-D scale, for the diagnosis of depression. Moreover, all findings consistently suggested that depression groups were more susceptible to the air pollution effects on cognitive health compared to non-depression groups. However, their stratified effect measures were neither statistically significant nor substantially different.

### 4.2. Genetic/molecular indicators

Collectively, the association between air pollution and cognitive or brain health was stronger among carriers of the APOE e4 allele, the strongest genetic risk factor for sporadic Alzheimer’s disease (Liu et al., 2013). A previous review study summarizing air pollution - neurological diseases associations examined a list of gene-air pollution interactions on cognitive outcomes (Genc et al., 2012) and reported an increased effect of air pollution in carriers of the APOE e4, which is consistent with our results. Many studies suggested the imbalance between the production and removal of amyloid-β (aβ) peptide is one of the major contributors to the injury of synapses which can lead to dementia, AD, and neurodegenerative diseases and apolipoprotein E (APOE) has been known as a regulator of lipid homeostasis APOE e4 may alter inflammatory and antioxidant processes in air pollution – AD pathogenesis, promoting carriers of one or more risk alleles to have less susceptibilities with exposures to air pollution (Shaffer et al., 2021). Relatively few other genetic or molecular indicators were examined. Further studies will be necessary to draw firm conclusions about the impact of other genetic or molecular risk factors on effect modification.

Despite evidence that persons with APOE e2 have a lower risk of Alzheimer’s disease (Chartier-Hariln et al., 1994; Corder et al., 1994), whether the effect of air pollution on cognitive or brain health is lessened in this group relative to wild type is unknown. But none of those selected studies have investigated this relationship. Future studies might also consider investigating other gene candidates related to ADRD susceptibility, such as *GSTP1*, as radical scavenger roles (Morales et al., 2009) or oxidative stress pathway genes, including Glutathione S-transferase Mu 1(*GSTM1*), *GSTP1*, NAD(P)H dehydrogenase quinone 1 (*NQO1*), Tumor necrosis factor (*TNF*), and Toll-like receptor 4 (*TLR4*) (Romieu et al., 2010).

### 4.3. Health behaviors-level effect modifiers

Relatively few studies reported the effect modification by physical activity. As physical activity increases inhalation, outdoor exercise might amplify the deleterious effects of air pollution on cognitive function by increasing uptake of harmful pollutants in ambient air (Panis et al., 2010). Despite regular physical activity promoting cardiovascular function and antioxidant stress processes (Zhang et al., 2024), which may offset the adverse effects of inhaling pollutants in the polluted outdoor environment on ADRD, further investigation is warranted to determine this (Yao et al., 2021). We found fairly consistent evidence of a beneficial modifying effect of a healthy diet on air pollution-ADRD associations among the small number of studies examining this modifier. The protective effect of plant-based eating habits might be explained by high levels of antioxidants, such as polyphenols (Zhang and Tsao, 2016), and vitamins (Suh et al., 2020), which may reduce oxidative stress and inflammation in the central nervous system induced by air pollution exposure. Although a modest number of studies considered effect modification by smoking or alcohol, the proportion suggesting either evidence was limited, and results were inconsistent.

### 4.4. Environmental modifiers

While relatively few studies considered environmental modifiers, these deserve further attention. Neighborhood stressors may be of interest, given prior evidence that they might modify the effects of other pollutants, such as lead exposure, on cognition (Glass et al., 2009). Indoor air pollution and noise are often elevated in places with high ambient air pollution and may have synergistic effects, implying that the adverse effect of air pollution on ADRD is amplified by living in a high-indoor air-pollution or noise environment.

Given that multiple air pollutants have plausible, related mechanisms hypothesized to affect cognitive or brain health, they may have synergistic effects on risk. Similarly, air pollution may affect cognitive and brain health at a specific point in the disease process or only for a specific underlying disease.

### 4.5. Other modifiers

A handful of studies considered alternative ambient air pollutants or alternative markers of cognitive and brain health as modifiers; however, they warrant further attention.

### 4.6. Strengths and limitations

This review has several strengths. It is the first systematic review summarizing potential non-genetic modifiers of ambient air pollution-cognition/brain health associations. Our standardized study protocol was registered on PROSPERO and relied on a systematic search of PubMed and MEDLINE for all articles reporting on the main effect, which were then reviewed for discussions of effect modification. In contrast to an approach that requires effect modification to be identifiable from the title, abstract, or keywords, this approach ensures a comprehensive examination of effect modification reporting, regardless of whether it is part of the study’s significant conclusions. Our review also has some limitations. While many authors have reported on effect modification, even when it is absent, we cannot know the extent to which they have chosen not to report non-significant effect modification. We expect that any publication bias present would skew results towards suggesting effect modification, even when none exists, due to preferential reporting. As another reporting issue, multiple hypothesis testing may occur when conducting numerous tests, leading to inflated Type I error rates. As the number of tests increases within each stratum of the effect modifiers, the possibility of obtaining at least one spurious result also grows. For instance, if an effect modifier has three categories (namely, 1, 2, and 3), pairwise comparisons of effect estimates (1 vs. 2, 2 vs. 3, and 1 vs. 3) can introduce multiple testing bias. In such cases, an apparently significant difference between strata may arise by chance because of inflated Type I error, leading to a false inference of effect modification. In addition, even when effect modification was reported, effect sizes were not always reported, limiting our ability to provide quantitative summaries or to understand when groups of studies reported similar modifications, even when individual studies did not find statistical support for effect modification and concluded differences across groups. Because individual studies are likely underpowered to detect effect modification, this may lead to a false conclusion that there is no evidence of effect modification when the collective evidence would suggest otherwise if all numbers were known. Related to this, we could not conduct meta-analyses due to the limited number of studies reporting numerical data and the substantial heterogeneity in exposures, outcomes, and the categorization or parameterization of modifiers. Although the stratum-specific effect sizes are not statistically significantly different, some modifiers may be noteworthy when identifying susceptible populations. For example, for comorbidities, only 5 studies reported statistical significance out of 22 studies (that attempted to investigate the differential susceptibility of air pollution exposures in subgroups varied by comorbidities status), however, a considerable number of studies showed substantial differences in effect size estimates although that are not statistically different (Chen et al., 2015; Crous-Bou et al., 2020; Yu et al., 2020). For example, one study (Yu et al., 2020) reported that for those populations who were exposed to high levels of traffic-related NO_x_ (≥3.44 ppb) having hyperglycemia or low HDL-cholesterol compared to those groups who were exposed to the same level of NO_x_ but not having hyperglycemia or low HDL-cholesterol, the hazard ratios were 2.4 (95% CI: 1.41, 4.0) vs. 1.1 (0.57, 2.2), and 2.5 (1.4, 4.3) vs. 1.0 (0.58, 1.8), respectively. This finding may reflect limited statistical power, indicating meaningful heterogeneity despite the absence of statistical significance. Similar evidence was observed for the smoking status (Tallon et al., 2017; Tzivian, Dlugaj, Winkler, Hennig, et al., 2016). According to Tzivian et al., the magnitude of effects of PM2.5 per IQR increase was substantially different on cognitive function, assessed by the Chicago Cognitive Function Measure, depending on smoking status (coefficients for current and non-smokers: −0.04 [−0.34, 0.26] and −0.86 [−1.55, −0.17], respectively). Further information about the difference in effect size estimates is presented in the forest plot formats (Figure S2).

Furthermore, the heterogeneous effects of air pollution may be attributable to other factors. For example, when studies employing different outcome measures were included in the comparison for the selected effect modifier, the comparisons between studies should be interpreted with caution because the underlying mechanisms of the air pollution-outcome relationship might differ.

A few potential reasons for the conflicting findings across the selected studies were the differences in 1) study designs, 2) variation and resolution of exposure assessment, 3) definitions of outcome (cognitive scores, incident dementia, brain volumes detected by MRI), 4) thresholds to classify the susceptible groups by modifiers, and 5) statistical methods.

## 5. Conclusions and future directions

This review systematically reviewed potential effect modifiers of air pollution on ADRD-related outcomes. Given the robust evidence that ongoing AD and inflammatory processes heighten susceptibility to air-pollution-related cognitive decline, public health efforts should prioritize reducing pollutant emissions and targeting the mitigation of ADRD burdens attributable to air pollution among high-risk populations. The limited evidence suggested potential effect modifiers, including race/ethnicity, geographic location, APOE e4 allele, SES, BMI, and diet. However, firm conclusions remain elusive for most effect modifiers, in part due to inconsistent consideration and reporting, as well as heterogeneity in exposures, outcomes, and parameterization of modifiers. Standardized reporting of effect modifiers of interest to the field and standardized categorization of modifiers would allow for stronger conclusions, provided authors are sufficiently cautious in their interpretation in any individual study. Several non-traditional modifiers also deserve further attention, including markers of indoor air pollution, noise, and other ambient exposures.

## Supplementary Material

List of Tables

Supplementary Materials 02

Supplementary materials 01

## Data Availability

No data was used for the research described in the article.

## Appendix A. Supporting information

Supplementary data associated with this article can be found in the online version at doi:10.1016/j.neuro.2026.103470.
