# Supplementary material for: A critical review of potential modifiers of air pollutant associations with dementia and related outcomes
Source: Neurotoxicology. Author manuscript; Available in PMC 2026 Jul 7. (PMC13339730; doi:10.1016/j.neuro.2026.103470)
Supplement: List of Tables [file NIHMS2188061-supplement-List_of_Tables.docx]

- .
- Table E1: Effect size by potential effect modifier - Age (Significant studies: 7/37)

| **Study** | | | **Effect Modifier** | | | **Outcome** | | | **Air**  **Pollutant** | **Exposure**  **Level** | **Types of Effect** | **Effect Size (A : Main Effect by Exposure)** | **Effect Size (C: Effect by Interaction term)** | **Effect Size  Estimate*** (A+C)** | **95% CI**s** | | **Direction of Effect Modifier Measure** | **p-value  for  interaction terms** | **Statistical Significance (S/NS**)** |
| --- | --- | --- | --- | --- | --- | --- | --- | --- | --- | --- | --- | --- | --- | --- | --- | --- | --- | --- | --- |
|  |  |  | **Age** | | | **Outcome Measure Method** | | **Outcome Variable** |  |  |  |  |  |  | **lower** | **upper** |  |  |  |
| Ranft et al., 2009 | | | Age | | | Cognitive assessment test | CERAD plus* | Cognitive impairment | Distance from traffic | NP** | Coefficients | - | - | 4 | -4.1 | 12.1 |  | NP | NS |
|  |  |  |  |  |  |  | Stroop |  |  |  |  |  |  | 7 | 0 | 13.9 | - |  |  |
|  |  |  |  |  |  |  | Sniffing |  |  |  |  |  |  | 2.8 | 0.6 | 5 |  |  |  |
| Wellenius et al., 2012 | | | Age | >77 | | Cognitive assessment test | MMSE* | Odds of having a MMSE* score of less than 26 | NP |  | Odd Ratio | - | - | 1.34 | 1.01 | 1.76 |  | 0.056 | NS |
|  |  |  |  | ≤77 | |  |  |  |  | NP |  |  |  | 0.89 | 0.65 | 1.21 |  |  |  |
| Ailshire & Crimmins, 2014 | | | Age | years | | Cognitive assessment test | Global Cognitive Score | Continuous score | PM2.5 | Per quartiles | Coefficient | - | - | Not provided | - | - | - | NP | NS |
| Gatto et al., 2014 | | | Age | <60 | | Cognitive assessment test | Verbal Learning | Sum of z score for all tests under verbal learning | PM 2.5 | per 10 μg/m^3^ | Coefficients | - | - | -0.39 | -0.87 | 0.09 |  | NP | NS |
|  |  |  |  | ≥60 | |  |  |  | PM 2.5 |  |  |  |  | -0.25 | -0.68 | 0.17 |  |  |  |
|  |  |  |  | <60 | |  | Logical Memory Factor | Sum of z score for all tests under logical memory factor | O_3_ |  |  |  |  | 0.51 | 0.11 | 0.91 | - |  |  |
|  |  |  |  | ≥60 | |  |  |  | O_3_ |  |  |  |  | Not provided | Not provided | Not provided |  |  |  |
| Tzivian, Dlugaj, Winkler, Hennig et al., 2016 | | | - | <65 | | Cognitive assessment test | Global Cognitive Score | Continuous score | PM2.5 | Per IQR increase | Coefficient | - | - | (Reference) | NP | NP | - | NP | NS |
|  |  |  |  | ≥65 | |  |  |  |  |  |  |  |  | positive |  |  |  |  |  |
| Tallon et al., 2017 | | | Age | <70 | | Change in cognitive test score | CCFM* | CCFM* Score | PM 2.5 |  | Coefficients | - | - | -0.19 | -0.41 | 0.02 | - | 0.28 | NS |
|  |  |  |  | ≥70 | |  |  |  |  |  |  |  |  | 0.47 | -0.09 | 1.02 |  |  |  |
|  |  |  |  | <70 | |  |  |  | NO_2_ | NP |  |  |  | -0.13 | -0.34 | 0.09 | - | 0.29 | NS |
|  |  |  |  | ≥70 | |  |  |  |  |  |  |  |  | -0.11 | -0.59 | 0.38 |  |  |  |
| Carey et al., 2018 | | | Age | 50-69/70-79 | | Health administrative data  (coded with ICD-10) | | Diagnosis of incident dementia | NO_2_ | Per IQR (7.5 μg/m^3^)  increase | Hazard Ratio | - | - | NP | NP | NP | - | NP | NS |
| Salinas-Rodriguez et al., 2018 | | | Age | <70 | | Cognitive assessment test | 3WMT* | remembering any of the words in the 3WMT* | PM 2.5 |  | Odd Ratio |  |  | 1.37 | 0.88 | 2.14 | - | 0.94 | NS |
|  |  |  |  | ≥70 | |  |  |  |  | Per 10 μg/m^3^ increase |  | - | - | 1.37 | 0.91 | 2.04 |  |  |  |
|  |  |  |  | <70 | |  | Semantic verbal fluency test | Continuous score |  |  | Coefficient |  |  | -0.8 | -1.18 | -0.42 |  | 0.49 | NS |
|  |  |  |  | ≥70 | |  |  |  |  |  |  |  |  | -0.8 | -1.18 | -0.42 |  |  |  |
| Cerza et al., 2019 | | | Age | - | | Hospitalization records  (coded with ICD-9) | | Diagnosis of dementia | NO_x_ | Per 10 μg/m^3^ increase | Odds ratio | - | - | NP | NP | | - | NP | NS |
| Hedge et al., 2019*** | | | Age | No | | Neuroimaging | MRI* | Left Thalamus | PM 2.5 | Per 1-unit increase | Coefficient | -14.54 | 0 | -14.54 | - | | - | P≥ 0.05 | NS |
|  |  |  |  | Yes | |  |  |  |  |  |  | -14.54 | 0.06 | -14.48 |  |  |  |  |  |
|  |  |  |  | No | |  |  | Left Thalamus | PM 2.5-10 |  |  | 36.34 | 0 | 36.34 |  |  |  |  |  |
|  |  |  |  | Yes | |  |  |  |  |  |  | 36.35 | -0.69 | 35.66 |  |  |  |  |  |
|  |  |  |  | No | |  |  | Left Thalamus | PM 10 |  |  | 20.9 | 0 | 20.9 |  |  |  |  |  |
|  |  |  |  | Yes | |  |  |  |  |  |  | 20.9 | -0.38 | 20.52 |  |  |  |  |  |
|  |  |  |  | No | |  |  | Left Thalamus | NO_2_ |  |  | -3.6 | 0 | -3.6 |  |  |  |  |  |
|  |  |  |  | Yes | |  |  |  |  |  |  | -3.6 | 0.05 | -3.55 |  |  |  |  |  |
|  |  |  |  | No | |  |  | Left Thalamus | NO |  |  | -1.85 | 0 | -1.85 |  |  |  |  |  |
|  |  |  |  | Yes | |  |  |  |  |  |  | -1.85 | 0.02 | -1.83 |  |  |  |  |  |
|  |  |  |  | No | |  |  | Right Thalamus | PM 2.5 |  |  | 10.88 | 0 | 10.88 |  |  |  |  |  |
|  |  |  |  | Yes | |  |  |  |  |  |  | 10.88 | -0.21 | 10.67 |  |  |  |  |  |
|  |  |  |  | No | |  |  | Right Thalamus | PM 2.5-10 |  |  | 34.41 | 0 | 34.41 |  |  |  |  |  |
|  |  |  |  | Yes | |  |  |  |  |  |  | 34.41 | -0.64 | 33.77 |  |  |  |  |  |
|  |  |  |  | No | |  |  | Right Thalamus | PM 10 |  |  | 18.46 | 0 | 18.46 |  |  |  |  |  |
|  |  |  |  | Yes | |  |  |  |  |  |  | 18.46 | -0.31 | 18.15 |  |  |  |  |  |
|  |  |  |  | No | |  |  | Right Thalamus | NO_2_ |  |  | -2.3 | 0 | -2.3 |  |  |  |  |  |
|  |  |  |  | Yes | |  |  |  |  |  |  | -2.3 | 0.04 | -2.26 |  |  |  |  |  |
|  |  |  |  | No | |  |  | Right Thalamus | NO |  |  | -1.31 | 0 | -1.31 |  |  |  |  |  |
|  |  |  |  | Yes | |  |  |  |  |  |  | -1.31 | 0.02 | -1.29 |  |  |  |  |  |
| Lee et al., 2019 | | | - | - | | Medicare enrollment and fee-for-service claims for hospitalization from the ResDAC | Dementia | Incident dementia | PM2.5 | per 1 μg/m3 | Hazard Ratio | - | - | - |  |  | NP | NP | NS |
| Shin et al., 2019 | | | Age | ≥75 | | Cognitive assessment test (MMSE-Korean version, Word list recall) | Korean MMSE | Cognitive score | PM2.5 | Per IQR increase | Coefficients | - | - | - |  |  | Positive | - | S |
|  |  |  |  | <75 | |  |  |  |  |  |  |  |  |  |  |  |  |  |  |
| Hedges 2020**** | | | Age | No | | Neuroimaging | MRI* | Left HV* (mm3) | PM2.5 | Per IQR increase | Coefficient | 22.1 | 0 | 22.1 |  |  | - | NP | NS |
|  |  |  |  | Yes | |  |  |  |  |  |  | 22.1 | -0.46 | 21.64 |  |  |  |  |  |
|  |  |  |  | No | |  |  | Right HV* (mm3) |  |  |  | -5.23 | 0 | -5.23 |  |  | - | NP | NS |
|  |  |  |  | Yes | |  |  |  |  |  |  | -5.23 | -0.02 | -5.25 |  |  |  |  |  |
|  |  |  |  | No | |  |  | Left HV* (mm3) | PM2.5-10 |  |  | 102.56 | 0 | 102.56 |  |  | Negative | <0.01 | S |
|  |  |  |  | Yes | |  |  |  |  |  |  | 102.56 | -1.84 | 100.72 |  |  |  |  |  |
|  |  |  |  | No | |  |  | Right HV* (mm3) |  |  |  | 80.26 | 0 | 80.26 |  |  | Negative | <0.05 | S |
|  |  |  |  | Yes | |  |  |  |  |  |  | 80.26 | -1.43 | 78.83 |  |  |  |  |  |
|  |  |  |  | No | |  |  | Left HV* (mm3) | PM10 |  |  | 52.12 | 0 | 52.12 |  |  | Negative | <0.01 | S |
|  |  |  |  | Yes | |  |  |  |  |  |  | 52.12 | -0.9 | 51.22 |  |  |  |  |  |
|  |  |  |  | No | |  |  | Right HV* (mm3) |  |  |  | 36.14 | 0 | 36.14 |  |  | Negative | <0.05 | S |
|  |  |  |  | Yes | |  |  |  |  |  |  | 36.14 | -0.62 | 35.52 |  |  |  |  |  |
|  |  |  |  | No | |  |  | Left HV* (mm3) | NO_2_ |  |  | -1.08 | 0 | -1.08 |  |  | - | NP | NS |
|  |  |  |  | Yes | |  |  |  |  |  |  | -1.08 | 0.02 | -1.06 |  |  |  |  |  |
|  |  |  |  | No | |  |  | Right HV* (mm3) |  |  |  | -4.06 | 0 | -4.06 |  |  | - | NP | NS |
|  |  |  |  | Yes | |  |  |  |  |  |  | -4.06 | 0.06 | -4 |  |  |  |  |  |
|  |  |  |  | No | |  |  | Left HV* (mm3) | NO_x_ |  |  | -0.99 | 0 | -0.99 |  |  | - | NP | NS |
|  |  |  |  | Yes | |  |  |  |  |  |  | -0.99 | 0.01 | -0.98 |  |  |  |  |  |
|  |  |  |  | No | |  |  | Right HV* (mm3) |  |  |  | -3.48 | 0 | -3.48 |  |  | - | NP | NS |
|  |  |  |  | Yes | |  |  |  |  |  |  | -3.48 | 0.05 | -3.43 |  |  |  |  |  |
| Kulick et al., 2020 | | | Age | <75 | | Cognitive assessment test | Test batteries from WHICAP | Change in the test score | NO_2_ | NP | Coefficient | - | - | -0.08 | -0.11 | -0.05 | - | 0.42 | NS |
|  |  |  |  | ≥75 | |  |  |  | NO_2_ |  |  |  |  | -0.083 | -0.11 | -0.06 |  |  |  |
|  |  |  |  | <75 | |  |  |  | PM2.5 |  |  |  |  | -0.115 | -0.15 | -0.08 | - | 0.62 | NS |
|  |  |  |  | ≥75 | |  |  |  | PM2.5 |  |  |  |  | -0.117 | -0.15 | -0.09 |  |  |  |
|  |  |  |  | <75 | |  |  |  | PM10 |  |  |  |  | -0.038 | -0.06 | -0.02 | - | 0.22 | NS |
|  |  |  |  | ≥75 | |  |  |  | PM10 |  |  |  |  | -0.043 | -0.06 | -0.02 |  |  |  |
| Gale et al., 2020*** | | | Continuous | No | | Neuroimaging | MRI | Pole (Left) | PM2.5 | NP | Coefficient | -0.59 | 0 | -0.59 | NP | |  | P > 0.05 | NS |
|  |  |  |  | Yes | |  |  |  |  |  |  | -0.59 | -0.75 | -1.34 |  |  |  |  |  |
|  |  |  |  | No | |  |  | Superior Gyrus (Left) |  |  |  | -84.84 | 0 | -84.84 |  |  | - |  |  |
|  |  |  |  | Yes | |  |  |  |  |  |  | -84.84 | 1.17 | -83.67 |  |  |  |  |  |
|  |  |  |  | No | |  |  | Medial Cortex (Left) |  |  |  | 23.79 | 0 | 23.79 |  |  |  |  |  |
|  |  |  |  | Yes | |  |  |  |  |  |  | 23.79 | -0.41 | 23.38 |  |  |  |  |  |
|  |  |  |  | No | |  |  | Orbital Cortex (Left) |  |  |  | 15.98 | 0 | 15.98 |  |  |  |  |  |
|  |  |  |  | Yes | |  |  |  |  |  |  | 15.98 | -0.42 | 15.56 |  |  |  |  |  |
|  |  |  |  | No | |  |  | Operculum Cortex (Left) |  |  |  | 5.43 | 0 | 5.43 |  |  |  |  |  |
|  |  |  |  | Yes | |  |  |  |  |  |  | 5.43 | -0.17 | 5.26 |  |  |  |  |  |
|  |  |  |  | No | |  |  | Pole (Right) |  |  |  | 35.83 | 0 | 35.83 |  |  |  |  |  |
|  |  |  |  | Yes | |  |  |  |  |  |  | 35.83 | -1.29 | 34.54 |  |  |  |  |  |
|  |  |  |  | No | |  |  | Superior Gyrus (Right) |  |  |  | 160.47 | 0 | 160.47 |  |  |  |  |  |
|  |  |  |  | Yes | |  |  |  |  |  |  | 160.47 | -2.69 | 157.78 |  |  |  |  |  |
|  |  |  |  | No | |  |  | Medial Cortex (Right) |  |  |  | 24.6 | 0 | 24.6 |  |  |  |  |  |
|  |  |  |  | Yes | |  |  |  |  |  |  | 24.6 | -0.42 | 24.18 |  |  |  |  |  |
|  |  |  |  | No | |  |  | Orbital Cortex (Right) |  |  |  | -16.55 | 0 | -16.55 |  |  |  |  |  |
|  |  |  |  | Yes | |  |  |  |  |  |  | -16.55 | 0.1 | -16.45 |  |  |  |  |  |
|  |  |  |  | No | |  |  | Operculum Cortex ((Right) |  |  |  | 7.2 | 0 | 7.2 |  |  |  |  |  |
|  |  |  |  | Yes | |  |  |  |  |  |  | 7.2 | -0.09 | 7.11 |  |  |  |  |  |
|  |  |  |  | No | |  |  | Pole (Left) | PM 2.5-10 (coarse) |  |  | -163.55 | 0 | -163.55 |  |  |  |  |  |
|  |  |  |  | Yes | |  |  |  |  |  |  | -163.55 | 2.04 | -161.51 |  |  |  |  |  |
|  |  |  |  | No | |  |  | Superior Gyrus (Left) |  |  |  | 55.19 | 0 | 55.19 |  |  |  |  |  |
|  |  |  |  | Yes | |  |  |  |  |  |  | 55.19 | -1.5 | 53.69 |  |  |  |  |  |
|  |  |  |  | No | |  |  | Medial Cortex (Left) |  |  |  | 3.62 | 0 | 3.62 |  |  |  |  |  |
|  |  |  |  | Yes | |  |  |  |  |  |  | 3.62 | -0.14 | 3.48 |  |  |  |  |  |
|  |  |  |  | No | |  |  | Orbital Cortex (Left) |  |  |  | 37.41 | 0 | 37.41 |  |  |  |  |  |
|  |  |  |  | Yes | |  |  |  |  |  |  | 37.41 | -0.74 | 36.67 |  |  |  |  |  |
|  |  |  |  | No | |  |  | Operculum Cortex (Left) |  |  |  | 8.61 | 0 | 8.61 |  |  |  |  |  |
|  |  |  |  | Yes | |  |  |  |  |  |  | 8.61 | -0.22 | 8.39 |  |  |  |  |  |
|  |  |  |  | No | |  |  | Pole (Right) |  |  |  | -121.03 | 0 | -121.03 |  |  |  |  |  |
|  |  |  |  | Yes | |  |  |  |  |  |  | -121.03 | 0.97 | -120.06 |  |  |  |  |  |
|  |  |  |  | No | |  |  | Superior Gyrus (Right) |  |  |  | 44.55 | 0 | 44.55 |  |  |  |  |  |
|  |  |  |  | Yes | |  |  |  |  |  |  | 44.55 | 0.89 | 45.44 |  |  |  |  |  |
|  |  |  |  | No | |  |  | Medial Cortex (Right) |  |  |  | -3.63 | 0 | -3.63 |  |  |  |  |  |
|  |  |  |  | Yes | |  |  |  |  |  |  | -3.63 | -0.12 | -3.75 |  |  |  |  |  |
|  |  |  |  | No | |  |  | Orbital Cortex (Right) |  |  |  | 39.27 | 0 | 39.27 |  |  |  |  |  |
|  |  |  |  | Yes | |  |  |  |  |  |  | 39.27 | -0.8 | 38.47 |  |  |  |  |  |
|  |  |  |  | No | |  |  | Operculum Cortex ((Right) |  |  |  | 7.3 | 0 | 7.3 |  |  |  |  |  |
|  |  |  |  | Yes | |  |  |  |  |  |  | 7.3 | -0.15 | 7.15 |  |  |  |  |  |
|  |  |  |  | No | |  |  | Pole (Left) | PM10 |  |  | -107.11 | 0 | -107.11 |  |  |  |  |  |
|  |  |  |  | Yes | |  |  |  |  |  |  | -107.11 | 1.37 | -105.74 |  |  |  |  |  |
|  |  |  |  | No | |  |  | Superior Gyrus (Left) |  |  |  | -16.52 | 0 | -16.52 |  |  |  |  |  |
|  |  |  |  | Yes | |  |  |  |  |  |  | -16.52 | -0.41 | -16.93 |  |  |  |  |  |
|  |  |  |  | No | |  |  | Medial Cortex (Left) |  |  |  | 15.8 | 0 | 15.8 |  |  |  |  |  |
|  |  |  |  | Yes | |  |  |  |  |  |  | 15.8 | -0.28 | 15.52 |  |  |  |  |  |
|  |  |  |  | No | |  |  | Orbital Cortex (Left) |  |  |  | 12.17 | 0 | 12.17 |  |  |  |  |  |
|  |  |  |  | Yes | |  |  |  |  |  |  | 12.17 | -0.26 | 11.91 |  |  |  |  |  |
|  |  |  |  | No | |  |  | Operculum Cortex (Left) |  |  |  | 4.24 | 0 | 4.24 |  |  |  |  |  |
|  |  |  |  | Yes | |  |  |  |  |  |  | 4.24 | -0.12 | 4.12 |  |  |  |  |  |
|  |  |  |  | No | |  |  | Pole (Right) |  |  |  | -47.28 | 0 | -47.28 |  |  |  |  |  |
|  |  |  |  | Yes | |  |  |  |  |  |  | -47.28 | 0.33 | -46.95 |  |  |  |  |  |
|  |  |  |  | No | |  |  | Superior Gyrus (Right) |  |  |  | 24.97 | 0 | 24.97 |  |  |  |  |  |
|  |  |  |  | Yes | |  |  |  |  |  |  | 24.97 | -0.42 | 24.55 |  |  |  |  |  |
|  |  |  |  | No | |  |  | Medial Cortex (Right) |  |  |  | 11.47 | 0 | 11.47 |  |  |  |  |  |
|  |  |  |  | Yes | |  |  |  |  |  |  | 11.47 | -0.21 | 11.26 |  |  |  |  |  |
|  |  |  |  | No | |  |  | Orbital Cortex (Right) |  |  |  | 11.97 | 0 | 11.97 |  |  |  |  |  |
|  |  |  |  | Yes | |  |  |  |  |  |  | 11.97 | -0.26 | 11.71 |  |  |  |  |  |
|  |  |  |  | No | |  |  | Operculum Cortex ((Right) |  |  |  | 7.85 | 0 | 7.85 |  |  |  |  |  |
|  |  |  |  | Yes | |  |  |  |  |  |  | 7.85 | -0.12 | 7.73 |  |  |  |  |  |
|  |  |  |  | No | |  |  | Pole (Left) | NO_2_ |  |  | -16.3 | 0 | -16.3 |  |  |  |  |  |
|  |  |  |  | Yes | |  |  |  |  |  |  | -16.31 | 0.18 | -16.13 |  |  |  |  |  |
|  |  |  |  | No | |  |  | Superior Gyrus (Left) |  |  |  | -5.04 | 0 | -5.04 |  |  |  |  |  |
|  |  |  |  | Yes | |  |  |  |  |  |  | -5.04 | 0.1 | -4.94 |  |  |  |  |  |
|  |  |  |  | No | |  |  | Medial Cortex (Left) |  |  |  | 1.06 | 0 | 1.06 |  |  |  |  |  |
|  |  |  |  | Yes | |  |  |  |  |  |  | 1.06 | -0.01 | 1.05 |  |  |  |  |  |
|  |  |  |  | No | |  |  | Orbital Cortex (Left) |  |  |  | -1 | 0 | -1 |  |  |  |  |  |
|  |  |  |  | Yes | |  |  |  |  |  |  | -1 | 0.01 | -0.99 |  |  |  |  |  |
|  |  |  |  | No | |  |  | Operculum Cortex (Left) |  |  |  | -0.43 | 0 | -0.43 |  |  |  |  |  |
|  |  |  |  | Yes | |  |  |  |  |  |  | -0.43 | 0 | -0.43 |  |  |  |  |  |
|  |  |  |  | No | |  |  | Pole (Right) |  |  |  | 0.18 | 0 | 0.18 |  |  |  |  |  |
|  |  |  |  | Yes | |  |  |  |  |  |  | 0.18 | -0.1 | 0.08 |  |  |  |  |  |
|  |  |  |  | No | |  |  | Superior Gyrus (Right) |  |  |  | 20.87 | 0 | 20.87 |  |  |  |  |  |
|  |  |  |  | Yes | |  |  |  |  |  |  | 20.87 | -0.32 | 20.55 |  |  |  |  |  |
|  |  |  |  | No | |  |  | Medial Cortex (Right) |  |  |  | 3.15 | 0 | 3.15 |  |  |  |  |  |
|  |  |  |  | Yes | |  |  |  |  |  |  | 3.15 | -0.02 | 3.13 |  |  |  |  |  |
|  |  |  |  | No | |  |  | Orbital Cortex (Right) |  |  |  | -7.87 | 0 | -7.87 |  |  |  |  |  |
|  |  |  |  | Yes | |  |  |  |  |  |  | -7.87 | 0.11 | -7.76 |  |  |  |  |  |
|  |  |  |  | No | |  |  | Operculum Cortex ((Right) |  |  |  | 2.03 | 0 | 2.03 |  |  |  |  |  |
|  |  |  |  | Yes | |  |  |  |  |  |  | 2.03 | -0.03 | 2 |  |  |  |  |  |
|  |  |  |  | No | |  |  | Pole (Left) | NO |  |  | -0.67 | 0 | -0.67 |  |  |  |  |  |
|  |  |  |  | Yes | |  |  |  |  |  |  | -0.67 | -0.04 | -0.71 |  |  |  |  |  |
|  |  |  |  | No | |  |  | Superior Gyrus (Left) |  |  |  | -1.88 | 0 | -1.88 |  |  |  |  |  |
|  |  |  |  | Yes | |  |  |  |  |  |  | -1.88 | 0.03 | -1.85 |  |  |  |  |  |
|  |  |  |  | No | |  |  | Medial Cortex (Left) |  |  |  | 1.09 | 0 | 1.09 |  |  |  |  |  |
|  |  |  |  | Yes | |  |  |  |  |  |  | 1.09 | -0.02 | 1.07 |  |  |  |  |  |
|  |  |  |  | No | |  |  | Orbital Cortex (Left) |  |  |  | 1.48 | 0 | 1.48 |  |  |  |  |  |
|  |  |  |  | Yes | |  |  |  |  |  |  | 1.48 | -0.04 | 1.44 |  |  |  |  |  |
|  |  |  |  | No | |  |  | Operculum Cortex (Left) |  |  |  | -0.41 | 0 | -0.41 |  |  |  |  |  |
|  |  |  |  | Yes | |  |  |  |  |  |  | -0.41 | 0 | -0.41 |  |  |  |  |  |
|  |  |  |  | No | |  |  | Pole (Right) |  |  |  | -0.37 | 0 | -0.37 |  |  |  |  |  |
|  |  |  |  | Yes | |  |  |  |  |  |  | -0.37 | -0.06 | -0.43 |  |  |  |  |  |
|  |  |  |  | No | |  |  | Superior Gyrus (Right) |  |  |  | 9.59 | 0 | 9.59 |  |  |  |  |  |
|  |  |  |  | Yes | |  |  |  |  |  |  | 9.59 | -0.15 | 9.44 |  |  |  |  |  |
|  |  |  |  | No | |  |  | Medial Cortex (Right) |  |  |  | 1.1 | 0 | 1.1 |  |  |  |  |  |
|  |  |  |  | Yes | |  |  |  |  |  |  | 1.1 | -0.02 | 1.08 |  |  |  |  |  |
|  |  |  |  | No | |  |  | Orbital Cortex (Right) |  |  |  | -2.63 | 0 | -2.63 |  |  |  |  |  |
|  |  |  |  | Yes | |  |  |  |  |  |  | -2.63 | 0.03 | -2.6 |  |  |  |  |  |
|  |  |  |  | No | |  |  | Operculum Cortex ((Right) |  |  |  | 0.53 | 0 | 0.53 |  |  |  |  |  |
|  |  |  |  | Yes | |  |  |  |  |  |  | 0.53 | -0.01 | 0.52 |  |  |  |  |  |
| Shi et al., 2020 | | | Age | Younger than 80 | | Alzheimer’s disease and related dementia | Hospital admission code (ICD-9: 331·0, 290; ICD-10: G30·9,  and F05) | Diagnosis of Alzheimer’s disease and related dementia | PM2.5 | Per 5 μg/m3 increase | Hazard Ratio |  |  | NP | NP | NP | - | NP | NS |
|  |  |  |  | Older than 80 | |  |  |  |  |  |  |  |  |  |  |  |  |  |  |
| Wang et al., 2020 | | | Age | 65-79 | | Cognitive assessment test | MMSE* | a score of less than 18 on the  Chinese version of the MMSE* | PM 2.5 | NP | Hazard Ratio | - | - | 1.038 | 0.99 | 1.087 | - | 0.68 | NS |
|  |  |  |  | 80-89 | |  |  |  |  |  |  |  |  | 1.077 | 1.022 | 1.136 |  |  |  |
|  |  |  |  | 90-99 | |  |  |  |  |  |  |  |  | 1.058 | 1.001 | 1.118 |  |  |  |
|  |  |  |  | ≥100 | |  |  |  |  |  |  |  |  | 1.021 | 0.952 | 1.095 |  |  |  |
| Yuchi et al., 2020 | | |  | <65 | | Medical Record (ICD 9^th^) | Dementia | Diagnosis of Non-Alzheimer Dementia | Highway <50m |  | Hazard Ratio | - | - | 1.02 | 0.89 | 1.16 | - | NP | NS |
|  |  |  |  | ≥65 | |  |  |  | Highway <50m | NP |  |  |  | 0.98 | 0.68 | 1.41 |  |  | NS |
|  |  |  |  | <65 | |  |  |  | Highway <50m + Greenness |  |  |  |  | 1.00 | 0.84 | 1.19 |  |  | NS |
|  |  |  |  | ≥65 | |  |  |  | Highway <50m + Greenness |  |  |  |  | 0.96 | 0.7 | 1.3 |  |  | NS |
|  |  |  |  | <65 | |  |  |  | Highway <150m |  |  |  |  | 1.09 | 1.01 | 1.19 |  |  | NS |
|  |  |  |  | ≥65 | |  |  |  | Highway <150m |  |  |  |  | 0.97 | 0.8 | 1.18 |  |  | NS |
|  |  |  |  | <65 | |  |  |  | Highway <150m + Greenness |  |  |  |  | 1.03 | 0.92 | 1.15 |  |  | NS |
|  |  |  |  | ≥65 | |  |  |  | Highway <150m + Greenness |  |  |  |  | 0.96 | 0.7 | 1.3 |  |  | NS |
|  |  |  |  | <65 | |  |  |  | Major road <50m |  |  |  |  | 1.13 | 1.05 | 1.21 |  |  | NS |
|  |  |  |  | ≥65 | |  |  |  | Major road <50m |  |  |  |  | 1.36 | 1.16 | 1.62 |  |  | NS |
|  |  |  |  | <65 | |  |  |  | Major road <50m + Greenness |  |  |  |  | 1.11 | 1.02 | 1.2 |  |  | NS |
|  |  |  |  | ≥65 | |  |  |  | Major road <50m + Greenness |  |  |  |  | 1.33 | 1.14 | 1.57 |  |  | NS |
|  |  |  |  | <65 | |  |  |  | Major road <50m or Highway <150m |  |  |  |  | 1.12 | 1.05 | 1.19 |  |  | NS |
|  |  |  |  | ≥65 | |  |  |  | Major road <50m or Highway <150m |  |  |  |  | 1.19 | 1.01 | 1.41 |  |  | NS |
|  |  |  |  | <65 | |  |  |  | Major road <50m or Highway <150m + Greenness |  |  |  |  | 1.09 | 1.01 | 1.18 |  |  | NS |
|  |  |  |  | ≥65 | |  |  |  | Major road <50m or Highway <150m + Greenness |  |  |  |  | 1.18 | 1.02 | 1.36 |  |  | NS |
|  |  |  |  | <65 | |  |  |  | PM2.5 (μg/m³) |  |  |  |  | 1.03 | 0.99 | 1.06 |  |  | NS |
|  |  |  |  | ≥65 | |  |  |  | PM2.5 (μg/m³) |  |  |  |  | 1.05 | 0.94 | 1.17 |  |  | NS |
|  |  |  |  | <65 | |  |  |  | PM2.5 (μg/m³) + Noise |  |  |  |  | 1.03 | 0.99 | 1.06 |  |  | NS |
|  |  |  |  | ≥65 | |  |  |  | PM2.5 (μg/m³) + Noise |  |  |  |  | 1.05 | 0.93 | 1.18 |  |  | NS |
|  |  |  |  | <65 | |  |  |  | PM2.5 (μg/m³) + Greenness |  |  |  |  | 1.03 | 0.99 | 1.06 |  |  | NS |
|  |  |  |  | ≥65 | |  |  |  | PM2.5 (μg/m³) + Greenness |  |  |  |  | 1.03 | 0.92 | 1.15 |  |  | NS |
|  |  |  |  | <65 | |  |  |  | Black carbon (μg/m³) |  |  |  |  | 1 | 0.96 | 1.04 |  |  | NS |
|  |  |  |  | ≥65 | |  |  |  | Black carbon (μg/m³) |  |  |  |  | 1.11 | 1.03 | 1.2 |  |  | NS |
|  |  |  |  | <65 | |  |  |  | Black carbon (μg/m³) + Noise |  |  |  |  | 1.01 | 0.96 | 1.05 |  |  | NS |
|  |  |  |  | ≥65 | |  |  |  | Black carbon (μg/m³) + Noise |  |  |  |  | 1.11 | 1.03 | 1.2 |  |  | NS |
|  |  |  |  | <65 | |  |  |  | Black carbon (μg/m³) + Greenness |  |  |  |  | 1 | 0.96 | 1.05 |  |  | NS |
|  |  |  |  | ≥65 | |  |  |  | Black carbon (μg/m³) + Greenness |  |  |  |  | 1.11 | 1.02 | 1.23 |  |  | NS |
|  |  |  |  | <65 | |  |  |  | NO2 (ppb) |  |  |  |  | 1.06 | 1.02 | 1.1 |  |  | NS |
|  |  |  |  | ≥65 | |  |  |  | NO2 (ppb) |  |  |  |  | 1.06 | 0.95 | 1.19 |  |  | NS |
|  |  |  |  | <65 | |  |  |  | NO2 (ppb) + Noise |  |  |  |  | 1.04 | 0.99 | 1.09 |  |  | NS |
|  |  |  |  | ≥65 | |  |  |  | NO2 (ppb) + Noise |  |  |  |  | 1.05 | 0.97 | 1.15 |  |  | NS |
|  |  |  |  | <65 | |  |  |  | NO2 (ppb) + Greenness |  |  |  |  | 1.06 | 1.01 | 1.1 |  |  | NS |
|  |  |  |  | ≥65 | |  |  |  | NO2 (ppb) + Greenness |  |  |  |  | 1.05 | 0.93 | 1.19 |  |  | NS |
|  |  |  |  | <65 | |  |  |  | NO (ppb) |  |  |  |  | 1 | 0.95 | 1.04 |  |  | NS |
|  |  |  |  | ≥65 | |  |  |  | NO (ppb) |  |  |  |  | 1.08 | 0.97 | 1.21 |  |  | NS |
|  |  |  |  | <65 | |  |  |  | NO (ppb) + Noise |  |  |  |  | 0.99 | 0.94 | 1.04 |  |  | NS |
|  |  |  |  | ≥65 | |  |  |  | NO (ppb) + Noise |  |  |  |  | 1.01 | 0.98 | 1.06 |  |  | NS |
|  |  |  |  | <65 | |  |  |  | NO (ppb) + Greenness |  |  |  |  | 1.01 | 0.98 | 1.03 |  |  | NS |
|  |  |  |  | ≥65 | |  |  |  | NO (ppb) + Greenness |  |  |  |  | 1.03 | 0.97 | 1.21 |  |  | NS |
| Younan et al., 2020 | | | Age | <76.84 | | Cognitive assessment test | AD-PS Score* | 5-year stan-  dardized change in AD-PS score | PM 2.5 | Per IQR change | Coefficient | - | - | 0.035 | 0.012 | 0.058 | - | 0.26 | NS |
|  |  |  |  | ≥76.84 | |  |  |  |  |  |  |  |  | 0.017 | -0.006 | 0.041 |  |  | NS |
| Yao et al., 2021 | | | Age | <80 | | Cognitive assessment test | Chinsese MMSE* | MMSE* score lower than 25 | distance to the  main roadway (<=100m vs. >100m) | NP | Odd Ratios | - | - | 1.5 | 1.22 | 1.84 | Negative | 0.008 | S |
|  |  |  |  | ≥80 | |  |  |  |  |  |  |  |  | 1.13 | 1.01 | 1.28 |  |  |  |
| Crous-bou et al., 2020 | | | Age | <=60 | | Neuroimaging | MRI | AD* signature for cortical thickness | NO2 | NP | Coefficient | - | - | -16.4 | NP | | - | 0.10 | NS |
|  |  |  |  | >60 | |  |  |  |  |  |  |  |  | -19.1 |  |  |  |  |  |
|  |  |  |  | <=60 | |  |  |  | NOx |  |  |  |  | -36.9 |  |  |  | 0.07 | NS |
|  |  |  |  | >60 | |  |  |  |  |  |  |  |  | -51.2 |  |  |  |  |  |
|  |  |  |  | <=60 | |  |  |  | PM2.5 |  |  |  |  | -3.57 |  |  |  | 0.40 | NS |
|  |  |  |  | >60 | |  |  |  |  |  |  |  |  | -2.71 |  |  |  |  |  |
|  |  |  |  | <=60 | |  |  |  | PM10 |  |  |  |  | -6.97 |  |  |  | 0.50 | NS |
|  |  |  |  | >60 | |  |  |  |  |  |  |  |  | -5.75 |  |  |  |  |  |
|  |  |  |  | <=60 | |  |  |  | PM2.5-abs |  |  |  |  | -0.62 |  |  |  | 0.13 | NS |
|  |  |  |  | >60 | |  |  |  |  |  |  |  |  | -0.92 |  |  |  |  |  |
|  |  |  |  | <=60 | |  |  |  | PMcoarse |  |  |  |  | -4.09 |  |  |  | 0.08 | NS |
|  |  |  |  | >60 | |  |  |  |  |  |  |  |  | -2.98 |  |  |  |  |  |
| Nunez et al., 2021 | | | Age | <70 | | Health administrative data  (coded with ICD-9) | | Incident AD* | PM 2.5 | 5.8 to 8.1 ug/m^3^ | Rate Ratio | - | - | 1.16 | 1.02 | 1.28 | - | Not provided | NS |
|  |  |  |  | ≥70 | |  |  |  |  |  |  |  |  | 1.11 | 1.03 | 1.18 |  |  |  |
|  |  |  |  | <70 | |  |  |  |  | 8.1 to 10.4 ug/m^3^ |  |  |  | 1.16 | 1.06 | 1.27 |  |  |  |
|  |  |  |  | ≥70 | |  |  |  |  |  |  |  |  | 1.04 | 0.99 | 1.09 |  |  |  |
| Ran et al., 2021 | | | Age | 65-75 | | Health administrative data  (coded with ICD-9) | | Incident all-cause, vascular dementia and AD* | PM 2.5 | Per IQR | Hazard Ratio | - | - | 1.08 | 0.98 | 1.18 | - | 0.597 | NS |
|  |  |  |  | >75 | |  |  |  |  |  |  |  |  | 1.06 | 0.98 | 1.14 |  |  | NS |
| Shaffer et al., 2021 | | | Age | <=73 | | Dementia assessment test | CASI* | Score below 86 | PM 2.5 | Per 1 ug/m^3^ increase | Hazard Ratio | - | - | 1.15 | 1.02 | 1.3 | - | 0.78 | NS |
|  |  |  |  | >73 | |  |  |  |  |  |  |  |  | 1.16 | 1.03 | 1.31 |  |  |  |
| Shi et al., 2021 | | | Age | <75 | | Medicare record data | Dementia | Diagnosis of dementia | PM2.5 | Per IQR increase | Hazard Ratio | - | - | NP | NP | NP | Negative | Reference | S |
|  |  |  |  | ≥75 | |  |  |  |  |  |  |  |  |  |  |  |  | <0.001 |  |
|  |  |  |  | <75 | |  |  |  | NO2 |  |  |  |  |  |  |  | Negative | Reference | S |
|  |  |  |  | ≥75 | |  |  |  |  |  |  |  |  |  |  |  |  | <0.001 |  |
|  |  |  |  | <75 | |  |  |  | O3 |  |  |  |  |  |  |  |  | Reference | NS |
|  |  |  |  | ≥75 | |  |  |  |  |  |  |  |  |  |  |  |  | 0.10 |  |
|  |  |  |  | <75 | |  | AD | Diagnosis of AD | PM2.5 |  |  |  |  |  |  |  | Positive | Reference | S |
|  |  |  |  | ≥75 | |  |  |  |  |  |  |  |  |  |  |  |  | <0.001 |  |
|  |  |  |  | <75 | |  |  |  | NO2 |  |  |  |  |  |  |  | Positive | Reference | S |
|  |  |  |  | ≥75 | |  |  |  |  |  |  |  |  |  |  |  |  | <0.001 |  |
|  |  |  |  | <75 | |  |  |  | O3 |  |  |  |  |  |  |  | Positive | Reference | S |
|  |  |  |  | ≥75 | |  |  |  |  |  |  |  |  |  |  |  |  | 0.03 |  |
| G.-C. Chen et al., 2022 | | | Age | <60 | | Hospitalization records  (coded with ICD-9 and ICD-10) | Dementia | AD | Air pollution score  (PM2.5, PM2.5-10, PM10, NO2, NOx) | Per IQR increase | Hazard Ratio | - | - | 1.13 | 0.99 | 1.30 | - | 0.56 | NS |
|  |  |  |  | ≥60 | |  |  |  |  |  |  |  |  | 1.13 | 1.07 | 1.19 |  |  |  |
|  |  |  |  | <60 | |  |  | Vascular dementia |  |  |  |  |  | 1.05 | 0.86 | 1.29 | - | 0.14 | NS |
|  |  |  |  | ≥60 | |  |  |  |  |  |  |  |  | 1.16 | 1.11 | 1.22 |  |  |  |
|  |  |  |  | <60 | |  |  | All-cause dementia |  |  |  |  |  | 1.13 | 1.04 | 1.23 | - | 0.71 | NS |
|  |  |  |  | ≥60 | |  |  |  |  |  |  |  |  | 1.11 | 1.07 | 1.15 |  |  |  |
| Lucht et al., 2022 | | | Age | <65/≥65 | | Neuroimaging | MRI | local gyrification index (lGI) | - | - | - | - | - | NP | NP | NP | - | - | NS |
| M. Li et al., 2022 | | | Age | <65 | | CSF sTREM2 score | Neuroinflammatory (a biomarker of AD, represented by CSF sTREM2 score) | Continuous score | PM2.5 | NP | Z score | - | - | -0.088 | NP | NP | - | 0.0343 | S |
|  |  |  |  | ≥65 | |  |  |  |  |  |  |  |  | -0.126 | NP | NP | - | 0.0068 |  |
|  |  |  |  | <65 | |  |  |  | O3 |  |  |  |  | -0.013 | NP | NP | - | 0.7643 |  |
|  |  |  |  | ≥65 | |  |  |  |  |  |  |  |  | -0.111 | NP | NP | - | 0.0280 |  |
| Yang et al., 2022 † | | | Age | 60-64 | | Alzheimer’s disease assessment test | MMSE*, MoCA*, and HAD* | Incident AD* based on the test score | PM 2.5 | Per 10 ug/m^3^ increase | Hazard Ratio | - | - | 0.96 | 0.72 | 1.24 |  | NP | NS |
|  |  |  |  | 65-69 | |  |  |  |  |  |  |  |  | 1.04 | 0.98 | 1.12 | - |  |  |
|  |  |  |  | 70-74 | |  |  |  |  |  |  |  |  | 1.11 | 1.1 | 1.52 |  |  |  |
|  |  |  |  | 75-85 | |  |  |  |  |  |  |  |  | 1.12 | 1.11 | 1.43 |  |  |  |
| Wang et al., 2022 | | | Age | <80 | | Dementia assessment test | Telephone Inter-  view for Cognitive Status | Diagnosis of incident dementia | PM 2.5 | Per IQR increase | Hazard Ratio | - | - | 0.84 | 0.67 | 1.07 | - | 0.63 | NS |
|  |  |  |  | ≥80 | |  |  |  | PM 2.5 |  |  |  |  | 0.79 | 0.69 | 0.91 |  |  |  |
|  |  |  |  | <80 | |  |  |  | NO_2_ |  |  |  |  | 0.88 | 0.7 | 1.11 | - | 0.37 | NS |
|  |  |  |  | ≥80 | |  |  |  | NO_2_ |  |  |  |  | 0.78 | 0.68 | 0.89 |  |  |  |
| Decrom et al., 2022 | | | Age | <70 | | Dementia assessment test | DSM-III-R*, NINCDS-ADRDA* | Diagnosis of incident dementia | Marker covering all pollutants (PM10, PM2.5, PM2.5 absorbance, NOx, NO2) | NP | Hazard Ratio | - | - | 1.22 | 0.93 | 1.61 | - | NP | NS |
|  |  |  |  | ≥70 | |  |  |  |  |  |  |  |  | 1.00 | 0.90 | 1.11 |  |  |  |
| Hu et al., 2022 | | | Age | <60 | | Cognitive assessment test | episodic memory, orientation and attention, and visuospatial ability. | A positive difference (Δ cognitive function >0) between the  2011 and 2015 cognitive function assessments; | PM1 | Per IQR increase | Odds Ratio | - | - | 0.78 | 0.74 | 0.83 |  | 0.985 | NS |
|  |  |  |  | ≥60 | |  |  |  | PM1 |  |  |  |  | 0.79 | 0.74 | 0.84 | - |  |  |
|  |  |  |  | <60 | |  |  |  | PM2.5 |  |  |  |  | 0.73 | 0.69 | 0.77 |  | 0.527 |  |
|  |  |  |  | ≥60 | |  |  |  | PM2.5 |  |  |  |  | 0.75 | 0.7 | 0.8 | - |  |  |
|  |  |  |  | <60 | |  |  |  | PM10 |  |  |  |  | 0.83 | 0.79 | 0.88 | - | 0.46 |  |
|  |  |  |  | ≥60 | |  |  |  | PM10 |  |  |  |  | 0.81 | 0.76 | 0.87 |  |  |  |
|  |  |  |  | <60 | |  |  |  | NO2 |  |  |  |  | 0.85 | 0.81 | 0.9 | - | 0.49 |  |
|  |  |  |  | ≥60 | |  |  |  | NO2 |  |  |  |  | 0.83 | 0.78 | 0.89 |  |  |  |
|  |  |  |  | <60 | |  |  |  | O3 |  |  |  |  | 1.04 | 0.99 | 1.1 | - | 0.189 |  |
|  |  |  |  | ≥60 | |  |  |  | O3 |  |  |  |  | 0.99 | 0.93 | 1.06 |  |  |  |
| Gao et al., 2022 | | | Age | 65-79 | | Cognitive assessment test | Chinses MMSE* | MMSE*  score below 18 points in combination with an MMSE* score decline from baseline ≥4  points | PM2.5 | Per 10 ug/m^3^ increase | Hazard Ratio | - | - | 1.16 | 1.031 | 1.305 | - | 0.3 | NS |
|  |  |  |  | ≥80 | |  |  |  |  |  |  |  |  | 1.086 | 1.02 | 1.157 |  |  |  |
| Hu et al., 2023 † | | | Age | Cognitive assessment test | | | Immediate and delayed word recall and serial 7s* | CHARLS* score | PM2.5 | Duration of exposure (5 yr, 10 yr, 10 yr+) | Coefficient | - | - | 0.234 | 0.171 | 0.297 | - | NP | NS |
| Lee et al., 2022 | | | Age | 50-65 | | Cognitive assessment test | MMSE* | MMSE* score | PM2.5 |  | Coefficient | - | - | -0.09 | -0.13 | -0.05 | Positive | P<0.00001 | S |
|  |  |  |  | 65-75 | |  |  |  |  |  |  |  |  | -0.12 | -0.19 | -0.05 |  |  |  |
|  |  |  |  | >75 | |  |  |  |  | Per IQR change |  |  |  | -0.27 | -0.44 | -0.11 |  |  |  |
|  |  |  |  | 50-65 | |  |  |  | PM10 |  |  |  |  | -0.12 | -0.18 | -0.06 | Negative | P<0.00001 | S |
|  |  |  |  | 65-75 | |  |  |  |  |  |  |  |  | -0.11 | -0.2 | -0.03 |  |  |  |
|  |  |  |  | >75 | |  |  |  |  |  |  |  |  | -0.05 | -0.23 | 0.13 |  |  |  |
| Shi et al., 2023 | | | Age | <75 | | Medicare record data | Dementia | Diagnosis of dementia | Nitrate | Per IQR increase | Hazard Ratio | - | - | - | NP | NP | Positive | NP | S |
|  |  |  |  | ≥75 | |  |  |  |  |  |  |  |  |  |  |  |  |  |  |
|  |  |  |  | <75 | |  |  |  | Sulfate |  |  |  |  |  |  |  |  |  |  |
|  |  |  |  | ≥75 | |  |  |  |  |  |  |  |  |  |  |  |  |  |  |
|  |  |  |  | <75 | |  |  |  | Ammonium |  |  |  |  |  |  |  |  |  |  |
|  |  |  |  | ≥75 | |  |  |  |  |  |  |  |  |  |  |  |  |  |  |
| Wyatt et al., 2023 | | | Age | 18-29 | | Cognitive assessment game | *Lost in Migration* game | Score representing cognitive performance index | PM2.5 |  | Score percentile change | - | - | -0.25 | -0.45 | -0.05 |  | NP | NS |
|  |  |  |  | 30s | |  |  |  |  |  |  |  |  | 0.19 | -0.0002 | 0.38 |  |  |  |
|  |  |  |  | 40s | |  |  |  |  | Per 1 ug/m^3^ increase |  |  |  | 0.01 | -0.17 | 0.19 |  |  |  |
|  |  |  |  | 50s | |  |  |  |  |  |  |  |  | -0.11 | -0.24 | 0.02 |  |  |  |
|  |  |  |  | 60-90 | |  |  |  |  |  |  |  |  | -0.05 | -0.13 | 0.03 |  |  |  |
|  |  |  |  | 18-29 | |  |  |  | O3 |  |  |  |  | -2.92 | -4.63 | -1.19 |  |  | NS |
|  |  |  |  | 30s | |  |  |  |  |  |  |  |  | -2.81 | -4.29 | -1.25 |  |  |  |
|  |  |  |  | 40s | |  |  |  |  |  |  |  |  | 0.57 | -0.81 | 1.96 |  |  |  |
|  |  |  |  | 50s | |  |  |  |  |  |  |  |  | 0.51 | -0.5 | 1.48 |  |  |  |
|  |  |  |  | 60-90 | |  |  |  |  |  |  |  |  | 1.22 | 0.54 | 1.87 |  |  |  |
|  | \| * AD: Alzheimer's Disease; CCFM: Chicago Cognitive Function Measure; MRI: Magnetic Resonance Imaging; MMSE: Mini-Mental State Examination; WM: White Matter; IADL: Impaired Activities of Daily Living; HbA1c: Glycosylated hemoglobin; CRP: C-reactive Protein; CVD: Cardiovascular Disease; SEVLT: Spanish English Verbal Learning Test; DSMMD: Diagnostic and Statistical Manual of Mental Disorders; 3WMT: Three-word memory test; SVF: Semantic Verbal fluency; MCI: Mild Cognitive Function; HV: Hippocampal Volumes; DADL: Disability in activities of daily living; AD-RD: Alzheimer’s disease and related dementias; MoCA: Montreal Cognitive Assessment; DSM-III: Diagnostic and Statistical Manual of Mental Disorders; NINCDS-ADRDA National Institute of Neurological and Communicative Diseases and Stroke/Alzheimer’s Disease and Related Disorders Association; CASI: Cognitive Abilities Screening Instrument; Hospital Anxiety and Depression Scale; AD-PS: Alzheimer’s disease pattern similarity \| \| --- \| \| ** CI: Confidence Interval; S/NS: Significance/Non-Significance; IQR: Inter-Quartile Range; RD: Residential Distance; NP: Not provided \| \| *** The total effect size (A+C) was manually calculated unless the effect of air pollution exposure on each category of the effect modifier was provided in the studies \| \| **** 95% CI is based on coefficient of effect modifiers  † Statistical significance was judged based on in-text statement (Hu et al., 2023) or p-value of subgroup (Yang et al., 2022)  ※ 11 studies tested age as an effect modifier but either 1) did not provide effect size measures in the tables, but tested and mentioned non significance (Carey et al., 2018; Lucht 2022, Tzivian, Dlugaj, Winkler, Hennig, et al., 20  16 , Alishire & Crimmins, 2014; Cerza et al., 2019; Shi et al., 2020) or 2) significance (Shi et al., 2021) or 3) only provided graphics without the measures (Shin et al., 2019). One study mentioned they tested modifying effect of age but did not mention about the results (Lee et al., 2019).  Significant studies: Shi et al., 2021; Shin et al., 2019, Shi et al., 2023 (significance of the modifiers in the table above is based on in-text reporting of the original study) \| | | | | | | | | | | | | | | | | | | |
|  | |  | | |  |  |  |  |  |  |  |  |  |  |  |  |  |  |  |

- Table E2: Effect size by potential effect modifier – Gender (9/36)

| **Study** | | **Effect Modifier** | **Outcome** | | | **Air Pollutants** | **Exposure**  **Level** | **Types of Effect** | **Effect**  **Size**  **(A: Main Effect by exposure)** | **Effect Size**  **(C: Effect by interaction term)** | **Effect Size  Estimate*****  **(A+C)** | **95% CI*s** | | **Direction of**  **Effect Modifier Measure** | **p-value  for  interaction terms** | **Statistical Significance (S/NS**)** |
| --- | --- | --- | --- | --- | --- | --- | --- | --- | --- | --- | --- | --- | --- | --- | --- | --- |
|  |  | **Gender** | **Outcome Measure Method** | | **Outcome Variable** |  |  |  |  |  |  | **lower** | **upper** |  |  |  |
| Ailshire & Crimmins, 2014 | | Male | Cognitive assessment test | Global Cognitive Score | Continuous score | PM2.5 | Per quartiles | Coefficient | - | - | NP** | - | NP |  | - | NS |
|  |  | Female |  |  |  |  |  |  |  |  |  |  |  | - |  |  |
| Gatto et al., 2014‡ | | Male | Cognitive assessment test | Verbal learning | Cognitive score | PM2.5 | NP | Coefficient | - | - | -0.41 | -0.99 | 0.17 | - | NP | NS |
|  |  | Female |  |  |  |  |  |  |  |  | -0.29 | -0.67 | 0.09 |  |  |  |
|  |  | Male |  | Logical Memory Factor |  | O_3_ |  |  |  |  | NP | NP | NP | - | NP | NP |
|  |  | Female |  |  |  |  |  |  |  |  | 0.46 | 0.09 | 0.83 |  |  |  |
| Wu et al., 2015 | | Men | AD | Diagnosis of AD | AD | PM10 | 2^nd^ tertile | Odds Ratio | - | - | 1.93 | 0.81 | 4.58 | - | 0.95 | NS |
|  |  | Women |  |  |  |  |  |  |  |  | 1.48 | 0.64 | 3.38 |  |  |  |
|  |  | Men |  |  |  |  | 3^rd^ tertile |  |  |  | 4.50 | 1.85 | 10.95 |  |  |  |
|  |  | Women |  |  |  |  |  |  |  |  | 3.75 | 1.60 | 8.81 |  |  |  |
|  |  | Men |  |  |  | Ozone | 2^nd^ tertile |  |  |  | 0.50 | 0.20 | 1.27 | - | 0.48 | NS |
|  |  | Women |  |  |  |  |  |  |  |  | 0.70 | 0.30 | 1.64 |  |  |  |
|  |  | Men |  |  |  |  | 3^rd^ tertile |  |  |  | 2.24 | 0.97 | 5.18 |  |  |  |
|  |  | Women |  |  |  |  |  |  |  |  | 1.93 | 0.86 | 4.34 |  |  |  |
|  |  | Men | Vascular dementia | Diagnosis of vascular dementia | Vascular dementia | PM10 | 2^nd^ tertile |  |  |  | 1.62 | 0.54 | 4.84 | - | 0.78 | NS |
|  |  | Women |  |  |  |  |  |  |  |  | 2.11 | 0.71 | 6.32 |  |  |  |
|  |  | Men |  |  |  |  | 3^rd^ tertile |  |  |  | 3.46 | 1.11 | 10.78 |  |  |  |
|  |  | Women |  |  |  |  |  |  |  |  | 2.90 | 0.94 | 8.98 |  |  |  |
|  |  | Men |  |  |  | Ozone | 2^nd^ tertile |  |  |  | 0.54 | 0.17 | 1.68 |  | 0.70 | NS |
|  |  | Women |  |  |  |  |  |  |  |  | 0.85 | 0.27 | 2.70 |  |  |  |
|  |  | Men |  |  |  |  | 3^rd^ tertile |  |  |  | 1.58 | 0.57 | 4.37 | - |  |  |
|  |  | Women |  |  |  |  |  |  |  |  | 2.28 | 0.75 | 6.94 |  |  |  |
| Tzivian, Dlugaj, Winkler, Hennig, et al., 2016† | | Male | Cognitive assessment test | Global Cognitive Score | Continuous score | PM2.5 | Per IQR increase | Coefficient | - | - | -0.14 | -0.33 | -0.04 |  | 0.04 | S |
|  |  | Female |  |  |  |  |  |  |  |  | --0.28 | -0.41 | -0.15 | Positive |  |  |
| Tzivian, Dlugaj, Winkler, Weinmayr, et al., 2016 | | Male | Cognitive assessment test | Diagnosis of cognitive impairment | Cognitive impairment | PM2.5 | Per IQR increase | Odd Ratio | - | - | (Reference) | NP | NP |  | NP | NS |
|  |  | Female |  |  |  |  |  |  |  |  | Qualitative |  |  | - |  |  |
| Chen et al., 2017 | | Male | Health administrative data  (coded with ICD-9 or ICD-10) | | Incident dementia | PM2.5, NO2 | per IQR (=14.2 ppb, 4.8 μg/m3 for NO2 and PM2.5, respectively ) | Hazard Ratio | - | - | Not provided | NP | NP |  | >0.05 | NS |
|  |  | Female |  |  |  |  |  |  |  |  |  |  |  | -- |  |  |
| Salinas-Rodriguez et al., 2018 | | Male | Cognitive assessment test | 3WMT* | remembering any of the words in the 3WMT* | PM2.5 | per 10 μg/m^3^ | Odd Ratio | - | - | 1.30 | 0.91 | 1.87 | - | 0.77 | NS |
|  |  | Female |  |  |  |  |  |  |  |  | 1.34 | 0.94 | 1.82 |  |  |  |
|  |  | Male |  | Semantic verbal fluency test | Continuous score |  |  | Coefficient |  |  | -0.85 | -1.25 | -0.44 | - | 0.28 | NS |
|  |  | Female |  |  |  |  |  |  |  |  | -0.83 | -1.21 | -0.45 |  |  |  |
| Carey et al., 2018 | | Male | Health administrative data  (coded with ICD-10) | | Diagnosis of incident dementia | NO_2_ | Per IQR (7.5 μg/m^3^)  increase | Hazard Ratio | - | - | NP | NP | NP | - | 0.27 | NS |
|  |  | Female |  |  |  |  |  |  |  |  |  |  |  |  |  |  |
| Hedges et al., 2019*** | | Male | Neuroimaging | MRI | Left HV | PM2.5 | Per 1-unit increase | Coefficients | -4.91 | 0 | -4.91 | NP | | - | P > 0.05 | NS |
|  |  | Female |  |  |  |  |  |  | -4.91 | -11.42 | -16.33 |  |  |  |  |  |
|  |  | Male |  |  | Right HV |  |  |  | 2.00 | 0 | 2 |  |  |  |  |  |
|  |  | Female |  |  |  |  |  |  | 2.00 | -8.32 | -6.32 |  |  |  |  |  |
|  |  | Male |  |  | Left HV | PM2.5-10 |  |  | -7.43 | 0 | -7.43 |  |  |  |  |  |
|  |  | Female |  |  |  |  |  |  | -7.43 | 1.34 | -6.09 |  |  |  |  |  |
|  |  | Male |  |  | Right HV |  |  |  | -4.87 | 0 | -4.87 |  |  |  |  |  |
|  |  | Female |  |  |  |  |  |  | -4.87 | -1.48 | -6.35 |  |  |  |  |  |
|  |  | Male |  |  | Left HV | PM10 |  |  | -2.33 | 0 | -2.33 |  |  |  |  |  |
|  |  | Female |  |  |  |  |  |  | -2.33 | -0.77 | -3.1 |  |  |  |  |  |
|  |  | Male |  |  | Right HV |  |  |  | 1.48 | 0 | 1.48 |  |  |  |  |  |
|  |  | Female |  |  |  |  |  |  | 1.48 | -4.58 | -3.1 |  |  |  |  |  |
|  |  | Male |  |  | Left HV | NO_2_ |  |  | 0.60 | 0 | 0.6 |  |  | Qualitative | P<0.01 | S |
|  |  | Female |  |  |  |  |  |  | 0.6 | -2.58 | -1.98 |  |  |  |  |  |
|  |  | Male |  |  | Right HV |  |  |  | 1.18 | 0 | 1.18 |  |  |  | P > 0.05 | NS |
|  |  | Female |  |  |  |  |  |  | 1.18 | -1.72 | -0.54 |  |  |  |  |  |
|  |  | Male |  |  | Left HV | NO |  |  | 0.24 | 0 | 0.24 |  |  | Qualitative | P < 0.01 | S |
|  |  | Female |  |  |  |  |  |  | 0.24 | -1.33 | -1.09 |  |  |  |  |  |
|  |  | Male |  |  | Right HV |  |  |  | 0.61 | 0 | 0.61 |  |  | Qualitative | P < 0.01 | S |
|  |  | Female |  |  |  |  |  |  | 0.61 | -0.98 | -0.37 |  |  |  |  |  |
| Oudin et al., 2019 | | Male | Diagnosis of VaD* using MMSE (Diagnostic and Statistical Manual of Mental Disorders, fourth edition criteria) | | Diagnosis of VaD* / Diagnosis of AD | NO_x_ | NP | Hazard Ratio | Not provided | - | - | - | - | - | - | NS |
|  |  | Female |  |  |  |  |  |  |  |  |  |  |  |  |  |  |
| Shin et al., 2019 | | Male | Cognitive assessment test (Recall Storage(%)) | Korean MMSE | Cognitive score | PM2.5 | Per IQR increase | Coefficients | - | - | - | - | - | Positive | - | S |
|  |  | Female |  |  |  |  |  |  |  |  |  |  |  |  |  |  |
| Kim et al., 2019 | | Male | Cognitive assessment test | MMSE* | 23 ≥ MMSE score | PM2.5 | Per 10-unit increase | Odds ratio | - | - | 1.01 | 0.93 | 1.11 |  | - | NP |
|  |  | Female |  |  |  |  |  |  |  |  | 1.04 | 0.90 | 1.19 |  |  |  |
|  |  | Male |  |  |  | PM2.5-10 |  |  |  |  | 1.04 | 0.95 | 1.15 |  |  |  |
|  |  | Female |  |  |  |  |  |  |  |  | 1.12 | 0.96 | 1.30 | - |  |  |
|  |  | Male |  |  |  | NO_2_ |  |  |  |  | 1.01 | 0.98 | 1.04 |  |  |  |
|  |  | Female |  |  |  |  |  |  |  |  | 1.00 | 0.94 | 1.06 |  |  |  |
| Cerza et al., 2019 | | Male | Hospitalization records  (coded with ICD-9) | | Diagnosis of dementia | NO_x_ | Per 10 μg/m^3^ increase | Odds ratio | - | - | 1.06 | 1.03 | 1.09 |  | 0.053 | NS |
|  |  | Female |  |  |  |  |  |  |  |  | 1.1 | 1.07 | 1.12 | - |  |  |
| Crous-Bou et al., 2020 | | Male | Neuroimaging | MRI | AD* signature for cortical thickness | NO_2_ | NP | Coefficients | - | - | -4.18 | NP | |  | 0.12 | NS |
|  |  | Female |  |  |  |  |  |  |  |  | -28.4 |  |  |  |  |  |
|  |  | Male |  |  |  | NO_x_ |  |  |  |  | -0.98 |  |  | - | 0.07 | NS |
|  |  | Female |  |  |  |  |  |  |  |  | -70.9 |  |  |  |  |  |
|  |  | Male |  |  |  | PM2.5 |  |  |  |  | -0.29 |  |  |  | 0.14 | NS |
|  |  | Female |  |  |  |  |  |  |  |  | -4.66 |  |  |  |  |  |
|  |  | Male |  |  |  | PM10 |  |  |  |  | -0.52 |  |  |  | 0.10 | NS |
|  |  | Female |  |  |  |  |  |  |  |  | -9.75 |  |  |  |  |  |
|  |  | Male |  |  |  | PM2.5-abs |  |  |  |  | -0.21 |  |  |  | 0.20 | NS |
|  |  | Female |  |  |  |  |  |  |  |  | -1.09 |  |  |  |  |  |
|  |  | Male |  |  |  | PMcoarse |  |  |  |  | -1.25 |  |  |  | 0.19 | NS |
|  |  | Female |  |  |  |  |  |  |  |  | -4.59 |  |  |  |  |  |
| Hedge et al., 2020 | | Male | Neuroimaging | MRI | Left Thalamus Volume | PM2.5 | Per IQR Increase | Coefficient | -10.09 | 0 | -10.09 | -22.99 | 2.82 | - | P > 0.05 | NS |
|  |  | Female |  |  |  |  |  |  | -10.09 | 6.85 | -3.24 | NP | |  |  |  |
|  |  | Male |  |  |  | PM2.5-10 |  |  | -9.6 | 0 | -9.6 | -24.07 | 4.88 |  |  |  |
|  |  | Female |  |  |  |  |  |  | -9.6 | -4.87 | -14.47 | NP | |  |  |  |
|  |  | Male |  |  |  | PM10 |  |  | -3.38 | 0 | -3.38 | -10.28 | 3.52 |  |  |  |
|  |  | Female |  |  |  |  |  |  | -3.38 | -1.38 | -4.76 | NP | |  |  |  |
|  |  | Male |  |  |  | NO_2_ |  |  | -0.13 | 0 | -0.13 | -2.03 | 1.78 |  |  |  |
|  |  | Female |  |  |  |  |  |  | -0.13 | 0.25 | 0.12 | NP | |  |  |  |
|  |  | Male |  |  |  | NO |  |  | -0.26 | 0 | -0.26 | -1.20 | 0.69 |  |  |  |
|  |  | Female |  |  |  |  |  |  | -0.26 | -0.11 | -0.37 | NP | |  |  |  |
|  |  | Male |  |  | Right Thalamus Volume | PM2.5 |  |  | -8.1 | 0 | -8.1 | -20.46 | 4.27 |  |  |  |
|  |  | Female |  |  |  |  |  |  | -8.1 | 3.32 | -4.78 | NP | |  |  |  |
|  |  | Male |  |  |  | PM2.5-10 |  |  | -1.66 | 0 | -1.66 | -15.53 | 12.20 |  |  |  |
|  |  | Female |  |  |  |  |  |  | -1.66 | -13.93 | -15.59 | NP | |  |  |  |
|  |  | Male |  |  |  | PM10 |  |  | 0.16 | 0 | 0.16 | -6.46 | 6.77 |  |  |  |
|  |  | Female |  |  |  |  |  |  | 0.16 | -5.46 | -5.3 | NP | |  |  |  |
|  |  | Male |  |  |  | NO_2_ |  |  | -0.37 | 0 | -0.37 | -2.20 | 1.45 |  |  |  |
|  |  | Female |  |  |  |  |  |  | -0.37 | 0.19 | -0.18 | NP | |  |  |  |
|  |  | Male |  |  |  | NO |  |  | -0.52 | 0 | -0.52 | -1.42 | 0.39 |  |  |  |
|  |  | Female |  |  |  |  |  |  | -0.52 | -0.01 | -0.53 | NP | |  |  |  |
| Gale et al., 2020*** | | Without effect of Gender | Neuro Imaging | MRI | Pole (Left) | PM2.5 | NP | Coefficient | -72.78 | 0 | -72.78 | NP | |  | P > 0.05 | NS |
|  |  | With effect of gender |  |  |  |  |  |  | -72.78 | 49.83 | -22.95 |  |  |  |  |  |
|  |  | Without Gender |  |  | Superior Gyrus (Left) |  |  |  | -1.61 | 0 | -1.61 |  |  |  |  |  |
|  |  | With gender |  |  |  |  |  |  | -1.61 | -21.36 | -22.97 |  |  |  |  |  |
|  |  | Without Gender |  |  | Medial Cortex (Left) |  |  |  | -3.69 | 0 | -3.69 |  |  |  |  |  |
|  |  | With gender |  |  |  |  |  |  | -3.69 | 3.64 | -0.05 |  |  |  |  |  |
|  |  | Without Gender |  |  | Orbital Cortex (Left) |  |  |  | -11.02 | 0 | -11.02 |  |  |  |  |  |
|  |  | With gender |  |  |  |  |  |  | -11.02 | 2.33 | -8.69 |  |  |  |  |  |
|  |  | Without Gender |  |  | Operculum Cortex (Left) |  |  |  | -6.31 | 0 | -6.31 |  |  |  |  |  |
|  |  | With gender |  |  |  |  |  |  | -6.31 | 2.49 | -3.82 |  |  |  |  |  |
|  |  | Without Gender |  |  | Pole (Right) |  |  |  | -55.71 | 0 | -55.71 |  |  |  |  |  |
|  |  | With gender |  |  |  |  |  |  | -55.71 | 22.03 | -33.68 |  |  |  |  |  |
|  |  | Without Gender |  |  | Superior Gyrus (Right) |  |  |  | -12.76 | 0 | -12.76 |  |  |  |  |  |
|  |  | With gender |  |  |  |  |  |  | -12.76 | 12.6 | -0.16 |  |  |  |  |  |
|  |  | Without Gender |  |  | Medial Cortex (Right) |  |  |  | -3.38 | 0 | -3.38 |  |  |  |  |  |
|  |  | With gender |  |  |  |  |  |  | -3.38 | 3.3 | -0.08 |  |  |  |  |  |
|  |  | Without Gender |  |  | Orbital Cortex (Right) |  |  |  | -8.78 | 0 | -8.78 |  |  |  |  |  |
|  |  | With gender |  |  |  |  |  |  | -8.78 | -2.99 | -11.77 |  |  |  |  |  |
|  |  | Without Gender |  |  | Operculum Cortex ((Right) |  |  |  | -0.09 | 0 | -0.09 |  |  |  |  |  |
|  |  | With gender |  |  |  |  |  |  | -0.09 | 2.76 | 2.67 |  |  |  |  |  |
|  |  | Without Gender |  |  | Pole (Left) | PM2.5-10 |  |  | -43.63 | 0 | -43.63 |  |  |  |  |  |
|  |  | With gender |  |  |  |  |  |  | -43.63 | 13.59 | -30.04 |  |  |  |  |  |
|  |  | Without Gender |  |  | Superior Gyrus (Left) |  |  |  | -33.29 | 0 | -33.29 |  |  |  |  |  |
|  |  | With gender |  |  |  |  |  |  | -33.29 | -9.9 | -43.19 |  |  |  |  |  |
|  |  | Without Gender |  |  | Medial Cortex (Left) |  |  |  | -9.57 | 0 | -9.57 |  |  |  |  |  |
|  |  | With gender |  |  |  |  |  |  | -9.57 | 9.17 | -0.4 |  |  |  |  |  |
|  |  | Without Gender |  |  | Orbital Cortex (Left) |  |  |  | -13.09 | 0 | -13.09 |  |  |  |  |  |
|  |  | With gender |  |  |  |  |  |  | -13.09 | 8.51 | -4.58 |  |  |  |  |  |
|  |  | Without Gender |  |  | Operculum Cortex (Left) |  |  |  | -7.16 | 0 | -7.16 |  |  |  |  |  |
|  |  | With gender |  |  |  |  |  |  | -7.16 | 4.09 | -3.07 |  |  |  |  |  |
|  |  | Without Gender |  |  | Pole (Right) |  |  |  | -63.25 | 0 | -63.25 |  |  |  |  |  |
|  |  | With gender |  |  |  |  |  |  | -63.25 | 4.84 | -58.41 |  |  |  |  |  |
|  |  | Without Gender |  |  | Superior Gyrus (Right) |  |  |  | -10.03 | 0 | -10.03 |  |  |  |  |  |
|  |  | With gender |  |  |  |  |  |  | -10.03 | -1.8 | -11.83 |  |  |  |  |  |
|  |  | Without Gender |  |  | Medial Cortex (Right) |  |  |  | -4.14 | 0 | -4.14 |  |  |  |  |  |
|  |  | With gender |  |  |  |  |  |  | -4.14 | 0.3 | -3.84 |  |  |  |  |  |
|  |  | Without Gender |  |  | Orbital Cortex (Right) |  |  |  | -14.58 | 0 | -14.58 |  |  |  |  |  |
|  |  | With gender |  |  |  |  |  |  | -14.58 | 8 | -6.58 |  |  |  |  |  |
|  |  | Without Gender |  |  | Operculum Cortex ((Right) |  |  |  | -2.48 | 0 | -2.48 |  |  |  |  |  |
|  |  | With gender |  |  |  |  |  |  | -2.48 | 1.36 | -1.12 |  |  |  |  |  |
|  |  | Without Gender |  |  | Pole (Left) | PM10 |  |  | -29.11 | 0 | -29.11 |  |  |  |  |  |
|  |  | With gender |  |  |  |  |  |  | -29.11 | 14.73 | -14.38 |  |  |  |  |  |
|  |  | Without Gender |  |  | Superior Gyrus (Left) |  |  |  | -2.54 | 0 | -2.54 |  |  |  |  |  |
|  |  | With gender |  |  |  |  |  |  | -2.54 | -12.72 | -15.26 |  |  |  |  |  |
|  |  | Without Gender |  |  | Medial Cortex (Left) |  |  |  | -3.22 | 0 | -3.22 |  |  |  |  |  |
|  |  | With gender |  |  |  |  |  |  | -3.22 | 3.14 | -0.08 |  |  |  |  |  |
|  |  | Without Gender |  |  | Orbital Cortex (Left) |  |  |  | -3.92 | 0 | -3.92 |  |  |  |  |  |
|  |  | With gender |  |  |  |  |  |  | -3.92 | 0.37 | -3.55 |  |  |  |  |  |
|  |  | Without Gender |  |  | Operculum Cortex (Left) |  |  |  | -4.41 | 0 | -4.41 |  |  |  |  |  |
|  |  | With gender |  |  |  |  |  |  | -4.41 | 2.14 | -2.27 |  |  |  |  |  |
|  |  | Without Gender |  |  | Pole (Right) |  |  |  | -31.2 | 0 | -31.2 |  |  |  |  |  |
|  |  | With gender |  |  |  |  |  |  | -31.2 | 8.6 | -22.6 |  |  |  |  |  |
|  |  | Without Gender |  |  | Superior Gyrus (Right) |  |  |  | 1.23 | 0 | 1.23 |  |  |  |  |  |
|  |  | With gender |  |  |  |  |  |  | 1.23 | -4.2 | -2.97 |  |  |  |  |  |
|  |  | Without Gender |  |  | Medial Cortex (Right) |  |  |  | -1.41 | 0 | -1.41 |  |  |  |  |  |
|  |  | With gender |  |  |  |  |  |  | -1.41 | 0.12 | -1.29 |  |  |  |  |  |
|  |  | Without Gender |  |  | Orbital Cortex (Right) |  |  |  | -5.03 | 0 | -5.03 |  |  |  |  |  |
|  |  | With gender |  |  |  |  |  |  | -5.03 | 2.15 | -2.88 |  |  |  |  |  |
|  |  | Without Gender |  |  | Operculum Cortex ((Right) |  |  |  | -0.18 | 0 | -0.18 |  |  |  |  |  |
|  |  | With gender |  |  |  |  |  |  | -0.18 | 0.91 | 0.73 |  |  |  |  |  |
|  |  | Without Gender |  |  | Pole (Left) | NO_2_ |  |  | -8.67 | 0 | -8.67 |  |  |  |  |  |
|  |  | With gender |  |  |  |  |  |  | -8.67 | 6.86 | -1.81 |  |  |  |  |  |
|  |  | Without Gender |  |  | Superior Gyrus (Left) |  |  |  | 2.5 | 0 | 2.5 |  |  |  |  |  |
|  |  | With gender |  |  |  |  |  |  | 2.5 | -2.28 | 0.22 |  |  |  |  |  |
|  |  | Without Gender |  |  | Medial Cortex (Left) |  |  |  | -0.11 | 0 | -0.11 |  |  |  |  |  |
|  |  | With gender |  |  |  |  |  |  | -0.11 | 0.59 | 0.48 |  |  |  |  |  |
|  |  | Without Gender |  |  | Orbital Cortex (Left) |  |  |  | -1.16 | 0 | -1.16 |  |  |  |  |  |
|  |  | With gender |  |  |  |  |  |  | -1.16 | 1 | -0.16 |  |  |  |  |  |
|  |  | Without Gender |  |  | Operculum Cortex (Left) |  |  |  | -0.69 | 0 | -0.69 |  |  |  |  |  |
|  |  | With gender |  |  |  |  |  |  | -0.69 | 0.21 | -0.48 |  |  |  |  |  |
|  |  | Without Gender |  |  | Pole (Right) |  |  |  | -9.18 | 0 | -9.18 |  |  |  |  |  |
|  |  | With gender |  |  |  |  |  |  | -9.18 | 5.67 | -3.51 |  |  |  |  |  |
|  |  | Without Gender |  |  | Superior Gyrus (Right) |  |  |  | 1.47 | 0 | 1.47 |  |  |  |  |  |
|  |  | With gender |  |  |  |  |  |  | 1.47 | -0.46 | 1.01 |  |  |  |  |  |
|  |  | Without Gender |  |  | Medial Cortex (Right) |  |  |  | -0.02 | 0 | -0.02 |  |  |  |  |  |
|  |  | With gender |  |  |  |  |  |  | -0.02 | 0.32 | 0.3 |  |  |  |  |  |
|  |  | Without Gender |  |  | Orbital Cortex (Right) |  |  |  | -0.92 | 0 | -0.92 |  |  |  |  |  |
|  |  | With gender |  |  |  |  |  |  | -0.92 | -0.07 | -0.99 |  |  |  |  |  |
|  |  | Without Gender |  |  | Operculum Cortex ((Right) |  |  |  | -0.1 | 0 | -0.1 |  |  |  |  |  |
|  |  | With gender |  |  |  |  |  |  | -0.1 | 0.3 | 0.2 |  |  |  |  |  |
|  |  | Without Gender |  |  | Pole (Left) | NO |  |  | -4.62 | 0 | -4.62 |  |  |  |  |  |
|  |  | With gender |  |  |  |  |  |  | -4.62 | 3.28 | -1.34 |  |  |  |  |  |
|  |  | Without Gender |  |  | Superior Gyrus (Left) |  |  |  | 0.66 | 0 | 0.66 |  |  |  |  |  |
|  |  | With gender |  |  |  |  |  |  | 0.66 | -1.55 | -0.89 |  |  |  |  |  |
|  |  | Without Gender |  |  | Medial Cortex (Left) |  |  |  | -0.17 | 0 | -0.17 |  |  |  |  |  |
|  |  | With gender |  |  |  |  |  |  | -0.17 | 0.33 | 0.16 |  |  |  |  |  |
|  |  | Without Gender |  |  | Orbital Cortex (Left) |  |  |  | -0.79 | 0 | -0.79 |  |  |  |  |  |
|  |  | With gender |  |  |  |  |  |  | -0.79 | 0.15 | -0.64 |  |  |  |  |  |
|  |  | Without Gender |  |  | Operculum Cortex (Left) |  |  |  | -0.39 | 0 | -0.39 |  |  |  |  |  |
|  |  | With gender |  |  |  |  |  |  | -0.39 | -0.01 | -0.4 |  |  |  |  |  |
|  |  | Without Gender |  |  | Pole (Right) |  |  |  | -4.9 | 0 | -4.9 |  |  |  |  |  |
|  |  | With gender |  |  |  |  |  |  | -4.9 | 1.87 | -3.03 |  |  |  |  |  |
|  |  | Without Gender |  |  | Superior Gyrus (Right) |  |  |  | 0.24 | 0 | 0.24 |  |  |  |  |  |
|  |  | With gender |  |  |  |  |  |  | 0.24 | -0.28 | -0.04 |  |  |  |  |  |
|  |  | Without Gender |  |  | Medial Cortex (Right) |  |  |  | -0.04 | 0 | -0.04 |  |  |  |  |  |
|  |  | With gender |  |  |  |  |  |  | -0.04 | 0.04 | 0 |  |  |  |  |  |
|  |  | Without Gender |  |  | Orbital Cortex (Right) |  |  |  | -0.66 | 0 | -0.66 |  |  |  |  |  |
|  |  | With gender |  |  |  |  |  |  | -0.66 | -0.39 | -1.05 |  |  |  |  |  |
|  |  | Without Gender |  |  | Operculum Cortex ((Right) |  |  |  | -0.13 | 0 | -0.13 |  |  |  |  |  |
|  |  | With gender |  |  |  |  |  |  | -0.13 | 0.12 | -0.01 |  |  |  |  |  |
| Chen et al., 2020 | | Male | Cognitive assessment test | MoCA-T (binary) | Score < 24 | PM2.5 | 2^nd^ tertile (29.00-29.98 ug/m^3^) | Odds ratio | - |  | 2.31 | 0.31 | 17.51 |  | NP | NS ‡ |
|  |  | Female |  |  |  |  |  |  |  |  | 5.72 | 1.03 | 31.69 | - |  |  |
|  |  | Male |  | MoCA-T (continuous) | Continuous score | PM2.5 | 2^nd^ tertile |  |  |  | -0.34 | -1.14 | 0.46 |  | 0.85 | NS |
|  |  | Female |  |  |  |  |  |  |  |  | -0.19 | -0.78 | 0.39 |  |  |  |
|  |  | Male |  |  |  |  | 3^rd^ tertile (> 29.98 ug/m^3^) |  |  |  | -0.44 | -1.18 | 0.29 |  | 0.09 |  |
|  |  | Female |  |  |  |  |  |  |  |  | -0.83 | -1.53 | -0.14 |  |  |  |
|  |  | Male |  | Trail Making Test A |  | PM10 | 2^nd^ tertile (49.57 – 51.20 ug/m^3^) |  |  |  | -0.22 | -0.43 | -0.01 |  | 0.47 |  |
|  |  | Female |  |  |  |  |  |  |  |  | -0.02 | -0.20 | 0.15 |  |  |  |
|  |  | Male |  |  |  |  | 3^rd^ tertile (51.20 ug/m^3^) |  |  |  | -0.44 | -0.72 | -0.15 |  | 0.22 |  |
|  |  | Female |  |  |  |  |  |  |  |  | -0.05 | -0.26 | 0.16 |  |  |  |
|  |  | Male |  | Verbal Fluency |  | PMcoarse | 2^nd^ tertile (22.45 – 26.50 ug/m^3^) |  |  |  | -0.08 | -0.31 | 0.15 |  | 0.52 |  |
|  |  | Female |  |  |  |  |  |  |  |  | -0.24 | -0.47 | -0.01 |  |  |  |
|  |  | Male |  |  |  |  |  |  |  |  | -0.24 | -0.50 | 0.01 |  | 0.48 |  |
|  |  | Female |  |  |  |  | 3^rd^ tertile (T3 > 26.50 ug/m^3^) |  |  |  | -0.20 | -0.45 | 0.05 |  |  |  |
|  |  | Male |  |  |  | NO_2_ | 2^nd^ tertile (T2 = 27.47 -28.62 ug/m^3^  ) |  |  |  | 0.22 | 0.05 | 0.40 |  | 0.31 |  |
|  |  | Female |  |  |  |  |  |  |  |  | 0.03 | -0.13 | 0.19 |  |  |  |
|  |  | Male |  |  |  | NO_2_ | 3^rd^ tertile (T3 > 28.62 ug/m^3^) |  |  |  | 0.08 | -0.15 | 0.31 |  | 0.63 |  |
|  |  | Female |  |  |  |  |  |  |  |  | -0.09 | -0.33 | 0.15 |  |  |  |
| Wang et al., 2020 | | Male | Cognitive assessment test | MMSE | Score ≥ 18 | PM2.5 | NP | Hazard Ratio | - | - | 1.073 | 1.028 | 1.12 |  | 0.06 | NS |
|  |  | Female |  |  |  |  |  |  |  |  | 1.036 | 1.001 | 1.073 |  |  |  |
| Kulick et al., 2020 | | Male | Cognitive assessment test | Test batteries from WHICAP | Change in the test score | NO_2_ | Per IQR (11.2 ppb) increase | Coefficient | - | - | -0.08 | -0.10 | -0.05 |  | 0.27 | NS |
|  |  | Female |  |  |  |  |  |  |  |  | -0.07 | -0.10 | -0.05 |  |  |  |
|  |  | Male |  |  |  | PM2.5 | Per IQR (4.42 ug/m^3^) increase |  |  |  | -0.091 | -0.12 | -0.07 |  | 0.39 |  |
|  |  | Female |  |  |  |  |  |  |  |  | -0.094 | -0.12 | -0.07 |  |  |  |
|  |  | Male |  |  |  | PM10 | Per IQR (7.95 ug/m^3^) increase |  |  |  | -0.047 | -0.06 | -0.03 |  | 0.22 |  |
|  |  | Female |  |  |  |  |  |  |  |  | -0.042 | -0.06 | -0.02 |  |  |  |
| Shi et al., 2020 | | Men | Alzheimer’s disease and related dementia (ADRD) | Hospital admission code (ICD-9: 331·0, 290; ICD-10: G30·9,  and F05) | Diagnosis of Alzheimer’s disease and related dementia | PM2.5 | Per 5 μg/m3 increase | Hazard Ratio | - | - | NP | NP | NP | Positive | NP | S |
|  |  | Women |  |  |  |  |  |  |  |  |  |  |  |  |  |  |
| Yuchi et al., 2020 | | Male | Medical Record (ICD 9^th^) | | Diagnosis of Non-Alzheimer’s dementia | Highway <50m | NP | Hazard Ratio | - | - | 1.07 | 0.88 | 1.29 |  | NP | NS |
|  |  | Female |  |  |  |  |  |  |  |  | 1.02 | 0.86 | 1.21 |  |  |  |
|  |  | Male |  |  |  | Highway <50m + Greenness |  |  |  |  | 1.06 | 0.84 | 1.35 |  |  |  |
|  |  | Female |  |  |  |  |  |  |  |  | 1.03 | 0.84 | 1.28 |  |  |  |
|  |  | Male |  |  |  | Highway <150m |  |  |  |  | 1.11 | 0.98 | 1.25 |  |  |  |
|  |  | Female |  |  |  |  |  |  |  |  | 1.09 | 0.99 | 1.21 |  |  |  |
|  |  | Male |  |  |  | Highway <150m + Greenness |  |  |  |  | 1.07 | 0.92 | 1.24 |  |  |  |
|  |  | Female |  |  |  |  |  |  |  |  | 1.04 | 0.91 | 1.18 |  |  |  |
|  |  | Male |  |  |  | Major road <50m |  |  |  |  | 1.14 | 1.03 | 1.28 |  |  |  |
|  |  | Female |  |  |  |  |  |  |  |  | 1.17 | 1.06 | 1.3 |  |  |  |
|  |  | Male |  |  |  | Major road <50m + Greenness |  |  |  |  | 1.09 | 0.96 | 1.24 |  |  |  |
|  |  | Female |  |  |  |  |  |  |  |  | 1.16 | 1.06 | 1.26 |  |  |  |
|  |  | Male |  |  |  | Major road <50m or Highway <150m |  |  |  |  | 1.14 | 1.04 | 1.25 |  |  |  |
|  |  | Female |  |  |  |  |  |  |  |  | 1.13 | 1.05 | 1.21 |  |  |  |
|  |  | Male |  |  |  | Major road <50m or Highway <150m + Greenness |  |  |  |  | 1.09 | 0.98 | 1.22 |  |  |  |
|  |  | Female |  |  |  |  |  |  |  |  | 1.13 | 1.02 | 1.23 |  |  |  |
|  |  | Male |  |  |  | PM2.5 (μg/m³) | Per IQR (1.5 ug/m^3^) |  |  |  | 0.98 | 0.94 | 1.04 |  |  |  |
|  |  | Female |  |  |  |  |  |  |  |  | 1.04 | 0.99 | 1.09 |  |  |  |
|  |  | Male |  |  |  | PM2.5 (μg/m³) + Noise |  |  |  |  | 0.98 | 0.93 | 1.04 |  |  |  |
|  |  | Female |  |  |  |  |  |  |  |  | 1.04 | 0.99 | 1.09 |  |  |  |
|  |  | Male |  |  |  | PM2.5 (μg/m³) + Greenness |  |  |  |  | 0.98 | 0.93 | 1.03 |  |  |  |
|  |  | Female |  |  |  |  |  |  |  |  | 1.04 | 0.99 | 1.09 |  |  |  |
|  |  | Male |  |  |  | Black carbon (μg/m³) | Per IQR (1.2 ug/m^3^) increase |  |  |  | 1.01 | 0.96 | 1.05 |  |  |  |
|  |  | Female |  |  |  |  |  |  |  |  | 1.02 | 0.97 | 1.06 |  |  |  |
|  |  | Male |  |  |  | Black carbon (μg/m³) + Noise |  |  |  |  | 1.00 | 0.94 | 1.04 |  |  |  |
|  |  | Female |  |  |  |  |  |  |  |  | 1.03 | 0.98 | 1.07 |  |  |  |
|  |  | Male |  |  |  | Black carbon (μg/m³) + Greenness |  |  |  |  | 1.00 | 0.96 | 1.05 |  |  |  |
|  |  | Female |  |  |  |  |  |  |  |  | 1.02 | 0.97 | 1.05 |  |  |  |
|  |  | Male |  |  |  | NO_2_ (ppb) | Per IQR (9.1 ppb) increase |  |  |  | 1.00 | 0.94 | 1.06 |  |  |  |
|  |  | Female |  |  |  |  |  |  |  |  | 1.05 | 0.99 | 1.1 |  |  |  |
|  |  | Male |  |  |  | NO_2_ (ppb) + Noise |  |  |  |  | 0.98 | 0.91 | 1.04 |  |  |  |
|  |  | Female |  |  |  |  |  |  |  |  | 1.04 | 0.98 | 1.1 |  |  |  |
|  |  | Male |  |  |  | NO_2_ (ppb) + Greenness |  |  |  |  | 0.99 | 0.94 | 1.06 |  |  |  |
|  |  | Female |  |  |  |  |  |  |  |  | 1.05 | 0.99 | 1.1 |  |  |  |
|  |  | Male |  |  |  | NO (ppb) | Per IQR (12.0 ppb) increase |  |  |  | 0.99 | 0.93 | 1.05 |  |  |  |
|  |  | Female |  |  |  |  |  |  |  |  | 1.01 | 0.96 | 1.06 |  |  |  |
|  |  | Male |  |  |  | NO (ppb) + Noise |  |  |  |  | 0.96 | 0.9 | 1.03 |  |  |  |
|  |  | Female |  |  |  |  |  |  |  |  | 1.02 | 0.95 | 1.08 |  |  |  |
|  |  | Male |  |  |  | NO (ppb) + Greenness |  |  |  |  | 1.01 | 0.98 | 1.05 |  |  |  |
|  |  | Female |  |  |  |  |  |  |  |  | 1.01 | 0.98 | 1.04 |  |  |  |
| Laccarino et al., 2021 | | Male | Clinical data from the IDEAS Study | Alzheimer disease (AD) | amyloid PET scan positivity (as a feature of AD) | PM2.5 | Per IQR increase | Z-score | - | 1.01  (95% CI: 0.99 – 1.04) | - | - | - | NP (Not stronger in female than male) | NP | NS |
|  |  | Female |  |  |  |  |  |  |  |  |  |  |  |  |  |  |
| Mortamais et al., 2021 | | Male | 3-step procedure | All cause dementia, AD, Vascular/mixed dementia(VaD) | Diagnosis of all cause dementia, AD, VaD | PM2.5 | Per 5 μg/m3 increase | Hazard Ratio | - | - | - | - | - | NP  (Not modified by sex) | NP | NS |
|  |  | Female |  |  |  |  |  |  |  |  |  |  |  |  |  |  |
| Shi et al., 2021§ | | Male | Medicare record data | Dementia | Diagnosis of dementia | PM2.5 | Per IQR increase | Hazard Ratio | - | - | NP | - | - | - | Reference | NS |
|  |  | Female |  |  |  |  |  |  |  |  |  |  |  |  | 0.64 |  |
|  |  | Male |  |  |  | NO2 |  |  |  |  |  |  |  | Negative | Reference | S |
|  | | Female |  |  |  |  |  |  |  |  |  |  |  |  | 0.003 |  |
|  | | Male |  |  |  | O3 |  |  |  |  |  |  |  |  | Reference | NS |
|  | | Female |  |  |  |  |  |  |  |  |  |  |  |  | 0.05 |  |
|  | | Male |  | Alzheimer’s disease (AD) | Diagnosis of AD | PM2.5 |  |  |  |  |  |  |  |  | Reference | NS |
|  | | Female |  |  |  |  |  |  |  |  |  |  |  |  | 0.68 |  |
|  | | Male |  |  |  | NO2 |  |  |  |  |  |  |  |  | Reference | NS |
|  | | Female |  |  |  |  |  |  |  |  |  |  |  |  | 0.33 |  |
|  | | Male |  |  |  | O3 |  |  |  |  |  |  |  |  | Reference | NS |
|  | | Female |  |  |  |  |  |  |  |  |  |  |  |  | 0.81 |  |
| Yao et al., 2021 | | Male | Cognitive assessment test | Chinese MMSE* | Score ≥ 25 | Distance to the main roadway | <=100m vs. >100m | Odds ratio | - | - | 1.19 | 1.02 | 1.4 |  | 0.75 | NS |
|  |  | Female |  |  |  |  |  |  |  |  | 1.23 | 1.07 | 1.41 | - |  |  |
| Ran et al., 2021 | | Male | Medical record (ICD-9^th^) | | Diagnosis of all cause dementia, Alzheimer’s disease, and vascular dementia | PM2.5 | Per IQR (3.8 ug/m^3^) increase | Hazard Ratio | - | - | 1.03 | 0.9 | 1.18 |  | 0.069 | NS |
|  |  | Female |  |  |  |  |  |  |  |  | 1.08 | 1 | 1.15 | - |  |  |
| Shaffer et al., 2021 | | Male | Dementia assessment test | CASI* | Score below 86 | PM2.5 | Per IQR (1.0 ug/m^3^) increase | Hazard Ratio | - | - | 1.23 | 1.08 | 1.39 | - | 0.007 | S |
|  |  | Female |  |  |  |  |  |  |  |  | 1.13 | 1 | 1.28 |  |  |  |
| Nunez et al., 2021 | | Male | Hospitalization records  (coded with ICD-9) | | Diagnosis of incident Alzheimer’s disease | PM2.5 | 5.8 – 8.1 ug/m^3^ | Rate ratio | - | - | 1.06 | 0.97 | 1.15 | - | NP | NS |
|  |  | Female |  |  |  |  |  |  |  |  | 1.07 | 0.98 | 1.16 |  |  |  |
|  |  | Male |  |  |  |  | 8.1 – 10.4 ug/m^3^ |  |  |  | 1.04 | 0.99 | 1.11 |  |  |  |
|  |  | Female |  |  |  |  |  |  |  |  | 1.06 | 1.01 | 1.12 |  |  |  |
| G.-C. Chen et al., 2022 | | Male | Hospitalization records  (coded with ICD-9 and ICD-10) | Dementia | Incident all-cause dementia | Air pollution score  (PM2.5, PM2.5-10, PM10, NO_2_, NO_x_) | Per IQR increase | Hazard Ratio | - | - | 1.11 | 1.06 | 1.16 | - | 0.88 | NS |
|  |  | Female |  |  |  |  |  |  |  |  | 1.12 | 1.07 | 1.17 |  |  |  |
|  |  | Male |  |  | Incident AD |  |  |  |  |  | 1.12 | 1.085 | 1.20 | - | 0.84 | NS |
|  |  | Female |  |  |  |  |  |  |  |  | 1.14 | 1.06 | 1.22 |  |  |  |
|  |  | Male |  |  | Incident vascular dementia |  |  |  |  |  | 1.11 | 1.02 | 1.20 | - | 0.998 | NS |
|  |  | Female |  |  |  |  |  |  |  |  | 1.13 | 1.02 | 1.24 |  |  |  |
| Hu et al., 2022 | | Male | Cognitive assessment test | episodic memory, orientation and attention, and visuospatial ability | A positive difference (Δ cognitive function >0) between the  2011 and 2015 cognitive function assessments; | PM 1 | Per IQR increase | Odd Ratio | - | - | 0.78 | 0.74 | 0.83 | - | 0.29 | NS |
|  |  | Female |  |  |  |  |  |  |  |  | 0.79 | 0.74 | 0.84 |  |  |  |
|  |  | Male |  |  |  | PM2.5 |  |  |  |  | 0.73 | 0.69 | 0.77 |  | 0.21 |  |
|  |  | Female |  |  |  |  |  |  |  |  | 0.75 | 0.7 | 0.8 |  |  |  |
|  |  | Male |  |  |  | PM10 |  |  |  |  | 0.83 | 0.79 | 0.88 |  | 0.28 |  |
|  |  | Female |  |  |  |  |  |  |  |  | 0.81 | 0.76 | 0.87 |  |  |  |
|  |  | Male |  |  |  | NO_2_ |  |  |  |  | 0.85 | 0.81 | 0.9 |  | 0.30 |  |
|  |  | Female |  |  |  |  |  |  |  |  | 0.83 | 0.78 | 0.89 |  |  |  |
|  |  | Male |  |  |  | NO |  |  |  |  | 1.04 | 0.99 | 1.1 |  | 0.96 |  |
|  |  | Female |  |  |  |  |  |  |  |  | 0.99 | 0.93 | 1.06 |  |  |  |
| Gao et al., 2022 | | Male | Cognitive assessment test | Chinese MMSE | Score ≥ 18 + declined ≥ 4 points | O_3_ | Per 10.4 ug/m^3^ increase | Hazard Ratio | - | - | 1.174 | 1.072 | 1.285 |  | 0.08 | NS |
|  |  | Female |  |  |  |  |  |  |  |  | 1.072 | 1.001 | 1.147 | - |  |  |
| M. Li et al., 2022 | | Male | CSF sTREM2 score | Neuroinflammatory (a biomarker of AD, represented by CSF sTREM2 score) | Continuous score | PM2.5 | NP | Z score | - | - | -0.149 | NP | NP | Negative  (stronger in Male) | 0.0002 | S |
|  |  | Female |  |  |  |  |  |  |  |  | -0.057 | NP | NP |  | 0.2330 |  |
| Lee et al., 2022 | | Male | Cognitive assessment test | MMSE | Continuous score | PM 2.5 | Per IQR change | Coefficient | - | - | -0.04 | -0.09 | 0.01 |  | < 0.0001 | S |
|  |  | Female |  |  |  |  |  |  |  |  | -0.16 | -0.22 | -0.09 |  |  |  |
|  |  | Male |  |  |  | PM10 |  |  |  |  | -0.05 | -0.12 | 0.01 |  | < 0.0001 |  |
|  |  | Female |  |  |  |  |  |  |  |  | -0.12 | -0.2 | -0.04 |  |  |  |
| Yang et al., 2022 | | Male | Combination of MMSE*, MoCA*, and MRI | | Diagnosis of Alzheimer’s disease | PM2.5 | Per 10 ug/m^3^ increase | Hazard Ratio | - | - | 1.03 | 1.01 | 1.12 |  | NP | NS |
|  |  | Female |  |  |  |  |  |  |  |  | 0.94 | 0.81 | 1.08 |  |  |  |
| Semmens et al., 2022 | | Male/Female | Neuroimaging | MRI | Incident dementia | PM2.5, NO_2_ | Per IQR increase | Hazard Ratio |  |  | NP | NP | NP |  | NP | NS |
| Shi et al., 2023 | | Male | Medicare record data | Dementia | Diagnosis of dementia | Nitrate | Per IQR increase | Hazard Ratio | - | - | NP | NP | NP | Negative (Stronger in Male) | NP | S |
|  |  | Female |  |  |  |  |  |  |  |  |  |  |  |  |  |  |
|  |  | Male |  |  |  | Sulfate |  |  |  |  |  |  |  |  |  |  |
|  |  | Female |  |  |  |  |  |  |  |  |  |  |  |  |  |  |
|  |  | Male |  |  |  | Ammonium |  |  |  |  |  |  |  |  |  |  |
|  |  | Female |  |  |  |  |  |  |  |  |  |  |  |  |  |  |
|  | * AD: Alzheimer's Disease; CCFM: Chicago Cognitive Function Measure; MRI: Magnetic Resonance Imaging; MMSE: Mini-Mental State Examination; WM: White Matter; IADL: Impaired Activities of Daily Living; HbA1c: Glycosylated hemoglobin; CRP: C-reactive Protein; CVD: Cardiovascular Disease; SEVLT: Spanish English Verbal Learning Test; DSMMD: Diagnostic and Statistical Manual of Mental Disorders; 3WMT: Three-word memory test; SVF: Semantic Verbal fluency; MCI: Mild Cognitive Function; HV: Hippocampal Volumes; DADL: Disability in activities of daily living; HFE: Hemochromatosis gene; VaD: Vascular Alzheimer’s Disease; NINCDS-ADRDA: National Institute of Neurological and Communicative Diseases and Stroke/Alzheimer’s Disease and Related Disorders Association; NINDS-AIREN: National Institute of Neurological Disorders and Stroke and the Association Internationale pour la Recherche et I’Enseignement en Neurosicenes; CERAD: The Consortium to Establish a Registry for Alzheimer’s Disease; SNPs: Single Nucleotide Polymorphism | | | | | | | | | | | | | | | |
|  | ** CI: Confidence Interval; S/NS: Significance/Non-Significance; IQR: Inter-Quartile Range; RD: Residential Distance; NP: Not provided | | | | | | | | | | | | | | | |
|  | *** The total effect size (A+C) was manually calculated unless the effect of air pollution exposure on each category of the effect modifier was provided in the studies | | | | | | | | | | | | | | | |
|  | **** 95% CI is based on coefficient of effect modifiers  ‡ Gatto et al., 2014: Judgement on Statistical significance was based on in-text information of overlapping 95% CIs (no p-value for interaction term provided); Chen et al., 2020: Statistical significance was Judged by overlapping 95% Cis  ※11 studies tested gender as an effect modifier but either 1) did not provide effect size measures in the tables, but tested and mentioned non significance (Mortamais et al., 2021; Chen et al., 2017; Shi et al., 2023, Alishire & Crimmins, 2014; Oudin et al., 2019; Laccarino et al., 2021) or 2) significance (Shin et al., 2019; Shi et al., 2020) or 3) only provided graphics without the measures (Tzivian, Dlugaj, Winkler, Weinmayr, et al., 2016) or 4) or provided p-value testing subgroup analysis and reported significance (Shi et al., 2021).  Significant studies: Shi et al., 2021; Shin et al., 2019; Shi et al., 2020 (women showed higher ADRD risk of PM2.5 exposure); Shi et al., 2023 (significance of the modifiers in the table above is based on in-text reporting of the original study)  § Instead of P-value for interaction, p-values for testing null hypothesis that tests associations are the same between study subgroups were provided | | | | | | | | | | | | | | | |

- Table E3: Effect size by potential effect modifier - Education (Significant studies: 4/20)

| **Study** | | **Effect Modifier** | | **Outcome** | | | | **Air**  **Pollutant** | **Exposure**  **Level** | **Types of Effect** | **Effect**  **Size**  **(A: Main Effect by exposure)** | **Effect Size**  **(C: Effect by interaction term)** | **Effect Size  Estimate*****  **(A+C)** | **95% CI*s** | | **Direction of Modifier Measure** | **p-value  for  interaction terms** | **Statistical Significance (S/NS**)** |
| --- | --- | --- | --- | --- | --- | --- | --- | --- | --- | --- | --- | --- | --- | --- | --- | --- | --- | --- |
|  |  | **Education** | | **Outcome Measure Method** | | | **Outcome Variable** |  |  |  |  |  |  | **lower** | **upper** |  |  |  |
| Wellenius et al., 2012 | | Highschool or less | | Cognitive assessment test | | MMSE* | Score ≥ 26 | Proximity to the major roads | NP** | coefficient | - | - | 0.86 | 0.66 | 1.12 | Positive | 0.007 | S |
|  |  | College or more | |  |  |  |  |  |  |  |  |  | 1.54 | 1.1 | 2.17 |  |  |  |
| Ailshire & Crimmins, 2014 | | Education | years | Cognitive assessment test | | Global Cognitive Score | Continuous score | PM2.5 | Per quartiles | Coefficient | - | - | Not provided | - | - | - | - | NS |
| Tzivian, Dlugaj, Winkler, Hennig, et al., 2016 | | Years of education | Low and medium (≤10 and 11–13 yr) | Cognitive assessment test | | Global Cognitive Score | Continuous score | PM2.5 | Per IQR increase | Coefficient | - | - | (reference) | - | - | - | - | NS |
|  |  |  | High (≥14 yr) |  |  |  |  |  |  |  |  |  | negative |  |  |  |  |  |
| Tzivian, Dlugaj, Winkler, Weinmayr, et al., 2016 | | Years of education | Low and medium (≤10 and 11–13 yr) | Cognitive assessment test | | Diagnosis of cognitive impairment | Cognitive impairment | PM2.5 | Per IQR increase | Odd Ratio | - | - | (reference) | - | - | - | - | NS |
|  |  |  | High (≥14 yr) |  |  |  |  |  |  |  |  |  | negative |  |  |  |  |  |
| Cerza et al., 2019 | | Level of education | Primary/junior high/high/university | Hospitalization records  (coded with ICD-9) | | | Diagnosis of dementia | NO_x_ | Per 10 μg/m^3^ increase | Odds ratio | - | - | - | - | - | - | - | NS |
| Salinas-Rodriguez et al., 2018 | | Years of formal education | <9 | Cognitive assessment test | | 3WMT* | remembering any of the words in the 3WMT* | PM 2.5 | Per 10 μg/m3 increase | Odd ratio | - | - | 1.37 | 1.06 | 1.75 | - | 0.14 | NS |
|  |  |  | ≥9 |  |  |  |  |  |  |  |  |  | 1.34 | 1.05 | 1.71 |  |  |  |
|  |  |  | <9 |  |  | Semantic verbal fluency test | Continuous |  |  | Coefficient |  |  | -0.64 | -0.98 | -0.3 | - | 0.63 |  |
|  |  |  | ≥9 |  |  |  |  |  |  |  |  |  | -0.69 | -1.02 | -0.35 |  |  |  |
| Shin et al., 2019 | | Years of education | <9 | Cognitive assessment test | | Korean MMSE | Cognitive score | PM2.5 | Per IQR increase | Coefficients | - | - | - | NP | | - | NP | NS |
|  |  |  | ≥9 |  |  |  |  |  |  |  |  |  |  |  |  |  |  |  |
| Hedges et al., 2019*** | | No college degree | | Neuroimaging | | MRI* | Left Thalamus | PM 2.5 | Per 1-unit increase | Coefficient | -10.79 | 0 | -10.79 | NP | |  | P > 0.05 | NS |
|  |  | College degree | |  |  |  |  |  |  |  | -10.79 | 0.03 | -10.76 |  |  |  |  |  |
|  |  | No college degree | |  |  |  | Right Thalamus |  |  |  | -0.99 | 0 | -0.99 |  |  |  |  |  |
|  |  | College degree | |  |  |  |  |  |  |  | -0.99 | -2.42 | -3.41 |  |  |  |  |  |
|  |  | No college degree | |  |  |  | Left Thalamus | PM 2.5-10 |  |  | -5.64 | 0 | -5.64 |  |  |  |  |  |
|  |  | College degree | |  |  |  |  |  |  |  | -5.64 | -2.47 | -8.11 |  |  |  |  |  |
|  |  | No college degree | |  |  |  | Right Thalamus |  |  |  | -3.72 | 0 | -3.72 |  |  |  |  |  |
|  |  | College degree | |  |  |  |  |  |  |  | -3.72 | -4.21 | -7.93 |  |  |  |  |  |
|  |  | No college degree | |  |  |  | Left Thalamus | PM 10 |  |  | -2.3 | 0 | -2.3 |  |  |  |  |  |
|  |  | College degree | |  |  |  |  |  |  |  | -2.3 | -0.85 | -3.15 |  |  |  |  |  |
|  |  | No college degree | |  |  |  | Right Thalamus |  |  |  | 0.43 | 0 | 0.43 |  |  |  |  |  |
|  |  | College degree | |  |  |  |  |  |  |  | 0.43 | -2.58 | -2.15 |  |  |  |  |  |
|  |  | No college degree | |  |  |  | Left Thalamus | NO_2_ |  |  | -1.27 | 0 | -1.27 |  |  |  |  |  |
|  |  | College degree | |  |  |  |  |  |  |  | -1.27 | 0.96 | -0.31 |  |  |  |  |  |
|  |  | No college degree | |  |  |  | Right Thalamus |  |  |  | -0.08 | 0 | -0.08 |  |  |  |  |  |
|  |  | College degree | |  |  |  |  |  |  |  | -0.08 | 0.67 | 0.59 |  |  |  |  |  |
|  |  | No college degree | |  |  |  | Left Thalamus | NO |  |  | -0.72 | 0 | -0.72 |  |  |  |  |  |
|  |  | College degree | |  |  |  |  |  |  |  | -0.72 | 0.5 | -0.22 |  |  |  |  |  |
|  |  | No college degree | |  |  |  | Right Thalamus |  |  |  | -0.11 | 0 | -0.11 |  |  |  |  |  |
|  |  | College degree | |  |  |  |  |  |  |  | -0.11 | -0.39 | -0.5 |  |  |  |  |  |
| Hedge 2020 | | No college degree | | Neuroimaging | | MRI | Left Thalamus Volume | PM2.5 | Per IQR Increase | Coefficient | -19.11 | 0 | -19.11 | NP | | Qualitative | P<0.01 | S |
|  |  | College degree | |  |  |  |  |  |  |  | -19.11 | 23.59 | 4.48 |  |  |  |  |  |
|  |  | No college degree | |  |  |  |  | PM2.5-10 |  |  | -11.55 | 0 | -11.55 |  |  | - | P>0.05 | NS |
|  |  | College degree | |  |  |  |  |  |  |  | -11.55 | -1.12 | -12.67 |  |  |  |  |  |
|  |  | No college degree | |  |  |  |  | PM10 |  |  | -7.29 | 0 | -7.29 |  |  |  |  |  |
|  |  | College degree | |  |  |  |  |  |  |  | -7.29 | 6.66 | -0.63 |  |  |  |  |  |
|  |  | No college degree | |  |  |  |  | NO_2_ |  |  | -2.32 | 0 | -2.32 |  |  | Qualitative | P<0.01 | S |
|  |  | College degree | |  |  |  |  |  |  |  | -2.32 | 4.24 | 1.92 |  |  |  |  |  |
|  |  | No college degree | |  |  |  |  | NO |  |  | -1.31 | 0 | -1.31 |  |  | Qualitative | P<0.01 |  |
|  |  | College degree | |  |  |  |  |  |  |  | -1.31 | 1.89 | 0.58 |  |  |  |  |  |
|  |  | No college degree | |  |  |  | Right Thalamus Volume | PM2.5 |  |  | -17.11 | 0 | -17.11 |  |  | Qualitative | P<0.05 |  |
|  |  | College degree | |  |  |  |  |  |  |  | -17.11 | 20.17 | 3.06 |  |  |  |  |  |
|  |  | No college degree | |  |  |  |  | PM2.5-10 |  |  | -10.22 | 0 | -10.22 |  |  | - | P>0.05 | NS |
|  |  | College degree | |  |  |  |  |  |  |  | -10.22 | 3.36 | -6.86 |  |  |  |  |  |
|  |  | No college degree | |  |  |  |  | PM10 |  |  | -6.42 | 0 | -6.42 |  |  | - |  |  |
|  |  | College degree | |  |  |  |  |  |  |  | -6.42 | 7.92 | 1.5 |  |  |  |  |  |
|  |  | No college degree | |  |  |  |  | NO_2_ |  |  | -2.54 | 0 | -2.54 |  |  | Qualitative | P<0.01 | S |
|  |  | College degree | |  |  |  |  |  |  |  | -2.54 | 4.13 | 1.59 |  |  |  |  |  |
|  |  | No college degree | |  |  |  |  | NO |  |  | -1.46 | 0 | -1.46 |  |  | Qualitative | P<0.01 |  |
|  |  | College degree | |  |  |  |  |  |  |  | -1.46 | 1.79 | 0.33 |  |  |  |  |  |
| Gale et al., 2020*** | | No college degree | | Neuroimaging | | MRI | Pole (Left) | PM 2.5 | NP | Coefficient | -95.47 | 0 | -95.47 | NP | | Negative | P<0.01 | S |
|  |  | College degree | |  |  |  |  |  |  |  | -95.47 | 90.87 | -4.6 |  |  |  |  |  |
|  |  | No college degree | |  |  |  | Superior Gyrus (Left) |  |  |  | -65.01 | 0 | -65.01 |  |  | Qualitative | P<0.001 |  |
|  |  | College degree | |  |  |  |  |  |  |  | -65.01 | 98.67 | 33.66 |  |  |  |  |  |
|  |  | No college degree | |  |  |  | Medial Cortex (Left) |  |  |  | -3.92 | 0 | -3.92 |  |  | Qualitative | P>0.05 |  |
|  |  | College degree | |  |  |  |  |  |  |  | -3.92 | 3.95 | 0.03 |  |  |  |  |  |
|  |  | No college degree | |  |  |  | Orbital Cortex (Left) |  |  |  | -19.83 | 0 | -19.83 |  |  | Negative |  |  |
|  |  | College degree | |  |  |  |  |  |  |  | -19.83 | 18.84 | -0.99 |  |  |  |  |  |
|  |  | No college degree | |  |  |  | Operculum Cortex (Left) |  |  |  | -10.62 | 0 | -10.62 |  |  | Negative | P<0.01 |  |
|  |  | College degree | |  |  |  |  |  |  |  | -10.62 | 10.52 | -0.1 |  |  |  |  |  |
|  |  | No college degree | |  |  |  | Pole (Right) |  |  |  | -104.78 | 0 | -104.78 |  |  | Qualitative |  |  |
|  |  | College degree | |  |  |  |  |  |  |  | -104.78 | 113.65 | 8.87 |  |  |  |  |  |
|  |  | No college degree | |  |  |  | Superior Gyrus (Right) |  |  |  | -45.69 | 0 | -45.69 |  |  | Qualitative | P<0.001 |  |
|  |  | College degree | |  |  |  |  |  |  |  | -45.69 | 74.15 | 28.46 |  |  |  |  |  |
|  |  | No college degree | |  |  |  | Medial Cortex (Right) |  |  |  | -3.19 | 0 | -3.19 |  |  | - | P>0.05 | NS |
|  |  | College degree | |  |  |  |  |  |  |  | -3.19 | 2.82 | -0.37 |  |  |  |  |  |
|  |  | No college degree | |  |  |  | Orbital Cortex (Right) |  |  |  | -24.38 | 0 | -24.38 |  |  | Qualitative | P<0.01 | S |
|  |  | College degree | |  |  |  |  |  |  |  | -24.38 | 26.47 | 2.09 |  |  |  |  |  |
|  |  | No college degree | |  |  |  | Operculum Cortex ((Right) |  |  |  | -2.63 | 0 | -2.63 |  |  | Qualitative | P<0.05 |  |
|  |  | College degree | |  |  |  |  |  |  |  | -2.63 | 7.45 | 4.82 |  |  |  |  |  |
|  |  | No college degree | |  |  |  | Pole (Left) | PM 2.5-10 |  |  | -22.94 | 0 | -22.94 |  |  |  | P>0.05 | NS |
|  |  | College degree | |  |  |  |  |  |  |  | -22.94 | -30.68 | -53.62 |  |  |  |  |  |
|  |  | No college degree | |  |  |  | Superior Gyrus (Left) |  |  |  | -38.78 | 0 | -38.78 |  |  |  |  |  |
|  |  | College degree | |  |  |  |  |  |  |  | -38.78 | 1.07 | -37.71 |  |  |  |  |  |
|  |  | No college degree | |  |  |  | Medial Cortex (Left) |  |  |  | -3.48 | 0 | -3.48 |  |  |  |  |  |
|  |  | College degree | |  |  |  |  |  |  |  | -3.48 | -3.25 | -6.73 |  |  |  |  |  |
|  |  | No college degree | |  |  |  | Orbital Cortex (Left) |  |  |  | -1.09 | 0 | -1.09 |  |  |  |  |  |
|  |  | College degree | |  |  |  |  |  |  |  | -1.09 | -17.1 | -18.19 |  |  |  |  |  |
|  |  | No college degree | |  |  |  | Operculum Cortex (Left) |  |  |  | -5.82 | 0 | -5.82 |  |  |  |  |  |
|  |  | College degree | |  |  |  |  |  |  |  | -5.82 | 1.6 | -4.22 |  |  |  |  |  |
|  |  | No college degree | |  |  |  | Pole (Right) |  |  |  | -51.84 | 0 | -51.84 |  |  |  |  |  |
|  |  | College degree | |  |  |  |  |  |  |  | -51.84 | -19.91 | -71.75 |  |  |  |  |  |
|  |  | No college degree | |  |  |  | Superior Gyrus (Right) |  |  |  | 2.31 | 0 | 2.31 |  |  |  |  |  |
|  |  | College degree | |  |  |  |  |  |  |  | 2.31 | -29.43 | -27.12 |  |  |  |  |  |
|  |  | No college degree | |  |  |  | Medial Cortex (Right) |  |  |  | -1.82 | 0 | -1.82 |  |  |  |  |  |
|  |  | College degree | |  |  |  |  |  |  |  | -1.82 | -4.82 | -6.64 |  |  |  |  |  |
|  |  | No college degree | |  |  |  | Orbital Cortex (Right) |  |  |  | -5.54 | 0 | -5.54 |  |  |  |  |  |
|  |  | College degree | |  |  |  |  |  |  |  | -5.54 | -11.12 | -16.66 |  |  |  |  |  |
|  |  | No college degree | |  |  |  | Operculum Cortex ((Right) |  |  |  | -2.85 | 0 | -2.85 |  |  |  |  |  |
|  |  | College degree | |  |  |  |  |  |  |  | -2.85 | 2.35 | -0.5 |  |  |  |  |  |
|  |  | No college degree | |  |  |  | Pole (Left) | PM 10 |  |  | -27.45 | 0 | -27.45 |  |  |  |  |  |
|  |  | College degree | |  |  |  |  |  |  |  | -27.45 | 11.86 | -15.59 |  |  |  |  |  |
|  |  | No college degree | |  |  |  | Superior Gyrus (Left) |  |  |  | -19.28 | 0 | -19.28 |  |  |  |  |  |
|  |  | College degree | |  |  |  |  |  |  |  | -19.28 | 21.41 | 2.13 |  |  |  |  |  |
|  |  | No college degree | |  |  |  | Medial Cortex (Left) |  |  |  | -1.41 | 0 | -1.41 |  |  |  |  |  |
|  |  | College degree | |  |  |  |  |  |  |  | -1.41 | -0.49 | -1.9 |  |  |  |  |  |
|  |  | No college degree | |  |  |  | Orbital Cortex (Left) |  |  |  | -2.71 | 0 | -2.71 |  |  |  |  |  |
|  |  | College degree | |  |  |  |  |  |  |  | -2.71 | -2.12 | -4.83 |  |  |  |  |  |
|  |  | No college degree | |  |  |  | Operculum Cortex (Left) |  |  |  | -4.83 | 0 | -4.83 |  |  |  |  |  |
|  |  | College degree | |  |  |  |  |  |  |  | -4.83 | 3.1 | -1.73 |  |  |  |  |  |
|  |  | No college degree | |  |  |  | Pole (Right) |  |  |  | -40.6 | 0 | -40.6 |  |  |  |  |  |
|  |  | College degree | |  |  |  |  |  |  |  | -40.6 | 28.36 | -12.24 |  |  |  |  |  |
|  |  | No college degree | |  |  |  | Superior Gyrus (Right) |  |  |  | -4.07 | 0 | -4.07 |  |  |  |  |  |
|  |  | College degree | |  |  |  |  |  |  |  | -4.07 | 6.61 | 2.54 |  |  |  |  |  |
|  |  | No college degree | |  |  |  | Medial Cortex (Right) |  |  |  | -0.78 | 0 | -0.78 |  |  |  |  |  |
|  |  | College degree | |  |  |  |  |  |  |  | -0.78 | -1.17 | -1.95 |  |  |  |  |  |
|  |  | No college degree | |  |  |  | Orbital Cortex (Right) |  |  |  | -3.15 | 0 | -3.15 |  |  |  |  |  |
|  |  | College degree | |  |  |  |  |  |  |  | -3.15 | -1.65 | -4.8 |  |  |  |  |  |
|  |  | No college degree | |  |  |  | Operculum Cortex ((Right) |  |  |  | -1.62 | 0 | -1.62 |  |  | - | P<0.05 | NS |
|  |  | College degree | |  |  |  |  |  |  |  | -1.62 | 3.93 | 2.31 |  |  |  |  |  |
|  |  | No college degree | |  |  |  | Pole (Left) | NO_2_ |  |  | -14.57 | 0 | -14.57 |  |  | - | P<0.001 |  |
|  |  | College degree | |  |  |  |  |  |  |  | -14.57 | 17.25 | 2.68 |  |  |  |  |  |
|  |  | No college degree | |  |  |  | Superior Gyrus (Left) |  |  |  | -5.81 | 0 | -5.81 |  |  |  |  |  |
|  |  | College degree | |  |  |  |  |  |  |  | -5.81 | 13.01 | 7.2 |  |  |  |  |  |
|  |  | No college degree | |  |  |  | Medial Cortex (Left) |  |  |  | -0.19 | 0 | -0.19 |  |  | - | P>0.05 | NS |
|  |  | College degree | |  |  |  |  |  |  |  | -0.19 | 0.71 | 0.52 |  |  |  |  |  |
|  |  | No college degree | |  |  |  | Orbital Cortex (Left) |  |  |  | -2.49 | 0 | -2.49 |  |  | Qualitative | P<0.05 | S |
|  |  | College degree | |  |  |  |  |  |  |  | -2.49 | 3.37 | 0.88 |  |  |  |  |  |
|  |  | No college degree | |  |  |  | Operculum Cortex (Left) |  |  |  | -1.52 | 0 | -1.52 |  |  | Qualitative | P<0.001 |  |
|  |  | College degree | |  |  |  |  |  |  |  | -1.52 | 1.71 | 0.19 |  |  |  |  |  |
|  |  | No college degree | |  |  |  | Pole (Right) |  |  |  | -18.99 | 0 | -18.99 |  |  | Qualitative |  |  |
|  |  | College degree | |  |  |  |  |  |  |  | -18.99 | 23.26 | 4.27 |  |  |  |  |  |
|  |  | No college degree | |  |  |  | Superior Gyrus (Right) |  |  |  | -4.31 | 0 | -4.31 |  |  | Qualitative | P<0.01 |  |
|  |  | College degree | |  |  |  |  |  |  |  | -4.31 | 10.11 | 5.8 |  |  |  |  |  |
|  |  | No college degree | |  |  |  | Medial Cortex (Right) |  |  |  | -0.36 | 0 | -0.36 |  |  | - | P>0.05 | NS |
|  |  | College degree | |  |  |  |  |  |  |  | -0.36 | 0.9 | 0.54 |  |  |  |  |  |
|  |  | No college degree | |  |  |  | Orbital Cortex (Right) |  |  |  | -2.83 | 0 | -2.83 |  |  | Qualitative | P<0.05 | S |
|  |  | College degree | |  |  |  |  |  |  |  | -2.83 | 3.42 | 0.59 |  |  |  |  |  |
|  |  | No college degree | |  |  |  | Operculum Cortex ((Right) |  |  |  | -0.63 | 0 | -0.63 |  |  | Qualitative |  |  |
|  |  | College degree | |  |  |  |  |  |  |  | -0.63 | 1.24 | 0.61 |  |  |  |  |  |
|  |  | No college degree | |  |  |  | Pole (Left) | NO |  |  | -6.47 | 0 | -6.47 |  |  | Qualitative | P<0.01 |  |
|  |  | College degree | |  |  |  |  |  |  |  | -6.47 | 6.75 | 0.28 |  |  |  |  |  |
|  |  | No college degree | |  |  |  | Superior Gyrus (Left) |  |  |  | -3.19 | 0 | -3.19 |  |  | Qualitative | P<0.001 |  |
|  |  | College degree | |  |  |  |  |  |  |  | -3.19 | 5.8 | 2.61 |  |  |  |  |  |
|  |  | No college degree | |  |  |  | Medial Cortex (Left) |  |  |  | -0.12 | 0 | -0.12 |  |  | - | P>0.05 | NS |
|  |  | College degree | |  |  |  |  |  |  |  | -0.12 | 0.22 | 0.1 |  |  |  |  |  |
|  |  | No college degree | |  |  |  | Orbital Cortex (Left) |  |  |  | -1.51 | 0 | -1.51 |  |  | Positive | P<0.05 | S |
|  |  | College degree | |  |  |  |  |  |  |  | -1.51 | -1.53 | -3.04 |  |  |  |  |  |
|  |  | No college degree | |  |  |  | Operculum Cortex (Left) |  |  |  | -0.77 | 0 | -0.77 |  |  | Negative | P<0.01 |  |
|  |  | College degree | |  |  |  |  |  |  |  | -0.77 | 0.72 | -0.05 |  |  |  |  |  |
|  |  | No college degree | |  |  |  | Pole (Right) |  |  |  | -9.04 | 0 | -9.04 |  |  | Qualitative | P<0.001 |  |
|  |  | College degree | |  |  |  |  |  |  |  | -9.04 | 9.75 | 0.71 |  |  |  |  |  |
|  |  | No college degree | |  |  |  | Superior Gyrus (Right) |  |  |  | -1.81 | 0 | -1.81 |  |  | Qualitative | P<0.05 |  |
|  |  | College degree | |  |  |  |  |  |  |  | -1.81 | 3.62 | 1.81 |  |  |  |  |  |
|  |  | No college degree | |  |  |  | Medial Cortex (Right) |  |  |  | -0.22 | 0 | -0.22 |  |  | - | P>0.05 | NS |
|  |  | College degree | |  |  |  |  |  |  |  | -0.22 | 0.39 | 0.17 |  |  |  |  |  |
|  |  | No college degree | |  |  |  | Orbital Cortex (Right) |  |  |  | -1.6 | 0 | -1.6 |  |  | Negative | P<0.05 | S |
|  |  | College degree | |  |  |  |  |  |  |  | -1.6 | 1.41 | -0.19 |  |  |  |  |  |
|  |  | No college degree | |  |  |  | Operculum Cortex ((Right) |  |  |  | -0.43 | 0 | -0.43 |  |  | Qualitative | P<0.01 |  |
|  |  | College degree | |  |  |  |  |  |  |  | -0.43 | 0.69 | 0.26 |  |  |  |  |  |
| Chen et al., 2020 | | Years of education <=12 | | Cognitive assessment test | | MoCA-T* (binary) | Score < 24 | PM2.5 | 2^nd^ tertile | Odd ratio | - | - | 2.26 | 0.68 | 7.53 | - | 0.97 | NS |
|  |  | >12 | |  |  |  |  |  |  |  |  |  | 2.68 | 0.41 | 17.56 |  |  |  |
|  |  | <=12 | |  |  |  |  |  | 3^rd^ tertile |  |  |  | 3.53 | 0.99 | 12.6 |  | 0.61 |  |
|  |  | >12 | |  |  |  |  |  |  |  |  |  | 8.1 | 1.26 | 52.19 |  |  |  |
|  |  | <=12 | |  |  | MoCA-T* (continuous) | Continuous score | PM2.5 | 2^nd^ tertile | Coefficient |  |  | 0.01 | -0.76 | 0.78 |  | 0.2 |  |
|  |  | >12 | |  |  |  |  |  |  |  |  |  | -0.36 | -0.98 | 0.26 |  |  |  |
|  |  | <=12 | |  |  |  |  |  | 3^rd^ tertile |  |  |  | -0.47 | -1.25 | 0.31 |  | 0.26 |  |
|  |  | >12 | |  |  |  |  |  |  |  |  |  | -0.72 | -1.38 | -0.05 |  |  |  |
|  |  | <=12 | |  |  | Trail Making Test A |  | PM10 | 2^nd^ tertile |  |  |  | -0.21 | -0.47 | 0.05 |  | 0.28 |  |
|  |  | >12 | |  |  |  |  |  |  |  |  |  | -0.06 | -0.22 | 0.11 |  |  |  |
|  |  | <=12 | |  |  |  |  |  | 3^rd^ tertile |  |  |  | -0.34 | -0.63 | -0.05 |  | 0.44 |  |
|  |  | >12 | |  |  |  |  |  |  |  |  |  | -0.19 | -0.42 | 0.04 |  |  |  |
|  |  | <=12 | |  |  | Verbal Fluency |  | PMcoarse | 2^nd^ tertile |  |  |  | -0.09 | -0.33 | 0.14 |  | 0.58 |  |
|  |  | >12 | |  |  |  |  |  |  |  |  |  | -0.23 | -0.44 | -0.02 |  |  |  |
|  |  | <=12 | |  |  |  |  |  | 3^rd^ tertile |  |  |  | -0.15 | -0.44 | 0.13 |  | 0.67 |  |
|  |  | >12 | |  |  |  |  |  |  |  |  |  | -0.28 | -0.5 | -0.06 |  |  |  |
|  |  | <=12 | |  |  |  |  | NO_2_ | 2^nd^ tertile |  |  |  | 0.1 | -0.09 | 0.29 |  | 0.47 |  |
|  |  | >12 | |  |  |  |  |  |  |  |  |  | 0.15 | 0.002 | 0.3 |  |  |  |
|  |  | <=12 | |  |  |  |  |  | 3^rd^ tertile |  |  |  | 0.04 | -0.24 | 0.31 |  | 0.61 |  |
|  |  | >12 | |  |  |  |  |  |  |  |  |  | -0.01 | -0.23 | 0.2 |  |  |  |
| Wang et al., 2020 | | Illiterate | | Cognitive assessment test | | MMSE* | Score ≥ 18 | PM 2.5 | NP | Hazard ratio | - | - | 1.056 | 1.022 | 1.091 | - | 0.79 | NS |
|  |  | Literate | |  |  |  |  |  |  |  |  |  | 1.043 | 0.995 | 1.094 |  |  |  |
| Yao et al., 2021 | | Years of education: none | | Cognitive assessment test | | Chinese MMSE* | Score ≥ 25 | Distance to the main roadway | <=100m vs. >100m | Odds ratio | - | - | 1.18 | 1.04 | 1.35 | - | 0.72 | NS |
|  |  | ≥1-6 years | |  |  |  |  |  |  |  |  |  | 1.24 | 1.05 | 1.47 |  |  |  |
| Alishire et al., 2021 | | 9-11 years | | Telephone interview for cognitive status (TICS) | | | Score ≥ 11 | PM 2.5 | Per IQR (5.0 ug/m^3^) increase | Coefficient | - | - | 0.796 | -0.31 | 1.90 | - | NP | NS |
|  |  | 12 years | |  |  |  |  |  |  |  |  |  | 0.182 | -0.73 | 1.09 |  |  |  |
|  |  | 13-18 years | |  |  |  |  |  |  |  |  |  | 0.233 | -0.66 | 1.13 |  |  |  |
| Mortamais et al., 2021 | | Primary (≤5 years) | | 3-step procedure | | All cause dementia, AD, Vascular/mixed dementia (VaD) | Diagnosis of all cause dementia, AD, VaD | PM2.5 | Per 5 μg/m3 increase | Hazard Ratio | - | - | NP | | | NP (not modified by education) | NP | NS |
|  |  | Lower secondary  (5–9 years) | |  |  |  |  |  |  |  | - | - |  |  |  |  |  |  |
|  |  | Higher secondary  (>9 years) | |  |  |  |  |  |  |  | - | - |  |  |  |  |  |  |
| Shaffer et al., 2021 | | None/GED/High school/other | | Dementia assessment test | | CASI* | Score below 86 | PM 2.5 | Per IQR (1.0 ug/m^3^) increase | Hazard Ratio | - | - | 1.17 | 1.03 | 1.33 | - | 0.86 | NS |
|  |  | College/Master’s/PhD | |  |  |  |  |  |  |  |  |  | 1.16 | 1.03 | 1.31 |  |  |  |
| Yang et al., 2022 | | Illiteracy | | Combination of MMSE*, MoCA*, and MRI | | | Diagnosis of Alzheimer’s disease | PM 2.5 | Per 10 ug/m^3^ increase | Hazard Ratio | - | - | 1.13 | 1.09 | 1.2 | - | NP | NP |
|  |  | Primary School | |  |  |  |  |  |  |  |  |  | 1.1 | 0.99 | 1.15 |  |  |  |
|  |  | Junior high school | |  |  |  |  |  |  |  |  |  | 0.98 | 0.77 | 1.24 |  |  |  |
|  |  | Senior high school and above | |  |  |  |  |  |  |  |  |  | 0.86 | 0.26 | 2.81 |  |  |  |
| Wang et al., 2022 | | Less than high school or GED | | Telepgone interview for cognitive status (TICSm) | | | Diagnosis of incident dementia | PM 2.5 | Per IQR (1.78 ug/m^3^) increase | Hazard ratio | - | - | 0.9 | 0.7 | 1.14 | - | 0.6 | NS |
|  |  | High school but less than College | |  |  |  |  |  |  |  |  |  | 0.78 | 0.66 | 0.93 |  |  |  |
|  |  | College or more | |  |  |  |  |  |  |  |  |  | 0.78 | 0.63 | 0.95 |  |  |  |
|  |  | Less than high school or GED | |  |  |  |  | NO_2_ | Per IQR (3.91 ppb) increase |  |  |  | 0.74 | 0.58 | 0.95 | - | 0.27 |  |
|  |  | High school but less than College | |  |  |  |  |  |  |  |  |  | 0.75 | 0.63 | 0.9 |  |  |  |
|  |  | College or more | |  |  |  |  |  |  |  |  |  | 0.91 | 0.75 | 1.12 |  |  |  |
| Hu et al., 2022 | | College or more | | Cognitive assessment test | episodic memory, orientation and attention, and visuospatial ability | | A positive difference (Δ cognitive function >0) between the  2011 and 2015 cognitive function assessments; | PM 1 | Per IQR increase | Odd ratio | - | - | 0.77 | 0.74 | 0.81 | Positive | 0.029 | S |
|  |  | Middle school or lower | |  |  |  |  |  |  |  |  |  | 0.81 | 0.75 | 0.88 |  |  |  |
|  |  | College or more | |  |  |  |  | PM 2.5 |  |  |  |  | 0.74 | 0.71 | 0.78 |  | 0.852 | NS |
|  |  | Middle school or lower | |  |  |  |  |  |  |  |  |  | 0.75 | 0.69 | 0.81 |  |  |  |
|  |  | College or more | |  |  |  |  | PM10 |  |  |  |  | 0.83 | 0.8 | 0.88 |  | 0.227 |  |
|  |  | Middle school or lower | |  |  |  |  |  |  |  |  |  | 0.8 | 0.76 | 0.86 |  |  |  |
|  |  | College or more | |  |  |  |  | NO_2_ |  |  |  |  | 0.84 | 0.8 | 0.88 |  | 0.766 |  |
|  |  | Middle school or lower | |  |  |  |  |  |  |  |  |  | 0.86 | 0.8 | 0.94 |  |  |  |
|  |  | College or more | |  |  |  |  | NO |  |  |  |  | 1.02 | 0.98 | 1.07 |  | 0.734 |  |
|  |  | Middle school or lower | |  |  |  |  |  |  |  |  |  | 1.01 | 0.93 | 1.1 |  |  |  |
| Gao et al., 2022 | | Literate | | Cognitive assessment test | Chinese MMSE* | | Score ≥ 18 + declined ≥ 4 point | O_3_ | Per 10.4 ug/m^3^ increase | Hazard ratio | - | - | 1.119 | 1.018 | 1.231 | - | 0.733 | NS |
|  |  | Illiterate | |  |  |  |  |  |  |  |  |  | 1.098 | 1.026 | 1.175 |  |  |  |
|  | \| * AD: Alzheimer's Disease; CCFM: Chicago Cognitive Function Measure; MRI: Magnetic Resonance Imaging; MMSE: Mini-Mental State Examination; WM: White Matter; IADL: Impaired Activities of Daily Living; HbA1c: Glycosylated hemoglobin; CRP: C-reactive Protein; CVD: Cardiovascular Disease; SEVLT: Spanish English Verbal Learning Test; DSMMD: Diagnostic and Statistical Manual of Mental Disorders; 3WMT: Three-word memory test; SVF: Semantic Verbal fluency; MCI: Mild Cognitive Function; HV: Hippocampal Volumes; DADL: Disability in activities of daily living; HFE: Hemochromatosis gene; VaD: Vascular Alzheimer’s Disease; NINCDS-ADRDA: National Institute of Neurological and Communicative Diseases and Stroke/Alzheimer’s Disease and Related Disorders Association; NINDS-AIREN: National Institute of Neurological Disorders and Stroke and the Association Internationale pour la Recherche et I’Enseignement en Neurosicenes; CERAD: The Consortium to Establish a Registry for Alzheimer’s Disease; SNPs: Single Nucleotide Polymorphism \| \| --- \| \| ** CI: Confidence Interval; S/NS: Significance/Non-Significance; IQR: Inter-Quartile Range; RD: Residential Distance; NP: Not provided \| \| *** The total effect size (A+C) was manually calculated unless the effect of air pollution exposure on each category of the effect modifier was provided in the studies \| \| **** 95% CI is based on coefficient of effect modifiers  ※ 6 studies tested education as an effect modifier but either 1) did not provide effect size measures in the tables, but tested and mentioned non significance (Alishire and Crimmins, 2014; Shin et al., 2019; Mortamais et al., 2021; Cerza et al., 2019) or 2) only provided graphics without the measures (Tzivian, Dlugaj, Winkler, Weinmayr, et al., 2016; Tzivian, Dlugaj, Winkler, Hennig, et al., 2016). \| | | | | | | | | | | | | | | | | | |

- Table E4: Effect size by potential effect modifier – Socioeconomic Status (SES) (7/14)

| **Study** | | **Effect Modifier** | | **Outcome** | | | **Air**  **Pollutant** | **Exposure**  **Level** | **Types of Effect** | **Effect**  **Size**  **(A: Main Effect by exposure)** | **Effect Size**  **(C: Effect by interaction term)** | **Effect Size  Estimate*****  **(A+C)** | **95% CI*s** | | **Direction of Modifier Measure** | **p-value  for  interaction terms** | **Statistical Significance (S/NS**)** |
| --- | --- | --- | --- | --- | --- | --- | --- | --- | --- | --- | --- | --- | --- | --- | --- | --- | --- |
|  |  | **SES** | | **Outcome Measure Method** | | **Outcome Variable** |  |  |  |  |  |  | **lower** | **upper** |  |  |  |
| Sun et al., 2008*** | | GDP per capita | Low | Cognitive assessment test | MMSE* | Cognitive score ** | AP* index† | Per 1-unit increase | Coefficient | -1.51 | 0 | -1.51 | NP** | | Qualitative | NP | - |
|  |  |  | Medium |  |  |  |  |  |  | -1.51 | 1.84 | 0.33‡ |  |  |  | <0.001 | S |
|  |  |  | High |  |  |  |  |  |  | -1.51 | 2.67 | 1.61‡ |  |  |  | <0.001 | S |
| Ailshire and Crimmins, 2014 | | Household income | In dollars | Cognitive assessment test | Global Cognitive Score | Continuous score | PM2.5 | Per quartiles | Coefficient | - | - | NP | NP | NP | - | - | NS |
| Carey et al., 2018 | | Deprivation index | 1 (least deprived)/2/3/4/5(most deprived) | Health administrative data  (coded with ICD-10) | | Diagnosis of incident dementia | NO_2_ | Per IQR (7.5 μg/m^3^)  increase | Hazard Ratio | - | - | NP | NP | NP | - | - | NS |
| Cerza et al., 2019§ | | SES status | Very low | Dementia | Hospital admission records (ICD9-CM:  290.0, 290.2, 290.3) | Incident senile dementia | O3 | Per 10 μg/m3 | Hazard Ratio | - | - | 1.27 | 1.18 | 1.36 | Negative | 0.047 | S |
|  |  |  | Very high |  |  |  |  |  |  |  |  | 1.08 | 0.98 | 1.18 |  |  |  |
| Shin et al., 2019 | | - | - | Cognitive assessment test | Korean MMSE | Cognitive score | PM2.5 | Per IQR increase | Coefficients | - | - | NP | NP | NP | - | - | NS |
| Shi et al., 2020 | | Medicaid Eligibility | Non-dual | Alzheimer’s disease and related dementia | Hospital admission code (ICD-9: 331·0, 290; ICD-10: G30·9,  and F05) | Diagnosis of Alzheimer’s disease and related dementia | PM2.5 | Per 5 μg/m3 increase | Hazard Ratio | - | - | NP | NP | NP | - | - | NS |
|  |  |  | Dual |  |  |  |  |  |  |  |  |  |  |  |  |  |  |
| Wang et al., 2020 | | GDP | Low-tertile | Cognitive assessment test | Chinese MMSE | Poor cognitive function | PM2.5 | Per 10 μg/m3 | Hazard Ratio | - | - | 1.081 | 1.026 | 1.138 | - | 0.16 | NS |
|  |  |  | Middle-tertile |  |  |  |  |  |  |  |  | 1.052 | 1.003 | 1.103 |  |  |  |
|  |  |  | Upper-tertile |  |  |  |  |  |  |  |  | 1.025 | 0.983 | 1.068 |  |  |  |
| Christensen et al., 2022◊ | | % of rented homes | | Cognitive assessment test | CFI* | CFI score** | CO | NP | Coefficient | - | 0.018 | NP | | |  | ≥0.05 | NS |
|  |  |  |  |  |  |  | EC |  |  |  | **0.032** |  |  |  | NP | <0.05 | S |
|  |  |  |  |  |  |  | NH_4_ |  |  |  | 0.004 |  |  |  |  | ≥0.05 | NS |
|  |  |  |  |  |  |  | NO_2_ |  |  |  | 0.015 |  |  |  |  | ≥0.05 | NS |
|  |  |  |  |  |  |  | NO_3_ |  |  |  | -0.003 |  |  |  |  | ≥0.05 | NS |
|  |  |  |  |  |  |  | NO_x_ |  |  |  | 0.022 |  |  |  |  | ≥0.05 | NS |
|  |  |  |  |  |  |  | OC |  |  |  | 0.004 |  |  |  |  | ≥0.05 | NS |
|  |  |  |  |  |  |  | O_3_ |  |  |  | -0.007 |  |  |  |  | ≥0.05 | NS |
|  |  |  |  |  |  |  | PM_10_ |  |  |  | -0.014 |  |  |  |  | ≥0.05 | NS |
|  |  |  |  |  |  |  | PM_2.5_ |  |  |  | 0.008 |  |  |  |  | ≥0.05 | NS |
|  |  |  |  |  |  |  | SO_2_ |  |  |  | 0.005 |  |  |  |  | ≥0.05 | NS |
|  |  |  |  |  |  |  | SO_4_ |  |  |  | 0.022 |  |  |  |  | ≥0.05 | NS |
|  |  | Negative median home value | |  |  |  | CO |  |  |  | -0.022 |  |  |  |  | ≥0.05 | NS |
|  |  |  |  |  |  |  | EC |  |  |  | -0.009 |  |  |  |  | ≥0.05 | NS |
|  |  |  |  |  |  |  | NH_4_ |  |  |  | -0.004 |  |  |  |  | ≥0.05 | NS |
|  |  |  |  |  |  |  | NO_2_ |  |  |  | -0.024 |  |  |  |  | ≥0.05 | NS |
|  |  |  |  |  |  |  | NO_3_ |  |  |  | -0.016 |  |  |  |  | ≥0.05 | NS |
|  |  |  |  |  |  |  | NO_x_ |  |  |  | -0.018 |  |  |  |  | ≥0.05 | NS |
|  |  |  |  |  |  |  | OC |  |  |  | 0.006 |  |  |  |  | ≥0.05 | NS |
|  |  |  |  |  |  |  | O_3_ |  |  |  | -0.007 |  |  |  |  | ≥0.05 | NS |
|  |  |  |  |  |  |  | PM_10_ |  |  |  | **0.070** |  |  |  | NP | <0.05 | S |
|  |  |  |  |  |  |  | PM_2.5_ |  |  |  | 0.007 |  |  |  |  | ≥0.05 | NS |
|  |  |  |  |  |  |  | SO_2_ |  |  |  | 0.005 |  |  |  |  | ≥0.05 | NS |
|  |  |  |  |  |  |  | SO_4_ |  |  |  | 0.015 |  |  |  |  | ≥0.05 | NS |
| Shi et al., 2021 | | Medicaid Eligibility | No | Medicare record data | Dementia | Diagnosis of dementia | PM2.5 | Per IQR increase | Hazard Ratio | - | - | NP | | | Positive | Reference | S |
|  |  |  | Yes |  |  |  |  |  |  |  |  |  |  |  |  | <0.001 |  |
|  |  |  | No |  |  |  | NO2 |  |  |  |  |  |  |  | Positive | Reference | S |
|  |  |  | Yes |  |  |  |  |  |  |  |  |  |  |  |  | <0.001 |  |
|  |  |  | No |  |  |  | O3 |  |  |  |  |  |  |  | Positive | Reference | S |
|  |  |  | Yes |  |  |  |  |  |  |  |  |  |  |  |  | <0.001 |  |
|  |  |  | No |  | AD | Diagnosis of AD | PM2.5 |  |  |  |  |  |  |  | Positive | Reference | S |
|  |  |  | Yes |  |  |  |  |  |  |  |  |  |  |  |  | <0.001 |  |
|  |  |  | No |  |  |  | NO2 |  |  |  |  |  |  |  |  | Reference | NS |
|  |  |  | Yes |  |  |  |  |  |  |  |  |  |  |  |  | 0.006 |  |
|  |  |  | No |  |  |  | O3 |  |  |  |  |  |  |  | Positive | Reference | S |
|  |  |  | Yes |  |  |  |  |  |  |  |  |  |  |  |  | 0.03 |  |
| Mortamais et al., 2021 | | Deprivation index | continuous | 3-step procedure | All cause dementia, AD, Vascular/mixed dementia (VaD) | Diagnosis of all cause dementia, AD, VaD | PM2.5 | Per 5 μg/m3 increase | Hazard Ratio | - | - | NP | NP | NP | NP (not modified by deprivation index) | - | S |
| Yao et al.,2021 | | Family income | < 30000 (Yuan)  ≥ 30000 (Yuan) | Cognitive assessment test | Chinese MMSE | Cognitive impairment | RD** to main roadway  ( ≤100m vs. >100m) | NP | Odds Ratio | - | - | 1.22 | 1.04 | 1.43 |  | 0.82 | NS |
|  |  |  |  |  |  |  |  |  |  |  |  | 1.2 | 1.05 | 1.37 | - |  |  |
| Z. Li et al., 2022 | | Neighborhood SES Clusters | 1 | Cognitive assessment test | CFI | Percent change (%) of CFI | CO | Per IQR (=328.4 ppb) increase | Coefficient | - | - | 3.5 | -1.9 | 9.2 |  | 0.0002 | S |
|  |  |  | 2 |  |  |  |  |  |  |  |  | -4.7 | -8.5 | -0.7 |  |  |  |
|  |  |  | 3 |  |  |  |  |  |  |  |  | -1.4 | -4 | 1.4 |  |  |  |
|  |  |  | 4 |  |  |  |  |  |  |  |  | 6 | 1.7 | 10.4 | Not applicable |  |  |
|  |  |  | 5 |  |  |  |  |  |  |  |  | -4.4 | -9.4 | 1 |  |  |  |
|  |  |  | 6 |  |  |  |  |  |  |  |  | 5.4 | -0.2 | 11.3 |  |  |  |
|  |  |  | 7 |  |  |  |  |  |  |  |  | 13 | 0.9 | 26.5 |  |  |  |
|  |  |  | 1 |  |  |  | NOx | Per IQR (=28.0 ppb) increase |  |  |  | 3.2 | -2.1 | 8.7 |  | 0.0002 | S |
|  |  |  | 2 |  |  |  |  |  |  |  |  | -4.2 | -7.8 | -0.5 |  |  |  |
|  |  |  | 3 |  |  |  |  |  |  |  |  | -1.1 | -3.7 | 1.6 |  |  |  |
|  |  |  | 4 |  |  |  |  |  |  |  |  | 6.8 | 2.5 | 11.3 | Not applicable |  |  |
|  |  |  | 5 |  |  |  |  |  |  |  |  | -4.4 | -0.98 | 1.3 |  |  |  |
|  |  |  | 6 |  |  |  |  |  |  |  |  | 4.9 | -0.4 | 10.4 |  |  |  |
|  |  |  | 7 |  |  |  |  |  |  |  |  | 13 | -0.1 | 27.8 |  |  |  |
|  |  |  | 1 |  |  |  | PM2.5 | Per IQR (=1.3 μg/m3) increase |  |  |  | 2.5 | -4.1 | 9.5 |  | <0.0001 | S |
|  |  |  | 2 |  |  |  |  |  |  |  |  | -6.5 | -11.5 | -1.1 |  |  |  |
|  |  |  | 3 |  |  |  |  |  |  |  |  | -1.5 | -5.4 | 2.4 |  |  |  |
|  |  |  | 4 |  |  |  |  |  |  |  |  | 6.8 | 1.8 | 12.1 |  |  |  |
|  |  |  | 5 |  |  |  |  |  |  |  |  | -5.1 | -10.3 | 0.3 | Not applicable |  |  |
|  |  |  | 6 |  |  |  |  |  |  |  |  | 9.8 | 2.2 | 18 |  |  |  |
|  |  |  | 7 |  |  |  |  |  |  |  |  | 17.3 | 2.5 | 34.2 |  |  |  |
| Hu et al., 2023 | | - | - | - | - | - | - | - | - | - | - | - | - | - | - | - | NS |
| Shi et al., 2023 | | Medicaid Eligibility | No | Medicare record data | Dementia | Diagnosis of dementia | Soil dust (DUST) | Per IQR increase | Hazard Ratio | - | - | - | - | - | Positive | NP | S |
|  |  |  | Yes |  |  |  |  |  |  |  |  |  |  |  |  |  |  |
|  |  |  | No |  |  |  | Sulfate |  |  |  |  |  |  |  |  |  |  |
|  |  |  | Yes |  |  |  |  |  |  |  |  |  |  |  |  |  |  |
|  |  |  | No |  |  |  | Ammonium |  |  |  |  |  |  |  |  |  |  |
|  |  |  | Yes |  |  |  |  |  |  |  |  |  |  |  |  |  |  |
|  | \| *MMSE: Mini-Mental State Examination; AP: Air pollution; CFI: Cognitive function instrument  ** Higher scores suggest worse cognitive impairment; ; S/NS: Significance/Non-Significance; NP: Not provided  *** including ICD-9: 331·0, 290; ICD-10: G30·9, and F05  † AP index includes 3 types of pollutants: SO2, NO2, and inhalable particulates (consisting of PM10, CO, O3) \| \| --- \|   ‡ People living in urban areas with a medium GDP had worse AP effect than a low GDP group; and even greater worse effect of AP with high GDP.  ※ 8 studies reported test results of effect modification by SES but provide all/or parts of effect size estimates but rather provided 1) graphics with overlapping 95% Cis (Carey et al., 2018; Shi et al., 2020; Shi et al., 2021; Shi et al., 2023; Shin et al., 2019; Ailshire and Crimmins, 2014; Hu et al., 2023) with effect sizes with information about statistical significance indicated in graphics or 2) in-text (Ailshire and Crimmins 2014) only without providing information about effect sizes.  Mortamais 2021, Carey 2018: not significant  § Information about effect sizes only available in text.  ◊ Only effect sizes of interaction term with its 95% CI (not listed in our table) and significancy at 0.05 level (which we used for judgement of the significance in our table).  Significant studies: Shin et al., 2019; Shi et al., 2023 (Medicaid eligibility; significance of the modifiers in the table above is based on in-text reporting of the original study); Shi et al., 2021(Medicaid eligibility) | | | | | | | | | | | | | | | | |

- Table E5: Effect size by potential effect modifier – Race/ethnicity (Significant studies: 3/5)

| **Study** | | **Effect Modifier** | **Outcome** | | | **Air**  **Pollutant** | **Exposure**  **Level** | **Types of Effect** | **Effect**  **Size**  **(A: Main Effect by exposure)** | **Effect Size**  **(C: Effect by interaction term)** | **Effect Size  Estimate**  **(A+C)** | **95% CI**s** | | **Direction of Modifier Measure** | **p-value  for  interaction terms** | **Statistical Significance (S/NS**)** |
| --- | --- | --- | --- | --- | --- | --- | --- | --- | --- | --- | --- | --- | --- | --- | --- | --- |
|  |  | **Race/Ethnicity** | **Outcome Measure Method** | | **Outcome Variable** |  |  |  |  |  |  | **lower** | **upper** |  |  |  |
| Ailshire & Crimmins, 2014 | | Non-Hispanic White | Cognitive assessment test | Global Cognitive Score | Continuous score | PM2.5 | Per quartiles | Coefficient | - | - | NP** | NP | NP | - | NP | NS |
|  |  | Non-Hispanic Black |  |  |  |  |  |  |  |  |  |  |  |  |  |  |
|  |  | Hispanic |  |  |  |  |  |  |  |  |  |  |  |  |  |  |
|  |  | Other |  |  |  |  |  |  |  |  |  |  |  |  |  |  |
| Kulick et al., 2020 | | White non-Hispanic | Cognitive assessment test | Test batteries from WHICAP* | Change in the test score | NO_2_ | Per IQR** (11.2 ppb) increase | Coefficient | - | - | -0.144 | -0.19 | -0.09 | - | NP | NS |
|  |  | Black non-Hispanic |  |  |  |  |  |  |  |  | -0.11 | -0.16 | -0.06 |  |  |  |
|  |  | Hispanic |  |  |  |  |  |  |  |  | -0.045 | -0.08 | -0.01 |  |  |  |
|  |  | White non-Hispanic |  |  |  | PM 2.5 | Per IQR (4.42 ug/m^3^) increase |  |  |  | -0.17 | -0.23 | -0.11 |  |  |  |
|  |  | Black non-Hispanic |  |  |  |  |  |  |  |  | -0.136 | -0.2 | -0.08 |  |  |  |
|  |  | Hispanic |  |  |  |  |  |  |  |  | -0.05 | -0.09 | -0.01 |  |  |  |
|  |  | White non-Hispanic |  |  |  | PM 10 | Per IQR (7.95 ug/m^3^) increase |  |  |  | -0.087 | -0.13 | -0.05 |  |  |  |
|  |  | Black non-Hispanic |  |  |  |  |  |  |  |  | -0.058 | -0.09 | -0.02 |  |  |  |
|  |  | Hispanic |  |  |  |  |  |  |  |  | -0.029 | -0.05 | -0.01 |  |  |  |
| Shi et al., 2020 | | White | Alzheimer’s disease and related dementia | Hospital admission code (ICD-9: 331·0, 290; ICD-10: G30·9,  and F05) | Diagnosis of Alzheimer’s disease and related dementia | PM2.5 | Per 5 μg/m3 increase | Hazard Ratio | - | - | NP | | | Qualitative | NP | S |
|  |  | Black |  |  |  |  |  |  |  |  |  |  |  |  |  |  |
|  |  | Other |  |  |  |  |  |  |  |  |  |  |  |  |  |  |
| Shi et al., 2021 | | White | Medicare record data | Dementia | Diagnosis of dementia | PM2.5 | Per IQR increase | Hazard Ratio | - | |  |  |  | Positive in Black group | Reference | S |
|  |  | Black |  |  |  |  |  |  |  |  |  |  |  |  | <0.001 |  |
|  |  | Other |  |  |  |  |  |  |  |  |  |  |  |  | 0.57 |  |
|  |  | White |  |  |  | NO2 |  |  |  |  |  |  |  | Positive in Black group | Reference | S |
|  |  | Black |  |  |  |  |  |  |  |  |  |  |  |  | <0.001 |  |
|  |  | Other |  |  |  |  |  |  |  |  |  |  |  |  | 0.12 |  |
|  |  | White |  |  |  | O3 |  |  |  |  |  |  |  | Positive in Black group | Reference | S |
|  |  | Black |  |  |  |  |  |  |  |  |  |  |  |  | 0.04 |  |
|  |  | Other |  |  |  |  |  |  |  |  |  |  |  |  | 0.38 |  |
|  |  | White |  | AD | Diagnosis of AD | PM2.5 |  |  |  |  |  |  |  | - | Reference | NS |
|  |  | Black |  |  |  |  |  |  |  |  |  |  |  |  | 0.50 |  |
|  |  | Other |  |  |  |  |  |  |  |  |  |  |  |  | 0.73 |  |
|  |  | White |  |  |  | NO2 |  |  |  |  |  |  |  | Positive in Black group | Reference | S |
|  |  | Black |  |  |  |  |  |  |  |  |  |  |  |  | <0.001 |  |
|  |  | Other |  |  |  |  |  |  |  |  |  |  |  |  | 0.40 |  |
|  |  | White |  |  |  | O3 |  |  |  |  |  |  |  | Negative in Other group | Reference | S |
|  |  | Black |  |  |  |  |  |  |  |  |  |  |  |  | 0.46 |  |
|  |  | Other |  |  |  |  |  |  |  |  |  |  |  |  | <0.001 |  |
| Shi et al., 2023 | | White | Medicare record data | Dementia | Diagnosis of dementia | Nitrate | Per IQR increase | Hazard Ratio | - | | NP | | | Positive in Black group | NP | S |
|  |  | Black |  |  |  |  |  |  |  |  |  |  |  |  |  |  |
|  |  | Other |  |  |  |  |  |  |  |  |  |  |  |  |  |  |
|  |  | White |  |  |  | Sulfate |  |  |  |  |  |  |  | Positive in Black group |  | S |
|  |  | Black |  |  |  |  |  |  |  |  |  |  |  |  |  |  |
|  |  | Other |  |  |  |  |  |  |  |  |  |  |  |  |  |  |
|  |  | White |  |  |  | Ammonium |  |  |  |  |  |  |  | Positive in Other group |  | S |
|  |  | Black |  |  |  |  |  |  |  |  |  |  |  |  |  |  |
|  |  | Other |  |  |  |  |  |  |  |  |  |  |  |  |  |  |
|  |  | White |  |  |  | Soil dust (DUST) |  |  |  |  |  |  |  | Positive in Other group |  | S |
|  |  | Black |  |  |  |  |  |  |  |  |  |  |  |  |  |  |
|  |  | Other |  |  |  |  |  |  |  |  |  |  |  |  |  |  |
|  | \| * WHICAP: Washington Heights-Inwood Community Aging Project;  ** CI: Confidence Interval; S/NS: Significance/Non-Significance; IQR: Inter-Quartile Range; NP: Not provided \| \| --- \| \| ※ 4 studies tested BMI as an effect modifier but did not provide effect size measures in the tables, but tested and reported 1) non significance (Alishire & Crimmins, 2014; Shi et al., 2021; Shi et al., 2023) or 2) significance (Shi et al., 2020)  Significant study: Shi et al., 2020 (reported significant modifying effect by race/ethnicity factor (White, Black, and other subgroups) in PM2.5-ADRD association), Shi et al., 2021, and Shi et al., 2023 (significance of the modifiers in the table above is based on in-text reporting of the original study) \| | | | | | | | | | | | | | | | |

- Table E6: Effect size by potential effect modifier – Geographic location (6/15)

| **Study** | | **Effect Modifier** | | **Outcome** | | | | **Air**  **Pollutant** | **Exposure**  **Level** | **Types of Effect** | **Effect**  **Size**  **(A: Main Effect by exposure)** | **Effect Size**  **(C: Effect by interaction term)** | **Effect Size  Estimate**  **(A+C)** | **95% CI*s** | | **Direction of Modifier Measure** | **p-value  for  interaction terms** | **Statistical Significance (S/NS**)** |
| --- | --- | --- | --- | --- | --- | --- | --- | --- | --- | --- | --- | --- | --- | --- | --- | --- | --- | --- |
|  |  | **Geographic Location** | | **Outcome Measure Method** | | | **Outcome Variable** |  |  |  |  |  |  | **lower** | **upper** |  |  |  |
| Ranft et al., 2009 | | - | - | Cognitive assessment test | CERAD plus*/ Stroop/Sniffing | | Cognitive impairment | Distance from traffic | NP | Coefficients | - | - | NP** | NP | NP | - | NP | NS |
| Loop et al., 2013 | | Urbanicity – Model 1† | Rural | SIS cognitive assessment test | Mild cognitive impairment | | Incident cognitive decline | PM2.5 | per 10 μg/m3 | Odds Ratio | - | - | 0.68 | 0.23 | 2.08 |  | NP | NS |
|  |  |  | Mixed |  |  |  |  |  |  |  |  |  | 0.41 | 0.14 | 1.16 |  |  |  |
|  |  |  | Urban |  |  |  |  |  |  |  |  |  | 1.40 | 1.06 | 1.85 |  |  |  |
|  |  | Urbanicity – Model 2† | Rural |  |  |  |  |  |  |  |  |  | 0.61 | 0.19 | 1.92 |  |  | NS |
|  |  |  | Mixed |  |  |  |  |  |  |  |  |  | 0.33 | 0.11 | 0.99 |  |  |  |
|  |  |  | Urban |  |  |  |  |  |  |  |  |  | 1.13 | 0.83 | 1.54 |  |  |  |
|  |  | Urbanicity – Model 3† | Rural |  |  |  |  |  |  |  |  |  | 0.60 | 0.19 | 1.93 |  |  | NS |
|  |  |  | Mixed |  |  |  |  |  |  |  |  |  | 0.32 | 0.11 | 0.98 |  |  |  |
|  |  |  | Urban |  |  |  |  |  |  |  |  |  | 1.07 | 0.78 | 1.47 |  |  |  |
|  |  | Urbanicity – Model 4† | Rural |  |  |  |  |  |  |  |  |  | 0.79 | 0.23 | 2.68 |  |  | NS |
|  |  |  | Mixed |  |  |  |  |  |  |  |  |  | 0.34 | 0.11 | 1.04 |  |  |  |
|  |  |  | Urban |  |  |  |  |  |  |  |  |  | 1.06 | 0.77 | 1.48 |  |  |  |
| Carey et al., 2018 | | Urbanicty | Inner London/outer London practice | Health administrative data  (coded with ICD-10) | | | Diagnosis of incident dementia | NO_2_ | Per IQR (7.5 μg/m^3^)  increase | Hazard Ratio | - | - | - | - | - | - | NP | NS |
| Lee et al., 2019‡ | | Level of urbanization | Rural | Medicare enrollment and fee-for-service claims for hospitalization from the ResDAC | Dementia | | Incident dementia | PM2.5 | per 1 μg/m3 | Hazard Ratio | - | - | 1.036 | 1.031 | 1.041 |  | NP | S |
|  |  |  | Micropolitan |  |  |  |  |  |  |  |  |  | NP | NP | NP |  |  |  |
|  |  |  | Metropolitan |  |  |  |  |  |  |  |  |  | 1.052 | 1.05 | 1.054 | Positive |  |  |
| Shin et al., 2019 | | Residence | Rural | Cognitive assessment test (Digit span test, frontal assessment battery (FAB) | | Korean MMSE | Cognitive score | PM2.5 | Per IQR increase | Coefficients | - | - | - | - | - |  | NP | S |
|  |  |  | Urban |  |  |  |  |  |  |  |  |  |  |  |  | Negative |  |  |
| Shi et al., 2020 | | Quartiles of population density | Density Q1 | Alzheimer’s disease and related dementia | | Hospital admission code (ICD-9: 331·0, 290; ICD-10: G30·9,  and F05) | Diagnosis of Alzheimer’s disease and related dementia | PM2.5 | Per 5 μg/m3 increase | Hazard Ratio | - | - | - | - | - | Qualitative | NP | S |
|  |  |  | Density Q2 |  |  |  |  |  |  |  |  |  |  |  |  |  |  |  |
|  |  |  | Density Q3 |  |  |  |  |  |  |  |  |  |  |  |  |  |  |  |
|  |  |  | Density Q4 |  |  |  |  |  |  |  |  |  |  |  |  |  |  |  |
| Wang et al., 2020 | | Residency | Urban | Chinese MMSE | | | Poor cognitive function | PM2.5 | per 10 μg/m3 | Hazard Ratio | - | - | 1.01 | 0.95 | 1.07 |  | 0.23 | NS |
|  |  |  | Rural |  |  |  |  |  |  |  |  |  | 1.06 | 1.03 | 1.09 | - |  |  |
|  |  | Geographic Region | East China |  |  |  |  |  |  |  |  |  | 1.06 | 1.02 | 1.10 |  | 0.15 | NS |
|  |  |  | Central China |  |  |  |  |  |  |  |  |  | 1.06 | 1.01 | 1.11 | - |  |  |
|  |  |  | West China |  |  |  |  |  |  |  |  |  | 1.15 | 0.97 | 1.37 |  |  |  |
| Nunez et al., 2021 | | Urbanicity | Central Metro | Health administrative data  (coded with ICD-9) | | | Incident AD* | PM2.5 | per 1 μg/m3 | Rate Ratio | - | - | 1.02 | 1.00 | 1.03 | - | NP | NS |
|  |  |  | Fringe Metro |  |  |  |  |  |  |  |  |  | 1.02 | 0.99 | 1.05 |  |  |  |
|  |  |  | Metro |  |  |  |  |  |  |  |  |  | 1.05 | 1.01 | 1.09 |  |  |  |
|  |  |  | Rural |  |  |  |  |  |  |  |  |  | 0.98 | 0.94 | 1.02 |  |  |  |
| Shi et al., 2021 | | Quartiles of population density | Density Q1 | Medicare record data | Dementia | | Diagnosis of dementia | PM2.5 | Per IQR increase | Hazard Ratio | - | | NP | | | Positive | Reference | S |
|  |  |  | Density Q2 |  |  |  |  |  |  |  |  |  |  |  |  |  | 0.001 |  |
|  |  |  | Density Q3 |  |  |  |  |  |  |  |  |  |  |  |  |  | <0.001 |  |
|  |  |  | Density Q4 |  |  |  |  |  |  |  |  |  |  |  |  |  | <0.001 |  |
|  |  |  | Density Q1 |  |  |  |  | NO2 |  |  | - | | NP | | | Positive | Reference | S |
|  |  |  | Density Q2 |  |  |  |  |  |  |  |  |  |  |  |  |  | <0.001 |  |
|  |  |  | Density Q3 |  |  |  |  |  |  |  |  |  |  |  |  |  | <0.001 |  |
|  |  |  | Density Q4 |  |  |  |  |  |  |  |  |  |  |  |  |  | 0.50 |  |
|  |  |  | Density Q1 |  |  |  |  | O3 |  |  | - | | NP | | | Positive | Reference | S |
|  |  |  | Density Q2 |  |  |  |  |  |  |  |  |  |  |  |  |  | 0.06 |  |
|  |  |  | Density Q3 |  |  |  |  |  |  |  |  |  |  |  |  |  | 0.003 |  |
|  |  |  | Density Q4 |  |  |  |  |  |  |  |  |  |  |  |  |  | 0.01 |  |
|  |  |  | Density Q1 |  | AD | | Diagnosis of AD | PM2.5 |  |  | - | | NP | | | Positive | Reference | S |
|  |  |  | Density Q2 |  |  |  |  |  |  |  |  |  |  |  |  |  | 0.006 |  |
|  |  |  | Density Q3 |  |  |  |  |  |  |  |  |  |  |  |  |  | <0.001 |  |
|  |  |  | Density Q4 |  |  |  |  |  |  |  |  |  |  |  |  |  | <0.001 |  |
|  |  |  | Density Q1 |  |  |  |  | NO2 |  |  | - | | NP | | | Positive | Reference | S |
|  |  |  | Density Q2 |  |  |  |  |  |  |  |  |  |  |  |  |  | <0.001 |  |
|  |  |  | Density Q3 |  |  |  |  |  |  |  |  |  |  |  |  |  | <0.001 |  |
|  |  |  | Density Q4 |  |  |  |  |  |  |  |  |  |  |  |  |  | <0.001 |  |
|  |  |  | Density Q1 |  |  |  |  | O3 |  |  | -- | | NP | | | Positive | Reference | S |
|  |  |  | Density Q2 |  |  |  |  |  |  |  |  |  |  |  |  |  | 0.11 |  |
|  |  |  | Density Q3 |  |  |  |  |  |  |  |  |  |  |  |  |  | 0.01 |  |
|  |  |  | Density Q4 |  |  |  |  |  |  |  |  |  |  |  |  |  | 0.01 |  |
| Yao et al., 2021 | | Residency | Rural | Cognitive assessment test | Chinese MMSE | | Cognitive impairment | RD** to main roadway  ( ≤100m vs. >100m) | NP | Odds Ratio | - | - | 1.18 | 1.03 | 1.47 |  | 0.65 | NS |
|  |  |  | Urban |  |  |  |  |  |  |  |  |  | 1.23 | 1.05 | 1.45 |  |  |  |
| Gao et al., 2022 | | Residency | Urban | Cognitive assessment test | Chinese MMSE | | Cognitive impairment | Ozone | per 10 μg/m3 | Hazard Ratio | - | - | 1.03 | 0.92 | 1.15 |  | 0.125 | NS |
|  |  |  | Rural |  |  |  |  |  |  |  |  |  | 1.13 | 1.06 | 1.21 |  |  |  |
|  |  | Geographic Region | Northern China |  |  |  |  |  |  |  |  |  | 0.95 | 0.80 | 1.13 | Not applicable | 0.001 | S |
|  |  |  | Eastern China |  |  |  |  |  |  |  |  |  | 1.13 | 1.03 | 1.25 |  |  |  |
|  |  |  | Central China |  |  |  |  |  |  |  |  |  | 1.32 | 1.16 | 1.50 |  |  |  |
|  |  |  | Western China |  |  |  |  |  |  |  |  |  | 0.85 | 0.71 | 1.01 |  |  |  |
|  |  |  | Southern China |  |  |  |  |  |  |  |  |  | 1.15 | 1.02 | 1.30 |  |  |  |
| M. Li et al., 2022 | | Residence | Urban | CSF sTREM2 score | Neuroinflammatory (a biomarker of AD, represented by CSF sTREM2 score) | | Continuous score | PM2.5 | NP | Z score | - | - | -0.133 | NP | NP | Negative (stronger in Urban residence group) | 0.0002 | S |
|  |  |  | Rural |  |  |  |  |  |  |  | - | - | -0.057 | NP | NP |  | 0.3464 |  |
|  |  |  | Urban |  |  |  |  | O3 |  |  | - | - | -0.090 | NP | NP | Qualitative | 0.0144 |  |
|  |  |  | Rural |  |  |  |  |  |  |  | - | - | 0.076 | NP | NP |  | 0.2115 |  |
| Decrom et al., 2022 | | Living in Ommoord§ | ≥ 10 years before baseline | Dementia assessment test | DSM-III-R*, NINCDS-ADRDA* | | Diagnosis of incident dementia | Marker covering all pollutants (PM10, PM2.5, PM2.5 absorbance, NOx, NO2) | per SD** | Hazard Ratio | - | - | 1.03 | 0.93 | 1.14 |  | NP | NS |
|  |  |  | ≥ 25 years before baseline |  |  |  |  |  |  |  |  |  | 1.05 | 0.94 | 1.18 | - |  |  |
| Wang et al., 2022 | | Region | Northeast | Cognitive assessment test | DSMMD* | | Incident dementia | PM2.5 | per IQR (= 1.78 μg/m3) | Hazard Ratio | - | - | 0.73 | 0.51 | 1.04 | - | 0.64 | NS |
|  |  |  | South |  |  |  |  |  |  |  |  |  | 0.75 | 0.53 | 1.04 |  |  |  |
|  |  |  | Midwest |  |  |  |  |  |  |  |  |  | 0.97 | 0.69 | 1.35 |  |  |  |
|  |  |  | West |  |  |  |  |  |  |  |  |  | 0.80 | 0.69 | 0.93 |  |  |  |
|  |  |  | Northeast |  |  |  |  | NO2 | per IQR (= 1.78 μg/m3) |  |  |  | 0.75 | 0.59 | 0.93 | - | 0.39 | NS |
|  |  |  | South |  |  |  |  |  |  |  |  |  | 0.97 | 0.72 | 1.30 |  |  |  |
|  |  |  | Midwest |  |  |  |  |  |  |  |  |  | 0.94 | 0.65 | 1.38 |  |  |  |
|  |  |  | West |  |  |  |  |  |  |  |  |  | 0.76 | 0.64 | 0.91 |  |  |  |
| Semmens et al., 2022 | | Study site | North Carolina/California/Maryland/Philadelphia | Neuroimaging | MRI | | Incident dementia | PM2.5, NO_2_ | Per IQR increase | Hazard Ratio | - | - | NP | NP | NP | - | NP | NS |
|  | *MMSE: MMSE: Mini-Mental State Examination RD: Residential distance; SIS: Six-Item Screener; ResDAC: Research Data Assistance Center; AD: Alzheimer’s disease; DSM-III-R: Diagnostic and Statistical Manual of Mental Disorders; NINCDS-ADRDA National Institute of Neurological and Communicative Diseases and Stroke/Alzheimer’s Disease and Related Disorders Association; DSMMD: Diagnostic and Statistical Manual of Mental Disorders  ** SD: Standard Deviation; S/NS: Significance/Non-Significance; NP: Not provided  † Model 1 included PM2.5 (mg/m3), length of follow up (days between baseline telephone interview and most recent cognitive assessment), and the potential confounders temperature, season, and incident stroke. Model 2 included the factors in model 1, and added the following demographic factors: age, race, region, education, and income. Model 3 included all the factors in model 2, and added behavioral factors: smoking status, alcohol use, exercise, and BMI. Model 4 included all factors in model 3 and added known comorbidities of cognitive impairment: presence of depressive symptoms, dyslipidemia (present or absent), diabetes (present or absent), and hypertension (present or absent).  ‡ The study only provided graphics without the full list of effect size measures; only parts of measures were presented in text for rural and metropolitan categories.  § The district of Ommoord is a small suburb area in Rotterdam, the Netherlands and is regarded as stable with little environmental changes occurred in the past two decades.  ※ Six studies reported test results of effect modification by residency but didn’t provide full information of effect size estimates but rather provided 1) parts/or none of effect size estimates including graphics with 95% CIs (Shin et al., 2019; Carey et al., 2018) or 2) no effect size estimate with graphics with parts of overlapping 95% Cis in subgroups with in-text statements about lower air pollution effect between dementia/and AD – PM2.5/and NO2 in rural areas. (Shi et al., 2021), Some studies (Ranft et al., 2009) mentioned that the effect modification analysis was performed without providing its results. One study (Shi et al., 2020) provided graphics that indicated subgroups analysis using population density (Q1 – Q4) as an effect modifier between ADRD-PM2.5(per 5 μg/m3 increase) association and marked it as statistically significant (at 0.05 level).  Statistical significance (at 0.05 level): Shi et al., 2020; Shin et al., 2019 (living in rural area showed worse cognitive function with exposure to PM2.5.) | | | | | | | | | | | | | | | | | |

- Table E7: Effect size by potential effect modifier – BMI (1/12)

| **Study** | | **Effect Modifier** | | **Outcome** | | | **Air**  **Pollutant** | **Exposure**  **Level** | **Types of Effect** | **Effect**  **Size**  **(A: Main Effect by exposure)** | **Effect Size**  **(C: Effect by interaction term)** | **Effect Size  Estimate**  **(A+C)** | **95% CI*s** | | **Direction of Modifier Measure** | **p-value  for  interaction terms** | **Statistical Significance (S/NS**)** |
| --- | --- | --- | --- | --- | --- | --- | --- | --- | --- | --- | --- | --- | --- | --- | --- | --- | --- |
|  |  | **BMI** | | **Outcome Measure Method** | | **Outcome Variable** |  |  |  |  |  |  | **lower** | **upper** |  |  |  |
| Power et al., 2011 | | BMI | <25 / ≥ 25 | Cognitive assessment test | 7 tests (MMSE,  the digit span backward test, a verbal fluency  task, constructional praxis, immediate recall  of a 10-word list, delayed recall of a 10-word  list, and a pattern comparison task) | Cognitive score | Black carbon | - | Coefficient | - | - | NP | NP | | - | >0.10 | NS |
| Chen et al., 2015 | | BMI | <25 | Neuroimaging | MRI | Total WM | PM2.5 |  | Coefficients | - | - | -2.86 | NP | | - | 0.12 | NS |
|  |  |  | 25-29 |  |  | Total WM |  | per IQR (3.49 μg/m3) |  |  |  | -6.11 |  |  |  |  |  |
|  |  |  | ≥ 30 |  |  | Total WM |  |  |  |  |  | -9.35 |  |  |  |  |  |
|  |  |  | <25 |  |  | Association Brain WM |  |  |  |  |  | -1.39 |  |  | - | 0.12 | NS |
|  |  |  | 25-29 |  |  | Association Brain WM |  |  |  |  |  | -4.49 |  |  |  |  |  |
|  |  |  | ≥ 30 |  |  | Association Brain WM |  |  |  |  |  | -7.18 |  |  |  |  |  |
|  |  |  | <25 |  |  | Frontal WM |  |  |  |  |  | -0.34 |  |  | - | 0.15 | NS |
|  |  |  | 25-29 |  |  | Frontal WM |  |  |  |  |  | -2.49 |  |  |  |  |  |
|  |  |  | ≥ 30 |  |  | Frontal WM |  |  |  |  |  | -3.04 |  |  |  |  |  |
|  |  |  | <25 |  |  | Parietal WM |  |  |  |  |  | 0.61 |  |  | Qualitative | <0.01 | S |
|  |  |  | 25-29 |  |  | Parietal WM |  |  |  |  |  | -0.47 |  |  |  |  |  |
|  |  |  | ≥ 30 |  |  | Parietal WM |  |  |  |  |  | -2.21 |  |  |  |  |  |
|  |  |  | <25 |  |  | Temporal WM |  |  |  |  |  | -1.66 |  |  | - | 0.87 | NS |
|  |  |  | 25-29 |  |  | Temporal WM |  |  |  |  |  | -1.53 |  |  |  |  |  |
|  |  |  | ≥ 30 |  |  | Temporal WM |  |  |  |  |  | -1.92 |  |  |  |  |  |
|  |  |  | <25 |  |  | Corpus Callosum |  |  |  |  |  | -0.08 |  |  | - | 0.72 | NS |
|  |  |  | 25-29 |  |  | Corpus Callosum |  |  |  |  |  | -0.15 |  |  |  |  |  |
|  |  |  | ≥ 30 |  |  | Corpus Callosum |  |  |  |  |  | -0.11 |  |  |  |  |  |
| Tzivian, Dlugaj, Winkler, Hennig, et al., 2016 | | BMI | ≤30 | Cognitive assessment test | Global Cognitive Score | Continuous score | PM2.5 | Per IQR increase | Coefficient | - | - | (Reference) | NP | NP |  | NP | NS |
|  |  |  | >30 |  |  |  |  |  |  |  |  | Positive |  |  | - |  |  |
| Tzivian, Dlugaj, Winkler, Weinmayr, et al., 2016 | | BMI | ≤30 | Cognitive assessment test | Diagnosis of cognitive impairment | Cognitive impairment | PM2.5 | Per IQR increase | Odd Ratio | - | - | (Reference) | NP | NP | - | NP | NS |
|  |  |  | >30 |  |  |  |  |  |  |  |  | Positive |  |  |  |  |  |
| Tallon et al., 2017 | | BMI | <30 | Cognitive assessment test | CCFM* | CCFM Score | PM2.5 | Per IQR | Coefficient | - | - | -0.15 | -0.38 | 0.07 | - | 0.47 | NS |
|  |  |  | ≥ 30 |  |  |  |  |  |  |  |  | -0.15 | -0.63 | 0.34 |  |  |  |
|  |  |  | <30 |  |  |  | NO2 |  |  |  |  | -0.12 | -0.34 | 0.10 | - | 0.85 | NS |
|  |  |  | ≥ 30 |  |  |  |  |  |  |  |  | 0.01 | -0.31 | 0.33 |  |  |  |
| Younan et al., 2020 | | BMI | <30 | Cognitive assessment test | AD-PS Score* | 5-year stan-  dardized change in AD-PS score | PM 2.5 | Per IQR change | Coefficient | - | - | 0.022 | 0.002 | 0.042 | - | 0.54 | NS |
|  |  |  | ≥ 30 |  |  |  |  |  |  |  |  | 0.032 | 0.004 | 0.061 |  |  |  |
| Ran et al., 2021 | | BMI | Under/normal weight | Health administrative data  (coded with ICD-9) | Dementia | Incident all-cause, vascular dementia and AD | PM2.5 | Per IQR | Hazard Ratio | - | - | 1.05 | 0.97 | 1.15 | - | 0.238 | NS |
|  |  |  | Overweight/obese |  |  |  |  |  |  |  |  | 1.08 | 0.98 | 1.17 |  |  | NS |
| Shaffer et al., 2021 | | Weight | underweight/normal | Dementia assessment test | CASI* | Score below 86 | PM 2.5 | Per 1 ug/m^3^ increase | Hazard Ratio | - | - | 1.17 | 1.04 | 1.32 | - | 0.27 | NS |
|  |  |  | overweight/obese |  |  |  |  |  |  |  |  | 1.14 | 1.00 | 1.29 |  |  |  |
| G.-C. Chen et al., 2022 | | BMI | < 30 | Hospitalization records  (coded with ICD-9 and ICD-10) | Dementia | Incident all-cause dementia | Air pollution score  (PM2.5, PM2.5-10, PM10, NO2, NOx) | Per IQR | Hazard Ratio | - | - | 1.11 | 1.07 | 1.15 |  | 0.077 | NS |
|  |  |  | ≥ 30 |  | Dementia | Incident all-cause dementia |  |  |  |  |  | 1.12 | 1.06 | 1.19 | - |  |  |
|  |  |  | < 30 |  | Dementia | Incident vascular dementia |  |  |  |  |  | 1.11 | 1.02 | 1.2 |  | 0.117 | NS |
|  |  |  | ≥ 30 |  | Dementia | Incident vascular dementia |  |  |  |  |  | 1.12 | 1.00 | 1.25 | - |  |  |
|  |  |  | < 30 |  | AD | Incident AD |  |  |  |  |  | 1.11 | 1.05 | 1.18 |  | 0.178 | NS |
|  |  |  | ≥ 30 |  | AD | Incident AD |  |  |  |  |  | 1.18 | 1.07 | 1.29 | - |  |  |
| M. Li et al., 2022 | | Obesity | Non-obesity | CSF sTREM2 score | Neuroinflammatory (a biomarker of AD, represented by CSF sTREM2 score) | Continuous score | PM2.5 | NP | Z score | - | - | -0.094 | NP | NP | - | 0.0433 | NS** |
|  |  |  | Obesity |  |  |  |  |  |  |  |  | --0.127 | NP | NP | - | 0.0022 |  |
| Wang et al., 2022 | | BMI | < 25 | Cognitive assessment test | DSMMD* | Incident dementia | PM2.5 | Per IQR (=1.78 μg/m3) | Hazard Ratio | - | - | 0.84 | 0.67 | 1.05 |  | 0.41 | NS |
|  |  |  | 25-29 |  |  |  |  |  |  |  |  | 0.85 | 0.71 | 1.02 | - |  |  |
|  |  |  | ≥ 30 |  |  |  |  |  |  |  |  | 0.73 | 0.6 | 0.88 |  |  |  |
|  |  |  | < 25 |  |  |  | NO2 | per IQR (= 3.91 ppb) |  |  |  | 0.93 | 0.75 | 1.16 |  | 0.26 | NS |
|  |  |  | 25-29 |  |  |  |  |  |  |  |  | 0.79 | 0.65 | 0.95 | - |  |  |
|  |  |  | ≥ 30 |  |  |  |  |  |  |  |  | 0.74 | 0.61 | 0.89 |  |  |  |
| Yang et al., 2022 | | BMI | Normal (< 24) | AD assessment test | MMSE*, MoCA*, and HAD* | Diagnosis of incident AD* based on the test score | PM 2.5 | per 10 μg/m3 | Hazard Ratio | - | - | 0.89 | 0.76 | 1.03 |  | NP | NS |
|  |  |  | Overweight (≥ 24 and ≤ 28) |  |  |  |  |  |  |  |  | 1.04 | 1.01 | 1.27 | - |  |  |
|  |  |  | Obese (≥ 28) |  |  |  |  |  |  |  |  | 1.03 | 1.00 | 1.12 |  |  |  |
|  | \| *MMSE: Mini-Mental State Examination; MoCA: Montreal Cognitive Assessment; HAD: Hospital Anxiety and Depression Scale; AD: Alzheimer's Disease; CASI: ; CASI: Cognitive Abilities Screening Instrument; CCFM: Chicago Cognitive Function Measure; DSMMD: Diagnostic and Statistical Manual of Mental Disorders; AD-PS: Alzheimer’s disease pattern similarity  ** Based on in-text information in the original study; S/NS: Significance/Non-Significance; NP: Not provided \| \| --- \| \| § Statistical significance is reported NS because it cannot be judged by shaded overlapping 95% CI without measures. \| \| ※ Five studies tested BMI as an effect modifier but either 1) did not provide effect size measures in the tables, but tested and mentioned non significance (Power et al., 2011;) or 2) only provided graphics without the measures (Tzivian, Dlugaj, Winkler, Weinmayr, et al., 2016; Tzivian, Dlugaj, Winkler, Hennig, et al., 2016). \| | | | | | | | | | | | | | | | | |

- Table E8: Effect size by potential effect modifier – Marital Status (0/2)

| **Study**  **(2 total)** | **Effect Modifier** | | **Outcome** | | | **Air**  **Pollutant** | **Exposure**  **Level** | **Types of Effect** | **Effect**  **Size**  **(A: Main Effect by exposure)** | **Effect Size**  **(C: Effect by interaction term)** | **Effect Size  Estimate**  **(A+C)** | **95% CI*s** | | **p-value  for  interaction terms** | **Statistical Significance (S/NS**)** |
| --- | --- | --- | --- | --- | --- | --- | --- | --- | --- | --- | --- | --- | --- | --- | --- |
|  | **Marital Status** | | **Outcome Measure Method** | | **Outcome Variable** |  |  |  |  |  |  | **lower** | **upper** |  |  |
| Hu et al., 2022 | Marital Status | Single | Cognitive assessment test | Episodic memory, orientation and attention, and visuospatial ability. | A positive difference (Δ cognitive function >0) between the  2011 and 2015 cognitive function assessments | PM1 | per IQR | Odds Ratio | - | - | 0.78 | 0.75 | 0.82 | 0.315 | NS |
|  |  | Married and Live together |  |  |  |  |  |  |  |  | 0.83 | 0.75 | 0.93 |  |  |
|  |  | Single |  |  |  | PM2.5 |  |  |  |  | 0.75 | 0.71 | 0.78 | 0.57 |  |
|  |  | Married and Live together |  |  |  |  |  |  |  |  | 0.72 | 0.64 | 0.8 |  |  |
|  |  | Single |  |  |  | PM10 |  |  |  |  | 0.83 | 0.79 | 0.87 | 0.751 |  |
|  |  | Married and Live together |  |  |  |  |  |  |  |  | 0.82 | 0.73 | 0.91 |  |  |
|  |  | Single |  |  |  | NO2 |  |  |  |  | 0.84 | 0.81 | 0.88 | 0.857 |  |
|  |  | Married and Live together |  |  |  |  |  |  |  |  | 0.87 | 0.78 | 0.97 |  |  |
|  |  | Single |  |  |  | O3 |  |  |  |  | 1.01 | 0.97 | 1.05 | 0.223 |  |
|  |  | Married and Live together |  |  |  |  |  |  |  |  | 1.10 | 0.99 | 1.23 |  |  |
| Yang et al., 2022 | Marital Status | Living alone | Alzheimer’s disease assessment test | MMSE*, MoCA*, and HAD* | Diagnosis of incident AD* based on the test score | PM 2.5 | Per 10 ug/m^3^ | Hazard Ratio | - | - | 1.01 | 0.89 | 1.14 | NP | NS |
|  |  | Cohabitation |  |  |  |  |  |  |  |  | 0.76 | 0.58 | 1.00 |  |  |
| *MMSE: Mini-Mental State Examination; MoCA: Montreal Cognitive Assessment; HAD: Hospital Anxiety and Depression Scale; AD: Alzheimer's Disease;  ** S/NS: Significance/Non-Significance | | | | | | | | | | | | | | | |

- Table E9: Effect size by potential effect modifier – Employment Status (0/3)

| **Study** | **Effect Modifier** | | **Outcome** | | | **Air**  **Pollutant** | **Exposure**  **Level** | **Types of Effect** | **Effect**  **Size**  **(A: Main Effect by exposure)** | **Effect Size**  **(C: Effect by interaction term)** | **Effect Size  Estimate**  **(A+C)** | **95% CI*s** | | **p-value  for  interaction terms** | **Statistical Significance (S/NS**)** |
| --- | --- | --- | --- | --- | --- | --- | --- | --- | --- | --- | --- | --- | --- | --- | --- |
|  | **Employment Status** | | **Outcome Measure Method** | | **Outcome Variable** |  |  |  |  |  |  | **lower** | **upper** |  |  |
| Ailshire & Crimmins, 2014 | Employment status | Employed | Cognitive assessment test | Global Cognitive Score | Continuous score | PM2.5 | Per quartiles | Coefficient | - | - | Not provided | NP** | NP | NP | NS |
|  |  | Not employed |  |  |  |  |  |  |  |  |  |  |  |  |  |
| M. Li et al., 2022 | Employment status | Employed | CSF sTREM2 score | Neuroinflammatory (a biomarker of AD, represented by CSF sTREM2 score) | Continuous score | PM2.5 | NP | Z-score | - | - | -0.111 | NP | NP | 0.0472 | NS** |
|  |  | Not employed |  |  |  |  |  |  |  |  | -0.116 |  |  | 0.0022 |  |
| Yang et al., 2022 | Employment Status | Farmers | Alzheimer’s disease assessment test | MMSE*, MoCA*, and HAD* | Incident AD* based on the test score | PM 2.5 |  | Hazard Ratio | - | - | 1.02 | 1.00 | 1.16 | NP | NS |
|  |  | Blue-collar |  |  |  |  | Per 10 ug/m^3^ |  |  |  | 1.09 | 0.43 | 1.25 |  |  |
|  |  | White-collar |  |  |  |  |  |  |  |  | 0.65 | 0.76 | 1.12 |  |  |
| *MMSE: Mini-Mental State Examination; MoCA: Montreal Cognitive Assessment; HAD: Hospital Anxiety and Depression Scale; AD: Alzheimer's Disease  ** based on in-text information in the original study; S/NS: Significance/Non-Significance; NP: Not provided  ※ Two studies reported test results of effect modification by employment status but provide all/or parts of effect size estimates but rather provided 1) in-text statement (non-significance) (Ailshire and Crimmins 2014) only without information about effect sizes | | | | | | | | | | | | | | | |

- Table E10: Effect size by potential effect modifier - comorbidities (Significant studies: 5/22)

| **Study** | | **Effect Modifier** | | **Outcome** | | | **Exposure** | **Exposure**  **Level** | **Types of Effect** | **Effect**  **Size**  **(A: Main Effect by exposure)** | **Effect Size**  **(C: Effect by interaction term)** | **Effect Size  Estimate*****  **(A+C)** | **95% CI*s** | | **Direction of Modifier Measure** | **p-value  for  interaction terms** | **Statistical Significance (S/NS**)** |
| --- | --- | --- | --- | --- | --- | --- | --- | --- | --- | --- | --- | --- | --- | --- | --- | --- | --- |
|  |  | **Comorbidity** | | **Outcome Measure Method** | | **Outcome Variable** |  |  |  |  |  |  | **lower** | **upper** |  |  |  |
| Power et al. 2011 | | Hypertension | No/Yes | Cognitive assessment test | 7 tests (MMSE,  the digit span backward test, a verbal fluency  task, constructional praxis, immediate recall  of a 10-word list, delayed recall of a 10-word  list, and a pattern comparison task) | Cognitive score | Black carbon | - | - | - | - | - | - | |  | >0.1 | NS |
| Tzivian, Dlugaj, Winkler, Hennig et al., 2016 | | CES-D score (Elevated depressive symptom) | <18 | Cognitive assessment test | Global Cognitive Score | Continuous score | PM2.5 | Per IQR increase | Coefficient | - | - | (Reference) | - | |  | - | NS |
|  |  |  | ≥18 |  |  |  |  |  |  |  |  | Positive |  |  |  |  |  |
| Carey et al., 2018 | | Comorbidity | No/Yes (ischemic heart  Disease, diabetes, heart failure, or stroke) | Health administrative data  (coded with ICD-10) | | Diagnosis of incident dementia | NO_2_ | Per IQR (7.5 μg/m^3^)  increase | Hazard Ratio | - | - | - | - | |  | - | NS |
| Hedges et al., 2019*** | | Overall Health | No | Neuroimaging | MRI | Left HV | PM2.5 | per 1-unit | Coefficients | -30.25 | 0 | -30.25 | NP** | |  | ≥0.05 | NS |
|  |  |  | Yes |  |  |  |  |  |  | -30.25 | 6.54 | -23.71 |  |  |  |  |  |
|  |  |  | No |  |  | Right HV |  |  |  | 10.93 | 0 | 10.93 |  |  |  |  |  |
|  |  |  | Yes |  |  |  |  |  |  | 10.93 | -4.43 | 6.5 |  |  |  |  |  |
|  |  |  | No |  |  | Left HV | PM2.5-10 |  |  | -23.68 | 0 | -23.68 |  |  |  |  |  |
|  |  |  | Yes |  |  |  |  |  |  | -23.68 | 5.66 | -18.02 |  |  |  |  |  |
|  |  |  | No |  |  | Right HV |  |  |  | 12.39 | 0 | 12.39 |  |  |  |  |  |
|  |  |  | Yes |  |  |  |  |  |  | 12.39 | -6.02 | 6.37 |  |  |  |  |  |
|  |  |  | No |  |  | Left HV | PM10 |  |  | -5.05 | 0 | -5.05 |  |  |  |  |  |
|  |  |  | Yes |  |  |  |  |  |  | -5.05 | 0.78 | -4.27 |  |  |  |  |  |
|  |  |  | No |  |  | Right HV |  |  |  | 12.38 | 0 | 12.38 |  |  |  |  |  |
|  |  |  | Yes |  |  |  |  |  |  | 12.38 | -4.41 | 7.97 |  |  |  |  |  |
|  |  |  | No |  |  | Left HV | NO2 |  |  | -1.73 | 0 | -1.73 |  |  |  |  |  |
|  |  |  | Yes |  |  |  |  |  |  | -1.73 | 0.33 | -1.4 |  |  |  |  |  |
|  |  |  | No |  |  | Right HV |  |  |  | 2.13 | 0 | 2.13 |  |  |  |  |  |
|  |  |  | Yes |  |  |  |  |  |  | 2.13 | -0.62 | 1.51 |  |  |  |  |  |
|  |  |  | No |  |  | Left HV | NO2 |  |  | -0.74 | 0 | -0.74 |  |  |  |  |  |
|  |  |  | Yes |  |  |  |  |  |  | -0.74 | 0.1 | -0.64 |  |  |  |  |  |
|  |  |  | No |  |  | Right HV |  |  |  | 0.94 | 0 | 0.94 |  |  |  |  |  |
|  |  |  | Yes |  |  |  |  |  |  | 0.94 | -0.29 | 0.65 |  |  |  |  |  |
| Salinas-Rodriguez et al., 2018 | | Diabetes | No | Cognitive assessment test | 3WMT | Cognitive impairment | PM2.5 | per 10 μg/m3 | Odds Ratio | - | - | 1.39 | 1.07 | 1.81 |  | 0.67 | NS |
|  |  |  | Yes |  |  |  |  |  |  |  |  | 1.37 | 1.07 | 1.76 |  |  |  |
|  |  |  | No |  | SVF Test | SVF score |  |  | Coefficient |  |  | -0.68 | -1.02 | -0.34 |  | 0.5 |  |
|  |  |  | Yes |  |  |  |  |  |  |  |  | -0.70 | -1.03 | -0.37 |  |  |  |
|  |  | Hypertension | No |  | 3WMT | Cognitive impairment |  |  | Odds Ratio |  |  | 1.45 | 1.07 | 1.97 |  | 0.46 |  |
|  |  |  | Yes |  |  |  |  |  |  |  |  | 1.43 | 1.08 | 1.89 |  |  |  |
|  |  |  | No |  | SVF Test | SVF score |  |  | Coefficient |  |  | -0.75 | -1.11 | -0.38 |  | 0.73 |  |
|  |  |  | Yes |  |  |  |  |  |  |  |  | -0.74 | -1.09 | -0.39 |  |  |  |
| Tzivian, Dlugaj, Winkler, Weinmayr, et al., 2016 † | | Depression  (CES-D scale) | No | Cognitive assessment test | MCI Diagnosis | Prevalence of MCI | PM2.5 | per IQR** | Odds Ratio | - | - | 1.13 | 0.97 | 1.31 |  | 0.43 | NS |
|  |  |  | Yes |  |  |  |  |  |  |  |  | 1.35 | 0.89 | 2.05 |  |  |  |
| Yao et al., 2021 | | Depression  (CES-D scale) | No | Cognitive assessment test | Chinese MMSE | Cognitive impairment | RD** to main roadway  ( ≤100m vs. >100m) | NP | Odds Ratio | - | - | 1.10 | 0.94 | 1.29 |  | 0.21 | NS |
|  |  |  | Yes |  |  |  |  |  |  |  |  | 1.29 | 1.13 | 1.48 |  |  |  |
| Tallon et al., 2017 | | CRP* | Low  (<1) | Cognitive assessment test | CCFM* | CCFM Score | PM2.5 | per IQR | Coefficient | - | - | -0.23 | -0.71 | 0.29 |  | 0.28 | NS |
|  |  |  | Elevated (>1) |  |  |  |  |  |  |  |  | -0.21 | -0.46 | 0 |  |  |  |
|  |  | Diabetes | No |  |  |  |  |  |  |  |  | -0.16 | -0.41 | 0.09 |  | 0.44 | NS |
|  |  |  | Yes |  |  |  |  |  |  |  |  | -0.41 | -0.97 | 0.16 |  |  |  |
|  |  | HbA1c* | <6.5 |  |  |  |  |  |  |  |  | -0.10 | -0.41 | 0.20 |  | 0.32 | NS |
|  |  |  | ≥6.5 |  |  |  |  |  |  |  |  | -0.72 | -1.45 | 0 |  |  |  |
|  |  | Hypertension | No |  |  |  |  |  |  |  |  | -0.23 | -0.45 | -0.01 |  | 0.15 | NS |
|  |  |  | Yes |  |  |  |  |  |  |  |  | -0.3 | -0.16 | 0.77 |  |  |  |
|  |  | Stroke | No |  |  |  |  |  |  |  |  | -0.48 | -0.82 | -0.13 | Negative | 0.046 | S |
|  |  |  | Yes |  |  |  |  |  |  |  |  | -0.03 | -0.82 | 0.77 |  |  |  |
|  |  | IADL* | No |  |  |  |  |  |  |  |  | -0.23 | -0.45 | -0.01 |  | 0.42 | NS |
|  |  |  | Yes |  |  |  |  |  |  |  |  | 0.94 | 0.47 | 1.41 |  |  |  |
|  |  | Depression  (CESD-11) | No |  |  |  |  |  |  |  |  | -0.19 | -0.44 | 0.77 |  | 0.94 | NS |
|  |  |  | Yes |  |  |  |  |  |  |  |  | -0.90 | -1.49 | -0.32 |  |  |  |
|  |  | Anxiety  (HADS) | No |  |  |  |  |  |  |  |  | -0.41 | -0.67 | -0.19 | Negative | 0.03 | S |
|  |  |  | Yes |  |  |  |  |  |  |  |  | -0.15 | -0.75 | 0.44 |  |  |  |
|  |  | Stress  (PSS) | No |  |  |  |  |  |  |  |  | -0.35 | -0.59 | -0.11 |  | 0.01 | NS |
|  |  |  | Yes |  |  |  |  |  |  |  |  | -0.19 | -0.70 | 0.32 |  |  |  |
|  |  | Social  Connectedness | No |  |  |  |  |  |  |  |  | 0.05 | -0.33 | 0.43 |  | 0.15 | NS |
|  |  |  | Yes |  |  |  |  |  |  |  |  | -0.36 | -1.03 | 0.30 |  |  |  |
|  |  | Loneliness | No |  |  |  |  |  |  |  |  | -0.22 | -0.56 | 0.11 | Not applicable  (same effect sizes) | 0.06 | S |
|  |  |  | Yes |  |  |  |  |  |  |  |  | -0.22 | -1.12 | 0.69 |  |  |  |
|  |  | CRP | Low  (<1) |  |  |  | NO2 |  |  |  |  | -0.14 | -0.36 | 0.08 |  | 0.11 | NS |
|  |  |  | Elevated (>1) |  |  |  |  |  |  |  |  | -0.26 | -0.60 | 0.07 |  |  |  |
|  |  | Diabetes | No |  |  |  |  |  |  |  |  | -0.01 | -0.34 | 0.14 |  | 0.60 | NS |
|  |  |  | Yes |  |  |  |  |  |  |  |  | -0.30 | -0.67 | 0.07 |  |  |  |
|  |  | HbA1c | <6.5 |  |  |  |  |  |  |  |  | -0.11 | -0.39 | 0.18 |  | 0.91 | NS |
|  |  |  | ≥6.5 |  |  |  |  |  |  |  |  | -0.62 | -1.07 | -0.17 |  |  |  |
|  |  | Hypertension | No |  |  |  |  |  |  |  |  | -0.01 | -0.31 | 0.12 |  | 0.66 | NS |
|  |  |  | Yes |  |  |  |  |  |  |  |  | -0.08 | -0.39 | -0.23 |  |  |  |
|  |  | Stroke | No |  |  |  |  |  |  |  |  | -0.18 | -0.53 | 0.17 |  | 0.62 | NS |
|  |  |  | Yes |  |  |  |  |  |  |  |  | -0.54 | -1.07 | -0.01 |  |  |  |
|  |  | IADL | No |  |  |  |  |  |  |  |  | -0.15 | -0.36 | 0.06 | Qualitative | 0.047 | S |
|  |  |  | Yes |  |  |  |  |  |  |  |  | 0.86 | 0.54 | 1.18 |  |  |  |
|  |  | Depression  (CESD-11) | No |  |  |  |  |  |  |  |  | 0.02 | -0.23 | 0.26 | Qualitative | 0.053 | S |
|  |  |  | Yes |  |  |  |  |  |  |  |  | -1.09 | -1.46 | -0.71 |  |  |  |
|  |  | Anxiety  (HADS) | No |  |  |  |  |  |  |  |  | -0.12 | -0.38 | 0.13 |  | 0.38 | NS |
|  |  |  | Yes |  |  |  |  |  |  |  |  | -0.71 | -0.10 | -0.31 |  |  |  |
|  |  | Elevated stress | No |  |  |  |  |  |  |  |  | -0.18 | -0.41 | 0.04 |  | 0.93 | NS |
|  |  |  | Yes |  |  |  |  |  |  |  |  | -0.83 | -1.17 | -0.48 |  |  |  |
|  |  | Social  Connectedness | No |  |  |  |  |  |  |  |  | -0.02 | -0.40 | 0.36 |  | 0.35 | NS |
|  |  |  | Yes |  |  |  |  |  |  |  |  | 0.27 | -0.16 | 0.69 |  |  |  |
|  |  | Loneliness | No |  |  |  |  |  |  |  |  | -0.04 | -0.28 | 0.21 |  | 0.27 | NS |
|  |  |  | Yes |  |  |  |  |  |  |  |  | -0.22 | -0.59 | 0.15 |  |  |  |
| Yu et al., 2020 | | Obesity | No | Cognitive assessment test | Modified MMSE and SEVLT | Incident dementia,  Cognitive Impairment without Dementia (CIND) | NOx (≥3.44 ppb) | NP | Hazard Ratio | - | - | 1.30 | 0.67 | 2.60 |  | NP | NS |
|  |  |  | Yes |  |  |  |  |  |  |  |  | 1.70 | 0.99 | 3.00 |  |  |  |
|  |  | Hyperglycemia | No |  |  |  |  |  |  |  |  | 1.10 | 0.57 | 2.20 |  |  | NS |
|  |  |  | Yes |  |  |  |  |  |  |  |  | 2.40 | 1.40 | 4.00 |  |  |  |
|  |  | Low HDL-cholesterol | No |  |  |  |  |  |  |  |  | 1.00 | 0.58 | 1.80 |  |  | NS |
|  |  |  | Yes |  |  |  |  |  |  |  |  | 2.50 | 1.40 | 4.30 |  |  |  |
| Ran et al., 2021 | | Hypertension | Yes | Hospitalization records  (coded with ICD-9) | | Incident all-cause dementia  (including AD*, vascular dementia) | PM2.5 | per IQR | Hazard Ratio | - | - | 1.08 | 0.98 | 1.19 |  | 0.60 | NS |
|  |  |  | No |  |  |  |  |  |  |  |  | 1.06 | 0.98 | 1.14 |  |  |  |
|  |  | Diabetes | Yes |  |  |  |  |  |  |  |  | 0.97 | 0.79 | 1.19 |  | 0.51 | NS |
|  |  |  | No |  |  |  |  |  |  |  |  | 1.07 | 1.01 | 1.15 |  |  |  |
|  |  | Heart Disease | Yes |  |  |  |  |  |  |  |  | 1.12 | 0.95 | 1.31 |  | 0.80 | NS |
|  |  |  | No |  |  |  |  |  |  |  |  | 1.06 | 0.99 | 1.13 |  |  |  |
| Grande et al., 2021 | | CVD* | No | Global cognitive assessment | MMSE | Fast cognitive decline | PM2.5 (≤8.6 g/m3) | NP | Odds Ratio | - | - | 1.46 | 1.06 | 2.01 | Positive | <0.001 | S |
|  |  |  | Yes |  |  |  |  |  |  |  |  | 1.57 | 1.13 | 2.16 |  |  |  |
|  |  |  | No |  |  |  | PM2.5 (> 8.6g/m3) |  |  |  |  | 0.87 | 0.76 | 1.01 |  | 0.40 | NS |
|  |  |  | Yes |  |  |  |  |  |  |  |  | 0.83 | 0.65 | 1.06 |  |  |  |
| Wang et al., 2022 | | Hyper-  cholesterolemia | No | Cognitive assessment test | DSMMD* | Incident dementia | PM2.5 | per IQR (= 1.78 μg/m3) | Hazard Ratio | - | - | 0.80 | 0.70 | 0.91 |  | 0.90 | NS |
|  |  |  | Yes |  |  |  |  |  |  |  |  | 0.81 | 0.63 | 1.06 |  |  |  |
|  |  | Hypertension | No |  |  |  |  |  |  |  |  | 0.82 | 0.71 | 0.94 |  | 0.63 | NS |
|  |  |  | Yes |  |  |  |  |  |  |  |  | 0.77 | 0.63 | 0.95 |  |  |  |
|  |  | CVD History | No |  |  |  |  |  |  |  |  | 0.79 | 0.69 | 0.90 |  | 0.41 | NS |
|  |  |  | Yes |  |  |  |  |  |  |  |  | 0.91 | 0.66 | 1.25 |  |  |  |
|  |  | Diabetes | No |  |  |  |  |  |  |  |  | 0.81 | 0.71 | 0.92 |  | 0.43 | NS |
|  |  |  | Yes |  |  |  |  |  |  |  |  | 0.65 | 0.37 | 1.12 |  |  |  |
|  |  | Hyper-  cholesterolemia | No |  |  |  | NO2 | per IQR (= 3.91 ppb) |  |  |  | 0.83 | 0.72 | 0.94 |  | 0.22 | NS |
|  |  |  | Yes |  |  |  |  |  |  |  |  | 0.69 | 0.53 | 0.90 |  |  |  |
|  |  | Hypertension | No |  |  |  |  |  |  |  |  | 0.80 | 0.69 | 0.92 |  | 0.91 | NS |
|  |  |  | Yes |  |  |  |  |  |  |  |  | 0.81 | 0.66 | 0.99 |  |  |  |
|  |  | CVD History | No |  |  |  |  |  |  |  |  | 0.81 | 0.71 | 0.92 |  | 0.72 | NS |
|  |  |  | Yes |  |  |  |  |  |  |  |  | 0.75 | 0.53 | 1.08 |  |  |  |
|  |  | Diabetes | No |  |  |  |  |  |  |  |  | 0.82 | 0.72 | 0.93 |  | 0.12 | NS |
|  |  |  | Yes |  |  |  |  |  |  |  |  | 0.54 | 0.32 | 0.91 |  |  |  |
| G.-C. Chen et al., 2022 | | Diabetes | No | Hospitalization records  (coded with ICD-9 and ICD-10) | | Incident all-cause dementia | Air pollution score  (PM2.5, PM2.5-10, PM10, NO2, NOx) | per IQR | Hazard Ratio | - | - | 1.11 | 1.07 | 1.15 |  | 0.56 | NS |
|  |  |  | Yes |  |  |  |  |  |  |  |  | 1.14 | 1.05 | 1.23 |  |  |  |
|  |  |  | No |  |  | Incident vascular dementia |  |  |  |  |  | 1.11 | 1.03 | 1.19 |  | 0.86 | NS |
|  |  |  | Yes |  |  |  |  |  |  |  |  | 1.12 | 0.99 | 1.28 |  |  |  |
|  |  |  | No |  |  | Incident AD |  |  |  |  |  | 1.13 | 1.08 | 1.19 |  | 0.72 | NS |
|  |  |  | Yes |  |  |  |  |  |  |  |  | 1.11 | 0.97 | 1.26 |  |  |  |
|  |  | Hypertension | No |  |  | Incident all-cause dementia |  |  |  |  |  | 1.12 | 1.06 | 1.19 |  | 0.22 | NS |
|  |  |  | Yes |  |  |  |  |  |  |  |  | 1.11 | 1.07 | 1.15 |  |  |  |
|  |  |  | No |  |  | Incident vascular dementia |  |  |  |  |  | 1.16 | 1.01 | 1.32 |  | 0.47 | NS |
|  |  |  | Yes |  |  |  |  |  |  |  |  | 1.10 | 1.03 | 1.19 |  |  |  |
|  |  |  | No |  |  | Incident AD |  |  |  |  |  | 1.16 | 1.07 | 1.27 |  | 1.73 | NS |
|  |  |  | Yes |  |  |  |  |  |  |  |  | 1.11 | 1.05 | 1.18 |  |  |  |
|  |  | CVD History | No |  |  | Incident all-cause dementia |  |  |  |  |  | 1.10 | 1.07 | 1.14 |  | 0.15 | NS |
|  |  |  | Yes |  |  |  |  |  |  |  |  | 1.14 | 1.07 | 1.23 |  |  |  |
|  |  |  | No |  |  | Incident vascular dementia |  |  |  |  |  | 1.12 | 1.04 | 1.20 |  | 0.89 | NS |
|  |  |  | Yes |  |  |  |  |  |  |  |  | 1.09 | 0.98 | 1.23 |  |  |  |
|  |  |  | No |  |  | Incident AD |  |  |  |  |  | 1.13 | 1.07 | 1.19 |  | 0.98 | NS |
|  |  |  | Yes |  |  |  |  |  |  |  |  | 1.13 | 1.00 | 1.26 |  |  |  |
|  |  | Respiratory diseases | No |  |  | Incident all-cause dementia |  |  |  |  |  | 1.12 | 1.08 | 1.15 |  | 0.90 | NS |
|  |  |  | Yes |  |  |  |  |  |  |  |  | 1.10 | 0.96 | 1.25 |  |  |  |
|  |  |  | No |  |  | Incident vascular dementia |  |  |  |  |  | 1.12 | 1.05 | 1.20 |  | 0.68 | NS |
|  |  |  | Yes |  |  |  |  |  |  |  |  | 1.09 | 0.91 | 1.45 |  |  |  |
|  |  |  | No |  |  | Incident AD |  |  |  |  |  | 1.13 | 1.08 | 1.19 |  | 0.79 | NS |
|  |  |  | Yes |  |  |  |  |  |  |  |  | 1.13 | 0.92 | 1.38 |  |  |  |
| Hedges et al., 2020*** | | Self-rated health | No | Neuroimaging | MRI | Left Thalamus Volume | PM2.5 |  | Coefficients | -33.58 | 0 | -33.58 | -4.07 | 22.20 |  | ≥0.05 | NS |
|  |  |  | Yes |  |  |  |  | NP |  | -33.58 | 9.07 | -24.51 |  |  |  |  |  |
|  |  |  | No |  |  |  | PM2.5-10 |  |  | -57.87 | 0 | -57.87 | 0.21 | 30.44 | Negative | <0.05 | S |
|  |  |  | Yes |  |  |  |  |  |  | -57.87 | 15.33 | -42.54 |  |  |  |  |  |
|  |  |  | No |  |  |  | PM10 |  |  | -20.1 | 0 | -20.1 | -1.85 | 12.57 |  | ≥0.05 | NS |
|  |  |  | Yes |  |  |  |  |  |  | -20.1 | 5.36 | -14.74 |  |  |  |  |  |
|  |  |  | No |  |  |  | NO2 |  |  | 0.39 | 0 | 0.39 | -2.06 | 1.80 |  |  |  |
|  |  |  | Yes |  |  |  |  |  |  | 0.39 | -0.13 | 0.26 |  |  |  |  |  |
|  |  |  | No |  |  |  | NOx |  |  | -0.67 | 0 | -0.67 |  |  |  |  |  |
|  |  |  | Yes |  |  |  |  |  |  | -0.67 | 0.12 | -0.55 | -0.81 | 1.05 |  |  |  |
|  |  |  | No |  |  | Right Thalamus Volume | PM2.5 |  |  | -31.47 | 0 | -31.47 | -4.17 | 21.00 |  |  |  |
|  |  |  | Yes |  |  |  |  |  |  | -31.47 | 8.42 | -23.05 |  |  |  |  |  |
|  |  |  | No |  |  |  | PM2.5-10 |  |  | -49.7 | 0 | -49.7 | -0.77 | 28.20 |  |  |  |
|  |  |  | Yes |  |  |  |  |  |  | -49.7 | 13.71 | -35.99 |  |  |  |  |  |
|  |  |  | No |  |  |  | PM10 |  |  | -15.14 | 0 | -15.14 | -2.71 | 11.11 |  |  |  |
|  |  |  | Yes |  |  |  |  |  |  | -15.14 | 4.2 | -10.94 |  |  |  |  |  |
|  |  |  | No |  |  |  | NO2 |  |  | -1.02 | 0 | -1.02 | -1.60 | 2.09 |  |  |  |
|  |  |  | Yes |  |  |  |  |  |  | -1.02 | 0.25 | -0.77 |  |  |  |  |  |
|  |  |  | No |  |  |  | NOx |  |  | -1.47 | 0 | -1.47 |  |  |  |  |  |
|  |  |  | Yes |  |  |  |  |  |  | -1.47 | 0.32 | -1.15 | -0.57 | 1.21 |  |  |  |
| Gale et al., 2020*** | | Self-rated health | No | Neuroimaging | MRI | Left Frontal Pole | PM2.5 |  | Coefficients | -61.2 | 0 | -61.2 | NP | |  | ≥0.05 | NS |
|  |  |  | Yes |  |  |  |  |  |  | -61.2 | 4.17 | -57.03 |  |  |  |  |  |
|  |  |  | No |  |  | Right Frontal Pole |  |  |  | -148.14 | 0 | -148.14 |  |  |  |  |  |
|  |  |  | Yes |  |  |  |  |  |  | -148.14 | 34.83 | -113.31 |  |  |  |  |  |
|  |  |  | No |  |  | Left Frontal Pole | PM2.5-10 |  |  | -183.26 | 0 | -183.26 |  |  |  |  |  |
|  |  |  | Yes |  |  |  |  |  |  | -183.26 | 49.01 | -134.25 |  |  |  |  |  |
|  |  |  | No |  |  | Right Frontal Pole |  |  |  | -360.37 | 0 | -360.37 |  |  | Negative | <0001 | S |
|  |  |  | Yes |  |  |  |  |  |  | -360.37 | 100.22 | -260.15 |  |  |  |  |  |
|  |  |  | No |  |  | Left Frontal Pole | PM10 |  |  | 69.2 | 0 | 69.2 |  |  |  | ≥0.05 | NS |
|  |  |  | Yes |  |  |  |  |  |  | -69.2 | 15.88 | -53.32 |  |  |  |  |  |
|  |  |  | No |  |  | Right Frontal Pole |  |  |  | -138.72 | 0 | -138.72 |  |  | Negative | <0.05 | S |
|  |  |  | Yes |  |  |  |  |  |  | -138.72 | 37.39 | -101.33 |  |  |  |  |  |
|  |  |  | No |  |  | Left Frontal Pole | NO2 |  |  | 4.78 | 0 | 4.78 |  |  |  | ≥0.05 | NS |
|  |  |  | Yes |  |  |  |  |  |  | 4.78 | -3.32 | 1.46 |  |  |  |  |  |
|  |  |  | No |  |  | Right Frontal Pole |  |  |  | -6.45 | 0 | -6.45 |  |  |  |  |  |
|  |  |  | Yes |  |  |  |  |  |  | -6.45 | 0.07 | -6.38 |  |  |  |  |  |
|  |  |  | No |  |  | Left Frontal Pole | NOx |  |  | 5.44 | 0 | 5.44 |  |  |  |  |  |
|  |  |  | Yes |  |  |  |  |  |  | 5.44 | -2.84 | 2.6 |  |  |  |  |  |
|  |  |  | No |  |  | Right Frontal Pole |  |  |  | 0.63 | 0 | 0.63 |  |  |  |  |  |
|  |  |  | Yes |  |  |  |  |  |  | 0.63 | -1.54 | -0.91 |  |  |  |  |  |
|  |  |  | No |  |  | Left Superior Gyrus | PM2.5 |  |  | -57.22 | 0 | -57.22 |  |  |  |  |  |
|  |  |  | Yes |  |  |  |  |  |  | -57.22 | 14.98 | -42.24 |  |  |  |  |  |
|  |  |  | No |  |  | Right Superior Gyrus |  |  |  | 18.54 | 0 | 18.54 |  |  |  |  |  |
|  |  |  | Yes |  |  |  |  |  |  | 18.54 | -8.33 | 10.21 |  |  |  |  |  |
|  |  |  | No |  |  | Left Superior Gyrus | PM2.5-10 |  |  | -53.55 | 0 | -53.55 |  |  |  |  |  |
|  |  |  | Yes |  |  |  |  |  |  | -53.55 | 5.1 | -48.45 |  |  |  |  |  |
|  |  |  | No |  |  | Right Superior Gyrus |  |  |  | 22.2 | 0 | 22.2 |  |  |  |  |  |
|  |  |  | Yes |  |  |  |  |  |  | 22.2 | -11.09 | 11.11 |  |  |  |  |  |
|  |  |  | No |  |  | Left Superior Gyrus | PM10 |  |  | -30.82 | 0 | -30.82 |  |  |  |  |  |
|  |  |  | Yes |  |  |  |  |  |  | -30.82 | 7.32 | -23.5 |  |  |  |  |  |
|  |  |  | No |  |  | Right Superior Gyrus |  |  |  | 7.24 | 0 | 7.24 |  |  |  |  |  |
|  |  |  | Yes |  |  |  |  |  |  | 7.24 | -2.71 | 4.53 |  |  |  |  |  |
|  |  |  | No |  |  | Left Superior Gyrus | NO2 |  |  | -2.72 | 0 | -2.72 |  |  |  |  |  |
|  |  |  | Yes |  |  |  |  |  |  | -2.72 | 1.35 | -1.37 |  |  |  |  |  |
|  |  |  | No |  |  | Right Superior Gyrus |  |  |  | 5.85 | 0 | 5.85 |  |  |  |  |  |
|  |  |  | Yes |  |  |  |  |  |  | 5.85 | -1.55 | 4.3 |  |  |  |  |  |
|  |  |  | No |  |  | Left Superior Gyrus | NOx |  |  | 1.78 | 0 | 1.78 |  |  |  |  |  |
|  |  |  | Yes |  |  |  |  |  |  | 1.78 | -0.65 | 1.13 |  |  |  |  |  |
|  |  |  | No |  |  | Right Superior Gyrus |  |  |  | 4.77 | 0 | 4.77 |  |  |  |  |  |
|  |  |  | Yes |  |  |  |  |  |  | 4.77 | -1.59 | 3.18 |  |  |  |  |  |
|  |  |  | No |  |  | Left Medial Cortex | PM2.5 |  |  | -0.67 | 0 | -0.67 |  |  |  |  |  |
|  |  |  | Yes |  |  |  |  |  |  | -0.67 | -0.39 | -1.06 |  |  |  |  |  |
|  |  |  | No |  |  | Right Medial Cortex |  |  |  | 4.44 | 0 | 4.44 |  |  |  |  |  |
|  |  |  | Yes |  |  |  |  |  |  | 4.44 | -2.06 | 2.38 |  |  |  |  |  |
|  |  |  | No |  |  | Left Medial Cortex | PM2.5-10 |  |  | -5.26 | 0 | -5.26 |  |  |  |  |  |
|  |  |  | Yes |  |  |  |  |  |  | -5.26 | 0.11 | -5.15 |  |  |  |  |  |
|  |  |  | No |  |  | Right Medial Cortex |  |  |  | -15.41 | 0 | -15.41 |  |  |  |  |  |
|  |  |  | Yes |  |  |  |  |  |  | -15.41 | 3.82 | -11.59 |  |  |  |  |  |
|  |  |  | No |  |  | Left Medial Cortex | PM10 |  |  | -0.18 | 0 | -0.18 |  |  |  |  |  |
|  |  |  | Yes |  |  |  |  |  |  | -0.18 | -0.49 | -0.67 |  |  |  |  |  |
|  |  |  | No |  |  | Right Medial Cortex |  |  |  | -2.84 | 0 | -2.84 |  |  |  |  |  |
|  |  |  | Yes |  |  |  |  |  |  | -2.84 | 0.5 | -2.34 |  |  |  |  |  |
|  |  |  | No |  |  | Left Medial Cortex | NO2 |  |  | 0.54 | 0 | 0.54 |  |  |  |  |  |
|  |  |  | Yes |  |  |  |  |  |  | 0.54 | -0.11 | 0.43 |  |  |  |  |  |
|  |  |  | No |  |  | Right Medial Cortex |  |  |  | 1.82 | 0 | 1.82 |  |  |  |  |  |
|  |  |  | Yes |  |  |  |  |  |  | 1.82 | -0.56 | 1.26 |  |  |  |  |  |
|  |  |  | No |  |  | Left Medial Cortex | NOx |  |  | 0.76 | 0 | 0.76 |  |  |  |  |  |
|  |  |  | Yes |  |  |  |  |  |  | 0.76 | -0.26 | 0.5 |  |  |  |  |  |
|  |  |  | No |  |  | Right Medial Cortex |  |  |  | 1.08 | 0 | 1.08 |  |  |  |  |  |
|  |  |  | Yes |  |  |  |  |  |  | 1.08 | -0.37 | 0.71 |  |  |  |  |  |
|  |  |  | No |  |  | Left Orbital Cortex | PM2.5 |  |  | -26.9 | 0 | -26.9 |  |  |  |  |  |
|  |  |  | Yes |  |  |  |  |  |  | -26.9 | 5.73 | -21.17 |  |  |  |  |  |
|  |  |  | No |  |  | Right Orbital Cortex |  |  |  | -7.86 | 0 | -7.86 |  |  |  |  |  |
|  |  |  | Yes |  |  |  |  |  |  | -7.86 | -0.82 | -8.68 |  |  |  |  |  |
|  |  |  | No |  |  | Left Orbital Cortex | PM2.5-10 |  |  | -20.22 | 0 | -20.22 |  |  |  |  |  |
|  |  |  | Yes |  |  |  |  |  |  | -20.22 | 3.82 | -16.4 |  |  |  |  |  |
|  |  |  | No |  |  | Right Orbital Cortex |  |  |  | -34.61 | 0 | -34.61 |  |  |  |  |  |
|  |  |  | Yes |  |  |  |  |  |  | -34.61 | 8.05 | -26.56 |  |  |  |  |  |
|  |  |  | No |  |  | Left Orbital Cortex | PM10 |  |  | -4.7 | 0 | -4.7 |  |  |  |  |  |
|  |  |  | Yes |  |  |  |  |  |  | -4.7 | 0.32 | -4.38 |  |  |  |  |  |
|  |  |  | No |  |  | Right Orbital Cortex |  |  |  | -6.06 | 0 | -6.06 |  |  |  |  |  |
|  |  |  | Yes |  |  |  |  |  |  | -6.06 | 0.71 | -5.35 |  |  |  |  |  |
|  |  |  | No |  |  | Left Orbital Cortex | NO2 |  |  | 1.01 | 0 | 1.01 |  |  |  |  |  |
|  |  |  | Yes |  |  |  |  |  |  | 1.01 | -0.55 | 0.46 |  |  |  |  |  |
|  |  |  | No |  |  | Right Orbital Cortex |  |  |  | 2.75 | 0 | 2.75 |  |  |  |  |  |
|  |  |  | Yes |  |  |  |  |  |  | 2.75 | -1.24 | 1.51 |  |  |  |  |  |
|  |  |  | No |  |  | Left Orbital Cortex | NOx |  |  | 0.41 | 0 | 0.41 |  |  |  |  |  |
|  |  |  | Yes |  |  |  |  |  |  | 0.41 | -0.38 | 0.03 |  |  |  |  |  |
|  |  |  | No |  |  | Right Orbital Cortex |  |  |  | 1.43 | 0 | 1.43 |  |  |  |  |  |
|  |  |  | Yes |  |  |  |  |  |  | 1.43 | -0.78 | 0.65 |  |  |  |  |  |
|  |  |  | No |  |  | Left Operculum Cortex | PM2.5 |  |  | -2.74 | 0 | -2.74 |  |  |  |  |  |
|  |  |  | Yes |  |  |  |  |  |  | -2.74 | -0.77 | -3.51 |  |  |  |  |  |
|  |  |  | No |  |  | Right Operculum Cortex |  |  |  | 7.41 | 0 | 7.41 |  |  |  |  |  |
|  |  |  | Yes |  |  |  |  |  |  | 7.41 | -2.04 | 5.37 |  |  |  |  |  |
|  |  |  | No |  |  | Left Operculum Cortex | PM2.5-10 |  |  | -13.41 | 0 | -13.41 |  |  |  |  |  |
|  |  |  | Yes |  |  |  |  |  |  | -13.41 | 2.78 | -10.63 |  |  |  |  |  |
|  |  |  | No |  |  | Right Operculum Cortex |  |  |  | 2.65 | 0 | 2.65 |  |  |  |  |  |
|  |  |  | Yes |  |  |  |  |  |  | 2.65 | -1.49 | 1.16 |  |  |  |  |  |
|  |  |  | No |  |  | Left Operculum Cortex | PM10 |  |  | -1.07 | 0 | -1.07 |  |  |  |  |  |
|  |  |  | Yes |  |  |  |  |  |  | -1.07 | -0.76 | -1.83 |  |  |  |  |  |
|  |  |  | No |  |  | Right Operculum Cortex |  |  |  | 4.08 | 0 | 4.08 |  |  |  |  |  |
|  |  |  | Yes |  |  |  |  |  |  | 4.08 | -1.27 | 2.81 |  |  |  |  |  |
|  |  |  | No |  |  | Left Operculum Cortex | NO2 |  |  | 0.8 | 0 | 0.8 |  |  |  |  |  |
|  |  |  | Yes |  |  |  |  |  |  | 0.8 | -0.46 | 0.34 |  |  |  |  |  |
|  |  |  | No |  |  | Right Operculum Cortex |  |  |  | 0.87 | 0 | 0.87 |  |  |  |  |  |
|  |  |  | Yes |  |  |  |  |  |  | 0.87 | -0.27 | 0.6 |  |  |  |  |  |
|  |  |  | No |  |  | Left Operculum Cortex | NOx |  |  | 0.48 | 0 | 0.48 |  |  |  |  |  |
|  |  |  | Yes |  |  |  |  |  |  | 0.48 | -0.3 | 0.18 |  |  |  |  |  |
|  |  |  | No |  |  | Right Operculum Cortex |  |  |  | 0.59 | 0 | 0.59 |  |  |  |  |  |
|  |  |  | Yes |  |  |  |  |  |  | 0.59 | -0.22 | 0.37 |  |  |  |  |  |
| Chen et al., 2017 | | Stroke | Yes | Health administrative data  (coded with ICD-9 or ICD-10) | | Incident dementia | NO2 | per IQR (=14.2 ppb, 4.8 μg/m3 for NO2 and PM2.5, respectively ) | Hazard Ratio | - | - | NP | NP | | - | 0.77 | NS |
|  |  |  | No |  |  |  |  |  |  |  |  |  |  |  |  |  |  |
|  |  |  | Yes |  |  |  | PM2.5 |  |  |  |  |  |  |  | Qualitative | 0.00 | S |
|  |  |  | No |  |  |  |  |  |  |  |  |  |  |  |  |  |  |
|  |  | Diabetes | Yes |  |  |  | NO2 |  |  |  |  |  |  |  | - | 0.33 | NS |
|  |  |  | No |  |  |  |  |  |  |  |  |  |  |  |  |  |  |
|  |  |  | Yes |  |  |  | PM2.5 |  |  |  |  |  |  |  | Negative | 0.03 | S |
|  |  |  | No |  |  |  |  |  |  |  |  |  |  |  |  |  |  |
|  |  | Hypertension | Yes |  |  |  | NO2 |  |  |  |  |  |  |  | - | 0.22 | NS |
|  |  |  | No |  |  |  |  |  |  |  |  |  |  |  |  |  |  |
|  |  |  | Yes |  |  |  | PM2.5 |  |  |  |  |  |  |  | - | 0.07 | NS |
|  |  |  | No |  |  |  |  |  |  |  |  |  |  |  |  |  |  |
| Chen et al., 2015 | | Existing CVD | No | Neuroimaging | MRI | Total WM* | PM2.5 | per IQR (3.49μg/m3) | Coefficients | - | - | -5.90 | NP | | - | 0.50 | NS |
|  |  |  | Yes |  |  |  |  |  |  |  |  | -8.35 |  |  |  |  |  |
|  |  |  | No |  |  | Association Brain WM |  |  |  |  |  | -4.11 |  |  |  | 0.40 | NS |
|  |  |  | Yes |  |  |  |  |  |  |  |  | -6.80 |  |  |  |  |  |
|  |  |  | No |  |  | Frontal WM |  |  |  |  |  | -1.83 |  |  |  | 0.35 | NS |
|  |  |  | Yes |  |  |  |  |  |  |  |  | -3.40 |  |  |  |  |  |
|  |  |  | No |  |  | Parietal WM |  |  |  |  |  | -0.61 |  |  |  | 0.36 | NS |
|  |  |  | Yes |  |  |  |  |  |  |  |  | -1.51 |  |  |  |  |  |
|  |  |  | No |  |  | Temporal WM |  |  |  |  |  | -1.67 |  |  |  | 0.81 | NS |
|  |  |  | Yes |  |  |  |  |  |  |  |  | -1.90 |  |  |  |  |  |
|  |  |  | No |  |  | Corpus Callosum |  |  |  |  |  | -0.10 |  |  |  | 0.26 | NS |
|  |  |  | Yes |  |  |  |  |  |  |  |  | -0.23 |  |  |  |  |  |
|  |  | Diabetes Mellitus | No |  |  | Total WM |  |  |  |  |  | -6.25 |  |  |  | 0.50 | NS |
|  |  |  | Yes |  |  |  |  |  |  |  |  | -3.89 |  |  |  |  |  |
|  |  |  | No |  |  | Association Brain WM |  |  |  |  |  | -4.47 |  |  |  | 0.40 | NS |
|  |  |  | Yes |  |  |  |  |  |  |  |  | -3.94 |  |  |  |  |  |
|  |  |  | No |  |  | Frontal WM |  |  |  |  |  | -2.05 |  |  |  | 0.35 | NS |
|  |  |  | Yes |  |  |  |  |  |  |  |  | -1.25 |  |  |  |  |  |
|  |  |  | No |  |  | Parietal WM |  |  |  |  |  | -0.71 |  |  |  | 0.36 | NS |
|  |  |  | Yes |  |  |  |  |  |  |  |  | -2.69 |  |  |  |  |  |
|  |  |  | No |  |  | Temporal WM |  |  |  |  |  | -1.72 |  |  |  | 0.81 | NS |
|  |  |  | Yes |  |  |  |  |  |  |  |  | 0.01 |  |  |  |  |  |
|  |  |  | No |  |  | Corpus Callosum |  |  |  |  |  | -0.12 |  |  |  | 0.26 | NS |
|  |  |  | Yes |  |  |  |  |  |  |  |  | -0.15 |  |  |  |  |  |
|  |  | Hypertension | No |  |  | Total WM |  |  |  |  |  | -6.25 |  |  |  | 0.50 | NS |
|  |  |  | Yes |  |  |  |  |  |  |  |  | -3.89 |  |  |  |  |  |
|  |  |  | No |  |  | Association Brain WM |  |  |  |  |  | -4.47 |  |  |  | 0.40 | NS |
|  |  |  | Yes |  |  |  |  |  |  |  |  | -3.94 |  |  |  |  |  |
|  |  |  | No |  |  | Frontal WM |  |  |  |  |  | -2.05 |  |  |  | 0.35 | NS |
|  |  |  | Yes |  |  |  |  |  |  |  |  | -1.25 |  |  |  |  |  |
|  |  |  | No |  |  | Parietal WM |  |  |  |  |  | -0.71 |  |  |  | 0.36 | NS |
|  |  |  | Yes |  |  |  |  |  |  |  |  | -2.69 |  |  |  |  |  |
|  |  |  | No |  |  | Temporal WM |  |  |  |  |  | -1.72 |  |  |  | 0.81 | NS |
|  |  |  | Yes |  |  |  |  |  |  |  |  | 0.01 |  |  |  |  |  |
|  |  |  | No |  |  | Corpus Callosum |  |  |  |  |  | -0.12 |  |  |  | 0.26 | NS |
|  |  |  | Yes |  |  |  |  |  |  |  |  | -0.15 |  |  |  |  |  |
| Gao et al., 2022 | | Disability | Yes | Cognitive assessment test | Chinese MMSE | Cognitive impairment | Ozone | per 10 μg/m3 | Hazard Ratio | - | - | 1.00 | 0.87 | 1.14 | - | 0.10 | NS |
|  |  |  | No |  |  |  |  |  |  |  |  | 1.13 | 1.06 | 1.20 |  |  |  |
|  |  | Chronic Disease | Yes |  |  |  |  |  |  |  |  | 1.11 | 1.02 | 1.20 |  | 0.90 | NS |
|  |  |  | No |  |  |  |  |  |  |  |  | 1.10 | 1.02 | 1.18 |  |  |  |
| Crous-Bou et al., 2020 | | Family history of AD | No | Neuroimaging | MRI | AD* signature for cortical thickness | NO2 | NP | Coefficients | - | - | -26.80 | NP | | - | 0.45 | NS |
|  |  |  | Yes |  |  |  |  |  |  |  |  | -14.30 |  |  |  |  |  |
|  |  |  | No |  |  |  | NOx |  |  |  |  | -72.80 |  |  |  | 0.41 | NS |
|  |  |  | Yes |  |  |  |  |  |  |  |  | -76.30 |  |  |  |  |  |
|  |  |  | No |  |  |  | PM2.5 |  |  |  |  | -4.71 |  |  |  | 0.15 | NS |
|  |  |  | Yes |  |  |  |  |  |  |  |  | -1.65 |  |  |  |  |  |
|  |  |  | No |  |  |  | PM10 |  |  |  |  | -7.68 |  |  |  | 0.53 | NS |
|  |  |  | Yes |  |  |  |  |  |  |  |  | -4.67 |  |  |  |  |  |
|  |  |  | No |  |  |  | PM2.5-abs |  |  |  |  | -1.32 |  |  |  | 0.38 | NS |
|  |  |  | Yes |  |  |  |  |  |  |  |  | -0.51 |  |  |  |  |  |
|  |  |  | No |  |  |  | PMcoarse |  |  |  |  | -2.92 |  |  |  | 0.74 | NS |
|  |  |  | Yes |  |  |  |  |  |  |  |  | -2.87 |  |  |  |  |  |
| Younan et al., 2020 | | Hypertension | No | Cognitive assessment test | AD-PS Score* | 5-year standardized change in AD-PS score | PM 2.5 | Per IQR change | Coefficient | - | - | 0.034 | 0.014 | 0.055 | - | 0.11 | NS |
|  |  |  | Yes |  |  |  |  |  |  |  |  | 0.007 | -0.021 | 0.036 |  |  |  |
|  |  | High cholesterol | No |  |  |  |  |  |  |  |  | 0.031 | 0.013 | 0.049 |  | 0.15 |  |
|  |  |  | Yes |  |  |  |  |  |  |  |  | 0.001 | -0.037 | 0.039 |  |  |  |
|  |  | Diabetes | No |  |  |  |  |  |  |  |  | 0.026 | 0.009 | 0.043 |  | 0.55 |  |
|  |  |  | Yes |  |  |  |  |  |  |  |  | -0.037 | -0.244 | 0.170 |  |  |  |
|  |  | Cardiovascular disease | No |  |  |  |  |  |  |  |  | 0.029 | 0.011 | 0.047 |  | 0.34 |  |
|  |  |  | Yes |  |  |  |  |  |  |  |  | 0.008 | -0.034 | 0.049 |  |  |  |
|  |  | White matter lesions (at MRI-1) | Low (≤ 2.7) |  |  |  |  |  |  |  |  | 0.032 | 0.010 | 0.054 |  | 0.52 |  |
|  |  |  | High (> 2.7) |  |  |  |  |  |  |  |  | 0.021 | -0.003 | 0.045 |  |  |  |
| Wang et al., 2020 | | DADL | No | Cognitive assessment test | Chinese MMSE | Poor cognitive function | PM2.5 | per 10 μg/m3 | Hazard Ratio | - | - | 1.06 | 1.03 | 1.09 | - | 0.29 | NS |
|  |  |  | Yes |  |  |  |  |  |  |  |  | 0.99 | 0.92 | 1.07 |  |  |  |
|  |  | Comorbidity | Yes |  |  |  |  |  |  |  |  | 1.08 | 1.01 | 1.15 |  | 0.73 | NS |
|  |  |  | No |  |  |  |  |  |  |  |  | 1.05 | 1.02 | 1.08 |  |  |  |
| Semmens et al., 2022 | | History of cardiovascular disease/angina/heart attack/stroke/heart failure/high blood pressure/diabetes | No/Yes | Neuroimaging | MRI | Incident dementia | PM2.5, NO_2_ | Per IQR increase | Hazard Ratio |  |  | NP | NP | NP |  | NP | NS |
|  | * AD: Alzheimer's Disease; CCFM: Chicago Cognitive Function Measure; MRI: Magnetic Resonance Imaging; MMSE: Mini-Mental State Examination; WM: White Matter; IADL: Impaired Activities of Daily Living; HbA1c: Glycosylated hemoglobin; CRP: C-reactive Protein; CVD: Cardiovascular Disease; SEVLT: Spanish English Verbal Learning Test; DSMMD: Diagnostic and Statistical Manual of Mental Disorders; 3WMT: Three-word memory test; SVF: Semantic Verbal fluency; MCI: Mild Cognitive Function; HV: Hippocampal Volumes; DADL: Disability in activities of daily living; AD-PS: Alzheimer’s disease pattern similarity  ** CI: Confidence Interval; S/NS: Significance/Non-Significance; IQR: Inter-Quartile Range; RD: Residential Distance; NP: Not provided  *** The total effect size (A+C) was manually calculated unless the effect of air pollution exposure on each category of the effect modifier was provided in the studies  **** 95% CI is based on coefficient of effect modifiers  † p-value of interaction term was provided in text selectively for certain modifiers  ※ 3 studies reported test results of effect modification by comorbidity status but provide all/or parts of effect size estimates but rather provided 1) graphics with no measures (Carey et al., 2018; Tzivian, Dlugaj, Winkler, Hennig, et al., 2016) or 2) did not provide effect size measures in the tables but tested and mentioned non-significance of the modifier (Power et al., 2011). | | | | | | | | | | | | | | | | |

- Table E11: Effect size by potential effect modifier – Dietary Diversity (3/6)

| **Study** | | **Effect Modifier** | | **Outcome** | | | **Air**  **Pollutant** | **Exposure**  **Level** | **Types of Effect** | **Effect**  **Size**  **(A: Main Effect by exposure)** | **Effect Size**  **(C: Effect by interaction term)** | **Effect Size  Estimate**  **(A+C)** | **95% CI*s** | | **Direction of Modifier Measure** | **p-value  for  interaction terms** | **Statistical Significance (S/NS)** |
| --- | --- | --- | --- | --- | --- | --- | --- | --- | --- | --- | --- | --- | --- | --- | --- | --- | --- |
|  |  | **Dietary Diversity** | | **Outcome Measure Method** | | **Outcome Variable** |  |  |  |  |  |  | **lower** | **upper** |  |  |  |
| Chen et al., 2021 | | MIND*-like diet score | <6.5 | Neuroimaging | MRI | Total brain volume | PM2.5 | per IQR(=3.22 μg/m3) | Coefficient | - | - | −0.51 | −2.28 | 1.25 | - | 0.69 | NS |
|  |  |  | ≥6.5 |  |  |  |  |  |  |  |  | −0.17 | −1.50 | 1.16 |  |  |  |
|  |  |  | <6.5 |  |  | Normal brain volume |  |  |  |  |  | −0.41 | −2.81 | 1.99 | - | 0.78 | NS |
|  |  |  | ≥6.5 |  |  |  |  |  |  |  |  | 1.04 | -0.79 | 2.87 |  |  |  |
|  |  |  | <6.5 |  |  | Total WM* volume |  |  |  |  |  | -12.47 | -17.17 | -7.78 | Qualitative | <0.001 | S |
|  |  |  | ≥6.5 |  |  |  |  |  |  |  |  | 0.16 | -3.41 | 3.72 |  |  |  |
|  |  |  | <6.5 |  |  | Frontal lobe volume |  |  |  |  |  | -4.56 | -6.61 | -2.51 | Qualitative | <0.001 | S |
|  |  |  | ≥6.5 |  |  |  |  |  |  |  |  | 0.7 | -0.99 | 2.39 |  |  |  |
|  |  |  | <6.5 |  |  | Parietal lobe volume |  |  |  |  |  | -1.97 | -3.35 | -0.59 | Qualitative | <0.001 | S |
|  |  |  | ≥6.5 |  |  |  |  |  |  |  |  | 0.9 | -0.1 | 1.9 |  |  |  |
|  |  |  | <6.5 |  |  | Temporal lobe volume |  |  |  |  |  | -3.27 | -4.44 | -2.1 | Negative | <0.001 | S |
|  |  |  | ≥6.5 |  |  |  |  |  |  |  |  | -0.48 | -1.35 | 0.4 |  |  |  |
|  |  |  | <6.5 |  |  | Corpus callosum volume |  |  |  |  |  | -0.16 | -0.29 | -0.02 | - | 0.45 | NS |
|  |  |  | ≥6.5 |  |  |  |  |  |  |  |  | -0.09 | -0.19 | 0.01 |  |  |  |
|  |  |  | <6.5 |  |  | Hippocampus volume |  |  |  |  |  | -0.01 | -0.12 | 0.11 |  | 0.48 | NS |
|  |  |  | ≥6.5 |  |  |  |  |  |  |  |  | -0.02 | -0.11 | 0.07 | - |  |  |
| Yao et al., 2021 | | Dietary Diversity | <5 | Cognitive assessment test | Chinese MMSE | Cognitive impairment | RD to main roadway  ( ≤100m vs. >100m) | NP** | Odds Ratio | - | - | 1.19 | 1.05 | 1.35 |  | 0.68 | NS |
|  |  |  | ≥6 |  |  |  |  |  |  |  |  | 1.22 | 1.03 | 1.47 | - |  |  |
| C. Chen et al., 2022 | | Folate intake | <500.09 (ug/d) | Cognitive assessment test | WHIMS* protocol | Incident all-cause dementia | PM2.5 | NP | Hazard Ratio | - | - | 1.53 | 1.07 | 2.21 | Negative | 0.02 | S |
|  |  |  | ≥500.09 |  |  |  |  |  |  |  |  | 0.86 | 0.59 | 1.24 |  |  |  |
|  |  | B12 intake | <10.46 |  |  |  |  |  |  |  |  | 1.47 | 1.02 | 2.11 | Negative | 0.051 | S |
|  |  |  | ≥10.46 |  |  |  |  |  |  |  |  | 0.9 | 0.62 | 1.3 |  |  |  |
|  |  | B6 intake | <2.41 |  |  |  |  |  |  |  |  | 1.5 | 1.03 | 2.19 | Negative | 0.046 | S |
|  |  |  | ≥2.41 |  |  |  |  |  |  |  |  | 0.91 | 0.64 | 1.3 |  |  |  |
| He et al., 2022 | | Eating fish/ a week | <3 | Cognitive assessment test | DSM-IV*, NINCDS-ADRDA* | Incident AD | PM2.5 (>50-60 μg/m3) | NP | Odds Ratio | - | - | 1.80 | 1.39 | 2.33 |  | 0.21‡ | NS |
|  |  |  | ≥3 |  |  |  |  |  |  |  |  | 1.38 | 0.78 | 2.47 | - |  |  |
|  |  |  | <3 |  |  |  | PM2.5 (>60 μg/m3) |  |  |  |  | 5.18 | 3.93 | 6.82 |  | 0.05‡ | NS |
|  |  |  | ≥3 |  |  |  |  |  |  |  |  | 2.89 | 1.50 | 5.59 | - |  |  |
|  |  |  | <3 |  |  |  | PM10 (≤70 mg/m3) |  |  |  |  | 0.66 | 0.23 | 1.86 |  | 0.20‡ | NS |
|  |  |  | ≥3 |  |  |  |  |  |  |  |  | 0.28 | 0.05 | 1.50 | - |  |  |
|  |  |  | <3 |  |  |  | PM10 (>80 mg/m3) |  |  |  |  | 1.91 | 0.86 | 4.24 |  | 0.40‡ | NS |
|  |  |  | ≥3 |  |  |  |  |  |  |  |  | 1.67 | 0.84 | 3.31 | - |  |  |
|  |  |  | <3 |  |  |  | CO (≤ 0.8 mg/m3) |  |  |  |  | 0.35 | 0.15 | 0.81 |  | 0.30‡ | NS |
|  |  |  | ≥3 |  |  |  |  |  |  |  |  | 0.18 | 0.04 | 0.87 | - |  |  |
|  |  |  | <3 |  |  |  | CO (0.9> mg/m3) |  |  |  |  | 0.39 | 0.16 | 0.91 | - | 0.42‡ | NS |
|  |  |  | ≥3 |  |  |  |  |  |  |  |  | 0.44 | 0.20 | 0.94 |  |  |  |
|  |  |  | <3 |  |  |  | NO2 (>30-40 μg/m3) |  |  |  |  | 0.60 | 0.14 | 2.50 |  | 0.44‡ | NS |
|  |  |  | ≥3 |  |  |  |  |  |  |  |  | 0.70 | 0.18 | 2.78 | - |  |  |
|  |  |  | <3 |  |  |  | NO2 (>40 μg/m3) |  |  |  |  | 0.93 | 0.24 | 3.63 |  | 0.50‡ | NS |
|  |  |  | ≥3 |  |  |  |  |  |  |  |  | 0.93 | 0.28 | 3.12 | -- |  |  |
|  |  |  | <3 |  |  |  | SO2(15-25 μg/m3) |  |  |  |  | 0.43 | 0.15 | 1.25 |  | 0.49‡ | NS |
|  |  |  | ≥3 |  |  |  |  |  |  |  |  | 0.44 | 0.19 | 1.03 | - |  |  |
|  |  |  | <3 |  |  |  | SO2(>25 μg/m3) |  |  |  |  | 1.17 | 0.42 | 3.24 |  | 0.44‡ | NS |
|  |  |  | ≥3 |  |  |  |  |  |  |  |  | 1.28 | 0.63 | 2.60 | - |  |  |
|  |  |  | <3 |  |  |  | O3(>90-100 μg/m3) |  |  |  |  | 0.34 | 0.18 | 0.66 |  | 0.32‡ | NS |
|  |  |  | ≥3 |  |  |  |  |  |  |  |  | 0.42 | 0.24 | 0.75 | - |  |  |
|  |  |  | <3 |  |  |  | O3(>100 μg/m3) |  |  |  |  | 0.50 | 0.22 | 1.13 | - | 0.43‡ | NS |
|  |  |  | ≥3 |  |  |  |  |  |  |  |  | 0.45 | 0.22 | 0.93 |  |  |  |
| Zhu et al., 2022 | | Plant-based diet | Lower PDI* Index | Cognitive assessment test | MMSE* | Poor cognitive function (MMSE<18) | PM2.5 | Per 10 mg/m3 | Hazard Ratio | - | - | 1.68 | 1.26 | 2.24 | Negative | 0.04 | S |
|  |  |  | Higher PDI Index |  |  |  |  |  |  |  |  | 1.28 | 0.98 | 1.68 |  |  |  |
| Semmens et al., 2022 | | Ginko *biloba* treatment | Placebo/Ginko treatment | Neuroimaging | MRI | Incident dementia | PM2.5, NO_2_ | Per IQR increase | Hazard Ratio | - | - | NP | NP | NP | - | NP | NS |
|  | *MIND: Mediterranean–DASH Intervention for Neurodegenerative Delay (as “brain healthy” foods); WM: White Matter; WHIMS: the Women’s Health Initiative (WHI) Memory Study; DSM-IV: Diagnostic and Statistical Manual of Mental Disorders; NINCDS-ADRDA: National Institute of Neurological and Communicative Disorders and Stroke and the Alzheimer’s Disease and Related Disorders Association-Alzheimer’s  Criteria; MMSE: Mini-Mental State Examination; PDI: Plant-based diet index  ** S/NS: Significance/Non-Significance; NP: Not provided  ‡ p-value is based on ratio of odds ratio, not interaction terms | | | | | | | | | | | | | | | | |

- Table E12: Effect size by potential effect modifier – Genetic indicator (7/26)

| **Study** | | **Effect Modifier** | | **Outcome** | | | | | **Air**  **Pollutant** | **Exposure**  **Level** | **Types of Effect** | **Effect**  **Size**  **(A: Main Effect by exposure)** | **Effect Size**  **(C: Effect by interaction term)** | **Effect Size  Estimate*****  **(A+C)** | **95% CI*s** | | **Direction of Modifier Measure** | **p-value  for  interaction terms** | **Statistical Significance (S/NS**)** |
| --- | --- | --- | --- | --- | --- | --- | --- | --- | --- | --- | --- | --- | --- | --- | --- | --- | --- | --- | --- |
|  |  | **Genetic Modifier** | | **Outcome Measure Method** | | | | **Outcome Variable** |  |  |  |  |  |  | **lower** | **upper** |  |  |  |
| Power et al., 2013 | | HFE* C282Y | Wild Type | Cognitive assessment test | | | MMSE* | MMSE* score 25 ≥ | Black Carbon | per 1-unit | Odds Ratio | - | - | 1.37 | 1.08 | 1.73 | - | 0.20 | NS |
|  |  |  | Variant |  |  |  |  |  |  |  |  |  |  | 0.91 | 0.50 | 1.64 |  |  |  |
|  |  | HFE* H63D | Wild Type |  |  |  |  |  |  |  |  |  |  | 1.24 | 0.97 | 1.59 |  | 0.22 |  |
|  |  |  | Variant |  |  |  |  |  |  |  |  |  |  | 1.74 | 1.06 | 2.87 |  |  |  |
|  |  | HFE* C282Y | Wild Type |  |  |  |  | Difference in MMSE* score (z-score) |  |  | Coefficient |  |  | -0.061 | -0.115 | -0.007 |  | 0.11 |  |
|  |  |  | Variant |  |  |  |  |  |  |  |  |  |  | 0.073 | -0.081 | 0.228 |  |  |  |
|  |  | HFE* H63D | Wild Type |  |  |  |  |  |  |  |  |  |  | -0.071 | -0.132 | -0.009 |  | 0.64 |  |
|  |  |  | Variant |  |  |  |  |  |  |  |  |  |  | -0.024 | -0.131 | 0.082 |  |  |  |
| Colicino et al., 2014 | | Mitochondrial DNA (mtDNA) | Cluster 1 carriers (J and T haplogroups) | Cognitive assessment test | | | MMSE* | MMSE* score 25 ≥ | Black Carbon | Per 0.7 Unit | Odds Ratio | - | - | 1.6 | 0.9 | 2.9 | - | NP** | NS |
|  |  |  | Cluster 2 carriers (H and V haplogroups) |  |  |  |  |  |  |  |  |  |  | 1.1 | 0.8 | .15 |  |  |  |
|  |  |  | Cluster 3 carriers (K and U haplogroups) |  |  |  |  |  |  |  |  |  |  | 1.0 | 0.6 | 1.6 |  |  |  |
|  |  |  | Cluster 4 carriers (I, W and V haplogroups) |  |  |  |  |  |  |  |  |  |  | 2.7 | 1,3 | 5,6 |  |  |  |
| Wu et al., 2015 | |  | Non-carrier | NINCDS-ADRDA* criteria | | | | Diagnosis of AD* | PM10 | 2^nd^ tertile | Odds Ratio | - | - | 1.19 | 0.57 | 2.48 | - | 0.17 | NS |
|  |  |  | carrier |  |  |  |  |  |  |  |  |  |  | 2.67 | 0.92 | 7.71 |  |  |  |
|  |  |  | Non-Carrier |  |  |  |  |  |  | 3^rd^ tertile |  |  |  | 4.24 | 2.11 | 8.54 |  |  |  |
|  |  |  | Carrier |  |  |  |  |  |  |  |  |  |  | 3.50 | 1.08 | 11.29 |  |  |  |
|  |  |  | Non-carrier |  |  |  |  |  | O_3_ | 2^nd^ tertile |  |  |  | 0.48 | 0.23 | 1.00 |  | 0.65 | NS |
|  |  |  | Carrier |  |  |  |  |  |  |  |  |  |  | 0.85 | 2.80 | 2.52 |  |  |  |
|  |  |  | Non-Carrier |  |  |  |  |  |  | 3^rd^ tertile |  |  |  | 1.80 | 0.92 | 3.55 |  |  |  |
|  |  |  | Carrier |  |  |  |  |  |  |  |  |  |  | 2.55 | 0.88 | 7.35 |  |  |  |
|  |  |  | Non-carrier | NINDS-AIREN* criteria | | | | Diagnosis of VaD* | PM 10 | 2^nd^ tertile |  |  |  | 1.45 | 0.65 | 3.24 |  | P ≥ 0.05 | NS |
|  |  |  | Carrier |  |  |  |  |  |  |  |  |  |  | NP | | |  |  |  |
|  |  |  | Non-Carrier |  |  |  |  |  |  | 3^rd^ tertile |  |  |  | 2.60 | 1.12 | 6.02 |  |  |  |
|  |  |  | Carrier |  |  |  |  |  |  |  |  |  |  | NP | | |  |  |  |
|  |  |  | Non-carrier |  |  |  |  |  | O_3_ | 2^nd^ tertile |  |  |  | 0.50 | 0.21 | 1.21 |  | 0.56 | NS |
|  |  |  | Carrier |  |  |  |  |  |  |  |  |  |  | 0.82 | 0.03 | 21.68 |  |  |  |
|  |  |  | Non-Carrier |  |  |  |  |  |  | 3^rd^ tertile |  |  |  | 1.47 | 0.67 | 3.22 |  |  |  |
|  |  |  | Carrier |  |  |  |  |  |  |  |  |  |  | NP | | |  |  |  |
| Schikowski et al., 2015 | | APOE E4 | Non-carrier | Cognitive assessment test | 10 subtests of CERAD-plus* | | | Standardized z-score | Traffic load | Per IQR (26.9 roads) increase | Coefficient | - | - | 0.1134 | NA | NA | Qualitative | 0.0152 | S |
|  |  |  | Carrier |  |  |  |  |  |  |  |  |  |  | -0.5338 |  |  |  |  |  |
|  |  |  | Non-carrier |  |  |  |  |  | NO_2_ | Per IQR (14.6 ug/m^3^)  increase |  |  |  | -0.3274 |  |  | - | 0.5097 | NS |
|  |  |  | Carrier |  |  |  |  |  |  |  |  |  |  | -0.3778 |  |  |  |  |  |
|  |  |  | Non-carrier |  |  |  |  |  | PM 10 | Per IQR (7.1 ug/m^3^) increase |  |  |  | -0.3175 |  |  |  | 0.1932 |  |
|  |  |  | Carrier |  |  |  |  |  |  |  |  |  |  | -0.3567 |  |  |  |  |  |
|  |  |  | Non-carrier |  |  |  |  |  | PM 2.5 | Per IQR (4.7 ug/m^3^) increase |  |  |  | -0.3168 |  |  |  | 0.4884 |  |
|  |  |  | Carrier |  |  |  |  |  |  |  |  |  |  | -0.3204 |  |  |  |  |  |
|  |  |  | Non-carrier |  |  |  |  |  | PM 2.5 abs* | Per IQR (1.0 10^-^m^-1^) increase |  |  |  | -0.1797 |  |  |  | 0.038 |  |
|  |  |  | Carrier |  |  |  |  |  |  |  |  |  |  | -0.4121 |  |  |  |  |  |
|  |  |  | Non-carrier |  |  |  |  |  | NO_x_ | Per IQR (43.1 ug/m^3^) increase |  |  |  | -0.3171 |  |  |  | 0.9951 |  |
|  |  |  | Carrier |  |  |  |  |  |  |  |  |  |  | -0.3032 |  |  |  |  |  |
| Colicino et al., 2016 | | SNPs* in microRNA | Heterozygous carriers of rs11077 XPO5 | Cognitive assessment test | MMSE* | | | MMSE*(score 25 ≥ | Black Carbon | Per 0.69 unit increase | Odds Ratio | - | - | 1.99 | 1.39 | 2,85 | - | NP | NS |
|  |  |  | Minor variant carriers of GEMIN4 rs2740348 |  |  |  |  |  |  |  |  |  |  | 1.34 | 1.05 | 1.70 |  |  |  |
| Fehsel et al., 2016 | | Estrogen Receptor beta polymorphisms | carriers of minor alleles of rs1256062 | Cognitive assessment test | Figure copying subtest | | | Z score | PM 2.5 | Per IQR (4.7 ug/m^3^) increase | Coefficient | - | - | NP | | | positive | 0.031 | S |
|  |  |  | carriers of two major alleles of rs1256062 |  |  |  |  |  |  |  |  |  |  |  |  |  |  |  |  |
|  |  |  | carriers of minor alleles of rs1256062 |  |  |  |  |  | PM 10 | Per IQR (7.1 ug/m^3^) increase |  |  |  |  |  |  | - | 0.604 | NS |
|  |  |  | carriers of two major alleles of rs1256062 |  |  |  |  |  |  |  |  |  |  |  |  |  |  |  |  |
|  |  |  | carriers of minor alleles of rs1256062 |  |  |  |  |  | PM 2.5 abs* | Per IQR (1.0 10^-5^m^-1^) increase |  |  |  |  |  |  | - | 0.059 | NS |
|  |  |  | carriers of two major alleles of rs1256062 |  |  |  |  |  |  |  |  |  |  |  |  |  |  |  |  |
|  |  |  | carriers of minor alleles of rs1256062 |  |  |  |  |  | NO_x_ | Per IQR (43.1 ug/m^3^) increase |  |  |  |  |  |  | - | 0.444 | NS |
|  |  |  | carriers of two major alleles of rs1256062 |  |  |  |  |  |  |  |  |  |  |  |  |  |  |  |  |
|  |  |  | carriers of minor alleles of rs1256062 |  |  |  |  |  | NO_2_ | Per IQR (14.6 ug/m^3^) increase |  |  |  |  |  |  | positive | 0.026 | S |
|  |  |  | carriers of two major alleles of rs1256062 |  |  |  |  |  |  |  |  |  |  |  |  |  |  |  |  |
|  |  |  | carriers of minor alleles of rs1256062 |  | CERAD* total score | | |  | PM 2.5 | Per IQR (4.7 ug/m^3^) increase |  |  |  |  |  |  | - | 0.867 | NS |
|  |  |  | carriers of two major alleles of rs1256062 |  |  |  |  |  |  |  |  |  |  |  |  |  |  |  |  |
|  |  |  | carriers of minor alleles of rs1256062 |  |  |  |  |  | PM 10 | Per IQR (7.1 ug/m^3^) increase |  |  |  |  |  |  | positive | 0.034 | S |
|  |  |  | carriers of two major alleles of rs1256062 |  |  |  |  |  |  |  |  |  |  |  |  |  |  |  |  |
|  |  |  | carriers of minor alleles of rs1256062 |  |  |  |  |  | PM 2.5 abs* | Per IQR (1.0 10^-5^m^-1^) increase |  |  |  |  |  |  | - | 0.93 | NS |
|  |  |  | carriers of two major alleles of rs1256062 |  |  |  |  |  |  |  |  |  |  |  |  |  |  |  |  |
|  |  |  | carriers of minor alleles of rs1256062 |  |  |  |  |  | NO_x_ | Per IQR (43.1 ug/m^3^) increase |  |  |  |  |  |  | positive | 0.042 | S |
|  |  |  | carriers of two major alleles of rs1256062 |  |  |  |  |  |  |  |  |  |  |  |  |  |  |  |  |
|  |  |  | carriers of minor alleles of rs1256062 |  |  |  |  |  | NO_2_ | Per IQR (14.6 ug/m^3^) increase |  |  |  |  |  |  | - | 0.873 | NS |
|  |  |  | carriers of two major alleles of rs1256062 |  |  |  |  |  |  |  |  |  |  |  |  |  |  |  |  |
| Tzivian, Dlugaj, Winkler, Hennig, et al., 2016† | | APOE E4 | Non-carrier | Cognitive assessment test | Global Cognitive Score | | | Continuous score | PM2.5 | Per IQR increase | Coefficient | - | - | (Reference) | NP | NP | - | NP | NS |
|  |  |  | Carrier |  |  |  |  |  |  |  |  |  |  | Negative |  |  |  |  |  |
| Tzivian, Dlugaj, Winkler, Weinmayr, et al., 2016 | | APOE E4 | Non-carrier | Cognitive assessment test | MCI *diagnosis | | | Prevalence of MCI | PM 2.5 | Per IQR (2.09 ug/m^3^) increase | Odds Ratio | - | - | (Reference) | NP | NP | - | NP | NS |
|  |  |  | Carrier |  |  |  |  |  |  |  |  |  |  | Negative | NP | NP |  |  |  |
| Cacciottolo et al., 2017 | | APOE Alleles | e3/3 | Cognitive assessment test | Modified MMSE* (3MS) | | | Global cognitive decline (Decrease of 3MS by 5-10 points) | PM 2.5 | PM 2.5 > 12 ug/m^3^ vs. PM 2.5 < 12 ug/m^3^ | Hazard Ratio | - | - | 1.65 | 1.23 | 2.23 | - | 0.29 | NS |
|  |  |  | e3/4 |  |  |  |  |  |  |  |  |  |  | 1.93 | 1.29 | 2.9 |  |  |  |
|  |  |  | e4/4 |  |  |  |  |  |  |  |  |  |  | 3.64 | 1.36 | 9.69 |  |  |  |
|  |  |  | e3/3 |  |  |  |  | Diagnosis of Dementia |  |  |  |  |  | 1.68 | 0.97 | 2.92 |  | 0.43 | NS |
|  |  |  | e3/4 |  |  |  |  |  |  |  |  |  |  | 1.91 | 1.17 | 3.14 |  |  |  |
|  |  |  | e4/4 |  |  |  |  |  |  |  |  |  |  | 3.95 | 1.18 | 13.19 |  |  |  |
| Colicino et al., 2017 | | Telomere length | 1^st^ quintile | Cognitive assessment test | MMSE* | | | 25 ≥ MMSE* score | Black Carbon | Per 0.69 unit increase | Odds Ratio | - | - | 1.26 | 0.83 | 1.9 | Positive | 0.04 | S |
|  |  |  | 2^nd^ quintile |  |  |  |  |  |  |  |  |  |  | 1.45 | 0.96 | 2.19 |  |  |  |
|  |  |  | 3^rd^ quintile |  |  |  |  |  |  |  |  |  |  | 1.8 | 0.89 | 3.64 |  |  |  |
|  |  |  | 4^th^ quintile |  |  |  |  |  |  |  |  |  |  | 1.05 | 0.63 | 1.75 |  |  |  |
|  |  |  | 5^th^ quintile |  |  |  |  |  |  |  |  |  |  | 3.23 | 1.37 | 7.59 |  |  |  |
|  |  | C-reactive Protein | 1^st^ quintile |  |  |  |  |  |  |  |  |  |  | 0.77 | 0.35 | 1.71 | Positive | 0.04 | S |
|  |  |  | 2^nd^ quintile |  |  |  |  |  |  |  |  |  |  | 1.54 | 0.72 | 3.31 |  |  |  |
|  |  |  | 3^rd^ quintile |  |  |  |  |  |  |  |  |  |  | 1.02 | 0.35 | 2.96 |  |  |  |
|  |  |  | 4^th^ quintile |  |  |  |  |  |  |  |  |  |  | 0.7 | 0.24 | 2.04 |  |  |  |
|  |  |  | 5^th^ quintile |  |  |  |  |  |  |  |  |  |  | 2.68 | 1.06 | 6.79 |  |  |  |
| Cleary et al., 2018 | | APOE e4 | Non-carrier/carrier | Cognitive assessment test | MMSE, Cognitive Dementia Rating Sum of Boxes (CDR-SB) | | | Continuous score | Ozone | Per tertiles increase | Coefficient | - | - | - | - | - | - | NP | NS |
| Oudin et al., 2019† | | APOE e4 | Non-carrier | Diagnosis of VaD* using MMSE (Diagnostic and Statistical Manual of Mental Disorders, fourth edition criteria) | | | | Diagnosis of VaD* | NO_x_ | NOx (>9-17 ug/m^3^)  N | Hazard Ratio | - | - | 0.91 | 0.56 | 1.47 | - | 0.3> | NS† |
|  |  |  | carrier |  |  |  |  |  |  |  |  |  |  | 1.44 | 0.82 | 2.53 |  |  |  |
|  |  |  | Non-carrier |  |  |  |  |  |  | NOx (>17-26 ug/m^3^) |  |  |  | 1.48 | 0.97 | 2.27 |  |  |  |
|  |  |  | carrier |  |  |  |  |  |  |  |  |  |  | 1.49 | 0.86 | 2.59 |  |  |  |
|  |  |  | Non-carrier |  |  |  |  |  |  | NOx (>26 ug/m^3^) |  |  |  | 1.4 | 0.9 | 2.17 |  |  |  |
|  |  |  | carrier |  |  |  |  |  |  |  |  |  |  | 1.44 | 0.84 | 2.47 |  |  |  |
|  |  |  | Non-carrier | Diagnosis of AD* using MMSE (Diagnostic and Statistical Manual of Mental Disorders, fourth edition criteria) | | | | Diagnosis of AD* |  | NOx (>9-17 ug/m^3^)  N |  |  |  | 1.04 | 0.53 | 2.05 |  |  |  |
|  |  |  | carrier |  |  |  |  |  |  |  |  |  |  | 1.51 | 0.8 | 2.87 |  |  |  |
|  |  |  | Non-carrier |  |  |  |  |  |  | NOx (>17-26 ug/m^3^) |  |  |  | 1.79 | 0.99 | 3.24 |  |  |  |
|  |  |  | carrier |  |  |  |  |  |  |  |  |  |  | 1.61 | 0.86 | 3.01 |  |  |  |
|  |  |  | Non-carrier |  |  |  |  |  |  | NOx (>26 ug/m^3^) |  |  |  | 1.72 | 0.94 | 3.15 |  |  |  |
|  |  |  | carrier |  |  |  |  |  |  |  |  |  |  | 1.41 | 0.75 | 2.66 |  |  |  |
| Kulick et al., 2020 | | APOE E4 | Non-carrier | Cognitive assessment test | Global Cognitive Score (GCS) | | | Change in cognitive scores (Z score) | NO_2_ | Per IQR (11.2 ug/m^3^) increase | Coefficient | - | - | -0.069 | -0.09 | -0.05 | Positive | <0.001 | S |
|  |  |  | carrier |  |  |  |  |  |  |  |  |  |  | -0.085 | -0.11 | -0.06 |  |  |  |
|  |  |  | Non-carrier |  |  |  |  |  | PM 2.5 | Per IQR ( 4.42 ug/m^3^) increase |  |  |  | -0.088 | -0.12 | -0.06 | Positive | <0.001 | S |
|  |  |  | carrier |  |  |  |  |  |  |  |  |  |  | -0.104 | -0.13 | -0.08 |  |  |  |
|  |  |  | Non-carrier |  |  |  |  |  | PM 10 | Per IQR (7.95 ug/m^3^) increase |  |  |  | -0.039 | -0.06 | -0.02 | Positive | <0.001 | S |
|  |  |  | carrier |  |  |  |  |  |  |  |  |  |  | -0.057 | -0.07 | -0.04 |  |  |  |
|  |  |  | Non-carrier |  | Memory Domain | | |  | NO_2_ | Per IQR (11.2 ug/m^3^) increase |  |  |  | -0.033 | -0.06 | -0.01 | Positive | 0.03 | S |
|  |  |  | carrier |  |  |  |  |  |  |  |  |  |  | -0.043 | -0.07 | -0.02 |  |  |  |
|  |  |  | Non-carrier |  |  |  |  |  | PM 2.5 | Per IQR (4.42 ug/m^3^) increase |  |  |  | -0.043 | -0.07 | -0.01 | Positive | 0.02 | S |
|  |  |  | carrier |  |  |  |  |  |  |  |  |  |  | -0.053 | -0.09 | -0.02 |  |  |  |
|  |  |  | Non-carrier |  |  |  |  |  | PM 10 | Per IQR (7.95 ug/m^3^) increase |  |  |  | -0.018 | -0.04 | 0.001 | Positive | 0.02 | S |
|  |  |  | carrier |  |  |  |  |  |  |  |  |  |  | -0.029 | -0.05 | -0.01 |  |  |  |
|  |  |  | Non-carrier |  | Executive Function Domain | | |  | NO_2_ | Per IQR (11.2 ug/m^3^) increase |  |  |  | -0.025 | -0.05 | 0.001 | Positive | <0.001 | S |
|  |  |  | carrier |  |  |  |  |  |  |  |  |  |  | -0.04 | -0.07 | -0.01 |  |  |  |
|  |  |  | Non-carrier |  |  |  |  |  | PM 2.5 | Per IQR ( 4.42 ug/m^3^) increase |  |  |  | -0.047 | -0.08 | -0.02 | Positive | <0.01 | S |
|  |  |  | carrier |  |  |  |  |  |  |  |  |  |  | -0.061 | -0.09 | -0.03 |  |  |  |
|  |  |  | Non-carrier |  |  |  |  |  | PM 10 | Per IQR (7.95 ug/m^3^) increase |  |  |  | -0.018 | -0.04 | 0.003 | Positive | <0.001 | S |
|  |  |  | carrier |  |  |  |  |  |  |  |  |  |  | -0.033 | -0.05 | -0.01 |  |  |  |
|  |  |  | Non-carrier |  | Language Domain | | |  | NO_2_ | Per IQR (11.2 ug/m^3^) increase |  |  |  | -0.046 | -0.07 | -0.02 | Positive | <0.001 | S |
|  |  |  | carrier |  |  |  |  |  |  |  |  |  |  | -0.066 | -0.09 | -0.04 |  |  |  |
|  |  |  | Non-carrier |  |  |  |  |  | PM 2.5 | Per IQR ( 4.42 ug/m^3^) increase |  |  |  | -0.059 | -0.09 | -0.03 | Positive | <0.001 | S |
|  |  |  | carrier |  |  |  |  |  |  |  |  |  |  | -0.078 | -0.11 | -0.05 |  |  |  |
|  |  |  | Non-carrier |  |  |  |  |  | PM 10 | Per IQR (7.95 ug/m^3^) increase |  |  |  | -0.017 | -0.04 | 0.001 | Positive | 0.001 | S |
|  |  |  | carrier |  |  |  |  |  |  |  |  |  |  | -0.038 | -0.06 | -0.02 |  |  |  |
| Chen et al., 2020 | | APOE E4 | Non-carrier | Cognitive assessment test | MoCA-T* (Binary) | | | Score < 24 | PM2.5 | 2^nd^ tertile | Odds ratio | - | - | 4.54 | 0.49 | 42.04 | - | 0.27 | NS |
|  |  |  | Non-carrier |  |  |  |  |  |  | 3^rd^ tertile |  |  |  | 1.66 | 0.45 | 6.11 |  |  |  |
|  |  |  | carrier |  |  |  |  |  |  | 2^nd^ tertile |  |  |  | 6.86 | 0.24 | 196.8 |  | 0.41 |  |
|  |  |  | carrier |  |  |  |  |  |  | 3^rd^ tertile |  |  |  | 3.99 | 1.15 | 13.87 |  |  |  |
|  |  |  | Non-carrier |  | MoCA-T* (Continuous) | | | Continuous score | PM 2.5 | 2^nd^ tertile (= 29.00-29.98 ug/m^3^) | Coefficient |  |  | -0.41 | -1.72 | 0.9 |  | 0.86 |  |
|  |  |  | Non-carrier |  |  |  |  |  |  | 3^rd^ tertile (>29.98 ug/m^3^) |  |  |  | -0.11 | -0.63 | 0.41 |  |  |  |
|  |  |  | carrier |  |  |  |  |  |  | 2^nd^ tertile (= 29.00-29.98 ug/m^3^) |  |  |  | -1.2 | -2.49 | 0.1 |  | 0.55 |  |
|  |  |  | carrier |  |  |  |  |  |  | 3^rd^ tertile (>29.98 ug/m^3^) |  |  |  | -0.45 | -1 | 0.11 |  |  |  |
|  |  |  | Non-carrier |  | Trail making test | | |  | PM 10 | 2^nd^ tertile (=49.57 – 51.20 ug/m^3^) |  |  |  | -0.05 | -0.38 | 0.28 |  | 0.96 |  |
|  |  |  | Non-carrier |  |  |  |  |  |  | 3^rd^ tertile (> 51.20 ug/m^3^) |  |  |  | -0.11 | -0.27 | 0.04 |  |  |  |
|  |  |  | carrier |  |  |  |  |  |  | 2^nd^ tertile (=49.57 – 51.20 ug/m^3^) |  |  |  | -0.01 | -0.5 | 0.47 |  | 0.63 |  |
|  |  |  | carrier |  |  |  |  |  |  | 3^rd^ tertile (> 51.20 ug/m^3^) |  |  |  | -0.25 | -0.44 | -0.07 |  |  |  |
|  |  |  | Non-carrier |  | Verbal fluency | | |  | PM coarse | 2^nd^ tertile (= 22.45 – 26.50 ug/m^3^) |  |  |  | -0.15 | -0.52 | 0.21 |  | 0.82 |  |
|  |  |  | Non-carrier |  |  |  |  |  |  | 3^rd^ tertile (> 26.50 ug/m^3^) |  |  |  | -0.12 | -0.29 | 0.05 |  |  |  |
|  |  |  | carrier |  |  |  |  |  |  | 2^nd^ tertile (= 22.45 – 26.50 ug/m^3^) |  |  |  | 0.05 | -0.5 | 0.61 |  | 0.37 |  |
|  |  |  | carrier |  |  |  |  |  |  | 3^rd^ tertile (> 26.50 ug/m^3^) |  |  |  | -0.21 | -0.4 | -0.03 |  |  |  |
|  |  |  | Non-carrier |  |  |  |  |  | NO_2_ | 2^nd^ tertile (= 27.47 -28.62 ug/m^3^) |  |  |  | 0.19 | -0.12 | 0.5 |  | 0.42 |  |
|  |  |  | Non-carrier |  |  |  |  |  |  | 3^rd^ tertile (> 28.62 ug/m^3^) |  |  |  | 0.1 | -0.03 | 0.22 |  |  |  |
|  |  |  | carrier |  |  |  |  |  |  | 2^nd^ tertile (= 27.47 -28.62 ug/m^3^) |  |  |  | 0.08 | -0.39 | 0.54 |  | 0.65 |  |
|  |  |  | carrier |  |  |  |  |  |  | 3^rd^ tertile (> 28.62 ug/m^3^) |  |  |  | -0.03 | -0.22 | 0.16 |  |  |  |
| Crous-Bou et al., 2020 | | APOE e4 | Non-carrier | Neuroimaging | MRI | | | AD* signature for cortical thickness | NO_2_ | NP | Coefficient | - | - | -7.5 | NP | | - | 0.10 | NS |
|  |  |  | carrier |  |  |  |  |  |  |  |  |  |  | -25.3 |  |  |  |  |  |
|  |  |  | Non-carrier |  |  |  |  |  | NO_x_ |  |  |  |  | 1.07 |  |  | Qualitative | 0.04 | S |
|  |  |  | carrier |  |  |  |  |  |  |  |  |  |  | -69.3 |  |  |  |  |  |
|  |  |  | Non-carrier |  |  |  |  |  | PM 2.5 |  |  |  |  | -2.07 |  |  | - | 0.37 | NS |
|  |  |  | carrier |  |  |  |  |  |  |  |  |  |  | 1.88 |  |  |  |  |  |
|  |  |  | Non-carrier |  |  |  |  |  | PM 10 |  |  |  |  | 0.31 |  |  | Positive | 0.04 | S |
|  |  |  | carrier |  |  |  |  |  |  |  |  |  |  | 3.69 |  |  |  |  |  |
|  |  |  | Non-carrier |  |  |  |  |  | PM 2.5 abs |  |  |  |  | -0.36 |  |  | - | 0.17 | NS |
|  |  |  | carrier |  |  |  |  |  |  |  |  |  |  | 0.56 |  |  |  |  |  |
|  |  |  | Non-carrier |  |  |  |  |  | PM coarse |  |  |  |  | -1.27 |  |  | - | 0.32 | NS |
|  |  |  | carrier |  |  |  |  |  |  |  |  |  |  | 1.83 |  |  |  |  |  |
| Alemany et al. 2021 | | APOE-e4 carrier | Carrier |  | CSF Aβ42/40 | | |  | NO2 | Per 1 standard deviation (10.9 ug/m^3^) increase |  |  |  | -0.11 |  |  | - | 0.844 | NS |
|  |  |  | Noncarrier |  |  |  |  |  |  |  |  |  |  | -0.04 |  |  |  |  |  |
|  |  |  | Carrier |  |  |  |  |  | PM2.5 | Per 1 standard deviation (2.2 ug/m^3^) increase |  |  |  | -0.10 |  |  | - | 0.626 | NS |
|  |  |  | Noncarrier |  |  |  |  |  |  |  |  |  |  | -0.01 |  |  |  |  |  |
|  |  |  | Carrier |  |  |  |  |  | PM10 | Per 1 standard deviation (4.1 ug/m^3^) increase |  |  |  | -0.04 |  |  | - | 0.959 | NS |
|  |  |  | Noncarrier |  |  |  |  |  |  |  |  |  |  | -0.06 |  |  |  |  |  |
|  |  |  | Carrier |  |  |  |  |  | PM2.5 abs | Per 1 standard deviation (0.6 ug/m^3^) increase |  |  |  | -0.11 |  |  | - | 0.645 | NS |
|  |  |  | Noncarrier |  |  |  |  |  |  |  |  |  |  | -0.01 |  |  |  |  |  |
|  |  |  | Carrier |  | phosphorylated tau (p-tau) | | |  | NO2 | Per 1 standard deviation (10.9 ug/m^3^) increase |  |  |  | 0.19 |  |  | - | 0.231 | NS |
|  |  |  | Noncarrier |  |  |  |  |  |  |  |  |  |  | -0.03 |  |  |  |  |  |
|  |  |  | Carrier |  |  |  |  |  | PM2.5 | Per 1 standard deviation (2.2 ug/m^3^) increase |  |  |  | 0.04 |  |  | - | 0.521 | NS |
|  |  |  | Noncarrier |  |  |  |  |  |  |  |  |  |  | 0.16 |  |  |  |  |  |
|  |  |  | Carrier |  |  |  |  |  | PM10 | Per 1 standard deviation (4.1 ug/m^3^) increase |  |  |  | 0.14 |  |  | - | 0.342 | NS |
|  |  |  | Noncarrier |  |  |  |  |  |  |  |  |  |  | -0.02 |  |  |  |  |  |
|  |  |  | Carrier |  |  |  |  |  | PM2.5 abs | Per 1 standard deviation (0.6 ug/m^3^) increase |  |  |  | 0.22 |  |  | - | 0.241 | NS |
|  |  |  | Noncarrier |  |  |  |  |  |  |  |  |  |  | 0.03 |  |  |  |  |  |
|  |  |  | Carrier |  | total tau (t-tau) | | |  | NO2 | Per 1 standard deviation (10.9 ug/m^3^) increase |  |  |  | 0.19 |  |  | - | 0.344 | NS |
|  |  |  | Noncarrier |  |  |  |  |  |  |  |  |  |  | 0.00 |  |  |  |  |  |
|  |  |  | Carrier |  |  |  |  |  | PM2.5 | Per 1 standard deviation (2.2 ug/m^3^) increase |  |  |  | 0.05 |  |  | - | 0.550 | NS |
|  |  |  | Noncarrier |  |  |  |  |  |  |  |  |  |  | 0.15 |  |  |  |  |  |
|  |  |  | Carrier |  |  |  |  |  | PM10 | Per 1 standard deviation (4.1 ug/m^3^) increase |  |  |  | 0.12 |  |  | - | 0.512 | NS |
|  |  |  | Noncarrier |  |  |  |  |  |  |  |  |  |  | 0.01 |  |  |  |  |  |
|  |  |  | Carrier |  |  |  |  |  | PM2.5 abs | Per 1 standard deviation (0.6 ug/m^3^) increase |  |  |  | 0.24 |  |  | - | 0.255 | NS |
|  |  |  | Noncarrier |  |  |  |  |  |  |  |  |  |  | 0.04 |  |  |  |  |  |
|  |  |  | Carrier |  | neurofilament light (NfL) | | |  | NO2 | Per 1 standard deviation (10.9 ug/m^3^) increase |  |  |  | 0.25 |  |  | - | 0.142 | NS |
|  |  |  | Noncarrier |  |  |  |  |  |  |  |  |  |  | 0.02 |  |  |  |  |  |
|  |  |  | Carrier |  |  |  |  |  | PM2.5 | Per 1 standard deviation (2.2 ug/m^3^) increase |  |  |  | 0.14 |  |  | - | 0.972 | NS |
|  |  |  | Noncarrier |  |  |  |  |  |  |  |  |  |  | 0.19 |  |  |  |  |  |
|  |  |  | Carrier |  |  |  |  |  | PM10 | Per 1 standard deviation (4.1 ug/m^3^) increase |  |  |  | 0.25 |  |  | - | 0.213 | NS |
|  |  |  | Noncarrier |  |  |  |  |  |  |  |  |  |  | 0.06 |  |  |  |  |  |
|  |  |  | Carrier |  |  |  |  |  | PM2.5 abs | Per 1 standard deviation (0.6 ug/m^3^) increase |  |  |  | 0.24 |  |  | - | 0.249 | NS |
|  |  |  | Noncarrier |  |  |  |  |  |  |  |  |  |  | 0.08 |  |  |  |  |  |
|  |  |  | Carrier |  | Centiloid values (CL) | | |  | NO2 | Per 1 standard deviation (10.9 ug/m^3^) increase |  |  |  | 0.16 |  |  | - | 0.938 | NS |
|  |  |  | Noncarrier |  |  |  |  |  |  |  |  |  |  | 0.26 |  |  |  |  |  |
|  |  |  | Carrier |  |  |  |  |  | PM2.5 | Per 1 standard deviation (2.2 ug/m^3^) increase |  |  |  | 0.02 |  |  | - | 0.360 | NS |
|  |  |  | Noncarrier |  |  |  |  |  |  |  |  |  |  | 0.25 |  |  |  |  |  |
|  |  |  | Carrier |  |  |  |  |  | PM10 | Per 1 standard deviation (4.1 ug/m^3^) increase |  |  |  | 0.08 |  |  | - | 0.760 | NS |
|  |  |  | Noncarrier |  |  |  |  |  |  |  |  |  |  | 0.20 |  |  |  |  |  |
|  |  |  | Carrier |  |  |  |  |  | PM2.5 abs | Per 1 standard deviation (0.6 ug/m^3^) increase |  |  |  | 0.04 |  |  | - | 0.345 | NS |
|  |  |  | Noncarrier |  |  |  |  |  |  |  |  |  |  | 0.31 |  |  |  |  |  |
| Mortamais et al., 2021 | | APOE e4 | Not-carrier | 3-step procedure | All cause dementia, AD, Vascular/mixed dementia (VaD) | | | Diagnosis of all cause dementia, AD, VaD | PM2.5 | Per 5 μg/m3 increase | Hazard ratio | - | | NP | NP | NP | NP (not modified by APOE e4 status) | NP | NS |
|  |  |  | Carriers |  |  |  |  |  |  |  |  |  |  |  |  |  |  |  |  |
| Shaffer et al., 2021 | | APOE E4 | Non-carrier | Health administrative data (Medical records review) | | | | Diagnosis of dementia | PM 2.5 | Per 1 μg/m^3^ increase | Hazard ratio | - | - | 1.14 | 1.01 | 1.29 | - | 0.1 | NS |
|  |  |  | carrier |  |  |  |  |  |  |  |  |  |  | 1.2 | 1.06 | 1.36 |  |  |  |
| M. Li et al., 2022 | | APOE e4 | Non-carrier | CSF sTREM2 score | Neuroinflammatory (a biomarker of AD, represented by CSF sTREM2 score) | | | Continuous score | PM2.5 | NP | Z-score | - | - | -0.115 | NP | NP | Positive | 0.0006 | S |
|  |  |  | Carrier |  |  |  |  |  |  |  |  |  |  | -0.124 |  |  |  | 0.1095 |  |
| Parra et al., 2022 | | APOE e4 | 0 e4 allele | Health administrative data  (coded with ICD-9* or ICD-10*) | | | | Diagnosis of All-cause dementia | PM 2.5 | Per IQR (1.25 ug/m^3^) increase | Hazard Ratio | - | - | 1.18 | 1.08 | 1.28 | - | 0.928 | NS |
|  |  |  | 1 e4 allele |  |  |  |  |  |  |  |  |  |  | 1.14 | 1.03 | 1.25 |  |  |  |
|  |  |  | 2 e4 allele |  |  |  |  |  |  |  |  |  |  | 1.25 | 1.05 | 1.49 |  |  |  |
|  |  |  | 0 e4 allele |  |  |  |  |  | PM2.5 absorb | Per IQR (0.29 ug/m^3^) increase |  |  |  | 1.07 | 0.99 | 1.15 |  | 0.557 | NS |
|  |  |  | 1 e4 allele |  |  |  |  |  |  |  |  |  |  | 1.08 | 0.99 | 1.18 |  |  |  |
|  |  |  | 2 e4 allele |  |  |  |  |  |  |  |  |  |  | 1.15 | 1.00 | 1.33 |  |  |  |
|  |  |  | 0 e4 allele |  |  |  |  |  | PM 2.5-10 | Per IQR (0.76 ug/m^3^) increase |  |  |  | 1.98 | 0.92 | 1.04 |  | 0.853 | NS |
|  |  |  | 1 e4 allele |  |  |  |  |  |  |  |  |  |  | 1.03 | 0.96 | 1.09 |  |  |  |
|  |  |  | 2 e4 allele |  |  |  |  |  |  |  |  |  |  | 0.96 | 0.84 | 1.09 |  |  |  |
|  |  |  | 0 e4 allele |  |  |  |  |  | PM 10 | Per IQR (1.74 ug/m^3^) increase |  |  |  | 1.02 | 0.96 | 1.09 |  | 0.916 | NS |
|  |  |  | 1 e4 allele |  |  |  |  |  |  |  |  |  |  | 1.04 | 0.97 | 1.12 |  |  |  |
|  |  |  | 2 e4 allele |  |  |  |  |  |  |  |  |  |  | 1.01 | 0.89 | 1.16 |  |  |  |
|  |  |  | 0 e4 allele |  |  |  |  |  | NO_2_ | Per IQR (9.39 ug/m^3^) increase |  |  |  | 1.15 | 1.05 | 1.26 |  | 0.527 | NS |
|  |  |  | 1 e4 allele |  |  |  |  |  |  |  |  |  |  | 1.18 | 1.07 | 1.31 |  |  |  |
|  |  |  | 2 e4 allele |  |  |  |  |  |  |  |  |  |  | 1.26 | 1.05 | 1.52 |  |  |  |
|  |  |  | 0 e4 allele |  |  |  |  |  | NO_x_ | Per IQR (16.02 ug/m^3^) increase |  |  |  | 1.11 | 1.04 | 1.18 |  | 0.801 | NS |
|  |  |  | 1 e4 allel |  |  |  |  |  |  |  |  |  |  | 1.11 | 1.03 | 1.2 |  |  |  |
|  |  |  | 2 e4 allele |  |  |  |  |  |  |  |  |  |  | 1.15 | 1 | 1.33 |  |  |  |
|  |  |  | 0 e4 allele |  |  |  |  | Incident AD* | PM 2.5 | Per IQR (1.25 ug/m^3^) increase |  |  |  | 1.26 | 1.08 | 1.46 |  | 0.177 | NS |
|  |  |  | 1 e4 allele |  |  |  |  |  |  |  |  |  |  | 1.14 | 0.99 | 1.32 |  |  |  |
|  |  |  | 2 e4 allele |  |  |  |  |  |  |  |  |  |  | 1.03 | 0.8 | 1.32 |  |  |  |
|  |  |  | 0 e4 allele |  |  |  |  |  | PM2.5 absorb | Per IQR (0.29 ug/m^3^) increase |  |  |  | 1.13 | 0.99 | 1.29 |  | 0.413 | NS |
|  |  |  | 1 e4 allele |  |  |  |  |  |  |  |  |  |  | 1.04 | 0.92 | 1.19 |  |  |  |
|  |  |  | 2 e4 allele |  |  |  |  |  |  |  |  |  |  | 1.05 | 0.85 | 1.29 |  |  |  |
|  |  |  | 0 e4 allele |  |  |  |  |  | PM 2.5-10 | Per IQR (0.76 ug/m^3^) increase |  |  |  | 0.99 | 0.88 | 1.1 |  | 0.78 | NS |
|  |  |  | 1 e4 allele |  |  |  |  |  |  |  |  |  |  | 1.06 | 0.97 | 1.17 |  |  |  |
|  |  |  | 2 e4 allele |  |  |  |  |  |  |  |  |  |  | 0.92 | 0.76 | 1.1 |  |  |  |
|  |  |  | 0 e4 allele |  |  |  |  |  | PM 10 | Per IQR (1.74 ug/m^3^) increase |  |  |  | 1.05 | 0.93 | 1.19 |  | 0.623 | NS |
|  |  |  | 1 e4 allele |  |  |  |  |  |  |  |  |  |  | 1.09 | 0.98 | 1.21 |  |  |  |
|  |  |  | 2 e4 allele |  |  |  |  |  |  |  |  |  |  | 0.96 | 0.8 | 1.16 |  |  |  |
|  |  |  | 0 e4 allele |  |  |  |  |  | NO_2_ | Per IQR (9.39 ug/m^3^) increase |  |  |  | 1.22 | 1.03 | 1.43 |  | 0.384 | NS |
|  |  |  | 1 e4 allele |  |  |  |  |  |  |  |  |  |  | 1.13 | 0.97 | 1.32 |  |  |  |
|  |  |  | 2 e4 allele |  |  |  |  |  |  |  |  |  |  | 1.07 | 0.82 | 1.4 |  |  |  |
|  |  |  | 0 e4 allele |  |  |  |  |  | NO_x_ | Per IQR (16.02 ug/m^3^) increase |  |  |  | 1.16 | 1.03 | 1.29 |  | 0.362 | NS |
|  |  |  | 1 e4 allele |  |  |  |  |  |  |  |  |  |  | 1.08 | 0.96 | 1.22 |  |  |  |
|  |  |  | 2 e4 allele |  |  |  |  |  |  |  |  |  |  | 1.06 | 0.85 | 1.31 |  |  |  |
| Semmens et al., 2022 | | APOE e4 | Non-carrier/carrier | Neuroimaging | | MRI | | Incident dementia | PM2.5, NO_2_ | Per IQR increase | Hazard Ratio | - | - | NP | NP | NP | - | NP | NS |
| Ma et al., 2022 | | APOE e4 | 0 e4 allele | Health administrative data  (coded with ICD-9* or ICD-10*) | | | | Diagnosis of incident dementia | NO_2_ | 2^nd^ tertile | Hazard Ratio | - | - | 1.15 | 0.94 | 1.41 | - | 0.43 | NS |
|  |  |  |  |  |  |  |  |  |  | 3^rd^ tertile |  |  |  | 1.47 | 1.19 | 1.82 |  |  |  |
|  |  |  | At least 1 e4 allele |  |  |  |  |  |  | 1^st^ tertile |  |  |  | 3.37 | 2.77 | 4.09 |  |  |  |
|  |  |  |  |  |  |  |  |  |  | 2^nd^ tertile |  |  |  | 3.22 | 2.64 | 3.93 |  |  |  |
|  |  |  |  |  |  |  |  |  |  | 3^rd^ tertile |  |  |  | 4.54 | 3.69 | 5.60 |  |  |  |
|  |  |  | 0 e4 allele |  |  |  |  |  | PM 2.5 | 2^nd^ tertile |  |  |  | 1.19 | 0.98 | 1.46 |  | 0.63 | NS |
|  |  |  |  |  |  |  |  |  |  | 3^rd^ tertile |  |  |  | 1.25 | 1.02 | 1.53 |  |  |  |
|  |  |  | At least 1 e4 allele |  |  |  |  |  |  | 1^st^ tertile |  |  |  | 3.31 | 2.72 | 4.04 |  |  |  |
|  |  |  |  |  |  |  |  |  |  | 2^nd^ tertile |  |  |  | 3.48 | 2.86 | 4.24 |  |  |  |
|  |  |  |  |  |  |  |  |  |  | 3^rd^ tertile |  |  |  | 3.78 | 3.10 | 4.61 |  |  |  |
|  |  | Polygenetic risk score | 0 e4 allele |  |  |  |  |  | NO_2_ | 2^nd^ tertile |  |  |  | 1.13 | 0.91 | 1.40 |  | 0.47 | NS |
|  |  |  |  |  |  |  |  |  |  | 3^rd^ tertile |  |  |  | 1.50 | 1.20 | 1.87 |  |  |  |
|  |  |  | At least 1 e4 allele |  |  |  |  |  |  | 1^st^ tertile |  |  |  | 3.07 | 2.51 | 3.74 |  |  |  |
|  |  |  |  |  |  |  |  |  |  | 2^nd^ tertile |  |  |  | 3.02 | 2.47 | 3.69 |  |  |  |
|  |  |  |  |  |  |  |  |  |  | 3^rd^ tertile |  |  |  | 4.08 | 3.30 | 5.04 |  |  |  |
|  |  |  | 0 e4 allele |  |  |  |  |  | PM 2.5 | 2^nd^ tertile |  |  |  | 1.16 | 0.94 | 1.43 |  | 0.95 | NS |
|  |  |  |  |  |  |  |  |  |  | 3^rd^ tertile |  |  |  | 1.19 | 0.96 | 1.47 |  |  |  |
|  |  |  | At least 1 e4 allele |  |  |  |  |  |  | 1^st^ tertile |  |  |  | 2.88 | 2.35 | 3.51 |  |  |  |
|  |  |  |  |  |  |  |  |  |  | 2^nd^ tertile |  |  |  | 3.11 | 2.55 | 3.78 |  |  |  |
|  |  |  |  |  |  |  |  |  |  | 3^rd^ tertile |  |  |  | 3.40 | 2.79 | 4.15 |  |  |  |
| Wang et al., 2022 | | APOE E4 | Non-carrier | Telephone interview for cognitive status (TICSm) | | | | Diagnosis of incident dementia | PM 2.5 | Per IQR (1.78 ug/m^3^) increase | Hazard Ratio | - | - | 0.74 | 0.62 | 0.89 | - | 0.67 | NS |
|  |  |  | carrier |  |  |  |  |  |  |  |  |  |  | 0.79 | 0.63 | 0.98 |  |  |  |
|  |  |  | Non-carrier |  |  |  |  |  | NO_2_ | Per IQR (3.91 ppb) increase |  |  |  | 0.75 | 0.64 | 0.89 |  | 0.96 | NS |
|  |  |  | carrier |  |  |  |  |  |  |  |  |  |  | 0.76 | 0.59 | 0.97 |  |  |  |
| Decrom et al., 2022 | | APOE E4 | Non-carrier | DSM-III-R*, NINCDS-ADRDA* | | | | Diagnosis of incident dementia | Not specified | Per IQR (3.91 ppb) increase | Hazard Ratio | - | - | 1.14 | 0.95 | 1.16 | - | NP | NS |
|  |  |  | carrier |  |  |  |  |  |  |  |  |  |  | 0.98 | 0.85 | 1.21 |  |  |  |
| G.-C. Chen et al., 2022 | | APOE E4 | Non-carrier | Health administrative data  (coded with ICD-9* or ICD-10*) | | | | Incident all-cause dementia | Air pollution score | Per IQR increase | Hazard Ratio | - | - | 1.12 | 1.07 | 1.17 | - | 0.37 | NS |
|  |  |  | carrier |  |  |  |  |  |  |  |  |  |  | 1.1 | 1.05 | 1.16 |  |  |  |
|  |  |  | Non-carrier |  |  |  |  | Incident Alzheimer’s disease |  |  |  |  |  | 1.14 | 1.07 | 1.22 |  | 0.18 | NS |
|  |  |  | carrier |  |  |  |  |  |  |  |  |  |  | 1.12 | 1.04 | 1.2 |  |  |  |
|  |  |  | Non-carrier |  |  |  |  | Incident Vascular dementia |  |  |  |  |  | 1.09 | 1.00 | 1.19 |  | 0.86 | NS |
|  |  |  | carrier |  |  |  |  |  |  |  |  |  |  | 1.14 | 1.03 | 1.26 |  |  |  |
| Zhang et al., 2023 | | Pollygenic risk score | 1^st^ tertile | Health administrative data  (coded with ICD-9* or ICD-10*) | | | | Diagnosis of all-cause dementia | PM 2.5 | Per IQR (1.3 ug/m^3^) increase | Hazard Ratio | - | - | 0.974 | 0.876 | 1.082 | - | 0.65 | NS |
|  |  |  | 2^nd^ tertile |  |  |  |  |  |  |  |  |  |  | 1.049 | 0.958 | 1.148 |  |  |  |
|  |  |  | 3^rd^ tertile |  |  |  |  |  |  |  |  |  |  | 1.003 | 0.938 | 1.073 |  |  |  |
|  |  |  | 1^st^ tertile |  |  |  |  |  | PM coarse | Per IQR (0.8 ug/m^3^) increase |  |  |  | 0.955 | 0.898 | 1.017 |  | 0.80 |  |
|  |  |  | 2^nd^ tertile |  |  |  |  |  |  |  |  |  |  | 0.997 | 0.946 | 1.05 |  |  |  |
|  |  |  | 3^rd^ tertile |  |  |  |  |  |  |  |  |  |  | 0.978 | 0.94 | 1.017 |  |  |  |
|  |  |  | 1^st^ tertile |  |  |  |  |  | PM 10 | Per IQR (2.3 ug/m^3^) increase |  |  |  | 1.113 | 0.998 | 1.241 |  | 0.14 |  |
|  |  |  | 2^nd^ tertile |  |  |  |  |  |  |  |  |  |  | 1.114 | 1.017 | 1.22 |  |  |  |
|  |  |  | 3^rd^ tertile |  |  |  |  |  |  |  |  |  |  | 1.054 | 0.984 | 1.129 |  |  |  |
|  |  |  | 1^st^ tertile |  |  |  |  |  | PM 2.5 absorb | Per IQR (0.3 ug/m^3^) increase |  |  |  | 1.007 | 0.918 | 1.105 |  | 0.91 |  |
|  |  |  | 2^nd^ tertile |  |  |  |  |  |  |  |  |  |  | 1.047 | 0.969 | 1.131 |  |  |  |
|  |  |  | 3^rd^ tertile |  |  |  |  |  |  |  |  |  |  | 1.058 | 0.997 | 1.123 |  |  |  |
|  |  |  | 1^st^ tertile |  |  |  |  |  | NO_2_ | Per IQR (10.5 ug/m^3^) increase |  |  |  | 1.166 | 1.041 | 1.306 |  | 0.36 |  |
|  |  |  | 2^nd^ tertile |  |  |  |  |  |  |  |  |  |  | 1.115 | 1.0174 | 1.223 |  |  |  |
|  |  |  | 3^rd^ tertile |  |  |  |  |  |  |  |  |  |  | 1.141 | 1.062 | 1.225 |  |  |  |
|  |  |  | 1^st^ tertile |  |  |  |  |  | NO | Per IQR (16.1 ug/m^3^) increase |  |  |  | 0.971 | 0.888 | 1.061 |  | 0.91 |  |
|  |  |  | 2^nd^ tertile |  |  |  |  |  |  |  |  |  |  | 1.067 | 0.99 | 1.151 |  |  |  |
|  |  |  | 3^rd^ tertile |  |  |  |  |  |  |  |  |  |  | 1.02 | 0.964 | 1.08 |  |  |  |
|  |  |  | 1^st^ tertile |  |  |  |  | Diagnosis of incident AD* | PM 2.5 | Per IQR (1.3 ug/m^3^) increase |  |  |  | 1.024 | 0.83 | 1.264 |  | 0.16 |  |
|  |  |  | 2^nd^ tertile |  |  |  |  |  |  |  |  |  |  | 1.117 | 0.95 | 1.313 |  |  |  |
|  |  |  | 3^rd^ tertile |  |  |  |  |  |  |  |  |  |  | 1.007 | 0.9 | 1.125 |  |  |  |
|  |  |  | 1^st^ tertile |  |  |  |  |  | PM coarse | Per IQR (0.8 ug/m^3^) increase |  |  |  | 0.919 | 0.804 | 1.051 |  | 0.31 |  |
|  |  |  | 2^nd^ tertile |  |  |  |  |  |  |  |  |  |  | 1.048 | 0.957 | 1.149 |  |  |  |
|  |  |  | 3^rd^ tertile |  |  |  |  |  |  |  |  |  |  | 1.022 | 0.961 | 1.088 |  |  |  |
|  |  |  | 1^st^ tertile |  |  |  |  |  | PM 10 | Per IQR (2.3 ug/m^3^) increase |  |  |  | 1 | 0.797 | 1.225 |  | 0.74 |  |
|  |  |  | 2^nd^ tertile |  |  |  |  |  |  |  |  |  |  | 1.158 | 0.982 | 1.365 |  |  |  |
|  |  |  | 3^rd^ tertile |  |  |  |  |  |  |  |  |  |  | 1.079 | 0.964 | 1.208 |  |  |  |
|  |  |  | 1^st^ tertile |  |  |  |  |  | PM 2.5 absorb | Per IQR (0.3 ug/m^3^) increase |  |  |  | 1.03 | 0.854 | 1.242 |  | 0.72 |  |
|  |  |  | 2^nd^ tertile |  |  |  |  |  |  |  |  |  |  | 1.138 | 0.991 | 1.306 |  |  |  |
|  |  |  | 3^rd^ tertile |  |  |  |  |  |  |  |  |  |  | 1.127 | 1.022 | 1.242 |  |  |  |
|  |  |  | 1^st^ tertile |  |  |  |  |  | NO_2_ | Per IQR (10.5 ug/m^3^) increase |  |  |  | 1.082 | 0.861 | 1.36 |  | 0.54 |  |
|  |  |  | 2^nd^ tertile |  |  |  |  |  |  |  |  |  |  | 1.148 | 0.963 | 1.369 |  |  |  |
|  |  |  | 3^rd^ tertile |  |  |  |  |  |  |  |  |  |  | 1.185 | 1.052 | 1.335 |  |  |  |
|  |  |  | 1^st^ tertile |  |  |  |  |  | NO | Per IQR (16.1 ug/m^3^) increase |  |  |  | 1.023 | 0.863 | 1.213 |  | 0.33 |  |
|  |  |  | 2^nd^ tertile |  |  |  |  |  |  |  |  |  |  | 1.12 | 0.977 | 1.284 |  |  |  |
|  |  |  | 3^rd^ tertile |  |  |  |  |  |  |  |  |  |  | 1.047 | 0.954 | 1.148 |  |  |  |
|  |  |  | 1^st^ tertile |  |  |  |  | Diagnosis of VaD* | PM 2.5 | Per IQR (1.3 ug/m^3^) increase |  |  |  | 0.853 | 0.648 | 1.122 |  | 0.65 |  |
|  |  |  | 2^nd^ tertile |  |  |  |  |  |  |  |  |  |  | 0.816 | 0.647 | 1.028 |  |  |  |
|  |  |  | 3^rd^ tertile |  |  |  |  |  |  |  |  |  |  | 1.044 | 0.87 | 1.253 |  |  |  |
|  |  |  | 1^st^ tertile |  |  |  |  |  | PM coarse | Per IQR (0.8 ug/m^3^) increase |  |  |  | 0.985 | 0.848 | 1.145 |  | 0.80 |  |
|  |  |  | 2^nd^ tertile |  |  |  |  |  |  |  |  |  |  | 0.92 | 0.797 | 1.062 |  |  |  |
|  |  |  | 3^rd^ tertile |  |  |  |  |  |  |  |  |  |  | 0.987 | 0.888 | 1.097 |  |  |  |
|  |  |  | 1^st^ tertile |  |  |  |  |  | PM 10 | Per IQR (2.3 ug/m^3^) increase |  |  |  | 1.201 | 0.909 | 1.586 |  | 0.14 |  |
|  |  |  | 2^nd^ tertile |  |  |  |  |  |  |  |  |  |  | 1.102 | 0.878 | 1.384 |  |  |  |
|  |  |  | 3^rd^ tertile |  |  |  |  |  |  |  |  |  |  | 1.054 | 0.874 | 1.273 |  |  |  |
|  |  |  | 1^st^ tertile |  |  |  |  |  | PM 2.5 absorb | Per IQR (0.3 ug/m^3^) increase |  |  |  | 1.145 | 0.918 | 1.428 |  | 0.91 |  |
|  |  |  | 2^nd^ tertile |  |  |  |  |  |  |  |  |  |  | 1.009 | 0.829 | 1.227 |  |  |  |
|  |  |  | 3^rd^ tertile |  |  |  |  |  |  |  |  |  |  | 1.017 | 0.864 | 1.197 |  |  |  |
|  |  |  | 1^st^ tertile |  |  |  |  |  | NO_2_ | Per IQR (10.5 ug/m^3^) increase |  |  |  | 1.307 | 0.986 | 1.731 |  | 0.36 |  |
|  |  |  | 2^nd^ tertile |  |  |  |  |  |  |  |  |  |  | 1.101 | 0.876 | 1.383 |  |  |  |
|  |  |  | 3^rd^ tertile |  |  |  |  |  |  |  |  |  |  | 1.048 | 0.856 | 1.283 |  |  |  |
|  |  |  | 1^st^ tertile |  |  |  |  |  | NO | Per IQR (16.1 ug/m^3^) increase |  |  |  | 1.107 | 0.826 | 1.252 |  | 0.91 |  |
|  |  |  | 2^nd^ tertile |  |  |  |  |  |  |  |  |  |  | 0.947 | 0.776 | 1.156 |  |  |  |
|  |  |  | 3^rd^ tertile |  |  |  |  |  |  |  |  |  |  | 0.996 | 0.853 | 1.162 |  |  |  |
|  | * AD: Alzheimer's Disease; CCFM: Chicago Cognitive Function Measure; MRI: Magnetic Resonance Imaging; MMSE: Mini-Mental State Examination; WM: White Matter; IADL: Impaired Activities of Daily Living; HbA1c: Glycosylated hemoglobin; CRP: C-reactive Protein; CVD: Cardiovascular Disease; SEVLT: Spanish English Verbal Learning Test; DSMMD: Diagnostic and Statistical Manual of Mental Disorders; 3WMT: Three-word memory test; SVF: Semantic Verbal fluency; MCI: Mild Cognitive Function; HV: Hippocampal Volumes; DADL: Disability in activities of daily living; HFE: Hemochromatosis gene; VaD: Vascular Alzheimer’s Disease; NINCDS-ADRDA: National Institute of Neurological and Communicative Diseases and Stroke/Alzheimer’s Disease and Related Disorders Association; NINDS-AIREN: National Institute of Neurological Disorders and Stroke and the Association Internationale pour la Recherche et I’Enseignement en Neurosicenes; CERAD: The Consortium to Establish a Registry for Alzheimer’s Disease; SNPs: Single Nucleotide Polymorphism  ** CI: Confidence Interval; S/NS: Significance/Non-Significance; IQR: Inter-Quartile Range; RD: Residential Distance; NP: Not provided  *** The total effect size (A+C) was manually calculated unless the effect of air pollution exposure on each category of the effect modifier was provided in the studies  **** 95% CI is based on coefficient of effect modifiers  *****Higher amount of CSF biomarker indicated more pathological level (Alemany et al., 2021)  † Oudin et al., 2019: an evidence of effect modification by APOE e4 was non-significant (0>0.3) indicated by non-significant interaction terms mentioned in text.  ※ 5 studies tested APOE e4 as an effect modifier but either 1) did not provide effect size measures in the tables, but tested and mentioned non significance (Mortamais et al., 2021) or significance based on interaction term using APOE e4 (Cleary et al., 2018) 2) only provided graphics without the measures (Semmens et al., 2022; Tzivian, Dlugaj, Winkler, Hennig, et al., 2016) | | | | | | | | | | | | | | | | | | |

- Table E13: Effect size by potential effect modifier – Physical Activity (2/7)

| **Study** | **Effect Modifier** | | **Outcome** | | | **Air**  **Pollutant** | **Exposure**  **Level** | **Types of Effect** | **Effect**  **Size**  **(A: Main Effect by exposure)** | **Effect Size**  **(C: Effect by interaction term)** | **Effect Size  Estimate**  **(A+C)** | **95% CI*s** | | **Direction of Modifier Measure** | **p-value  for  interaction terms** | **Statistical Significance (S/NS**)** |
| --- | --- | --- | --- | --- | --- | --- | --- | --- | --- | --- | --- | --- | --- | --- | --- | --- |
|  | **Physical Activity** | | **Outcome Measure Method** | | **Outcome Variable** |  |  |  |  |  |  | **lower** | **upper** |  |  |  |
| Tallon et al., 2017 | Physical activity | None | Cognitive assessment test | CCFM* | CCFM Score | PM2.5 | per IQR | Coefficient | - | - | -0.21 | -0.43 | 0.01 | - | 0.73 | NS |
|  |  | ≥1/a week |  |  |  |  |  |  |  |  | -0.2 | -0.67 | 0.27 |  |  |  |
|  |  | None |  |  |  | NO2 |  |  |  |  | -0.12 | -0.33 | 0.09 |  | 0.63 | NS |
|  |  | ≥1/a week |  |  |  |  |  |  |  |  | -1.19 | -1.66 | -0.71 |  |  |  |
| Cullen et al., 2018 | Time spent outdoor | | Cognitive assessment test | | Cognitive score change | PM2.5, PM2.5-10, PM10, NOx | - | Coefficient | - | - | NP** | NP | NP | - | NP | NS |
| Shin et al., 2019 | Physical activity | Inactive/active | Cognitive assessment test (Word list recognition) | Korean MMSE | Cognitive score | PM2.5 | Per IQR increase | Coefficients | - | - | NP | NP | NP | Negative | NP | S |
| Wang et al., 2020 | Regular exercise | No | Cognitive assessment test | Chinese MMSE | Poor cognitive function | PM2.5 | per 10 μg/m3 | Hazard Ratio | - | - | 1.057 | 1.008 | 1.11 | - | 0.82 | NS |
|  |  | Yes |  |  |  |  |  |  |  |  | 1.049 | 1.015 | 1.084 |  |  |  |
| Yao et al., 2021 | Physical activity | No | Cognitive assessment test | Chinese MMSE | Cognitive impairment | RD** to main roadway  ( ≤100m vs. >100m) | NP | Odds Ratio | - | - | 1.15 | 1.01 | 1.31 |  | 0.53 | NS |
|  |  | Yes |  |  |  |  |  |  |  |  | 1.31 | 1.11 | 1.54 | - |  |  |
| Gao et al., 2022 | Physical activity | No | Cognitive assessment test | Chinese MMSE | Cognitive impairment | Ozone | per 10 μg/m3 | Hazard Ratio | - | - | 1.091 | 1.034 | 1.257 |  | 0.416 | NS |
|  |  | Yes |  |  |  |  |  |  |  |  | 1.14 | 1.02 | 1.166 | - |  |  |
|  | Social/leisure activity | No |  |  |  |  |  |  |  |  | 1.143 | 0.975 | 1.341 | - | 0.65 | NS |
|  |  | Yes |  |  |  |  |  |  |  |  | 1.1 | 1.034 | 1.17 |  |  |  |
| Lee et al., 2022 | Physical activity | None | Cognitive assessment test | Korean MMSE* | Korean MMSE* score | PM2.5 (1-year averaging) | Per IQR | Coefficient | - | - | -0.13 | -0.19 | -0.07 | Negative | <.0001 | S |
|  |  | ≥1/a week |  |  |  |  |  |  |  |  | -0.07 | -0.12 | -0.02 |  |  |  |
|  |  | None |  |  |  |  |  |  |  |  | -0.1 | -0.17 | -0.03 | Negative | <.0001 | S |
|  |  | ≥1/a week |  |  |  | PM10 (1-year averaging) |  |  |  |  | -0.08 | -0.15 | -0.01 |  |  |  |

*MMSE: MMSE: Mini-Mental State Examination RD: Residential distance; CCFM: Chicago Cognitive Function Measure.

** S/NS: Significance/Non-Significance; NP: Not provided

※ Two studies reported test results of effect modification by physical activity in text. One study (Shin et al., 2019) that provided graphics using cognitive scales with its 95% CIs suggested that physically inactive groups had significantly higher risk of Word list recognition function than those who are physically active. Another study (Cullen et al., 2018) stated that there was no evidence of significant interaction between time spent outdoor and types of air pollution tested in this study.

Significance: Shin et al., 2019

- Table E14: Effect size by potential effect modifier – Smoking Status (6/15)

| **Study** | | **Effect Modifier** | | **Outcome** | | | **Air**  **Pollutant** | **Exposure**  **Level** | **Types of Effect** | **Effect**  **Size**  **(A: Main Effect by exposure)** | **Effect Size**  **(C: Effect by interaction term)** | **Effect Size  Estimate**  **(A+C)** | **95% CI*s** | | **Direction of Modifier Measure** | **p-value  for  interaction terms** | **Statistical Significance (S/NS**)** |
| --- | --- | --- | --- | --- | --- | --- | --- | --- | --- | --- | --- | --- | --- | --- | --- | --- | --- |
|  |  | **Smoking Status** | | **Outcome Measure Method** | | **Outcome Variable** |  |  |  |  |  |  | **lower** | **upper** |  |  |  |
| Power et al., 2011 | | Smoking | Never/former/current | Cognitive assessment test | 7 tests (MMSE,  the digit span backward test, a verbal fluency  task, constructional praxis, immediate recall  of a 10-word list, delayed recall of a 10-word  list, and a pattern comparison task) | Cognitive score | Black carbon | - | - | - | - | NP** | NP | NP | - | 0.07 | NS |
| Ailshire and Crimmins, 2014 | | Smoking | Never | Cognitive assessment test | Global Cognitive Score | Continuous score | PM2.5 | Per quartiles | Coefficient | - | - | NP | NP | NP | Positive | 0.04 | S |
|  |  |  | Former |  |  |  |  |  |  |  |  | NP |  |  |  |  |  |
|  |  |  | Current |  |  |  |  |  |  |  |  | NP |  |  |  |  |  |
| Tzivian, Dlugaj, Winkler, Weinmayr, et al., 2016 | | Smoking | No | Cognitive assessment test | MCI Diagnosis | Prevalence of MCI | PM2.5 | per IQR | Odds Ratio | - | - | 1.01 | 0.85 | 1.21 | Positive | 0.02 | S |
|  |  |  | Former/Current |  |  |  |  |  |  |  |  | 1.39 | 1.12 | 1.71 |  |  |  |
| Tzivian, Dlugaj, Winkler, Hennig, et al., 2016 | | Smoking | No | Cognitive assessment test | | Global cognitive score | PM2.5 | per IQR | Coefficient | - | - | -0.41 | -0.31 | -0.45 |  | NP | NS |
|  |  |  | Former/Current |  |  |  |  |  |  |  |  | -0.23 | -0.15 | -0.32 | - |  |  |
| Tallon et al., 2017 | | Smoking | No | Cognitive assessment test | CCFM* | CCFM Score | PM2.5 | per IQR | Coefficient | - | - | -0.04 | -0.34 | 0.26 |  | 0.1 | NS |
|  |  |  | Yes |  |  |  |  |  |  |  |  | -0.86 | -1.55 | -0.17 | - |  |  |
|  |  |  | No |  |  |  | NO2 |  |  |  |  | 0.08 | -0.22 | 0.38 |  | 0.08 | NS |
|  |  |  | Yes |  |  |  |  |  |  |  |  | -0.3 | -0.74 | 0.15 |  |  |  |
| Carey et al., 2018 | | Smoking | Never/Ex/Current | Health administrative data  (coded with ICD-10) | | Diagnosis of incident dementia | NO_2_ | Per IQR (7.5 μg/m^3^)  increase | Hazard Ratio | - | - | NP | NP | NP | - | NP | NS |
| Salinas-Rodriguez et al., 2018 | | Smoking  (100 cigarettes smoking and/or current smoker) | No | Cognitive assessment test | 3WMT | Cognitive impairment | PM2.5 | per 10 ug/m3 increase | Odds Ratio | - | - | 1.37 | 1.06 | 1.76 | - | 0.76 | NS |
|  |  |  | Yes |  |  |  |  |  |  |  |  | 1.36 | 1.07 | 1.73 |  |  |  |
|  |  |  | No |  | SVF Test | SVF score |  |  | Coefficient |  |  | -0.72 | -1.06 | -0.39 | - | 0.99 | NS |
|  |  |  | Yes |  |  |  |  |  |  |  |  | -0.72 | -1.05 | -0.39 |  |  |  |
| Shin et al., 2019 | | Smoking | Current | Cognitive assessment test | Korean MMSE | Cognitive score | PM2.5 | Per IQR increase | Coefficients | - | - | NP | NP | NP | Positive (stronger in current smoking group) | NP | S |
|  |  |  | Former or nonsmoker |  |  |  |  |  |  |  |  |  |  |  |  |  |  |
| Kulick et al., 2020 | | Smoking | Never | Cognitive assessment test | Test batteries from WHICAP | Change in the test score | NO2 | Per IQR | Coefficient | - | - | -0.08 | -0.1 | -0.05 | - | 0.32 | NS |
|  |  |  | Former/Current |  |  |  |  |  |  |  |  | -0.07 | -0.1 | -0.05 |  |  |  |
|  |  |  | Never |  |  |  | PM2.5 |  |  |  |  | -0.09 | -0.12 | -0.07 | - | 0.61 |  |
|  |  |  | Former/Current |  |  |  |  |  |  |  |  | -0.09 | -0.12 | -0.06 |  |  |  |
|  |  |  | Never |  |  |  | PM10 |  |  |  |  | -0.05 | -0.07 | -0.03 | - | 0.13 |  |
|  |  |  | Former/Current |  |  |  |  |  |  |  |  | -0.04 | -0.06 | -0.030. |  |  |  |
| Wang et al., 2020 | | Smoking | Former/Never | Cognitive assessment test | Chinese MMSE | Poor cognitive function | PM2.5 | per 10 μg/m3 | Hazard Ratio | - | - | 1.04 | 1.01 | 1.08 | - | 0.24 | NS |
|  |  |  | Current |  |  |  |  |  |  |  |  | 1.08 | 1.02 | 1.13 |  |  |  |
| Yao et al., 2021 | | Tobacco smoking | Not-current | Cognitive assessment test | Chinese MMSE | Cognitive impairment | RD** to main roadway  ( ≤100m vs. >100m) | NP | Odds Ratio | - | - | 1.22 | 1.08 | 1.38 |  | 0.81 | NS |
|  |  |  | Current |  |  |  |  |  |  |  |  | 1.19 | 0.98 | 1.45 | - |  |  |
| G.-C. Chen et al., 2022 | | Smoking | Never | Hospitalization records  (coded with ICD-9 and ICD-10) | Dementia | Incident all-cause dementia | Air pollution score (PM2.5, PM2.5-10, PM10, NO2, NOx) | Per IQR | Hazard Ratio | - | - | 1.12 | 1.07 | 1.17 | Negative | 0.021 | S |
|  |  |  | Former |  |  |  |  |  |  |  |  | 1.12 | 1.06 | 1.17 |  |  |  |
|  |  |  | Current |  |  |  |  |  |  |  |  | 1.03 | 0.95 | 1.15 |  |  |  |
|  |  |  | Never |  |  | Incident vascular dementia |  |  |  |  |  | 1.16 | 1.08 | 1.25 | Negative | 0.005 | S |
|  |  |  | Former |  |  |  |  |  |  |  |  | 1.11 | 1.04 | 1.2 |  |  |  |
|  |  |  | Current |  |  |  |  |  |  |  |  | 1.03 | 0.89 | 1.2 |  |  |  |
|  |  |  | Never |  | AD | Incident AD |  |  |  |  |  | 1.14 | 1.03 | 1.26 | Negative | 0.019 | S |
|  |  |  | Former |  |  |  |  |  |  |  |  | 1.11 | 1.01 | 1.21 |  |  |  |
|  |  |  | Current |  |  |  |  |  |  |  |  | 1.00 | 0.84 | 1.19 |  |  |  |
| Gao et al., 2022 | | Smoking | Former/Never | Cognitive assessment test | Chinese MMSE | Cognitive impairment | Ozone | per 10 μg/m3 | Hazard Ratio | - | - | 1.106 | 1.038 | 1.177 | - | 0.894 | NS |
|  |  |  | Current |  |  |  |  |  |  |  |  | 1.096 | 0.958 | 1.252 |  |  |  |
| Lee et al., 2022 | | Smoking | Never | Cognitive assessment test | Korean MMSE* | Korean MMSE* score | PM2.5 (1-year averaging) | Per IQR | Coefficient | - | - | -0.15 | -0.2 | -0.09 | Negative | <0.0001 | S |
|  |  |  | Former |  |  |  |  |  |  |  |  | -0.04 | -0.11 | 0.03 |  |  |  |
|  |  |  | Current |  |  |  |  |  |  |  |  | -0.04 | -0.17 | 0.09 |  |  |  |
|  |  |  | Never |  |  |  | PM10 (1-year averaging) |  |  |  |  | -0.13 | -0.2 | -0.06 | Negative | 0.0004 | S |
|  |  |  | Former |  |  |  |  |  |  |  |  | -0.02 | -0.11 | 0.06 |  |  |  |
|  |  |  | Current |  |  |  |  |  |  |  |  | -0.04 | -0.18 | 0.11 |  |  |  |
| M. Li et al., 2022 | | Smoking | Non-smokers | CSF sTREM2 score | Neuroinflammatory (a biomarker of AD, represented by CSF sTREM2 score) | Continuous score | PM2.5 | NP | Z score | - | - | -0.087 | NP | NP | Positive | 0.0194 | S |
|  |  |  | Smokers |  |  |  |  |  |  |  |  | -0.179 |  |  |  | 0.0011 |  |
|  | *MMSE: MMSE: Mini-Mental State Examination RD: Residential distance; CCFM: Chicago Cognitive Function Measure;  ** S/NS: Significance/Non-Significance; NP: Not provided  ※ Five studies reported test results of effect modification by smoking status but didn’t provide effect size estimates but rather provided 1) graphics with overlapping 95% Cis (Shin et al., 2019; Carey et al., 2018) or 2) in-text (Ailshire and Crimmins 2014; Power et al., 2011).  Significant studies: Ailshire and Crimmins, 2014; Shin et al., 2019 reported in text at 0.05 level | | | | | | | | | | | | | | | | |

- Table E15: Effect size by potential effect modifier – Drinking Status (4/8)

| **Study** | | **Effect Modifier** | | **Outcome** | | | **Air**  **Pollutant** | **Exposure**  **Level** | **Types of Effect** | **Effect**  **Size**  **(A: Main Effect by exposure)** | **Effect Size**  **(C: Effect by interaction term)** | **Effect Size  Estimate**  **(A+C)** | **95% CI*s** | | **Direction of Modifier Measure** | **p-value  for  interaction terms** | **Statistical Significance (S/NS**)** |
| --- | --- | --- | --- | --- | --- | --- | --- | --- | --- | --- | --- | --- | --- | --- | --- | --- | --- |
|  |  | **Drinking Status** | | **Outcome Measure Method** | | **Outcome Variable** |  |  |  |  |  |  | **lower** | **upper** |  |  |  |
| Tzivian, Dlugaj, Winkler, Weinmayr, et al., 2016 | | Alcohol drinking | No/moderate | Cognitive assessment test | MCI Diagnosis | Prevalence of MCI | PM2.5 | per IQR | Odds Ratio | - | - | 1.27 | 1.07 | 1.5 | Negative | 0.05 | S |
|  |  |  | High consumption |  |  |  |  |  |  |  |  | 0.96 | 0.75 | 1.21 |  |  |  |
| Tzivian, Dlugaj, Winkler, Hennig, et al., 2016 | | Alcohol drinking | No/moderate | Cognitive assessment test | | Global cognitive score | PM2.5 | per IQR | Coefficient | - | - | -0.41 | -0.57 | -0.24 | Negative | 0.01 | S |
|  |  |  | High consumption |  |  |  |  |  |  |  |  | -0.23 | -0.37 | 0.1 |  |  |  |
| Salinas-Rodriguez et al., 2018 | | Alcohol drinking | No | Cognitive assessment test | 3WMT | Cognitive impairment | PM2.5 | per 10 ug/m3 increase | Odds Ratio | - | - | 1.36 | 1.06 | 1.74 | - | 0.84 | NS |
|  |  |  | Yes |  |  |  |  |  |  |  |  | 1.35 | 1.05 | 1.73 |  |  |  |
|  |  |  | No |  | SVF Test | SVF score |  |  | Coefficient |  |  | -0.74 | -1.07 | -0.42 | - | 0.11 |  |
|  |  |  | Yes |  |  |  |  |  |  |  |  | -0.61 | -0.96 | -0.26 |  |  |  |
| Shin et al., 2019 | | Alcohol drinking | <1 per week | Cognitive assessment test (recognition test) | Korean MMSE | Cognitive score | PM2.5 | Per IQR increase | Coefficients | - | - | NP** | NP | NP |  | NP | S |
|  |  |  | More than 1 per week |  |  |  |  |  |  |  |  |  |  |  | Positive |  |  |
| Wang et al., 2020 | | Alcohol drinking | Former/Never | Cognitive assessment test | Chinese MMSE | Poor cognitive function | PM2.5 | per 10 μg/m3 | Hazard Ratio | - | - | 1.06 | 1.03 | 1.09 | - | 0.75 | NS |
|  |  |  | Current |  |  |  |  |  |  |  |  | 1.03 | 0.98 | 1.09 |  |  |  |
| Yao et al., 2021 | | Alcohol drinking | Not-current | Cognitive assessment test | Chinese MMSE | Cognitive impairment | RD** to main roadway | ≤100m vs. >100m | Odds Ratio | - | - | 1.20 | 1.07 | 1.35 | - | 0.72 | NS |
|  |  |  | Current |  |  |  |  |  |  |  |  | 1.23 | 0.99 | 1.52 |  |  |  |
| Gao et al., 2022 | | Alcohol drinking | Former/Never | Cognitive assessment test | Chinese MMSE | Cognitive impairment | Ozone | per 10 μg/m3 | Hazard Ratio | - | - | 1.09 | 0.96 | 1.24 | - | 0.829 | NS |
|  |  |  | Current |  |  |  |  |  |  |  |  | 1.11 | 1.04 | 1.18 |  |  |  |
| Lee et al., 2022 | | Alcohol drinking | Never | Cognitive assessment test | Korean MMSE* | Korean MMSE* score | PM2.5 | 1-year averaging | Coefficient | - | - | -0.15 | -0.21 | -0.09 | Qualitative | 0.01 | S |
|  |  |  | Former |  |  |  |  |  |  |  |  | 0.06 | -0.09 | 0.20 |  |  |  |
|  |  |  | Current |  |  |  |  |  |  |  |  | -0.09 | -0.15 | -0.03 |  |  |  |
|  |  |  | Never |  |  |  |  | 2-year averaging |  |  |  | -0.19 | -0.25 | -0.12 | Qualitative | 0.002 | S |
|  |  |  | Former |  |  |  |  |  |  |  |  | 0.03 | -0.13 | 0.20 |  |  |  |
|  |  |  | Current |  |  |  |  |  |  |  |  | -0.11 | -0.18 | -0.03 |  |  |  |
|  |  |  | Never |  |  |  |  | 3-year averaging |  |  |  | -0.17 | -0.24 | -0.10 | Qualitative | 0.002 | S |
|  |  |  | Former |  |  |  |  |  |  |  |  | 0.07 | -0.10 | 0.23 |  |  |  |
|  |  |  | Current |  |  |  |  |  |  |  |  | -0.11 | -0.18 | -0.03 |  |  |  |
|  |  |  | Never |  |  |  | PM10 | 1-year averaging |  |  |  | -0.13 | -0.2 | -0.05 | Qualitative | 0.02 | S |
|  |  |  | Former |  |  |  |  |  |  |  |  | 0.03 | -0.16 | 0.22 |  |  |  |
|  |  |  | Current |  |  |  |  |  |  |  |  | -0.08 | -0.15 | -0.01 |  |  |  |
|  |  |  | Never |  |  |  |  | 2-year averaging |  |  |  | -0.18 | -0.26 | -0.1 | Qualitative | 0.002 | S |
|  |  |  | Former |  |  |  |  |  |  |  |  | 0.02 | -0.17 | 0.21 |  |  |  |
|  |  |  | Current |  |  |  |  |  |  |  |  | -0.08 | -0.16 | -0.01 |  |  |  |
|  |  |  | Never |  |  |  |  | 3-year averaging |  |  |  | -0.16 | -0.24 | -0.09 | Qualitative | 0.001 | S |
|  |  |  | Former |  |  |  |  |  |  |  |  | 0.04 | -0.14 | 0.21 |  |  |  |
|  |  |  | Current |  |  |  |  |  |  |  |  | -0.09 | -0.16 | -0.02 |  |  |  |
|  | *MMSE: MMSE: Mini-Mental State Examination RD: Residential distance  ** S/NS: Significance/Non-Significance; NP: Not provided;  ※ One study (Shin et al., 2019) provided test results of modification effect of drinking status in graphics and no effect size measures were provided. Based on in-text information two groups were statistically different (alcohol intake less or equal to 1 per week vs. 1 per week) and reported drinking group showed worsen cognitive function with PM2.5 exposure.  Significant study: Shin et al., 2019 | | | | | | | | | | | | | | | | |

- Table E16: Effect size by potential effect modifier – Neighborhood Stress (1/1)

| **Study** | | **Effect Modifier** | | **Outcome** | | | **Air**  **Pollutant** | **Exposure**  **Level** | **Types of Effect** | **Effect**  **Size**  **(A: Main Effect by exposure)** | **Effect Size**  **(C: Effect by interaction term)** | **Effect Size  Estimate*****  **(A+C)** | **95% CI*s** | | **Direction of Modifier Measure** | **p-value  for  interaction terms** | **Statistical Significance (S/NS**)** |
| --- | --- | --- | --- | --- | --- | --- | --- | --- | --- | --- | --- | --- | --- | --- | --- | --- | --- |
|  |  | **Neighborhood Stress** | | **Outcome Measure Method** | | **Outcome Variable** |  |  |  |  |  |  | **lower** | **upper** |  |  |  |
| Ailshire et al., 2017 | | Neighborhood Stressor | Low | Cognitive assessment test | SPMSQ* | Cognitive error score** | PM2.5 | Per 1 mg/m3 | Coefficients | 1.03 | 0 | 1.03 | NP** | |  | <0.05 | S*** |
|  |  |  | High |  |  |  |  |  |  | 1.03 | 1.09 | 2.12 |  |  | Not applicable  (same effect sizes) |  |  |
|  | *SPMSQ: Short Portable Mental Status Questionnaire  ** Ranging from 0-5, higher scores indicate poorer cognitive function.; S/NS: Significance/Non-Significance; NP: Not provided  *** The total effect size (A+C) was manually calculated unless the effect of air pollution exposure on each category of the effect modifier was provided in the studies  **** Cognitive function is significantly higher in high-stress neighborhood group. | | | | | | | | | | | | | | | | |

- Table E17: Effect size by potential effect modifier – Fuel types for cooking (1/2)

| **Study** | **Effect Modifier** | | **Outcome** | | | **Air**  **Pollutant** | **Exposure**  **Level** | **Types of Effect** | **Effect**  **Size**  **(A: Main Effect by exposure)** | **Effect Size**  **(C: Effect by interaction term)** | **Effect Size  Estimate**  **(A+C)** | **95% CI*s** | | **Direction of Modifier Measure** | **p-value  for  interaction terms** | **Statistical Significance (S/NS**)** |
| --- | --- | --- | --- | --- | --- | --- | --- | --- | --- | --- | --- | --- | --- | --- | --- | --- |
|  | **Fuel types for cooking** | | **Outcome Measure Method** | | **Outcome Variable** |  |  |  |  |  |  | **lower** | **upper** |  |  |  |
| Ssalinas- Rodriguez et al., 2018 | Fuel types | Unclean | Cognitive assessment test | 3WMT | Cognitive impairment | PM2.5 | per 10 μg/m3 | Odds Ratio | - | - | 1.33 | 1.04 | 1.71 | - | 0.51 | NS |
|  |  | Clean |  |  |  |  |  |  |  |  | 1.31 | 1.01 | 1.71 |  |  |  |
|  | Fuel types | Unclean |  | SVF Test | SVF score |  |  | Coefficient |  |  | -0.42 | -1.16 | 0.32 | - | 0.61 | NS |
|  |  | Clean |  |  |  |  |  |  |  |  | -0.46 | -1.27 | 0.35 |  |  |  |
| Yao et al., 2021 | Fuel types | Solid fuel | Cognitive assessment test | Chinese MMSE | Cognitive impairment | RD** to main roadway  ( ≤100m vs. >100m) |  | Odds Ratio | - | - | 1.37 | 1.13 | 1.67 | positive | 0.028 | S |
|  |  | Clean |  |  |  |  | NP** |  |  |  | 1.13 | 1.04 | 1.21 |  |  |  |

** S/NS: Significance/Non-Significance; NP: Not provided

- Table E18: Effect size by potential effect modifier – Ventilation in indoor cooking (1/2)

| **Study** | **Effect Modifier** | | **Outcome** | | | **Air**  **Pollutant** | **Exposure**  **Level** | **Types of Effect** | **Effect**  **Size**  **(A: Main Effect by exposure)** | **Effect Size**  **(C: Effect by interaction term)** | **Effect Size  Estimate**  **(A+C)** | **95% CI*s** | | **Direction of Modifier Measure** | **p-value  for  interaction terms** | **Statistical Significance (S/NS**)** |
| --- | --- | --- | --- | --- | --- | --- | --- | --- | --- | --- | --- | --- | --- | --- | --- | --- |
|  | **Ventilation in indoor cooking** | | **Outcome Measure Method** | | **Outcome Variable** |  |  |  |  |  |  | **lower** | **upper** |  |  |  |
| Ssalinas-Rodriguez et al., 2018 | Ventilation  Ventilation | Without ventilation | Cognitive assessment test | 3WMT | Cognitive impairment | PM2.5 | per 10 μg/m3 | Odds Ratio | - | - | 1.35 | 1.06 | 1.72 |  | 0.21 | NS |
|  |  | With ventilation |  |  |  |  |  |  |  |  | 1.30 | 1.01 | 1.67 | - |  |  |
|  | Ventilation  Ventilation | Without ventilation |  | SVF Test | SVF score |  |  | Coefficient |  |  | -0.35 | -1.06 | 0.35 |  | 0.93 | NS |
|  |  | With ventilation |  |  |  |  |  |  |  |  | -0.37 | -1.27 | 0.35 | - |  |  |
| Yao et al., 2021 | Ventilation  Ventilation | None | Cognitive assessment test | Chinese MMSE | Cognitive impairment | RD to main roadway  ( ≤100m vs. >100m) | NP** | Odds Ratio | - | - | 1.86 | 1.31 | 2.64 |  | 0.001 | S |
|  |  | Mechanical or natural |  |  |  |  |  |  |  |  | 1.16 | 1.03 | 1.26 | Negative |  |  |

** S/NS: Significance/Non-Significance; NP: Not provided

- Table E19: Effect size by potential effect modifier – Noise (1/2)

| **Study** | | **Effect Modifier** | | **Outcome** | | | **Air**  **Pollutant** | **Exposure**  **Level** | **Types of Effect** | **Effect**  **Size**  **(A: Main Effect by exposure)** | **Effect Size**  **(C: Effect by interaction term)** | **Effect Size  Estimate**  **(A+C)** | **95% CI*s** | | **Direction of Modifier Measure** | **p-value  for  interaction terms** | **Statistical Significance (S/NS**)** |
| --- | --- | --- | --- | --- | --- | --- | --- | --- | --- | --- | --- | --- | --- | --- | --- | --- | --- |
|  |  | **Noise** | | **Outcome Measure Method** | | **Outcome Variable** |  |  |  |  |  |  | **lower** | **upper** |  |  |  |
| Tzivian, Dlugaj, Winkler, Weinmayr, et al., 2016 | | Noise | Low (<60 dB) | Cognitive assessment test | MCI Diagnosis | Prevalence of MCI | PM2.5 | per IQR | Odds Ratio | - | - | 1.1 | 0.93 | 1.29 |  | 0.43 | NS |
|  |  |  | High (≥60 dB) |  |  |  |  |  |  |  |  | 1.3 | 1.01 | 1.67 |  |  |  |
| Tzivian et al., 2017† | | Road traffic noise | Low (<60 dB) | Cognitive assessment test | | global cognitive score (GCS) | PM2.5 | per IQR | z-score | - | - | −0.16 | −0.33 | 0.01 | positive | 0.04 | NS |
|  |  |  | High (≥60 dB) |  |  |  |  |  |  |  |  | −0.48 | −0.72 | −0.23 |  |  |  |
|  | †p-value of interaction term was provided in text selectively for certain modifiers  ** S/NS: Significance/Non-Significance | | | | | | | | | | | | | | | | |

- Table E20: Effect size by potential effect modifier – Others (Individuals; 2/4)

| **Study** | | **Effect Modifier** | | **Outcome** | | | **Air**  **Pollutant** | **Exposure**  **Level** | **Types of Effect** | **Effect**  **Size**  **(A: Main Effect by exposure)** | **Effect Size**  **(C: Effect by interaction term)** | **Effect Size  Estimate**  **(A+C)** | **95% CI*s** | | **Direction of Modifier Measure** | **p-value  for  interaction terms** | **Statistical Significance (S/NS**)** |
| --- | --- | --- | --- | --- | --- | --- | --- | --- | --- | --- | --- | --- | --- | --- | --- | --- | --- |
|  |  |  |  | **Outcome Measure Method** | | **Outcome Variable** |  |  |  |  |  |  | **lower** | **upper** |  |  |  |
| Ranft et al., 2009 | | Exposure to traffic | - | Cognitive assessment test | CERAD plus*/ Stroop/Sniffing | Cognitive impairment | Distance from traffic | NP | Coefficients | - | - | NP** | NP | NP | - | NP | NS |
| Gao et al., 2022 | | Exposure to PM2.5 | Lower tertile | Cognitive assessment test | Chinese MMSE | Cognitive impairment | Ozone | per 10 μg/m3 | Hazard Ratio | - | - | 1.142 | 1.142 | 1.039 | - | 0.142 | NS |
|  |  |  | Middle tertile |  |  |  |  |  |  |  |  | 1.159 | 1.159 | 1.052 |  |  |  |
|  |  |  | Upper tertile |  |  |  |  |  |  |  |  | 1.025 | 1.025 | 0.93 |  |  |  |
| Wyatt et al., 2023 | | Game Stage | Stage 1 | Cognitive assessment game | *Lost in Migration* game | Score representing cognitive performance index | PM2.5 | per 1 μg/m3 | Change in score percentile | - | - | -0.14 | -0.25 | -0.03 | - | NP | NS |
|  |  |  | Stage 4 |  |  |  |  |  |  |  |  | -0.14 | -0.21 | -0.07 |  |  |  |
|  |  | User behavior | Habitual users |  |  |  | PM2.5 |  |  |  |  | -0.45 | -0.65 | -0.23 | - | NP | NP |
|  |  |  | Less habitual |  |  |  |  |  |  |  |  | NP | | |  |  |  |
|  |  | Speed to complete 20 games | Fast completion times |  |  |  | O3 |  |  |  |  | -0.69 | -1.41 | 0.04 | Qualitative | NP | S |
|  |  |  | Slower completion times |  |  |  |  |  |  |  |  | 1.32 | 0.60 | 1.99 |  |  |  |
|  | ※ One study (Ranft et al., 2009) tested traffic exposure as an indicator of air pollution exposure as an effect modifier but reported it as non-significant (P-value<0.1) in text.  ** S/NS: Significance/Non-Significance; NP: Not provided | | | | | | | | | | | | | | | | |

- Table E20: Effect size by potential effect modifier – Others (Individuals; 2/4) - Continued

| **Study** | **Effect Modifier** | | | **Outcome** | | | **Air**  **Pollutant** | **Exposure**  **Level** | **Types of Effect** | **Effect**  **Size**  **(A: Main Effect by exposure)** | **Effect Size**  **(C: Effect by interaction term)** | **Effect Size  Estimate**  **(A+C)** | **95% CI*s** | | **Direction of Modifier Measure** | **p-value  for  interaction terms** | **Statistical Significance (S/NS**)** |
| --- | --- | --- | --- | --- | --- | --- | --- | --- | --- | --- | --- | --- | --- | --- | --- | --- | --- |
|  | **Outcome Measure Method** | | **Outcome Measure Method** | | | **Outcome Variable** |  |  |  |  |  |  | **lower** | **upper** |  |  |  |
| Alemany et al., 2021 | Amyloid Status (neurodegenerative disease) | Positive | CSF Biomarker amount***** (Except CSF Aβ42/40, which was a ratio | | phosphorylated tau (p-tau) | continuous | NO2 | Per 1 standard deviation (10.9 ug/m^3^) increase | Coefficient | - | - | 0.29 | NP** | |  | 0.081 | NS |
|  |  | Negative |  |  |  |  |  |  |  |  |  | 0.01 |  |  |  |  |  |
|  |  | Positive |  |  |  |  | PM2.5 | Per 1 standard deviation (2.2 ug/m^3^) increase |  |  |  | 0.37 |  |  | Positive | 0.03 | S |
|  |  | Negative |  |  |  |  |  |  |  |  |  | 0.01 |  |  |  |  |  |
|  |  | Positive |  |  |  |  | PM10 | Per 1 standard deviation (4.1 ug/m^3^) increase |  |  |  | 0.30 |  |  | Qualitative | 0.043 | S |
|  |  | Negative |  |  |  |  |  |  |  |  |  | -0.04 |  |  |  |  |  |
|  |  | Positive |  |  |  |  | PM2.5 abs | Per 1 standard deviation (0.6 ug/m^3^) increase |  |  |  | 0.32 |  |  |  | 0.08 | NS |
|  |  | Negative |  |  |  |  |  |  |  |  |  | 0.02 |  |  |  |  |  |
|  |  | Positive |  |  | total tau (t-tau) |  | NO2 | Per 1 standard deviation (10.9 ug/m^3^) increase |  |  |  | 0.34 |  |  | Qualitative | 0.039 | S |
|  |  | Negative |  |  |  |  |  |  |  |  |  | -0.01 |  |  |  |  |  |
|  |  | Positive |  |  |  |  | PM2.5 | Per 1 standard deviation (2.2 ug/m^3^) increase |  |  |  | 0.38 |  |  |  | 0.017 | NS |
|  |  | Negative |  |  |  |  |  |  |  |  |  | 0.00 |  |  |  |  |  |
|  |  | Positive |  |  |  |  | PM10 | Per 1 standard deviation (4.1 ug/m^3^) increase |  |  |  | 0.33 |  |  | Qualitative | 0.022 | S |
|  |  | Negative |  |  |  |  |  |  |  |  |  | -0.05 |  |  |  |  |  |
|  |  | Positive |  |  |  |  | PM2.5 abs | Per 1 standard deviation (0.6 ug/m^3^) increase |  |  |  | 0.38 |  |  | Positive | 0.024 | S |
|  |  | Negative |  |  |  |  |  |  |  |  |  | 0.02 |  |  |  |  |  |
|  |  | Positive |  |  | neurofilament light (NfL) |  | NO2 | Per 1 standard deviation (10.9 ug/m^3^) increase |  |  |  | 0.36 |  |  | Qualitative | 0.012 | S |
|  |  | Negative |  |  |  |  |  |  |  |  |  | -0.02 |  |  |  |  |  |
|  |  | Positive |  |  |  |  | PM2.5 | Per 1 standard deviation (2.2 ug/m^3^) increase |  |  |  | 0.31 |  |  |  | 0.208 | NS |
|  |  | Negative |  |  |  |  |  |  |  |  |  | 0.12 |  |  |  |  |  |
|  |  | Positive |  |  |  |  | PM10 | Per 1 standard deviation (4.1 ug/m^3^) increase |  |  |  | 0.34 |  |  | Positive | 0.046 | S |
|  |  | Negative |  |  |  |  |  |  |  |  |  | 0.04 |  |  |  |  |  |
|  |  | Positive |  |  |  |  | PM2.5 abs | Per 1 standard deviation (0.6 ug/m^3^) increase |  |  |  | 0.41 |  |  | Positive | 0.015 | S |
|  |  | Negative |  |  |  |  |  |  |  |  |  | 0.03 |  |  |  |  |  |
|  |  | Positive |  |  | Centiloid values (CL) |  | NO2 | Per 1 standard deviation (10.9 ug/m^3^) increase |  |  |  | 0.36 |  |  | Positive | 0.001 | S |
|  |  | Negative |  |  |  |  |  |  |  |  |  | 0.08 |  |  |  |  |  |
|  |  | Positive |  |  |  |  | PM2.5 | Per 1 standard deviation (2.2 ug/m^3^) increase |  |  |  | 0.23 |  |  | Positive | 0.048 | S |
|  |  | Negative |  |  |  |  |  |  |  |  |  | 0.04 |  |  |  |  |  |
|  |  | Positive |  |  |  |  | PM10 | Per 1 standard deviation (4.1 ug/m^3^) increase |  |  |  | 0.22 |  |  | Positive | 0.023 | S |
|  |  | Negative |  |  |  |  |  |  |  |  |  | 0.06 |  |  |  |  |  |
|  |  | Positive |  |  |  |  | PM2.5 abs | Per 1 standard deviation (0.6 ug/m^3^) increase |  |  |  | 0.26 |  |  | Positive | 0.026 | S |
|  |  | Negative |  |  |  |  |  |  |  |  |  | 0.11 |  |  |  |  |  |
| ** S/NS: Significance/Non-Significance; NP: Not provided  ***** Higher amount of CSF biomarker indicated more pathological level (Alemany et al., 2021) | | | | | | | | | | | | | | | | | |
