# Supplementary material for: A critical review of potential modifiers of air pollutant associations with dementia and related outcomes
Source: Neurotoxicology. Author manuscript; Available in PMC 2026 Jul 7. (PMC13339730; doi:10.1016/j.neuro.2026.103470)
Supplement: Supplementary Materials 02 [file NIHMS2188061-supplement-Supplementary_Materials_02.docx]

**Table S1. Bias Assessment of the Cohort Studies**

| Articles | Were the two groups similar and recruited from the same population? | Were the exposures measured similarly to assign people to both exposed and unexposed groups? | Was the exposure measured in a valid and reliable way? | Were confounding factors identified? | Were strategies to deal with confounding factors stated? | Were the groups/participants free of the outcome at the start of the study (or at the moment of exposure)? | Were the outcomes measured in a valid and reliable way? | Was the follow up time reported and sufficient to be long enough for outcomes to occur? | Was follow up complete, and if not, were the reasons to loss to follow up described and explored? | Were strategies to address incomplete follow up utilized? | Was appropriate statistical analysis used? | Bias assessment score |
| --- | --- | --- | --- | --- | --- | --- | --- | --- | --- | --- | --- | --- |
| Carey et al., 2018 | Y / Y | Y/Y | Y / Y | Y / Y | Y / Y | Y / Y | Y / Y | Y / Y | N / N | N / N | Y / Y | Low |
| C. Chen et al., 2022 | Y / Y | Y / Y | Y / Y | Y / Y | Y / Y | Y / Y | Y / Y | Y / Y | Unclear / Unclear | N / Y | Y / Y | Low |
| Colicino et al., 2016 | Y / Y | Y / Y | Y / Y | Y / Y | Y / Y | Y / Y | Y / Y | Y / Y | Y / Y | Y / Y | Y / Y | Low |
| Colicino et al., 2014 | Y / Y | Y / Y | Y / Y | Y / Y | Y / Y | Y / Y | Y / Y | Y / Y | N / N | N / N | Y / Y | Low |
| Colicino et al., 2017 | Y / Y | Y / Y | Y / Y | Y / Y | Y / Y | Unclear / Unclear | Y / Y | Y / Y | Y / Y | N / N | Y / Y | Low |
| Fehsel et al., 2016 | Y / Y | Y / Y | Y / Y | Y / Y | Y / Y | Unclear / Unclear | Y / Y | Y / Y | Unclear / Unclear | Unclear / Unclear | Y / Y | Low |
| He et al., 2022 | Y / Y | Y / Y | Y / Y | Y / Y | Y / Y | Unclear / Unclear | Y / Y | Y / Y | Y / Y | N / N | Y / Y | Low |
| Loop et al., 2013 | Y / Y | Y / Y | Y / Y | Y / Y | Y / Y | Y / Y | Y / Y | Y / Y | Y / Y | N / N | Y / Y | Low |
| Schikowski et al., 2015 | Y / Y | Y / Y | Y / Y | Y / Y | Y / Y | Unclear / Unclear | Y / Y | Y / Y | Y / Y | N / N | Y / Y | Low |
| Shin et al., 2019 | Y / Y | Y / Y | Y / Y | Y / Y | Y / Y | Y / Y | Y / Y | N / N | Unclear / Unclear | N / N | Y / Y | Low |
| Oudin et al., 2019 | Y / Y | Y / Y | Y / Y | Y / Y | Y / Y | Y / Y | Y / Y | Y / Y | Y / Y | N / N | Y / Y | Low |
| Cleary et al., 2018 | Y / Y | Y / Y | Y/Y | Y / Y | Y / Y | Unclear / Y | Y / Y | Y/Y | Y/Y | Y/Y | Y/Y | Low |
| Chen et al., 2021 | Y / Y | Y / Y | Y / Y | Y / Y | Y / Y | Y / Y | Y / Y | Y / Y | Y / Y | N / N | Y / Y | Low |
| Tallon et al., 2017 | Y / Y | Y / Y | Unclear / Unclear | Y / Y | Y / Y | N / Unclear | N / Unclear | Y / Y | Y / Y | N / N | Y / Y | Low |
| Parra et al., 2022 | Y / Y | Y / Y | Y / Y | Y / Y | Y / Y | Y / Y | N / N | Y / Y | Y / Y | Y / Y | Y / Y | Low |
| Alemany et al., 2021 | Y / Y | Y / Y | N / N | Y / Y | Y / Y | Y / Y | Y / Y | N /N | N /N | N /N | Y / Y | Low |
| Yu et al., 2020 | Y / Y | Y / Y | Y /Y | Y /Y | Y /Y | Y /Y | Y /Y | Y /Y | Y /Y | N /N | Y /Y | Low |
| Ran et al., 2021 | Y / Y | Y / Y | Y /Y | Y /Y | Y /Y | Y /Y | Y /Y | Y /Y | Y /Y | Y /Y | Y /Y | Low |
| Grande et al., 2021 | Y / Y | Y / Y | Y /Y | Y /Y | Y /Y | Y /Y | Y /Y | Y /Y | N /N | N/N | Y /Y | Low |
| Power et al., 2013 | Y / Y | Y / Y | Unclear / Unclear | Y /Y | Y /Y | Unclear/N | Y /Y | Y /Y | Unclear /N | Unclear /N | Y /Y | Low |
| Zhu et al., 2022 | Y / Y | Y / Y | Y /Y | Y /Y | Y /Y | Y /Y | Y /Y | Y /Y | Y /Y | N /N | Y /Y | Low |
| Ma et al., 2022 | Y / Y | Y / Y | Y / Y | Y / Y | Y / Y | Y / Y | Y / Y | Y / Y | Y / Y | N / N | Y / Y | Low |
| Hedges et al., 2020 | Y / Y | Y / Y | Y / Y | Y / Y | Y / Y | Unclear | Y / Y | Y / Y | Unclear/Unclear | N / N | Y / Y | Low |
| Chen et al., 2017 | Y / Y | Y / Y | Y / Y | Y / Y | Y / Y | Y / Y | Y / Y | Y / Y | Y /Unclear | N /N | Y / Y | Low |
| Wang et al., 2020 | Y / Y | Y / Y | Y / Y | Y / Y | Y / Y | Y / Y | Y / Y | Y / Y | Y / Y | N /N | Y / Y | Low |
| Ranft et al., 2009 | Y / Y | Y / Y | Y / Y | Y / Y | Y / Y | Unclear /Unclear | Y / Y | Y / Y | Y / Y | N /N | Y / Y | Low |
| Wellenius et al., 2012 | Y / Y | Y / Y | Y / Y | Y / Y | Y / Y | Unclear /Y | Y / Y | N /Y | N /N | N /N | Y / Y | Low |
| Shi et al., 2020 | Y / Y | Y / Y | Y / Y | Y / Y | Y / Y | Unclear /Y | Y / Y | Y / Y | N /Unclear | N /N | Y / Y | Low |
| Kulick et al., 2020 | Y / Y | Y / Y | Y / Y | Y / Y | Y / Y | Y / Y | Y / Y | Y / Y | Y / Y | Y / Y | Y / Y | Low |
| Yang et al., 2022 | Y / Y | Y / Y | Y / Y | Y / Y | Y / Y | Y / Y | Y / Y | Y / Y | Y / Y | N / N | Y / Y | Low |
| Younan et al., 2021 | Y / Y | Y / Y | Y / Y | Y / Y | Y / Y | Unclear / Y | Y / Y | N / Y | Y / Y | N / N | Y / Y | Low |
| Lucht et al., 2022 | Y / Y | Y / Y | Y / Y | Y / Y | Y / Y | N / N | Y / Y | Y / Y | Y / Y | N / N | Y / Y | Low |
| Shaffer et al., 2021 | Y / Y | Y / Y | Y / Y | Y / Y | Y / Y | N / Y | Y / Y | Y / Y | Y / Y | N / Y | Y / Y | Low |
| Cerza et al., 2019 | Y / Y | Y / Y | N / N | Y / Y | Y / Y | N / Y | Y / Y | Y / Y | Y / Y | N / N | Y / Y | Low |
| Wang et al., 2022 | Y / Y | Y / Y | Y / Y | Y / Y | Y / Y | Y / Y | Y / Y | N / Y | Y / Y | N / N | Y / Y | Low |
| Gao et al., 2022 | Y / Y | Y / Y | Y / Y | Y / Y | Y / Y | Y / Y | Y / Y | Y / Y | Y / Y | N / N | Y / Y | Low |
| G.-C. Chen et al., 2022 | Y / Y | Y / Y | Y / Y | Y / Y | Y / Y | Y / Y | Y / Y | N / Y | N / N | N / N | Y / Y | Low |
| Wyatt et al., 2023 | Y / Y | Y / Y | Y / Y | Y / Y | Y / Y | Y / y | Y / Y | N / N | N / N | N / N | Y / Y | Low |
| Decrom et al., 2022 | Y / Y | Y / Y | Y / Y | Y / Y | Y / Y | Y / Y | Y / Y | N / Y | N / N | N / N | Y / Y | Low |
| Lee et al., 2022 | Y / Y | Y / Y | Y / Y | Y / Y | Y / Y | N / N | Y / Y | Y / Y | Y / Y | N / N | Y / Y | Low |
| Crous-Bou et al., 2020 | Y / Y | Y / Y | N / Y | Y / Y | Y / Y | Y / Y | Y / Y | Unclear / Y | N / N | N / N | Y / Y | Low |
| Chen et al., 2020 | Y / Y | Y / Y | Y / Y | Y / Y | Y / Y | Y / Y | Y / Y | N / Unclear | Y / Y | N / N | Y / Y | Low |
| Shi et al., 2023 | Y / Y | Y / Y | Y / Y | Y / Y | Y / Y | Y / Y | Y / Y | Y / Y | N / N | N / N | Y / Y | Low |
| Hu et al., 2022 | Y / Y | Y / Y | Unclear / Y | Y / Y | Y / Y | N / N | Y / Y | Y / Y | Y / Y | N / N | Y / Y | Low |
| Mortamais et al., 2021 | Y / Y | Y / Y | Y / Y | Y / Y | Y / Y | Y / Y | Y / Y | Y / Y | Y / Y | N / N | Y / Y | Low |
| Cullen et al., 2018 | Y / Y | Y / Y | Y / Y | Y / Y | Y / Y | Y / Y | Y / Y | N / Y? | N / N | N / N | Y / Y | Low |
| Shi et al., 2021 | Y / Y | Y / Y | Y / Y | Y / Y | Y / Y | Y / Y | Y / Y | Y / Y | N / N | N / N | Y / Y | Low |
| Chen et al., 2015 | Y / Y | Y / Y | Y / Y | Y / Y | Y / Y | Y / Y | Y / Y | Y / Y | N | N /N | Y / Y | Low |
| Hu et al., 2023 | Y / Y | Y / Y | Y / Y | Y / Y | Y / Y | Unclear / Unclear | Y / Y | Y / Y | Y / Y | N / N | Y / Y | Low |
| Lee et al., 2019 | Y / Y | Y / Y | Y / Y | Y / Y | Y / Y | Y / Y | Y / Y | Y / Y | N / N | N / N | Y / Y | Low |
| M. Li et al., 2022 | Y / Y | Y / Y | Y / Y | Y / Y | Y / Y | Y / Y | Y / Y | Unclear / Unclear | N / N | N / N | Y / Y | Low |
| Power et al., 2011 | Y / Y | Y / Y | Unclear / Y | Y / Y | Y / Y | Y / Y | Y / Y | Y / Y | Y / Y | N / N | Y / Y | Low |
| Semmens et al., 2022 | Y / Y | Y / Y | Y / Y | Y / Y | Y / Y | Y / Y | Y / Y | Y / Y | N / N | N / N | Y / Y | Low |
| Yuchi et al., 2020 | Y / Y | Y / Y | Y / Y | Y / Y | Y / Y | Y / Y | Y / Y | Y / Y | N / N | N / N | Y / Y | Low |

*Results are listed based on an assessment by YL/JH up until Grande et al., 2021; and by YL/DL for Power et al., 2013 to Semmens et al., 2022.

**Table S2. Bias Assessment for the Cross-Sectional Studies**

| Articles | Inclusion criteria clearly defined? | Study subjects and setting described in detail? | Were exposure measurements valid and reliable? | Objective, standard criteria used for measurement of the condition? | Confounding factors identified? | Were strategies to deal with confounding factors stated? | Were outcome measurements valid and reliable? | Was statistical analysis appropriate? | Bias score |
| --- | --- | --- | --- | --- | --- | --- | --- | --- | --- |
| Ailshire et al., 2014 | N / N | Y / Y | Y / Y | Not applicable / Not applicable | Y / Y | Y / Y | Y/Y | Y / Y | Low |
| Ailshire et al., 2017 | N / N | Y / Y | Y / Y | Not applicable / Not applicable | Y / Y | Y / Y | Y / Y | Y / Y | Low |
| Ailshire et al., 2021 | Y / Y | Y / Y | Y / Y | Not applicable / Not applicable | Y / Y | Y / Y | Y/Y | Y / Y | Low |
| Cacciottolo et al., 2017 | Y / Y | Y / Y | Y / Y | Not applicable / Not applicable | Y / Y | Y / Y | Y / Y | Y / Y | Low |
| Hedges et al., 2019 | N / N | Y / Y | Y / Y | Not applicable / Not applicable | Y / Y | Y / Y | Y / Y | Y / Y | Low |
| Kim et al., 2019 | Y / Y | Y / Y | Y / Y | Not applicable / Not applicable | Y / Y | Y / Y | Y / Y | Y / Y | Low |
| Nunez et al., 2021 | Y / Y | Y / Y | Y / Y | Not applicable / Not applicable | Y / Y | Y / Y | Y / Y | Y / Y | Low |
| Tzivian, Dlugaj, Winkler, Weinmayr, et al., 2016 | Y / Y | Y / Y | Y / Y | Not applicable / Not applicable | Y / Y | Y / Y | Y / Y | Y / Y | Low |
| Tzivian, Dlugaj, Winkler, Hennig, et al., 2016 | Y / Y | Y / Y | N / N | Not applicable / Not applicable | Y / Y | Y / Y | Y / Y | Y / Y | Low |
| Yao et al., 2021 | Y / Y | Y / Y | N / N | Not applicable / Not applicable | Y / Y | Y / Y | Y / Y | Y / Y | Low |
| Tzivian et al., 2017 | Y / Y | Y / Y | Y / Y | Not applicable / Not applicable | Y / Y | Y / Y | Y / Y | Y / Y | Low |
| Salinas-Rodriguez et al., 2018 | N / N | Y / Y | Y / Y | Not applicable / Not applicable | Y / Y | Y / Y | Y / Y | Y / Y | Low |
| Laccarino et al., 2021 | N / N | Y / Y | Y / Y | Not applicable / Not applicable | Y / Y | Y / Y | Y / Y | Y / Y | Low |
| Z. Li et al., 2022 | Y / Y | Y / Y | Y / Y | Not applicable / Not applicable | Y / Y | Y / Y | Y / Y | Y / Y | Low |
| Sun et al., 2008 | N / N | Y / Y | N / N | Not applicable / Not applicable | Y / Y | Y / Y | Y / Y | Y / Y | Middle |
| Gale et al., 2020 | Y / Y | Y / Y | Y / Y | Not applicable / Not applicable | Y / Y | Y / Y | Y / Y | Y / Y | Low |
| Gatto et al., 2014 | Y /Y | Y /Y | Y / Y | Not applicable / Not applicable | Y / Y | Y / Y | Y / Y | Y / Y | Low |
| Christensen et al., 2022 | Y / Y | Y / Y | Y / Y | Not applicable / Not applicable | Y / Y | Y / Y | Y / Y | Y / Y | Low |

*Results are listed based on an assessment by YL/JH up until Z. Li et al., 2022; and by YL/DL for Sun et al., 2008 to Christensen et al., 2022.

**Table S3. Bias Assessment for the Case-Control Studies**

|  | Were the groups comparable other than the presence of disease in cases or the absence of disease in controls? | Were cases and controls matched appropriately? | Were the same criteria used for identification of cases and controls? | Was exposure measured in a standard, valid and reliable way? | Was exposure measured in the same way for cases and controls? | Were confounding factors identified? | Were strategies to deal with confounding factors stated? | Were outcomes assessed in a standard, valid and reliable way for cases and controls? | Was the exposure period of interest long enough to be meaningful? | Was appropriate statistical analysis used? | Bias Score |
| --- | --- | --- | --- | --- | --- | --- | --- | --- | --- | --- | --- |
| Wu et al., 2015 | Y / Y | Y / Y | Y / Y | Y / Unclear | Y / Y | Y / Y | Y / Y | Y / Unclear | Y / Y | Y / Y | Low |

*Results are listed based on an assessment by YL/JH

**Table S4. Summary of effect modifiers of the included studies**

| **Studies** | **Effect Modifier (Study focus)** | | | | | | | | | | | | | | | | | | | |
| --- | --- | --- | --- | --- | --- | --- | --- | --- | --- | --- | --- | --- | --- | --- | --- | --- | --- | --- | --- | --- |
|  | **Health Behaviors** | | | | **Genetic /Molecular** | **Environmental** | | | | **Individual** | | | | | | | | | | |
|  | Physical  Activity | Smoking Status | Drinking Status | Dietary Diversity |  | Neighborhood Stressor | Noise | Cooking Fuel | Ventilation  in  indoor cooking | Gender | Employment Status | Race | Age | Education | BMI | Geographic location | Marital  Status | Socio-Economic Status  (SES) | Co-morbidity | Others |
| Schikowski et al., 2015 |  |  |  |  | X |  |  |  |  |  |  |  |  |  |  |  |  |  |  |  |
| Fehsel et al., 2016 |  |  |  |  | X |  |  |  |  |  |  |  |  |  |  |  |  |  |  |  |
| Ailshire et al., 2017 |  |  |  |  |  | X |  |  |  |  |  |  |  |  |  |  |  |  |  |  |
| Ailshire et al., 2021 |  |  |  |  |  |  |  |  |  |  |  |  |  | X |  |  |  |  |  |  |
| Cacciottolo et al., 2017 |  |  |  |  | X |  |  |  |  |  |  |  |  |  |  |  |  |  |  |  |
| Cleary et al., 2018 |  |  |  |  | X |  |  |  |  |  |  |  |  |  |  |  |  |  |  |  |
| Colicino et al., 2014 |  |  |  |  | X |  |  |  |  |  |  |  |  |  |  |  |  |  |  |  |
| Colicino et al., 2016 |  |  |  |  | X |  |  |  |  |  |  |  |  |  |  |  |  |  |  |  |
| Colicino et al., 2017 |  |  |  |  | X |  |  |  |  |  |  |  |  |  |  |  |  |  |  |  |
| Hedges et al., 2019 |  |  |  |  |  |  |  |  |  | X |  |  | X | X |  |  |  |  | X |  |
| Salinas-Rodriguez et al., 2018 |  | X | X |  |  |  |  | X | X | X |  |  | X | X |  |  |  |  | X |  |
| Tzivian, Dlugaj, Winkler, Weinmayr, et al., 2016 |  | X | X |  | X |  |  |  |  | X |  |  |  | X | X |  |  |  | X |  |
| Tzivian, Dlugaj, Winkler, Hennig, et al., 2016 |  | X | X |  | X |  | X |  |  | X |  |  | X | X | X |  |  |  | X |  |
| Wu et al.,2015 |  |  |  |  | X |  |  |  |  | X |  |  |  |  |  |  |  |  |  |  |
| Ailshire & Crimmins, 2014 |  | X |  |  |  |  |  |  |  | X | X | X | X | X |  |  |  | X |  |  |
| Yao et al., 2021 | X | X | X | X |  |  |  | X | X | X |  |  | X | X |  | X |  | X | X |  |
| Tzivian et al., 2017 |  |  |  |  |  |  | X |  |  |  |  |  |  |  |  |  |  |  |  |  |
| Kim et al., 2019 |  |  |  |  |  |  |  |  |  | X |  |  |  |  |  |  |  |  |  |  |
| Carey et al., 2018 |  | X |  |  |  |  |  |  |  | X |  |  | X |  |  | X |  | X | X |  |
| C. Chen et al., 2022 |  |  |  | X |  |  |  |  |  |  |  |  |  |  |  |  |  |  |  |  |
| He et al., 2022 |  |  |  | X |  |  |  |  |  |  |  |  |  |  |  |  |  |  |  |  |
| Loop et al., 2013 |  |  |  |  |  |  |  |  |  |  |  |  |  |  |  | X |  |  |  |  |
| Shin et al., 2019 | X | X | X |  |  |  |  |  |  | X |  |  | X | X |  | X |  | X |  |  |
| Oudin et al., 2019 |  |  |  |  | X |  |  |  |  | X |  |  |  |  |  |  |  |  |  |  |
| Chen et al., 2021 |  |  |  | X |  |  |  |  |  |  |  |  |  |  |  |  |  |  |  |  |
| Laccarino et al., 2021 |  |  |  |  |  |  |  |  |  | X |  |  |  |  |  |  |  |  |  |  |
| Z. Li et al., 2022 |  |  |  |  |  |  |  |  |  |  |  |  |  |  |  |  |  | X |  |  |
| Tallon et al., 2017 | X | X |  |  |  |  |  |  |  |  |  |  | X |  | X |  |  |  | X |  |
| Parra et al., 2022 |  |  |  |  | X |  |  |  |  |  |  |  |  |  |  |  |  |  |  |  |
| Alemany et al., 2021 |  |  |  |  | X |  |  |  |  |  |  |  |  |  |  |  |  |  |  | Amyloid status |
| Yu et al., 2020 |  |  |  |  |  |  |  |  |  |  |  |  |  |  |  |  |  |  | X |  |
| Ran et al., 2021 |  |  |  |  |  |  |  |  |  | X |  |  | X |  | X |  |  |  | X |  |
| Grande et al., 2021 |  |  |  |  |  |  |  |  |  |  |  |  |  |  |  |  |  |  | X |  |
| Sun et al., 2008 |  |  |  |  |  |  |  |  |  |  |  |  |  |  |  |  |  | X |  |  |
| Power et al., 2013 |  |  |  |  | X |  |  |  |  |  |  |  |  |  |  |  |  |  |  |  |
| Zhu et al., 2022 |  |  |  | X |  |  |  |  |  |  |  |  |  |  |  |  |  |  |  |  |
| Mortamais et al., 2021 |  |  |  |  | X |  |  |  |  | X |  |  |  | X |  |  |  | X |  |  |
| Cullen et al., 2018 | X |  |  |  |  |  |  |  |  |  |  |  |  |  |  |  |  |  |  |  |
| Christensen et al., 2022 |  |  |  |  |  |  |  |  |  |  |  |  |  |  |  |  |  | X |  |  |
| Shi et al., 2021 |  |  |  |  |  |  |  |  |  | X |  | X | X |  |  | X |  | X |  |  |
| Ma et al., 2022 |  |  |  |  | X |  |  |  |  |  |  |  |  |  |  |  |  |  |  |  |
| Kulick et al., 2020 |  | X |  |  | X |  |  |  |  | X |  | X | X |  |  |  |  |  |  |  |
| Zhang et al., 2023 |  |  |  |  | X |  |  |  |  |  |  |  |  |  |  |  |  |  |  |  |
| Yang et al., 2022 |  |  |  |  |  |  |  |  |  | X | X |  | X | X | X |  | X |  |  |  |
| Hedges et al., 2020 |  |  |  |  |  |  |  |  |  | X |  |  | X | X |  |  |  |  | X |  |
| Gatto et al., 2014 |  |  |  |  |  |  |  |  |  | X |  |  | X |  |  |  |  |  |  |  |
| Gale et al., 2020 |  |  |  |  |  |  |  |  |  | X |  |  | X | X |  |  |  |  | X |  |
| Lucht et al., 2022 |  |  |  |  |  |  |  |  |  |  |  |  | X |  |  |  |  |  |  |  |
| Shaffer et al., 2021 |  |  |  |  | X |  |  |  |  | X |  |  | X | X | X |  |  |  |  |  |
| Cerza et al., 2019 |  |  |  |  |  |  |  |  |  | X |  |  | X | X |  |  |  | X |  |  |
| Wang et al., 2022 |  |  |  |  | X |  |  |  |  |  |  |  | X | X | X | X |  |  | X |  |
| Gao et al., 2022 | X | x | X |  |  |  |  |  |  | X |  |  | X | X |  | X |  |  | X | PM2.5 exposure |
| G.-C. Chen et al., 2022 |  | X |  |  | X |  |  |  |  | X |  |  | X |  | X |  |  |  | X |  |
| Wyatt et al., 2023 |  |  |  |  |  |  |  |  |  |  |  |  | X |  |  |  |  |  |  | Game stage, speed to complete 20 games, user behavior |
| deCrom et al., 2022 |  |  |  |  | X |  |  |  |  |  |  |  | X |  |  | X |  |  |  |  |
| Lee et al., 2022 | X | X | X |  |  |  |  |  |  | X |  |  | X |  |  |  |  |  |  |  |
| Wellenius et al., 2012 |  |  |  |  |  |  |  |  |  |  |  |  | X | X |  |  |  |  |  |  |
| Crous-Bou et al., 2020 |  |  |  |  | X |  |  |  |  | X |  |  | X |  |  |  |  |  | X |  |
| Chen et al., 2020 |  |  |  |  | X |  |  |  |  | X |  |  |  | X |  |  |  |  |  |  |
| Younan et al., 2020 |  |  |  |  |  |  |  |  |  |  |  |  | X |  | X |  |  |  | X |  |
| Ranft et al., 2009 |  |  |  |  |  |  |  |  |  |  |  |  | X |  |  | X |  |  |  | Air pollution |
| Wang et al., 2020 | X | X | X |  |  |  |  |  |  | X |  |  | X | X |  | X |  | X | X |  |
| Chen et al., 2017 |  |  |  |  |  |  |  |  |  | X |  |  |  |  |  |  |  |  | X |  |
| Shi et al., 2023 |  |  |  |  |  |  |  |  |  | X |  | X | X |  |  |  |  | X |  |  |
| Hu et al., 2022 |  |  |  |  |  |  |  |  |  | X |  |  | X | X |  |  | X |  |  |  |
| Power et al., 2011 |  | X |  |  |  |  |  |  |  |  |  |  |  |  | X |  |  |  | X |  |
| Chen et al., 2015 |  |  |  |  |  |  |  |  |  |  |  |  |  |  | X |  |  |  | X |  |
| Semmens et al., 2022 |  |  |  | X | X |  |  |  |  | X |  |  |  |  |  | X |  |  | X |  |
| Yuchi et al., 2020 |  |  |  |  |  |  |  |  |  | X |  |  | X |  |  |  |  |  |  |  |
| M. Li et al., 2022 |  | X |  |  | X |  |  |  |  | X | X |  | X |  | X | X |  |  |  |  |
| Hu et al., 2023 |  |  |  |  |  |  |  |  |  |  |  |  | X |  |  |  |  | x |  |  |
| Lee et al., 2019 |  |  |  |  |  |  |  |  |  |  |  |  | X |  |  | X |  |  |  |  |
| Nunez et al., 2021 |  |  |  |  |  |  |  |  |  | X |  |  | X |  |  | X |  |  |  |  |
| Shi et al., 2020 |  |  |  |  |  |  |  |  |  | X |  | X | X |  |  | X |  | X |  |  |
| % of reporting individual EM out of all papers (%) | 9.46 (7/74) | 20.27 (15/74) | 10.81 (8/74) | 8.11 (6/74) | 35.14 (26/74) | 1.35 (1/74) | 2.70 (2/74) | 2.70 (2/74) | 2.70 (2/74) | 48.65 (36/74) | 4.05 (3/74) | 6.76 (5/74) | 50.00 (37/74) | 27.03 (20/74) | 16.22 (12/74) | 20.27 (15/74) | 2.70 (2/74) | 18.92 (14/74) | 29.73 (22/74) | 5.41 (4/74) |
| % of studies reporting EM in each factor out of all papers (%) | 28.38 (21/74) | | | |  | 6.76 (5/74) | | | | 74.32 (55/74) | | | | | | | | | | |
