# Supplementary material for: A critical review of potential modifiers of air pollutant associations with dementia and related outcomes
Source: Neurotoxicology. Author manuscript; Available in PMC 2026 Jul 7. (PMC13339730; doi:10.1016/j.neuro.2026.103470)
Supplement: Supplementary materials 01 [file NIHMS2188061-supplement-Supplementary_materials_01.docx]

**Supplemental Materials**

A Critical Review of Potential Effect Modifiers of Air Pollution on Dementia and Related Outcomes

**Table of Contents**

**Appendix S1:** Study selection criteria

**Appendix S2:** Search sequence and terms

**Appendix S3:** Study Characteristics

**Figure S1:** PRISMA flowchart for identifying eligible articles for inclusion in this systematic review

**Figure S2a**: Summary of associations between air pollution exposure and ADRD by individual-level modifiers in qualifying studies

- - Figure S2a.1.1: Summary of coefficients between types of air pollution exposure (unit) and cognitive function by age in qualifying studies
  - Figure S2a.1.2: Summary of logistic regression between types of air pollution exposure (unit) and cognitive function by age in qualifying studies
  - Figure S2a.1.3: Summary of logistic regression between types of air pollution exposure (unit) and dementia by age in qualifying studies
  - Figure S2a.2.1: Summary of the coefficient between types of air pollution exposure (unit) and cognitive test by gender in qualifying studies
  - Figure S2a.2.2: Summary of logistic regression between types of air pollution exposure (unit) and cognitive test by gender in qualifying studies
  - Figure S2a.2.3: Summary of logistic regression between types of air pollution exposure (unit) and dementia by gender in qualifying studies
  - Figure S2a.3.1: Summary of logistic regression between types of air pollution exposure (unit) and dementia by BMI in qualifying studies
  - Figure S2a.4.1: Summary of logistic regression between types of air pollution exposure (unit) and cognitive test by education in qualifying studies
  - Figure S2a.5.1: Summary of logistic regression between types of air pollution exposure (unit) and dementia by diabetes in qualifying studies
  - Figure S2a.5.2: Summary of logistic regression between types of air pollution exposure (unit) and dementia by hypertension in qualifying studies

**Figure S2b**: Summary of associations between air pollution exposure and ADRD by health behaviors-level modifiers in qualifying studies

- - Figure S2b.1.1: Summary of coefficients between types of air pollution exposure (unit) and cognitive function by drinking status in qualifying studies
  - Figure S2b.1.2: Summary of logistic regression between types of air pollution exposure (unit) and cognitive function by drinking status in qualifying studies
  - Figure S2b.2.1: Summary of coefficients between types of air pollution exposure (unit) and cognitive function by smoking status in qualifying studies
  - Figure S2b.2.2: Summary of logistic regression between types of air pollution exposure (unit) and cognitive function by smoking status in qualifying studies
  - Figure S2b.2.3: Summary of logistic regression between types of air pollution exposure (unit) and dementia by physical activity in qualifying studies

**Figure S2c**: Summary of associations between air pollution exposure and ADRD by genetic/molecular modifiers in qualifying studies

- - Figure S2c.1.1: Summary of logistic regression between types of air pollution exposure (unit) and Alzheimer's Disease by APOE e4 status in qualifying studies
  - Figure S2c.1.2: Summary of logistic regression between types of air pollution exposure (unit) and Alzheimer's Disease by APOE e4 status in qualifying studies

**References**

**Appendix S1: Study selection criteria**

Inclusion criteria:

1. English-written literature

2. Epidemiological studies or population-based studies

3. Adults (18 years or older)

4. Reported the results of potential effect modifiers of air pollution on dementia and related outcomes

5. Did not require the presence of specific diseases at the time of study recruitment

6. Long-term exposure to ambient air pollution for more than a 1-year averaging period

7. Air pollution particles should include one or more of the following list; ambient outdoor ozone (O3), sulfur dioxide (SO2), nitrogen dioxide (NO2), oxides of nitrogen (NOx), particulate matter (PM), including respirable particles less than 10 mm (PM10), PM2.5–10, or fine particles less than 2.5 mm (PM2.5), and/or traffic-related air pollution, including accepted surrogates for PM or traffic-related pollution such as distance to road, soot, or black carbon;

8. reported one of the following cognitive functions as health outcomes (cognitive test scores, change in cognitive test scores), diagnosis of dementia, mild cognitive impairment, dementia subtypes (i.e., Alzheimer's disease), neuroimaging features associated with dementia, or progression of neuroimaging features associated with dementia

Exclusion criteria:

1. Review articles, commentary, animal or molecular experimental studies, conference abstracts, or non-peer review publications.

2. Exposure is based on an indoor setting

**Appendix S2: Search Sequence and Terms**

Embase and Medline through Ovid Search Sequence and Terms (conducted on 1 February, 2024)

1. (Alzheimer* or dementia or mild cognitive impairment* or cognitive decline or cognitive impairment* or impaired cognition or impaired cognitive function or cognitive change or neurocognitive disorder* or mild neurocognitive disorder* or ADRD or cognitive dysfunction* or ).ti,ab.
2. (air pollut* or particulate matter or particulate matter* or nitrogen dioxide or nitrogen oxides or ozone or sulfur dioxide or volatile organic or carbon monoxide or "PM2.5?10" or PM* or coarse particulate matter or PM10 or "PM2.5" or traffic?related pollution or traffic pollution or vehicle pollution or car pollution or black carbon or soot or O3 or ambient fine particles).ti,ab.
3. #1 AND #2

PubMed Search Sequence and Terms (conducted on 1 February 2024)

1. (((((((((((("Alzheimer Disease"[Mesh]) OR (dementia)) OR (alzheimer*)) OR (alzheimers)) OR (alzheimer's)) OR ("Cognitive Dysfunction"[Mesh])) OR (cognitive decline[Text Word])) OR (cognitive declines[Text Word])) OR (neuropsychology)) OR (cognit*)) OR (cognitive impairment)) OR (impaired cognition)) OR (cognitive change)

2. (((((((((((((("Air Pollution"[Mesh]) OR (particulate matter)) OR (nitrogen dioxide)) OR (ozone)) OR (sulfur dioxide)) OR (volatile organic)) OR (carbon monoxide)) OR (PM10)) OR (PM2.5)) OR (traffic-related)) OR (air pollution)) OR (particulate matter)) OR (ozone)) OR (nitrogen dioxide)) OR (black carbon) OR (Nitrogen Oxides[MeSH Terms]) OR (oxides of nitrogen) OR (NOx) OR (coarse particulate matter) OR (PM2.5?10) OR (air pollut*) OR (particulate matter*) OR (PM*) OR (traffic?related pollution) OR (traffic pollution) OR (vehicle pollution) OR (car pollution) OR (soot) or (O3) or (ambient fine particles)

3. 1 AND 2

**Appendix S3: Study characteristics**

*3.2.1 Study Designs*

The majority of studies employed the **cohort design** (n=55) followed by **cross-sectional** (n= 18) and **case-control** (n=1) study designs. A list of included studies, categorized by study design, is available in Tables **S3-S5**.

*3.2.2 Geographic settings*

The selected studies were conducted in various locations worldwide. Most of the studies included in our review focused on nationwide settings, whereas several studies were set in a specific urbanized area. The geographic settings of all selected studies included the **United States** (Ailshire et al. 2017; Ailshire and Walsemann 2021; Ailshire and Crimmins 2014; Cacciottolo et al. 2017; Chen et al. 2021; C Chen et al. 2022; Christensen et al. 2022; Colicino et al. 2014; Colicino et al. 2016; Colicino et al. 2017; Gatto et al. 2014; Iaccarino et al. 2021; Kulick et al. 2020; Z Li et al. 2022; Loop et al. 2013; Power et al. 2013; Shaffer et al. 2021; Shi et al. 2020; Shi et al. 2021; Shi et al. 2023; Tallon et al. 2017; Wang et al. 2022; Wellenius et al. 2012; Wyatt et al. 2023; Younan et al. 2021; Yu et al. 2020), **Germany** (Fehsel et al. 2016; Lucht et al. 2022; Ranft et al. 2009; Schikowski et al. 2015; Tzivian et al. 2016a; Tzivian et al. 2016b), **UK** (Carey et al. 2018; G-C Chen et al. 2022; Cullen et al. 2018; Gale et al. 2020; Hedges et al. 2019; Hedges et al. 2020; Ma et al. 2022; Parra et al. 2022; Zhang et al. 2023), **South Korea** (Lee et al. 2022; Shin et al. 2019) , **China** (Gao et al. 2022; He et al. 2022; Hu et al. 2023; Hu et al. 2022; Sun and Gu 2008; Wang et al. 2020; Yang et al. 2022; Yao et al. 2021; Zhu et al. 2022), **Sweden** (Grande et al. 2021; Oudin et al. 2019), **Spain** (Alemany et al. 2021; Crous-Bou et al. 2020), **Taiwan** (Chen et al. 2020; Wu et al. 2015), Netherlands (de Crom et al. 2022), **Italy** (Cerza et al. 2019), **France** (Mortamais et al. 2021) or **Canada** (Chen et al. 2017). Details are further provided in **Table S2**.

*3.2.3 Air pollution exposures*

The majority of studies reported on associations with particular matter (PM) by their sizes in micrometers, including less than 2.5 micrometers (**PM_2.5_**), between 2.5 and 10 micrometers (**PM_coarse_**), and less than 10 micrometers (**PM_10_**) (Ailshire et al. 2017; Ailshire and Walsemann 2021; Ailshire and Crimmins 2014; Alemany et al. 2021; Cacciottolo et al. 2017; Carey et al. 2018; Cerza et al. 2019; Chen et al. 2021; C Chen et al. 2022; G-C Chen et al. 2022; Chen et al. 2017; Chen et al. 2015; Chen et al. 2020; Christensen et al. 2022; Cleary et al. 2018; Crous-Bou et al. 2020; Cullen et al. 2018; de Crom et al. 2022; Fehsel et al. 2016; Gale et al. 2020; Gatto et al. 2014; Grande et al. 2021; He et al. 2022; Hedges et al. 2019; Hedges et al. 2020; Hu et al. 2023; Hu et al. 2022; Iaccarino et al. 2021; Kim et al. 2019; Kulick et al. 2020; Lee et al. 2022; Lee et al. 2019; M Li et al. 2022; Z Li et al. 2022; Loop et al. 2013; Lucht et al. 2022; Ma et al. 2022; Mortamais et al. 2021; Nunez et al. 2021; Parra et al. 2022; Ran et al. 2021; Ranft et al. 2009; Salinas-Rodríguez et al. 2018; Schikowski et al. 2015; Semmens et al. 2022; Shaffer et al. 2021; Shi et al. 2020; Shi et al. 2023; Shin et al. 2019; Sun and Gu 2008; Tallon et al. 2017; Tzivian et al. 2016a; Tzivian et al. 2016b; Tzivian et al. 2017; Wang et al. 2020; Wang et al. 2022; Wu et al. 2015; Wyatt et al. 2023; Younan et al. 2021; Yuchi et al. 2020; Zhang et al. 2023; Zhu et al. 2022). Associations with nitrogen oxide (**NO_x_**) and nitrogen dioxide (**NO_2_**) were frequently reported as well (Alemany et al. 2021; Carey et al. 2018; Cerza et al. 2019; G-C Chen et al. 2022; Chen et al. 2017; Christensen et al. 2022; Crous-Bou et al. 2020; Cullen et al. 2018; de Crom et al. 2022; Fehsel et al. 2016; Gale et al. 2020; Gatto et al. 2014; He et al. 2022; Hedges et al. 2019; Hedges et al. 2020; Hu et al. 2022; Kim et al. 2019; Kulick et al. 2020; Z Li et al. 2022; Lucht et al. 2022; Ma et al. 2022; Mortamais et al. 2021; Oudin et al. 2019; Parra et al. 2022; Schikowski et al. 2015; Semmens et al. 2022; Shi et al. 2021; Shin et al. 2019; Sun and Gu 2008; Tallon et al. 2017; Tzivian et al. 2016a; Tzivian et al. 2016b; Tzivian et al. 2017; Wang et al. 2022; Yuchi et al. 2020; Zhang et al. 2023). Other air pollutants such as ozone (**O_3_**) (Carey et al. 2018; Cerza et al. 2019; Chen et al. 2017; Christensen et al. 2022; Gao et al. 2022; Gatto et al. 2014; He et al. 2022; Hu et al. 2022; Iaccarino et al. 2021; Mortamais et al. 2021; Shi et al. 2021; Shin et al. 2019; Sun and Gu 2008; Wu et al. 2015; Wyatt et al. 2023; Yuchi et al. 2020), black carbon (**BC**) (Colicino et al. 2014; Colicino et al. 2016; Colicino et al. 2017; Power et al. 2011; Power et al. 2013), and carbon monoxide (**CO**) (Christensen et al. 2022; He et al. 2022; Z Li et al. 2022; Shin et al. 2019; Sun and Gu 2008) were also studied.

Multiple modelling approaches for air pollution exposure estimation were used across studies, including **land use regression (LUR)** (Alemany et al. 2021; Cerza et al. 2019; G-C Chen et al. 2022; Chen et al. 2017; Colicino et al. 2014; Colicino et al. 2016; Colicino et al. 2017; Crous-Bou et al. 2020; Cullen et al. 2018; de Crom et al. 2022; Fehsel et al. 2016; Gale et al. 2020; Hedges et al. 2019; Hedges et al. 2020; Lucht et al. 2022; Ma et al. 2022; Mortamais et al. 2021; Oudin et al. 2019; Parra et al. 2022; Schikowski et al. 2015; Shaffer et al. 2021; Tzivian et al. 2016a; Tzivian et al. 2016b; Tzivian et al. 2017; Wang et al. 2020; Yuchi et al. 2020; Zhang et al. 2023), **universal kriging models** (Kim et al. 2019; Kulick et al. 2020; Wang et al. 2020), Bayesian maximum entropy (BME) (Cacciottolo et al. 2017; Chen et al. 2021; C Chen et al. 2022; Wu et al. 2015; Younan et al. 2021), **a satellite-based spatial model built with Moderate Resolution Imaging Spectroradiometer** (MODIS) (Hu et al. 2023; Lee et al. 2019; Loop et al. 2013; Salinas-Rodríguez et al. 2018; Yang et al. 2022; Zhu et al. 2022), **dispersion models** (Carey et al. 2018; Grande et al. 2021; M Li et al. 2022; Tallon et al. 2017; Yu et al. 2020), **chemical transport models** (Chen et al. 2017; Christensen et al. 2022; Cleary et al. 2018; Lee et al. 2022; Z Li et al. 2022), and **ensemble models** (Shi et al. 2020; Shi et al. 2021; Shi et al. 2023), Other studies directly used **air pollutant concentrations from monitoring stations** (Ailshire et al. 2017; Ailshire and Crimmins 2014; Gatto et al. 2014; He et al. 2022; Shin et al. 2019; Wyatt et al. 2023), and others used the **proxy exposure of distance from major roads** (Ranft et al. 2009; Sun and Gu 2008; Wellenius et al. 2012; Yao et al. 2021; Yuchi et al. 2020).

Most of the studies relied on air pollution levels **at the participant’s residential address** as individual air pollution exposure estimation (Alemany et al. 2021; Cacciottolo et al. 2017; Carey et al. 2018; Cerza et al. 2019; Chen et al. 2021; C Chen et al. 2022; G-C Chen et al. 2022; Chen et al. 2015; Chen et al. 2020; Colicino et al. 2014; Colicino et al. 2016; Colicino et al. 2017; Crous-Bou et al. 2020; Cullen et al. 2018; de Crom et al. 2022; Fehsel et al. 2016; Gale et al. 2020; Gao et al. 2022; Gatto et al. 2014; Grande et al. 2021; Hedges et al. 2019; Hedges et al. 2020; Hu et al. 2022; Iaccarino et al. 2021; Kim et al. 2019; Kulick et al. 2020; Lee et al. 2022; M Li et al. 2022; Z Li et al. 2022; Loop et al. 2013; Lucht et al. 2022; Ma et al. 2022; Mortamais et al. 2021; Nunez et al. 2021; Oudin et al. 2019; Parra et al. 2022; Power et al. 2011; Power et al. 2013; Ran et al. 2021; Ranft et al. 2009; Schikowski et al. 2015; Semmens et al. 2022; Shaffer et al. 2021; Shi et al. 2020; Tallon et al. 2017; Tzivian et al. 2016a; Tzivian et al. 2016b; Tzivian et al. 2017; Wang et al. 2020; Wang et al. 2022; Wellenius et al. 2012; Wu et al. 2015; Yang et al. 2022; Yao et al. 2021; Younan et al. 2021; Yu et al. 2020; Zhang et al. 2023; Zhu et al. 2022). Some studies used air pollution data **at the census tract level** of the participant’s residential address (Ailshire et al. 2017; Ailshire and Walsemann 2021; Ailshire and Crimmins 2014; Christensen et al. 2022; Salinas-Rodríguez et al. 2018; Wyatt et al. 2023), **ZIP code or postal code** (Chen et al. 2017; Cleary et al. 2018; Lee et al. 2019; Shi et al. 2021; Shi et al. 2023; Yuchi et al. 2020) or a larger geographic resolution such as **county level or city level** (He et al. 2022; Hu et al. 2023).

*3.2.4 ADRD Outcomes*

Most of the selected studies utilized cognitive test scores as the primary outcome. Types of cognitive test scores varied, and included both global or domain-specific cognitive tests. Further detailed information of types of outcomes considered by the selected studies are provided in **Table S2**.

**Figure S1: PRISMA flowchart for identifying eligible articles for inclusion in this systematic review**

**
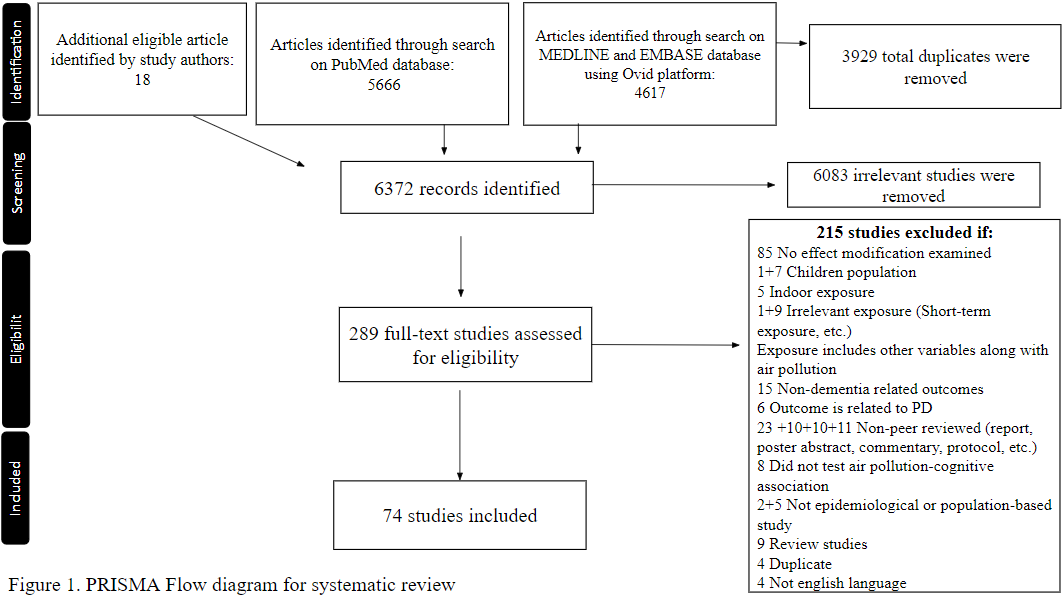
**

**Figure S2a: Summary of associations between air pollution exposure and ADRD by individual-level modifiers in qualifying studies**

- Figure S2a.1.1: Summary of regression coefficients between types of air pollution exposure (unit) and cognitive function by age in qualifying studies


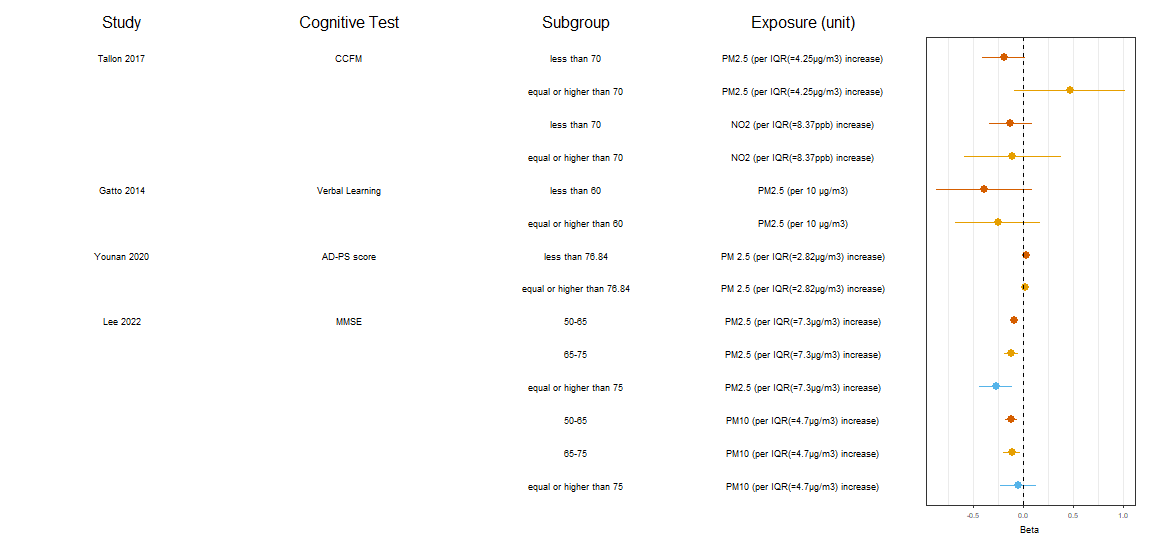


- Figure S2a.1.2: Summary of odds ratios and hazard ratios between types of air pollution exposure (unit) and cognitive function by age in qualifying studies


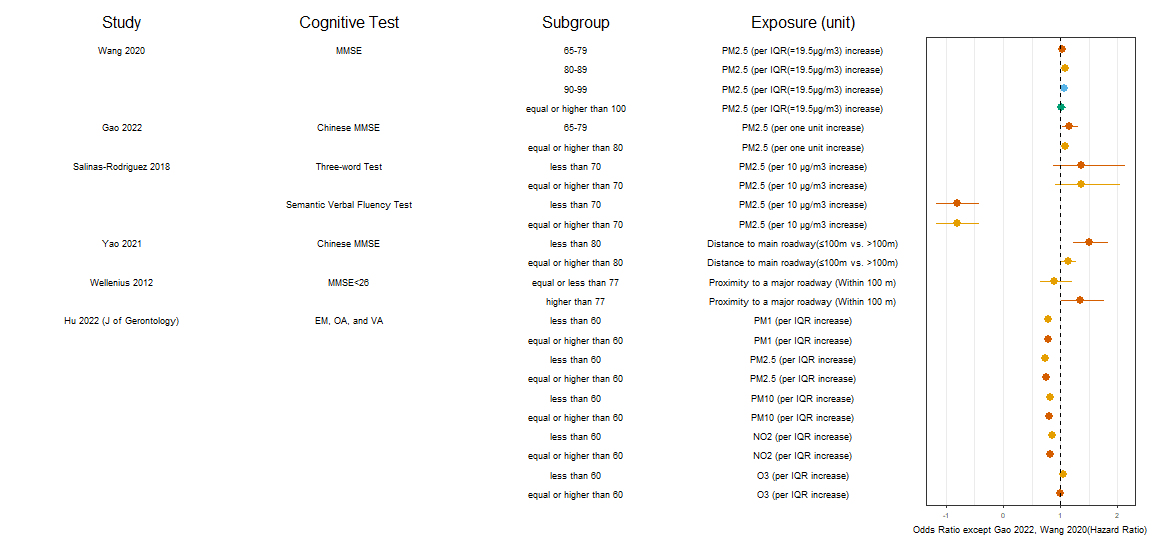


- Figure S2a.1.3: Summary of hazard ratios between types of air pollution exposure (unit) and dementia by age in qualifying studies


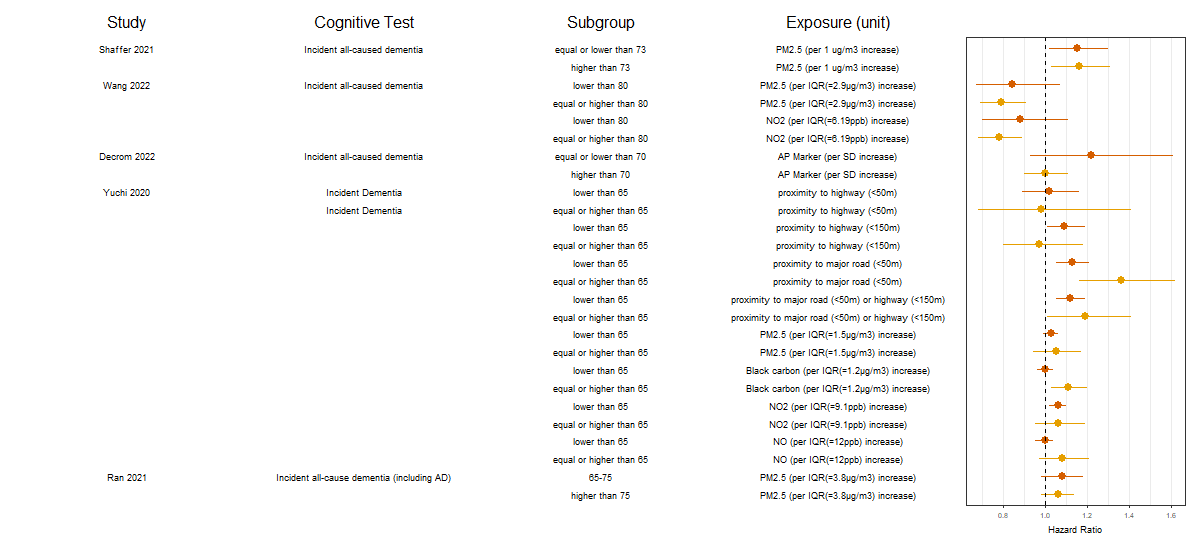


- Figure S2a.2.1: Summary of regression coefficients between types of air pollution exposure (unit) and cognitive test by gender in qualifying studies


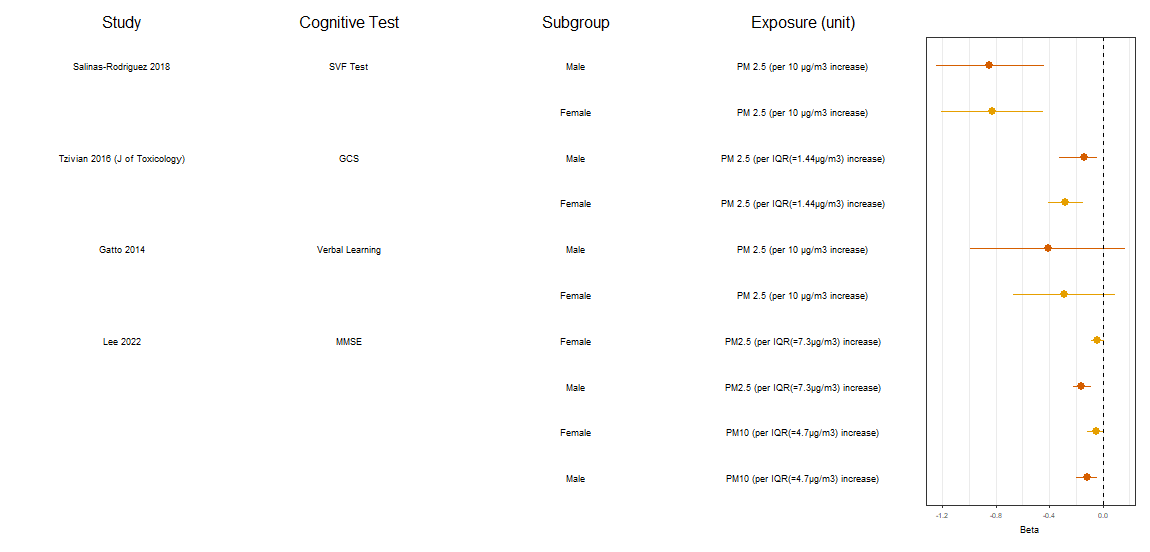


- Figure S2a.2.2: Summary of odds ratios and hazard ratios between types of air pollution exposure (unit) and cognitive test by gender in qualifying studies


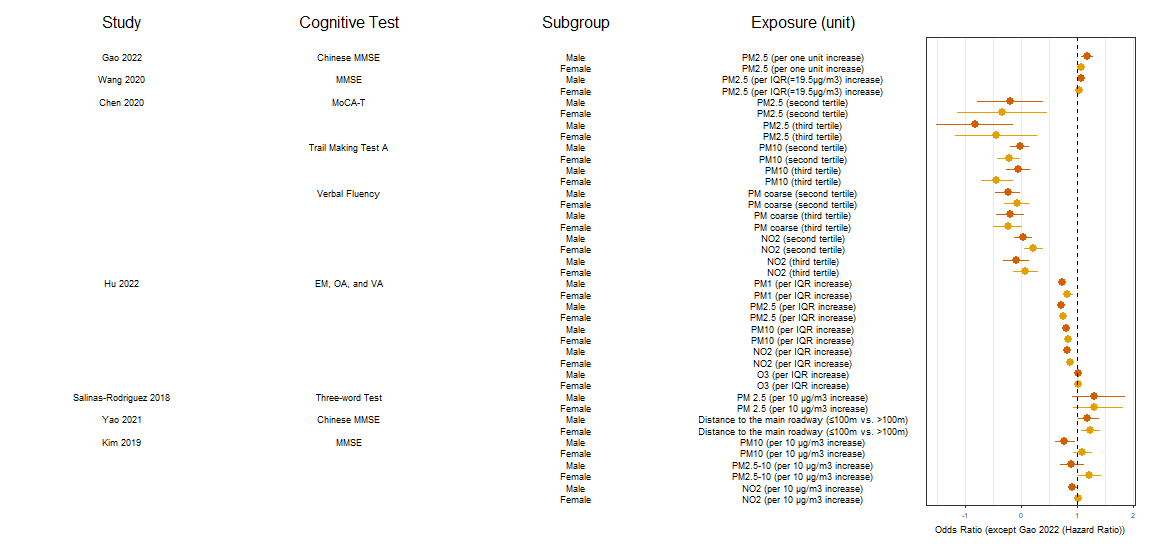


- Figure S2a.2.3: Summary of hazard ratios between types of air pollution exposure (unit) and dementia by gender in qualifying studies


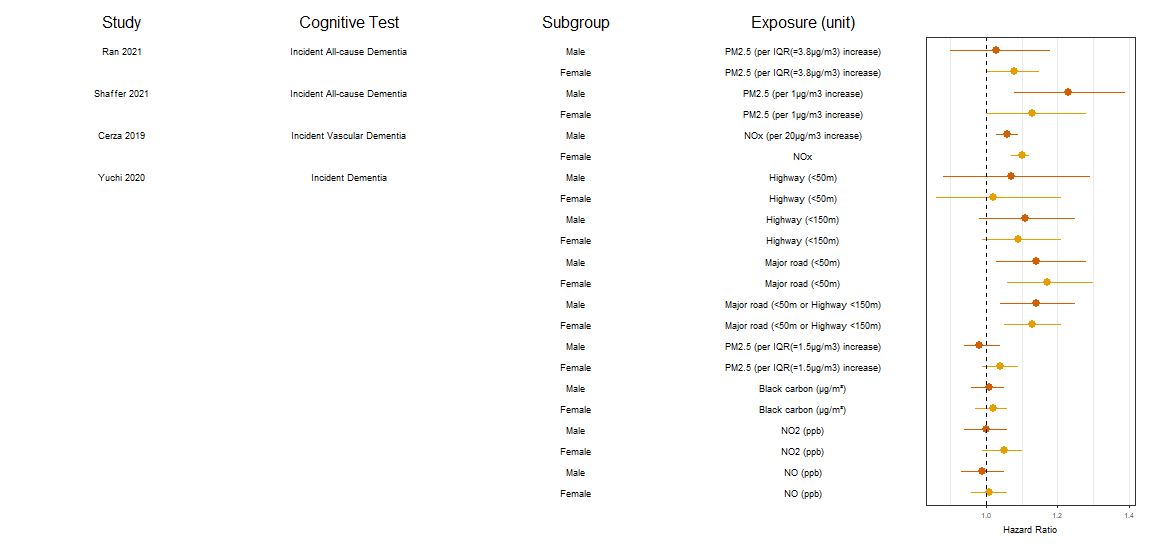


- Figure S2a.3.1: Summary of hazard ratios between types of air pollution exposure (unit) and dementia by BMI in qualifying studies


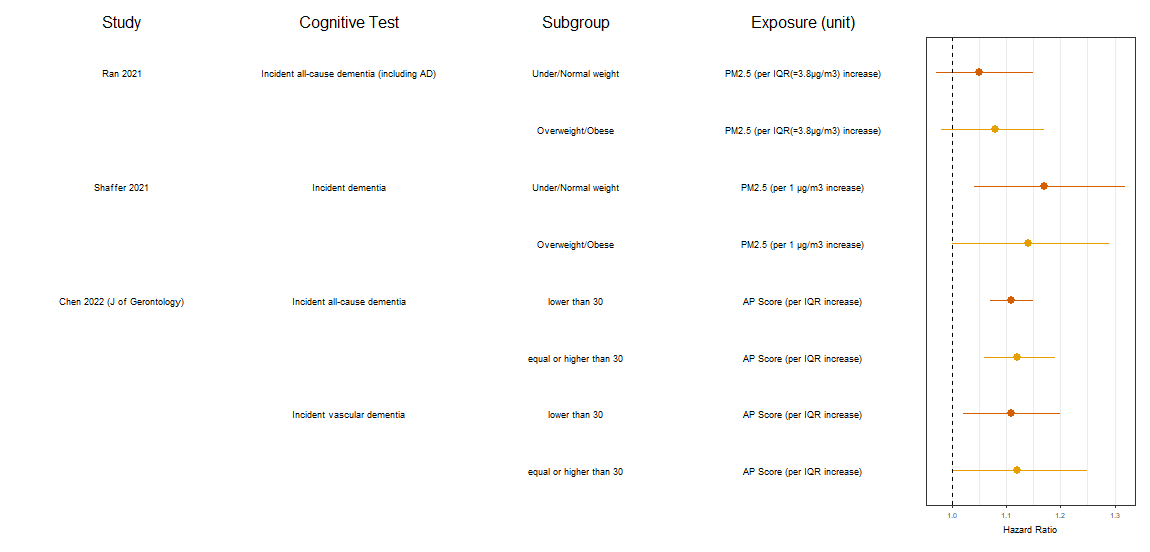


- Figure S2a.4.1: Summary of odds ratios and hazard ratios between types of air pollution exposure (unit) and cognitive test by education in qualifying studies


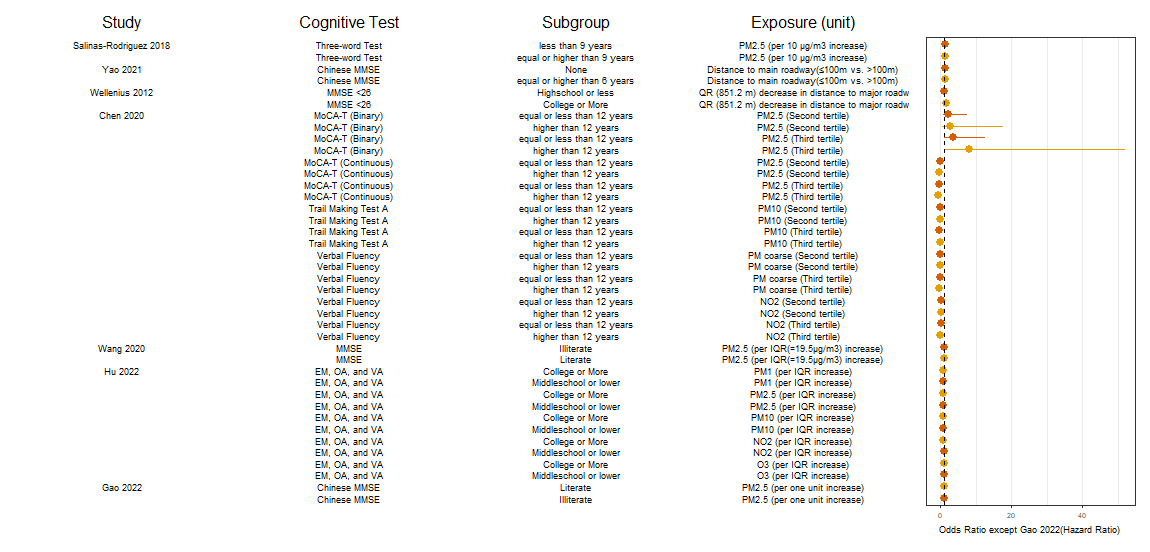


**Figure S2a.5: Summary of associations between air pollution exposure and ADRD by comorbidity in qualifying studies**

- Figure S2a.5.1: Summary of hazard ratios between types of air pollution exposure (unit) and dementia by diabetes in qualifying studies


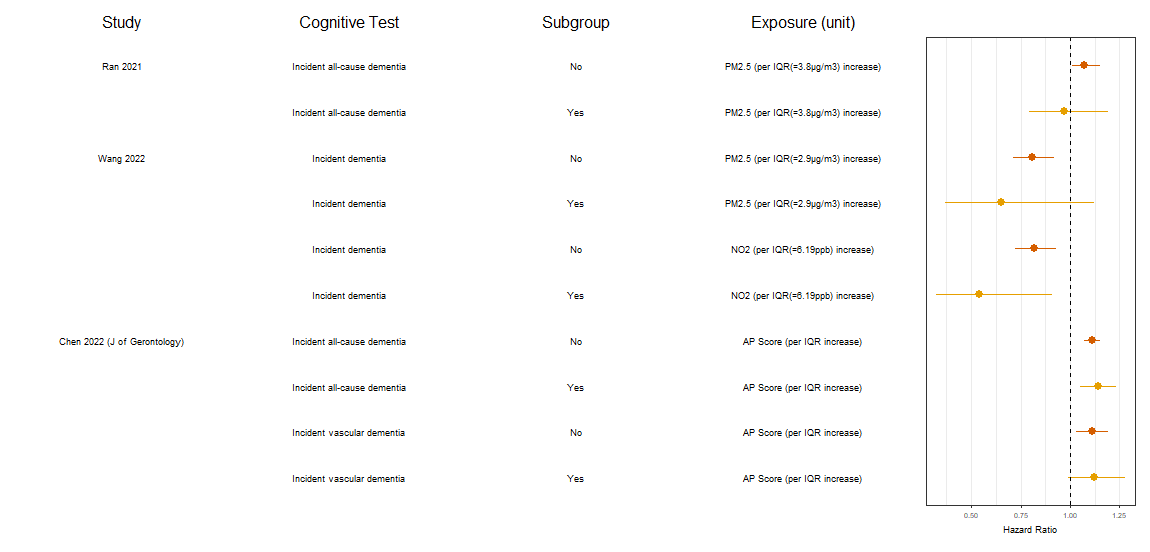


- Figure S2a.5.2: Summary of hazard ratios between types of air pollution exposure (unit) and dementia by hypertension in qualifying studies


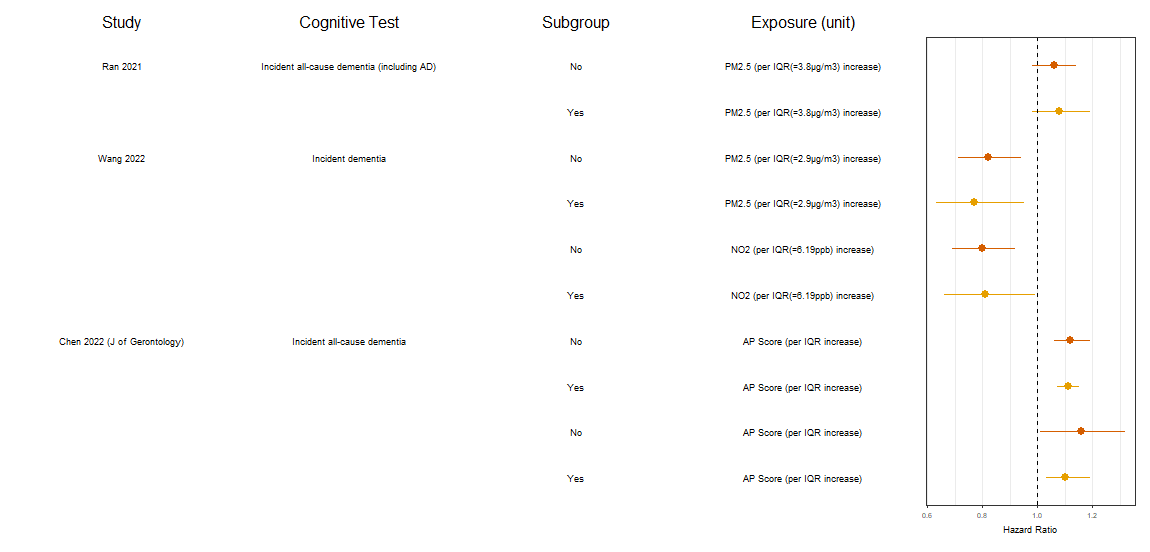


**Figure S2b: Summary of associations between air pollution exposure and ADRD by health behaviors-level modifiers in qualifying studies**

- Figure S2b.1.1: Summary of regression coefficients between types of air pollution exposure (unit) and cognitive function by drinking status in qualifying studies


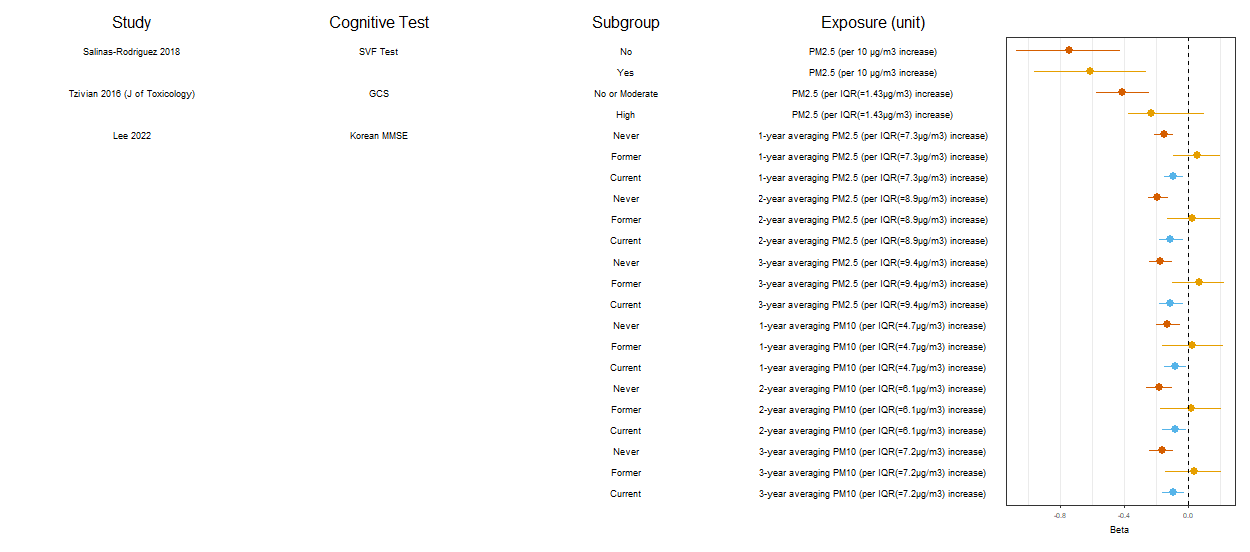


- Figure S2b.1.2: Summary of odds ratios and hazard ratios between types of air pollution exposure (unit) and cognitive function by drinking status in qualifying studies


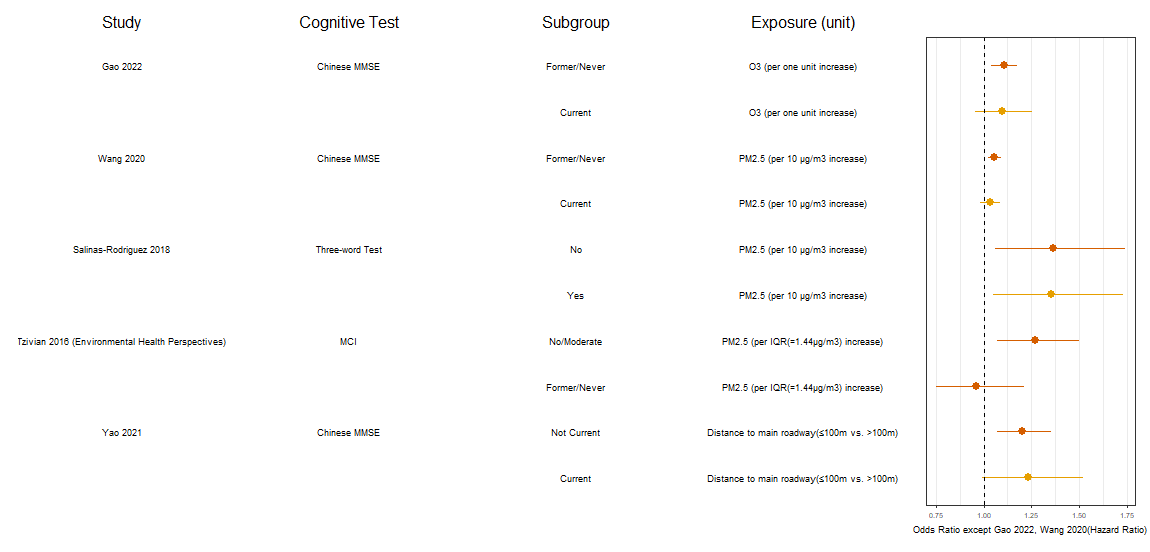


- Figure S2b.2.1: Summary of regression coefficients between types of air pollution exposure (unit) and cognitive function by smoking status in qualifying studies


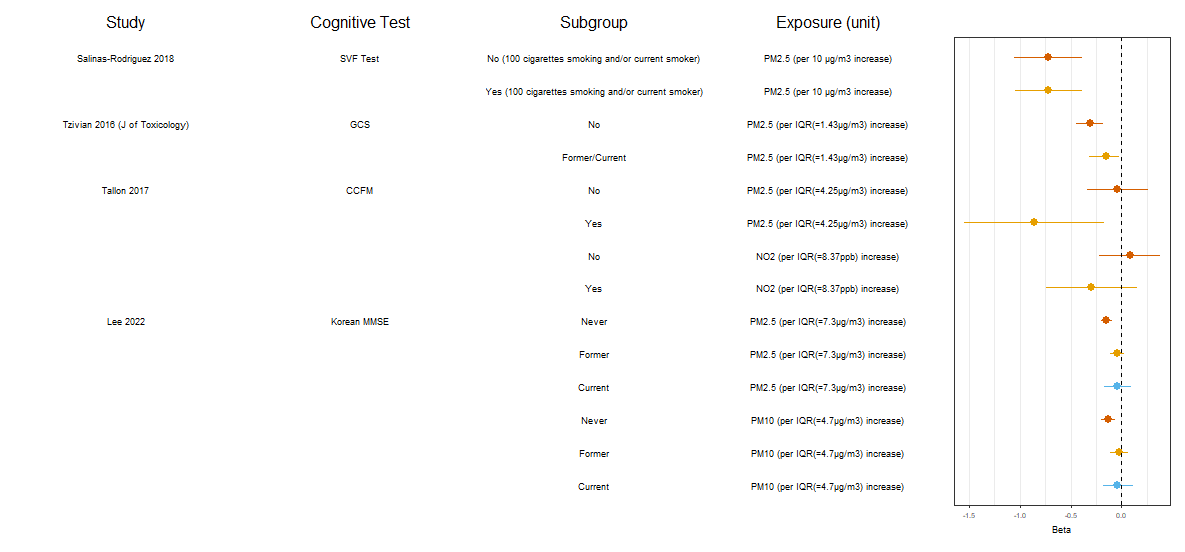


- Figure S2b.2.2: Summary of odds ratios and hazard ratios between types of air pollution exposure (unit) and cognitive function by smoking status in qualifying studies


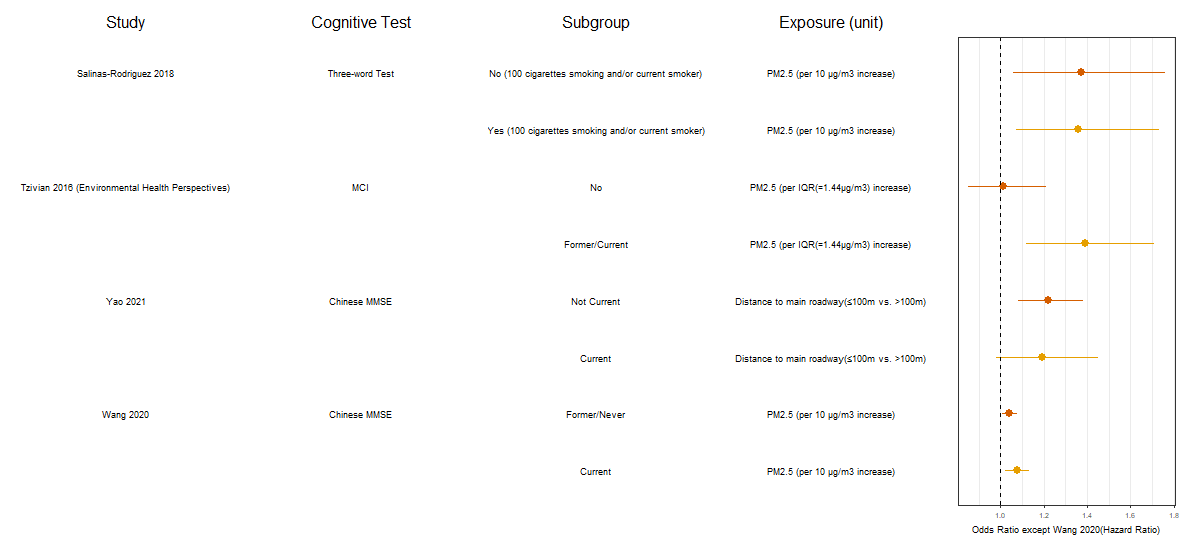


- Figure S2b.2.3: Summary of hazard ratios between types of air pollution exposure (unit) and dementia by physical activity in qualifying studies


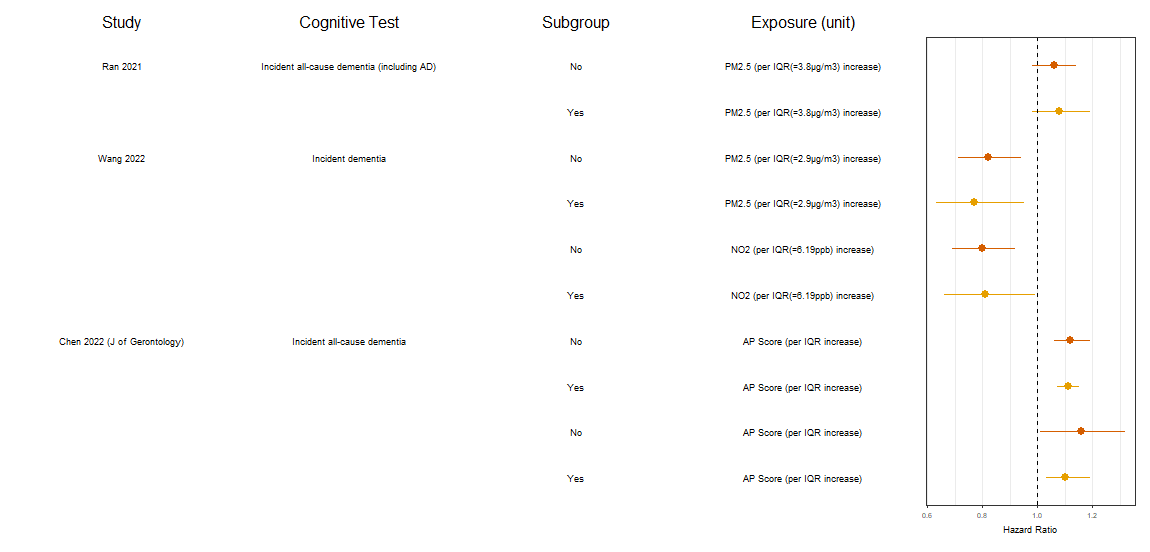


**Figure S2c: Summary of associations between air pollution exposure and ADRD by genetic/molecular modifiers in qualifying studies**

- Figure S2c.1.1: Summary of odds ratios and hazard ratios between types of air pollution exposure (unit) and Alzheimer's Disease by APOE e4 status in qualifying studies


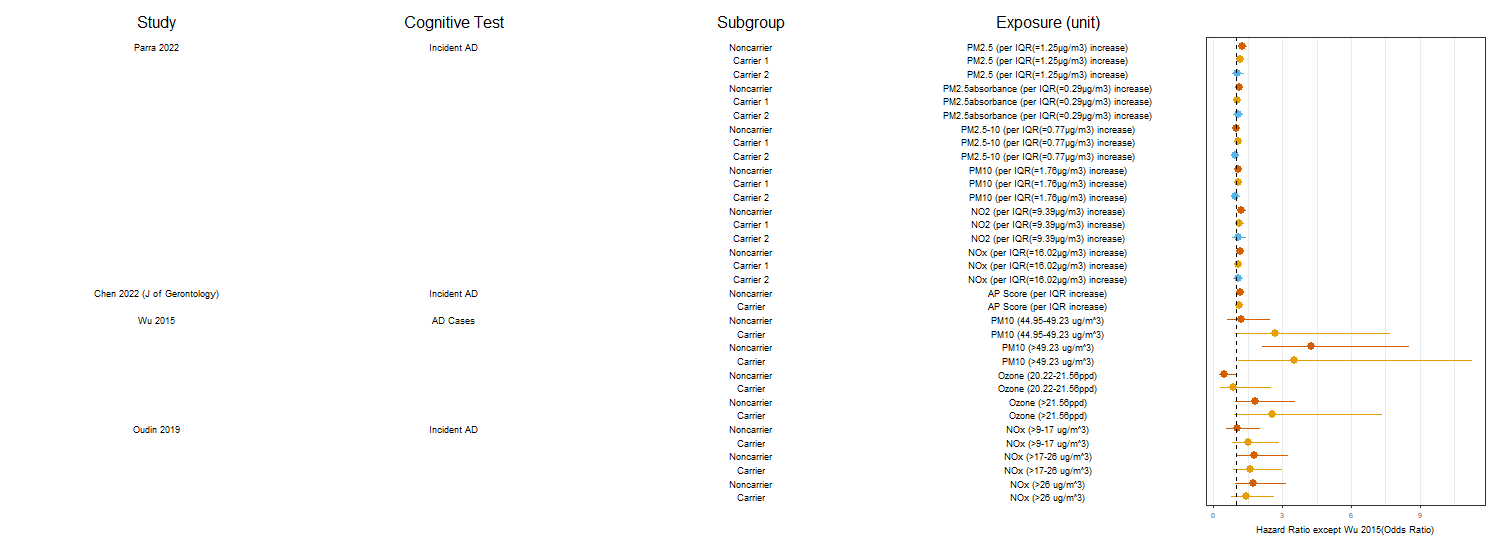


- Figure S2c.1.2: Summary of odds ratios and hazard ratios between types of air pollution exposure (unit) and Dementia by APOE e4 status in qualifying studies


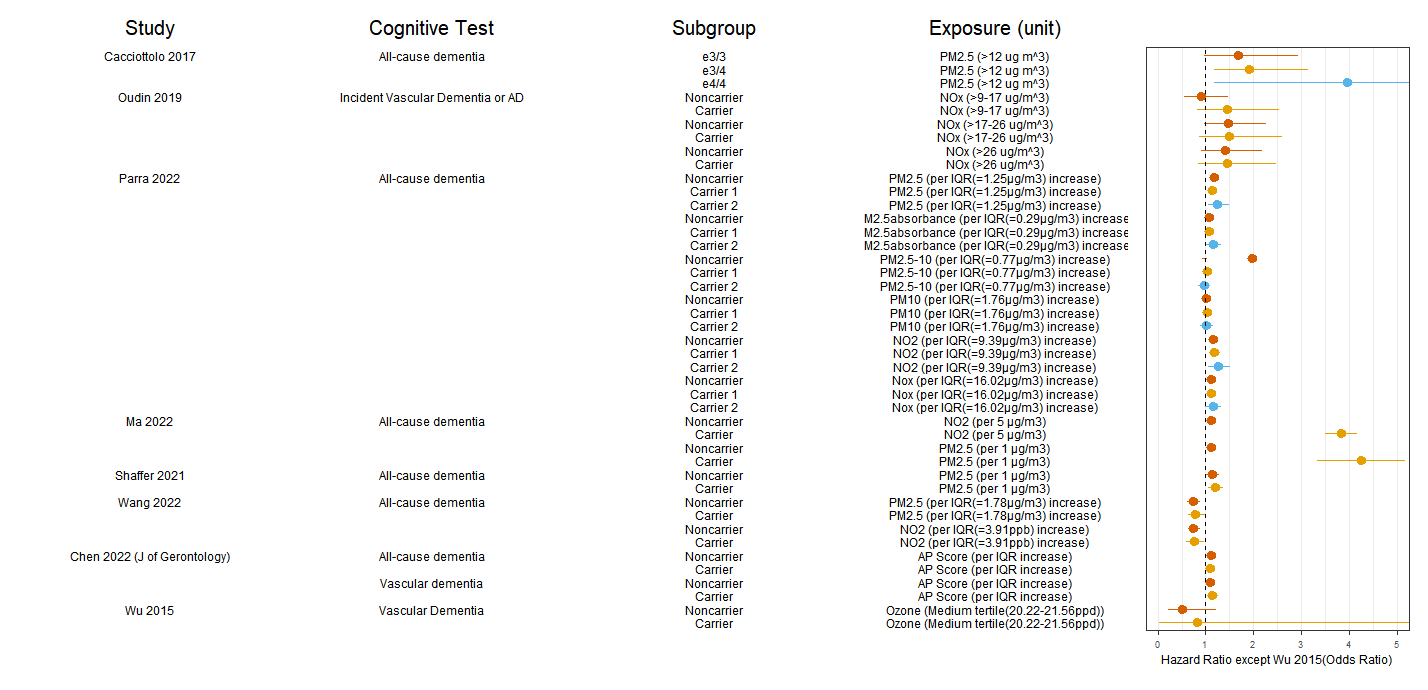


**References**

Ailshire J, Karraker A, Clarke P. 2017. Neighborhood social stressors, fine particulate matter air pollution, and cognitive function among older us adults. Social science & medicine 172:56-63.

Ailshire J, Walsemann KM. 2021. Education differences in the adverse impact of pm 2.5 on incident cognitive impairment among us older adults. Journal of Alzheimer's Disease 79:615-625.

Ailshire JA, Crimmins EM. 2014. Fine particulate matter air pollution and cognitive function among older us adults. Am J Epidemiol 180:359-366.

Alemany S, Crous-Bou M, Vilor-Tejedor N, Milà-Alomà M, Suárez-Calvet M, Salvadó G, et al. 2021. Associations between air pollution and biomarkers of alzheimer's disease in cognitively unimpaired individuals. Environ Int 157:106864.

Cacciottolo M, Wang X, Driscoll I, Woodward N, Saffari A, Reyes J, et al. 2017. Particulate air pollutants, apoe alleles and their contributions to cognitive impairment in older women and to amyloidogenesis in experimental models. Transl Psychiatry 7:e1022.

Carey IM, Anderson HR, Atkinson RW, Beevers SD, Cook DG, Strachan DP, et al. 2018. Are noise and air pollution related to the incidence of dementia? A cohort study in london, england. BMJ open 8:e022404.

Cerza F, Renzi M, Gariazzo C, Davoli M, Michelozzi P, Forastiere F, et al. 2019. Long-term exposure to air pollution and hospitalization for dementia in the rome longitudinal study. Environmental Health 18:1-12.

Chen C, Hayden KM, Kaufman JD, Espeland MA, Whitsel EA, Serre ML, et al. 2021. Adherence to a mind-like dietary pattern, long-term exposure to fine particulate matter air pollution, and mri-based measures of brain volume: The women's health initiative memory study-mri. Environmental health perspectives 129:127008.

Chen C, Whitsel EA, Espeland MA, Snetselaar L, Hayden KM, Lamichhane AP, et al. 2022. B vitamin intakes modify the association between particulate air pollutants and incidence of all‐cause dementia: Findings from the women's health initiative memory study. Alzheimer's & dementia.

Chen G-C, Nyarko Hukportie D, Wan Z, Li F-R, Wu X-B. 2022. The association between exposure to air pollution and dementia incidence: The modifying effect of smoking. The Journals of Gerontology: Series A:glac228.

Chen H, Kwong JC, Copes R, Hystad P, van Donkelaar A, Tu K, et al. 2017. Exposure to ambient air pollution and the incidence of dementia: A population-based cohort study. Environment international 108:271-277.

Chen J-C, Wang X, Wellenius GA, Serre ML, Driscoll I, Casanova R, et al. 2015. Ambient air pollution and neurotoxicity on brain structure: Evidence from women's health initiative memory study. Annals of neurology 78:466-476.

Chen J-H, Kuo T-Y, Yu H-L, Wu C, Yeh S-L, Chiou J-M, et al. 2020. Long-term exposure to air pollutants and cognitive function in taiwanese community-dwelling older adults: A four-year cohort study. Journal of Alzheimer's Disease 78:1585-1600.

Christensen GM, Li Z, Pearce J, Marcus M, Lah JJ, Waller LA, et al. 2022. The complex relationship of air pollution and neighborhood socioeconomic status and their association with cognitive decline. Environment international 167:107416.

Cleary EG, Cifuentes M, Grinstein G, Brugge D, Shea TB. 2018. Association of low-level ozone with cognitive decline in older adults. J Alzheimers Dis 61:67-78.

Colicino E, Power MC, Cox DG, Weisskopf MG, Hou L, Alexeeff SE, et al. 2014. Mitochondrial haplogroups modify the effect of black carbon on age-related cognitive impairment. Environ Health 13:42.

Colicino E, Giuliano G, Power MC, Lepeule J, Wilker EH, Vokonas P, et al. 2016. Long-term exposure to black carbon, cognition and single nucleotide polymorphisms in microrna processing genes in older men. Environ Int 88:86-93.

Colicino E, Wilson A, Frisardi MC, Prada D, Power MC, Hoxha M, et al. 2017. Telomere length, long-term black carbon exposure, and cognitive function in a cohort of older men: The va normative aging study. Environ Health Perspect 125:76-81.

Crous-Bou M, Gascon M, Gispert JD, Cirach M, Sánchez-Benavides G, Falcon C, et al. 2020. Impact of urban environmental exposures on cognitive performance and brain structure of healthy individuals at risk for alzheimer's dementia. Environment international 138:105546.

Cullen B, Newby D, Lee D, Lyall DM, Nevado-Holgado AJ, Evans JJ, et al. 2018. Cross-sectional and longitudinal analyses of outdoor air pollution exposure and cognitive function in uk biobank. Scientific reports 8:1-14.

de Crom TO, Ginos BN, Oudin A, Ikram MK, Voortman T, Ikram MA. 2022. Air pollution and the risk of dementia: The rotterdam study. Journal of Alzheimer's Disease:1-11.

Fehsel K, Schikowski T, Janner M, Huls A, Voussoughi M, Schulte T, et al. 2016. Estrogen receptor beta polymorphisms and cognitive performance in women: Associations and modifications by genetic and environmental influences. J Neural Transm (Vienna) 123:1369-1379.

Gale SD, Erickson LD, Anderson JE, Brown BL, Hedges DW. 2020. Association between exposure to air pollution and prefrontal cortical volume in adults: A cross-sectional study from the uk biobank. Environmental Research 185:109365.

Gao Q, Zang E, Bi J, Dubrow R, Lowe SR, Chen H, et al. 2022. Long-term ozone exposure and cognitive impairment among chinese older adults: A cohort study. Environment International 160:107072.

Gatto NM, Henderson VW, Hodis HN, John JAS, Lurmann F, Chen J-C, et al. 2014. Components of air pollution and cognitive function in middle-aged and older adults in los angeles. Neurotoxicology 40:1-7.

Grande G, Wu J, Ljungman PLS, Stafoggia M, Bellander T, Rizzuto D. 2021. Long-term exposure to pm2.5 and cognitive decline: A longitudinal population-based study. J Alzheimers Dis 80:591-599.

He F, Tang J, Zhang T, Lin J, Li F, Gu X, et al. 2022. Impact of air pollution exposure on the risk of alzheimer's disease in china: A community-based cohort study. Environmental Research 205:112318.

Hedges DW, Erickson LD, Kunzelman J, Brown BL, Gale SD. 2019. Association between exposure to air pollution and hippocampal volume in adults in the uk biobank. Neurotoxicology 74:108-120.

Hedges DW, Erickson LD, Gale SD, Anderson JE, Brown BL. 2020. Association between exposure to air pollution and thalamus volume in adults: A cross-sectional study. PLoS One 15:e0230829.

Hu K, Hale JM, Kulu H, Liu Y, Keenan K. 2023. A longitudinal analysis of the association between long-term exposure to air pollution and cognitive function among adults aged 45 and older in china. The Journals of Gerontology: Series B 78:556-569.

Hu X, Nie Z, Ou Y, Qian Z, McMillin SE, Aaron HE, et al. 2022. Air quality improvement and cognitive function benefit: Insight from clean air action in china. Environmental Research 214:114200.

Iaccarino L, La Joie R, Lesman-Segev OH, Lee E, Hanna L, Allen IE, et al. 2021. Association between ambient air pollution and amyloid positron emission tomography positivity in older adults with cognitive impairment. JAMA neurology 78:197-207.

Kim H, Noh J, Noh Y, Oh SS, Koh S-B, Kim C. 2019. Gender difference in the effects of outdoor air pollution on cognitive function among elderly in korea. Frontiers in public health 7:375.

Kulick ER, Elkind MS, Boehme AK, Joyce NR, Schupf N, Kaufman JD, et al. 2020. Long-term exposure to ambient air pollution, apoe-ε4 status, and cognitive decline in a cohort of older adults in northern manhattan. Environment international 136:105440.

Lee JJ, Kim JH, Song DS, Lee K. 2022. Effect of short-to long-term exposure to ambient particulate matter on cognitive function in a cohort of middle-aged and older adults: Koges. International Journal of Environmental Research and Public Health 19:9913.

Lee M, Schwartz J, Wang Y, Dominici F, Zanobetti A. 2019. Long-term effect of fine particulate matter on hospitalization with dementia. Environmental Pollution 254:112926.

Li M, Ma Y-H, Fu Y, Liu J-Y, Hu H-Y, Zhao Y-L, et al. 2022. Association between air pollution and csf strem2 in cognitively normal older adults: The cable study. Annals of clinical and translational neurology 9:1752-1763.

Li Z, Christensen GM, Lah JJ, Marcus M, Russell AG, Ebelt S, et al. 2022. Neighborhood characteristics as confounders and effect modifiers for the association between air pollution exposure and subjective cognitive functioning. Environmental Research 212:113221.

Loop MS, Kent ST, Al-Hamdan MZ, Crosson WL, Estes SM, Estes Jr MG, et al. 2013. Fine particulate matter and incident cognitive impairment in the reasons for geographic and racial differences in stroke (regards) cohort. PloS one 8:e75001.

Lucht S, Glaubitz L, Moebus S, Schramm S, Jockwitz C, Caspers S, et al. 2022. Long-term air pollution, noise, and structural measures of the default mode network in the brain: Results from the 1000brains cohort. International journal of hygiene and environmental health 239:113867.

Ma H, Li X, Zhou T, Wang M, Heianza Y, Qi L. 2022. Long-term exposure to low-level air pollution, genetic susceptibility and risk of dementia. International Journal of Epidemiology.

Mortamais M, Gutierrez L-A, de Hoogh K, Chen J, Vienneau D, Carrière I, et al. 2021. Long-term exposure to ambient air pollution and risk of dementia: Results of the prospective three-city study. Environment international 148:106376.

Nunez Y, Boehme AK, Weisskopf MG, Re DB, Navas-Acien A, van Donkelaar A, et al. 2021. Fine particle exposure and clinical aggravation in neurodegenerative diseases in new york state. Environmental health perspectives 129:027003.

Oudin A, Andersson J, Sundström A, Nordin Adolfsson A, Oudin Åström D, Adolfsson R, et al. 2019. Traffic-related air pollution as a risk factor for dementia: No clear modifying effects of apoe ɛ4 in the betula cohort. Journal of Alzheimer's Disease 71:733-740.

Parra KL, Alexander GE, Raichlen DA, Klimentidis YC, Furlong MA. 2022. Exposure to air pollution and risk of incident dementia in the uk biobank. Environmental Research 209:112895.

Power MC, Weisskopf MG, Alexeeff SE, Coull BA, Avron IS, Schwartz J. 2011. Traffic-related air pollution and cognitive function in a cohort of older men. Environmental Health Perspectives 119:682-687.

Power MC, Weisskopf MG, Alexeeff SE, Wright RO, Coull BA, Spiro A, 3rd, et al. 2013. Modification by hemochromatosis gene polymorphisms of the association between traffic-related air pollution and cognition in older men: A cohort study. Environ Health 12:16.

Ran J, Schooling CM, Han L, Sun S, Zhao S, Zhang X, et al. 2021. Long-term exposure to fine particulate matter and dementia incidence: A cohort study in hong kong. Environ Pollut 271:116303.

Ranft U, Schikowski T, Sugiri D, Krutmann J, Krämer U. 2009. Long-term exposure to traffic-related particulate matter impairs cognitive function in the elderly. Environmental research 109:1004-1011.

Salinas-Rodríguez A, Fernández-Niño JA, Manrique-Espinoza B, Moreno-Banda GL, Sosa-Ortiz AL, Qian ZM, et al. 2018. Exposure to ambient pm2. 5 concentrations and cognitive function among older mexican adults. Environment international 117:1-9.

Schikowski T, Vossoughi M, Vierkotter A, Schulte T, Teichert T, Sugiri D, et al. 2015. Association of air pollution with cognitive functions and its modification by apoe gene variants in elderly women. Environ Res 142:10-16.

Semmens EO, Leary CS, Fitzpatrick AL, Ilango SD, Park C, Adam CE, et al. 2022. Air pollution and dementia in older adults in the ginkgo evaluation of memory study. Alzheimer's & dementia : the journal of the Alzheimer's Association.

Shaffer RM, Blanco MN, Li G, Adar SD, Carone M, Szpiro AA, et al. 2021. Fine particulate matter and dementia incidence in the adult changes in thought study. Environmental health perspectives 129:087001.

Shi L, Wu X, Yazdi MD, Braun D, Awad YA, Wei Y, et al. 2020. Long-term effects of pm2· 5 on neurological disorders in the american medicare population: A longitudinal cohort study. The Lancet Planetary Health 4:e557-e565.

Shi L, Steenland K, Li H, Liu P, Zhang Y, Lyles RH, et al. 2021. A national cohort study (2000–2018) of long-term air pollution exposure and incident dementia in older adults in the united states. Nature communications 12:6754.

Shi L, Zhu Q, Wang Y, Hao H, Zhang H, Schwartz J, et al. 2023. Incident dementia and long-term exposure to constituents of fine particle air pollution: A national cohort study in the united states. Proceedings of the National Academy of Sciences 120:e2211282119.

Shin J, Han S-H, Choi J. 2019. Exposure to ambient air pollution and cognitive impairment in community-dwelling older adults: The korean frailty and aging cohort study. International journal of environmental research and public health 16:3767.

Sun R, Gu D. 2008. Air pollution, economic development of communities, and health status among the elderly in urban china. Am J Epidemiol 168:1311-1318.

Tallon LA, Manjourides J, Pun VC, Salhi C, Suh H. 2017. Cognitive impacts of ambient air pollution in the national social health and aging project (nshap) cohort. Environment international 104:102-109.

Tzivian L, Dlugaj M, Winkler A, Hennig F, Fuks K, Sugiri D, et al. 2016a. Long-term air pollution and traffic noise exposures and cognitive function:A cross-sectional analysis of the heinz nixdorf recall study. J Toxicol Environ Health A 79:1057-1069.

Tzivian L, Dlugaj M, Winkler A, Weinmayr G, Hennig F, Fuks KB, et al. 2016b. Long-term air pollution and traffic noise exposures and mild cognitive impairment in older adults: A cross-sectional analysis of the heinz nixdorf recall study. Environ Health Perspect 124:1361-1368.

Tzivian L, Jokisch M, Winkler A, Weimar C, Hennig F, Sugiri D, et al. 2017. Associations of long-term exposure to air pollution and road traffic noise with cognitive function—an analysis of effect measure modification. Environment international 103:30-38.

Wang J, Li T, Lv Y, Kraus VB, Zhang Y, Mao C, et al. 2020. Fine particulate matter and poor cognitive function among chinese older adults: Evidence from a community-based, 12-year prospective cohort study. Environmental health perspectives 128:067013.

Wang X, Younan D, Millstein J, Petkus AJ, Garcia E, Beavers DP, et al. 2022. Association of improved air quality with lower dementia risk in older women. Proceedings of the National Academy of Sciences 119:e2107833119.

Wellenius GA, Boyle LD, Coull BA, Milberg WP, Gryparis A, Schwartz J, et al. 2012. Residential proximity to nearest major roadway and cognitive function in community‐dwelling seniors: Results from the mobilize boston study. Journal of the American Geriatrics Society 60:2075-2080.

Wu YC, Lin YC, Yu HL, Chen JH, Chen TF, Sun Y, et al. 2015. Association between air pollutants and dementia risk in the elderly. Alzheimers Dement (Amst) 1:220-228.

Wyatt LH, Cleland SE, Wei L, Paul N, Patil A, Ward-Caviness C, et al. 2023. Long-term exposure to ambient o3 and pm2. 5 is associated with reduced cognitive performance in young adults: A retrospective longitudinal repeated measures study in adults aged 18–90 years. Environmental Pollution:121085.

Yang L, Wan W, Yu C, Xuan C, Zheng P, Yan J. 2022. Associations between pm2. 5 exposure and alzheimer's disease prevalence among elderly in eastern china. Environmental Health 21:1-9.

Yao Y, Jin X, Cao K, Zhao M, Zhu T, Zhang J, et al. 2021. Residential proximity to major roadways and cognitive function among chinese adults 65 years and older. Sci Total Environ 766:142607.

Younan D, Wang X, Casanova R, Barnard R, Gaussoin SA, Saldana S, et al. 2021. Pm2. 5 associated with gray matter atrophy reflecting increased alzheimer risk in older women. Neurology 96:e1190-e1201.

Yu Y, Haan M, Paul KC, Mayeda ER, Jerrett M, Wu J, et al. 2020. Metabolic dysfunction modifies the influence of traffic-related air pollution and noise exposure on late-life dementia and cognitive impairment: A cohort study of older mexican-americans. Environ Epidemiol 4:e122.

Yuchi W, Sbihi H, Davies H, Tamburic L, Brauer M. 2020. Road proximity, air pollution, noise, green space and neurologic disease incidence: A population-based cohort study. Environmental Health: A Global Access Science Source 19:8.

Zhang Z, Chen L, Wang X, Wang C, Yang Y, Li H, et al. 2023. Associations of air pollution and genetic risk with incident dementia: A prospective cohort study. American Journal of Epidemiology 192:182-194.

Zhu A, Chen H, Shen J, Wang X, Li Z, Zhao A, et al. 2022. Interaction between plant-based dietary pattern and air pollution on cognitive function: A prospective cohort analysis of chinese older adults. Lancet Reg Health West Pac 20:100372.
